# A new class of anticancer activity with computational studies for a novel bioactive aminophosphonates based on pyrazole moiety

**DOI:** 10.1038/s41598-023-40265-8

**Published:** 2023-09-06

**Authors:** Mohamed H. Baren, Seham A. Ibrahim, Munirah M. Al-Rooqi, Saleh A. Ahmed, Mohammed M. El-Gamil, Hend A. Hekal

**Affiliations:** 1 Chemistry Department, Faculty of Science, Tanta University, Tanta, 31527 Egypt; 2 Department of Chemistry, Faculty of Applied Sciences, Umm Al-Qura University, Makkah, 21955 Saudi Arabia; 3 Department of Toxic and Narcotic Drug, Forensic Medicine, Mansoura Laboratory, Medico legal Organization, Ministry of Justice, Mansoura, Egypt

**Keywords:** Chemical biology, Computational biology and bioinformatics

## Abstract

The present study involves synthesis a new series of α-aminophosphonates **2a-f** and **4a-d** derivatives in good yield with a simple workup via Kabachnik-Fields reaction in the presence of lithium perchlorate as Lewis acid catalyst. All the newly synthesized compounds were confirmed using various physical, spectroscopic, and analytical data. The in vitro anticancer activities of each compound were evaluated against colorectal carcinoma Colon cancer (HCT-116) and Epdermoid carcinoma (HEP2) and also Human lung fibroblast normal cell line (WI38) compared with Doxorubicin. The results showed that Compounds **2a, 4b** and** 4d** exhibited more potent inhibitory activity for Epdermoid Carcinoma (HEP2) compared with doxorubicin. For colon carcinoma cells (HCT-116) Compounds **2a, 2d** and **4b** gave the strongest activity among all compounds compared with doxorubicin. Moreover, all designed structures were docked into the active site of VEGFR2 and FGFR1 proteins. The result reveals that compound **2b** and have the strongest inhibitory activity of the VEGFR2 and FGFR1 proteins indicating that these substances might conceivably operate as VEGFR2 and FGFR1 inhibitors and hence might take role in anticancer activities with various binding interactions. The 3D-QSAR models produced strong statistical results since they were defined by PLS factors 4 and confirmed by parameters as R2, R2 CV, Stability, F-value, P-value, RMSE, Q2, and Pearson-r.

## Introduction

**Figure 1:**
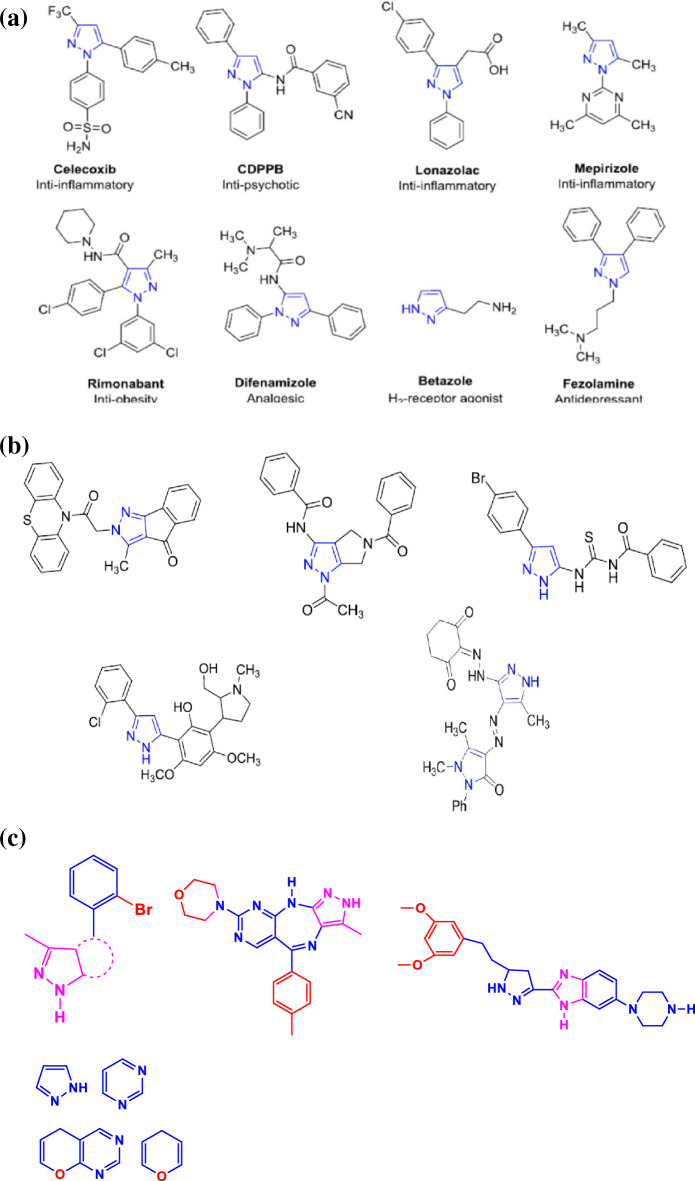
(**a**) Represented FDA-approved drugs containing a pyrazole nucleus. (**b**) Pyrazole derivatives showing anticancer activity. (**c**) Pyrazole derivatives showing VEGFR-2, FGFR1 inhibitory activities.

At least half of all organic chemistry research worldwide is focused on heterocyclic chemistry, which is also known as the major area of classical organic synthesis^1^. Heterocyclic compounds play a vitally important role in medicinal chemistry and are receiving healthy funding with a wider range of potential for processing and synthesizing a wide variety of pharmacological characteristics. Along with being widely present in natural products^2^. There are numerous therapeutic applications for heterocyclic compounds, including pharmacological and medicinal properties^3–13^. Pyrazole-containing compounds represent one of the most significant families of N-heterocycles. Due to their shown utility and adaptability as synthetic intermediates in the synthesis of key molecules in biological, physical–chemical, material science, and industrial applications, As a result, it is extremely desirable to synthesize pyrazole derivatives with a variety of structural characteristics, and numerous researchers are still working to create this useful scaffold and discover new and better uses for it. Moreover, The pyrazole moiety has been shown to have pharmacological potential by its presence in a variety of therapeutic agents, including the antipsychotic CDPPB, the anti-obesity medication rimonabant, the analgesic difenamizole, the H2-receptor agonist betazole, and the antidepressant fezolamide (Fig. 1a)^14, 15^.

According to literature survey, a series of some new pyrazolyl derivatives were synthesized and investigated for their antitumor activity against different cancer cell lines. These results indicated that the pyrazolyl derivatives displayed antiproliferative activity in a range of human tumor cell lines, including HCT116 human colon carcinoma and Epdermoid Carcinoma (HEP2), Epdermoid Carcinoma (HEP2) and also Human lung fibroblast normal cell line (WI38) besides different tumor cell lines. For the moment, researchers have been drawn to the design of more potent pyrazole derivatives having great diversity of biological activity (Fig. 1b)^14^. Moreover, the literature survey results introduce novel pyrazole-based moieties as dihydro-pyrano-pyrazole and pyrazolo-pyrimidine derivatives with 2-bromophenyl moiety as promising scaffolds to produce potent VEGFR-2 inhibitors. Additionally, over the last decade, the family of the fibroblast growth factor receptors (FGFRs) has become an attractive validated therapeutic target notably in cancer diseases.

A series of novel pyrazole-benzimidazole derivatives was identified as a selective and potent pan-FGFR inhibitor. pyrazolo[4,3-b]pyrimido[4,5-e][1,4]diazepine scaffold was identified as a selective and potent pan-FGFR and VEGFR-2 inhibitors(Fig. 1c)^16–18^.

The biological and therapeutic properties of 5-amino-pyrazoles as well as their flexibility in synthetic processes have recently drawn a lot of attention and given them a prominent position^19^. The -NH_2_ functionality makes 5-amino-pyrazoles one of a class of adaptable aromatic heterocycles containing nitrogen that have a high degree of controllability for creating different synthesis schemes. α-Aminophosphonates (AAPs) are most important group of organophosphorus compounds that are structural analogues of amino acids where a carboxylic substituent is substituted by phosphonic acid or related groups^20^. Recently, α-aminophosphonates have received enormous attention from scientific researchers in pharmaceutical and medicinal chemistry due to they have extremely high levels of wide biological activities such as anti-HIV, peptidemimics, antibacterial, antibiotics, inhibitors of serine hydrolase, herbicidal, antiviral, anticancer, enzyme inhibitors, antifungal, and anti-proliferative, enzyme inhibitors, inhibitors of serine hydrolase anti-Alzheimer and apoptosis inducing^21, 22^. Moreover, α-aminophosphonate derivatives containing a pyrazole moiety showed a significant inhibitory effect on acetylcholinesterase (AChE)^23^. Vascular endothelial growth factor (VEGF) is a key signalling molecule that controls the tumor angiogenesis process. VEGF overexpression was discovered in a number of cancer, including Epdermoid carcinoma (HEP2) and Colon cancer (HCT-116). All VEGF responses in endothelial cells are mediated by VEGFR-2. Therefore, VEGFR-2 should be the primary target of any new drugs being developed to treat cancers in humans that are dependent on angiogenesis^24^.

Various methods for the synthesis of α-aminophosphonates were reported. However, one pot Kabachnik-Fields reaction, a one-pot multicomponent synthesis involving an amine, an aldehyde, and a phosphite in the presence of a Lewis acid catalyst remains the most efficient, simple, general, and high yielding method^25^.

Based on these facts and keeping in view the wide range of biological activities of pyrazole moieties, and aminophosphonate scaffolds, in this work, we expect that the incorporation of these moieties in the same scaffold structure may lead to good activities and potent powerful anticancer medicines. Thus, as a continuation of our prior work in the synthesis of biologically active heterocycles^26–28^, we have designed and synthesized a series of new α-aminophosphonates derivatives bearing pyrazole moiety and were evaluated against references and cancer cell lines. Additionally, the newly synthesized compounds’ biological activity was examined in connection to changes in their molecular and electronic structure using density functional theory in an effort to connect theoretical and experimental results. To determine the target enzyme and the most active compound's mechanisms of action, a molecular docking simulation will be run.

## Results and discussion

### Synthesis of aminophosphonates and spectroscopic characterization

**Figure 2:**
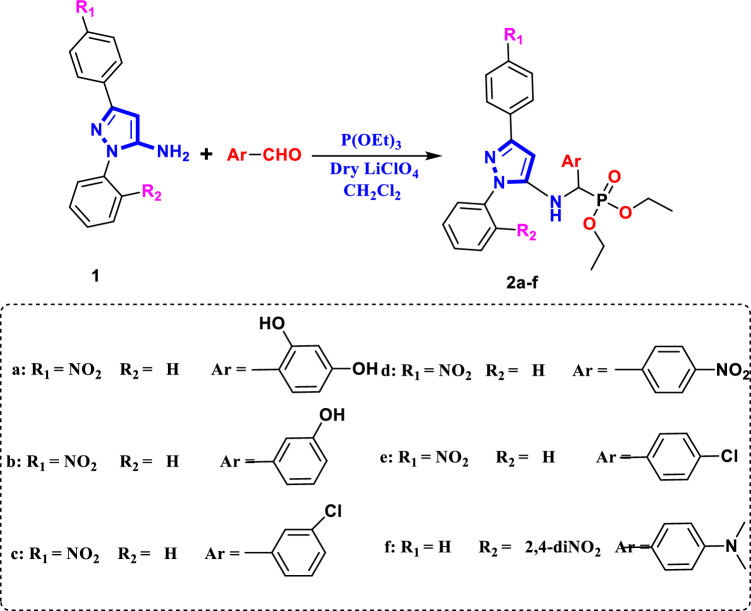
Synthesis pathway of compounds **2a-f**.

**Figure 3:**
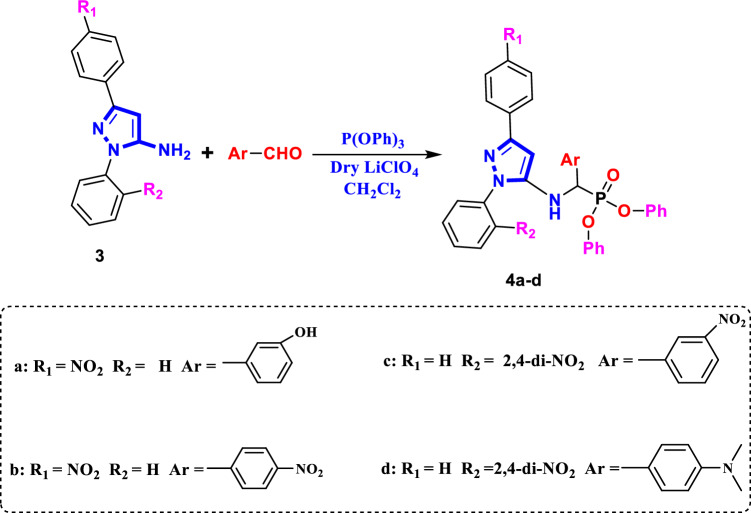
Synthesis pathway of compounds **4a-d**.

A new series of α-aminophosphonates bearing pyrazole skeleton were synthesized via three component Kabachnic-Fields reaction of 3-(4-nitrophenyl)-1-phenyl-1H-pyrazol-5-amine **1**/or 3-(4-nitrophenyl)-1-phenyl-1H-pyrazol-5-amine **2**, different aromatic aldehydes, triethylphosphite/or triphenylphosphite in CH_2_Cl_2_ in the presence of lithum perchlorate as a Lewis acid catalyst via one-pot Kabachnik–Fields^25^, gives Diethyl{(3-(Aryl)-1-phenyl-1H-pyrazol-5-ylamino)(Aryl′)} methyl phosphonate **2a-f** and Diphenyl{(1-(Aryl)-3-phenyl-1H-pyrazol-5-ylamino)(Aryl′)} methyl phosphonate **4a-d**, respectively, in a very good yield (Figs. 2 and 3). The structures of investigated α-aminophosphonates **2a-f** and **4a-d**, were confirmed by corrected elemental analysis, FT-IR, ^1^H NMR and ^13^C-NMR spectroscopy. The FT-IR spectrum was characterized by the following absorption bands: A band at 1267–1193 cm^–1^ is attributed to the stretching vibration of P=O group. υ (P–O–C) appeared at 1104–1011 cm^–1^ and υ (P–CH) is absorbed at 765–701 cm^–1^.The CH aromatic stretching band is absorbed at 3162–3025 cm^–1^, while the CH aliphatic bands are appeared at 2982–2853 cm^–1^. Finally, NH/OH groups are absorbed at 3464–3413 cm^–1^.

The ^1^H-NMR (DMSO) spectra of α-aminophosphonate derivatives, **2a-f**, showed the following signals: δ 5.02–5.76 as singlet was assigned to P–CH and the singlet signals at the range of δ 6.23–7.12 were attributed to pyrazole–CH. The Ar–H signals appeared at the range of δ 6.32–9.26 ppm, also the –NH proton appeared at δ 5.61–5.99 and signals at δ 6.42–7.21 were attributed to pyrazole–CH. The methylene and methyl protons of P–O–CH_2_CH_3_ resonated as quarter and triplet respectively at δ 3.62–4.39 and 1.26–1.39 ppm. The ^13^C-NMR (DMSO) of the studied compounds are shown as the following signals at δ 146.42–167.94 and 143.32–158.29 (C=N_imine_) and (C=N pyrazole ring), respectively, 139.33–148.97 (C–NO_2_), 54.04–66.82 (P–CH _aliphatic_), 81.93–89.39 (CH_pyrazole_), 114.35–148.34 (C_Aromatic_). Additionally, a peak at m/z corresponding to the product’s molecular ion was visible in the mass spectrum for the all product. By using elemental microanalysis to characterize the examined substances, it was found that the calculated value and the measured value agreed well. The selected spectroscopic data are reported in the (“Experimental” section) (Figs. S1–S30 in supplementary materials**)**.

### Biological evaluation

#### Antitumor activity

Nitrogen-containing heterocycles exhibit anticancer effect in various types of cancer through inhibiting cell growth and induction of cell differentiation and apoptosis.

Moreover, therapeutic drugs that inhibiting EGFR and VEGFR-2 can enhance the effectiveness of cancer therapy and resolve resistance issues^29^.

**Figure 4:**
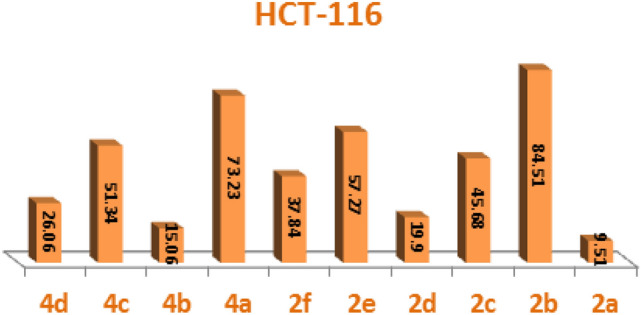
IC_50_ of compounds **2a-f** and **4a-d**, against colon carcinoma cells (**HCT-116**).

**Figure 5:**
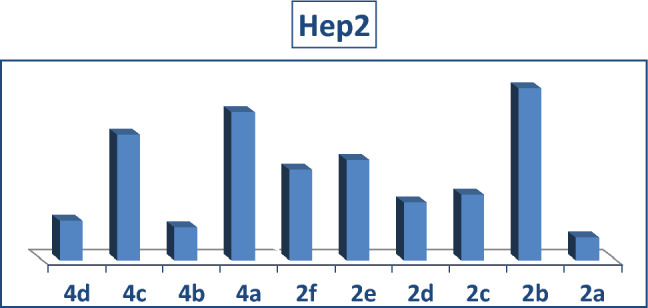
IC_50_ of compounds, **2a-f** and **4a-d**, against Epdermoid Carcinoma (**HEP2**).

**Figure 6:**
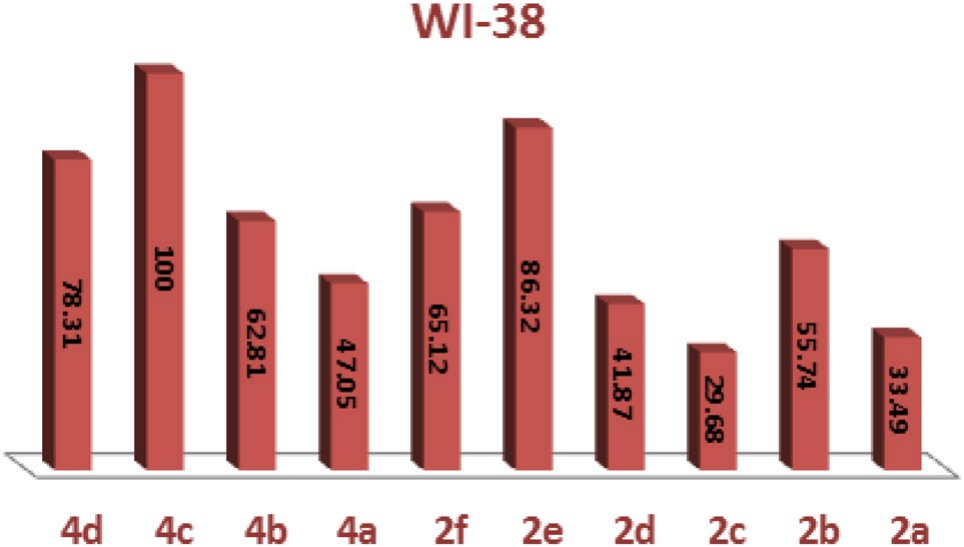
IC50 of compounds **2a-f** and **4a-d**, against normal cell line (**WI38**).

To create novel molecules with high inhibitory potentials, pyrazole derivatives were widely utilized^30^.The cytotoxic potency of the synthesized phosphonates, **2a-f** and **4a-d** was determined in vitro using the standard MTT method^31^ against Colorectal carcinoma Colon cancer (HCT-116) and Epdermoid Carcinoma (HEP2) and also Human lung fibroblast normal cell line (WI38), using Doxorubicin as a positive control. The IC_50_ values were estimated for each compound and the results are shown in (Figs. 4, 5 and 6) and summarized in (Table 1). Compounds **2a**, **4b** and **4d** gave the highest activity for Epdermoid Carcinoma (**HEP2**), while the rest compounds have moderate to weak activity against the same cell line as shown in Table 1. For colon carcinoma cells (**HCT-116**) Compounds **2a, 2d** and **4b** gave the strongest activity among all compounds. Finally, all the studied phosphonate compounds have moderate to weak cytotoxicity activity on the normal cell WI-38.

**Table 1:** Cytotoxic activity in compounds **2a-f** and **4a-d**, against 2 human tumor cells and 1 normal cell.

Compound	In vitro cytotoxicity IC_50_ (µM)*		
	83IW	611CCH	Hep2
XOD	570 ± H776	57I ± 076I	57H ± W708
6a	678 ± II783	577 ± 370C	573 ± C6760
2b	I7C ± 00778	876 ± W870C	07C ± 3I7I8
2c	676 ± 637HW	67W ± 807HW	670 ± I073I
2d	677 ± 8C7W7	C7H ± C3735	67I ± IC7H0
2e	878 ± WH7I6	I78 ± 07767	I76 ± 087I7
2f.	I7H ± H07C6	678 ± I77W8	673 ± 837C5
4a	673 ± 87750	I73 ± 7I76I	876 ± W57IC
4b	I70 ± H67WC	C7C ± C075H	C78 ± C777H
4c	C55 >	I75 ± 0C7I8	I70 ± HW7CH
4d	I73 ± 7W7IC	C73 ± 6H75H	C7W ± 6C76W

*IC50 (µM): 1–10 (very strong), 11–20 (strong), 21–50 (moderate), 51–100 (weak) and above 100 (non-cytotoxic), *DOX* doxorubicin.

From the above data, we could conclude that compound **2a** which contain 2, 3-diOH electron donating substituent and compounds **2d** and **4b** which have electron withdrawing NO_2_ group, are the most potent derivatives against the tested cancer cell lines and also have low cytotoxic activity on the normal cell line (Table 1).

#### Structure–activity relationship (SAR) of studied compounds 2a-f and 4a-d

The experimental cytotoxicity of the investigated compounds to their structures was used to postulate a structure–activity relationship of the produced α-aminophosphonate derivatives:

(i) The variety of substituents in the aryl aldehyde moiety of AAPs is important for the wide range of cytotoxic activity against different cell lines (HCT-116, Hep2 and WI-38), (Figs. 4, 5 and 6).
(ii) Compound **2b** which has one OH group in position **3** in the aryl aldehyde moiety is in more active compound towards the cell lines, but compound **2a** which has 2-OH groups in position **2** and **3** in the aryl aldehyde moiety enhanced the cytotoxic activity and showed a medium cytotoxic activity against the normal lung cell WI-38 compared with doxorubicin which has OH moieties.
(iii) Compound **2c** which has one Cl group in position **3** in the aryl aldehyde moiety enhance the biological activity against the tested cell lines.
(iv) The di-ethyl phosphonate, **2d**, and the di-phenyl phosphonate compound **4b** which have electron withdrawing substituent NO_2_ in the aryl aldehyde moiety have potent cytotoxic activity against the cell line among all compounds.
(v) The di-phenyl phosphonate compound **4d** which has electron donating group N(CH_3_) in the aryl substituted aldehyde moiety is more active than the di-ethyl phosphonate of the same substituent **2f** which is may be due to the increasing number of phenyl rings which increase the resonance and accordingly increase the antitumor activity.
(vi) The presence of two electron donating OH group in compounds **2a** improves potency more than compound **2d** which have an electron withdrawing group NO_2_ (Table 1).

### Molecular computational calculation

#### Geometry optimization and global reactivity descriptors using DFT

**Figure 7:**
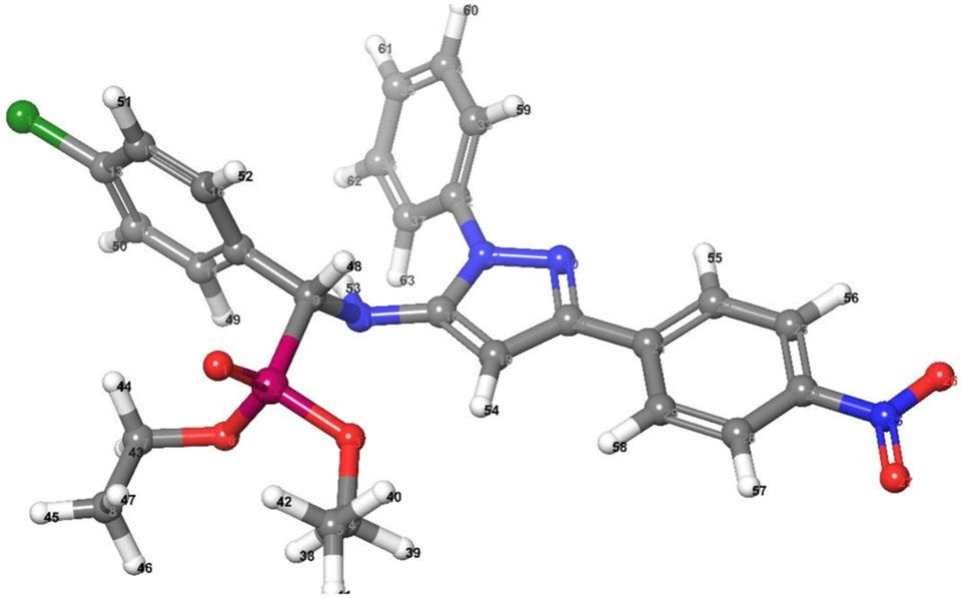
Optimized molecular structure of **2e**.

Figure 7 and Fig. S31 in supplementary materials illustrate the molecular structure and atom numbering of the examined compounds. One can come to the conclusion that the values of bond lengths and angles are close to the actual values based on the analysis of the data generated for bond lengths and angles (Tables S1–S20 in supplementary materials).

**Figure 8:**
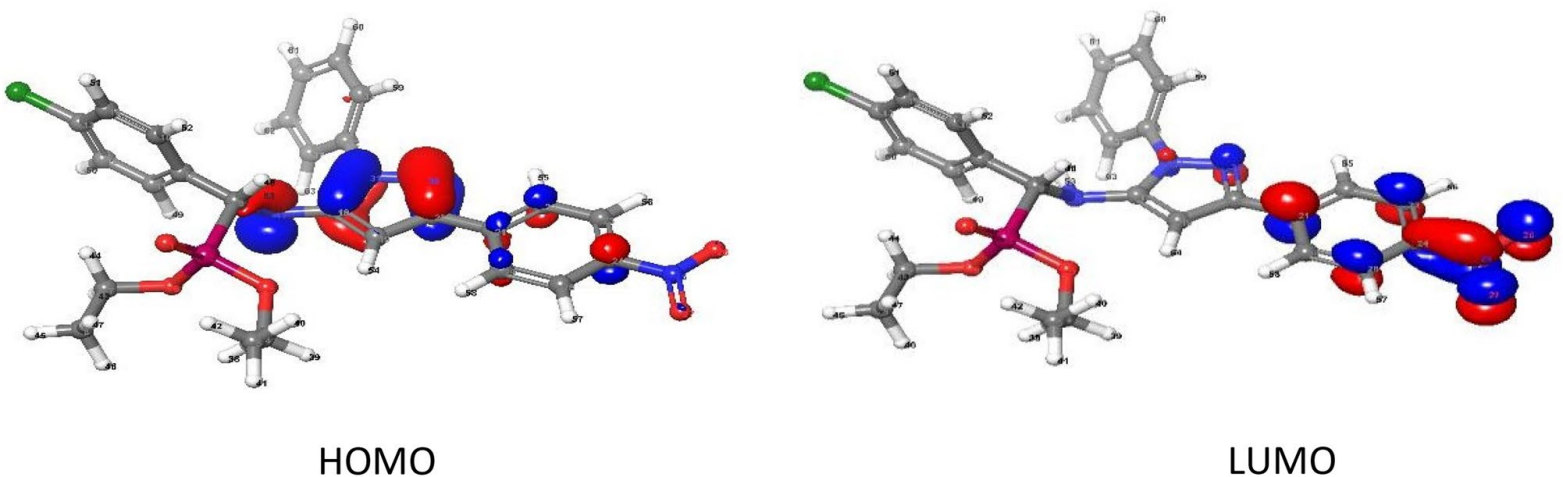
3D plots frontier orbital energies HOMO and LUMO using DFT method for **2e**.

The “HOMO energy level” that occupies the highest molecular orbital is essentially an electron donor molecular orbital. The lowest unoccupied molecular orbital, or “LUMO”, is mostly used as an electron acceptor. Both orbitals were called frontier molecular orbitals (FMOs) (Fig. 8 and Figs. S32 and S33 in supplementary materials) have evaluated kinetic stability, electronic transitions, and electro-optical properties^32^. The FMOs theory proposed that aromatic compounds have a coordination site (electrophilic attack). Furthermore, most reactions are caused by the interaction of one moiety's HOMO and another’s LUMO.

Table 2 displays the energy gap (Δ*E* = *E*_HOMO_*—E*_LUMO_), and chemical descriptors for the examined compounds. These descriptors can be assessed using the formulae provided by Pearson, and Padmanabhan et al.^33, 34^.

**Table 2:** Calculated gas phase energy, and energetic descriptors for investigated compound.

Compound	Gas phase energy	*E*_H_	*E*_*L*_	(*E*_H_ *-E*_L_)	*Χ*	µ	*η*	*S*	*ω*	Ϭ	∆Nmax	Dipole
	(Kcal/mol)	(eV)	(eV)	(eV)	(eV)	(eV)	(eV)	(eV^–1^)	(eV)	(eV)	(e)	(debye)
2a	− 2.09 × 10^03^	− 5.5368	− 2.1340	3.403	3.835	− 3.835	1.701	0.851	4.323	0.588	2.2543	11.735
2b	− 2.02 × 10^03^	− 5.6493	− 2.2011	3.448	3.925	− 3.925	1.724	0.862	4.468	0.580	2.2766	10.703
2c	− 2.40 × 10^03^	− 5.7892	− 2.2519	3.537	4.021	− 4.021	1.769	0.884	4.570	0.565	2.2732	8.202
2d	− 2.15 × 10^03^	− 5.9575	− 2.7981	3.159	4.378	− 4.378	1.580	0.790	6.066	0.633	2.7713	5.145
2e	− 2.40 × 10^03^	− 6.0750	− 2.2486	3.826	4.162	− 4.162	1.913	0.957	4.527	0.523	2.1753	8.256
2f.	− 2.28 × 10^03^	− 5.2973	− 3.1307	2.167	4.214	− 4.214	1.083	0.542	8.196	0.923	3.8901	5.439
4a	− 2.32 × 10^03^	− 5.5697	− 2.1003	3.469	3.835	− 3.835	1.735	0.867	4.239	0.576	2.2108	12.066
4b	− 2.45 × 10^03^	− 6.1983	− 2.6910	3.507	4.445	− 4.445	1.754	0.877	5.633	0.570	2.5345	4.465
4c	− 2.53 × 10^03^	− 5.9731	− 2.9909	2.982	4.482	− 4.482	1.491	0.746	6.736	0.671	3.0058	6.499
4d	− 2.59 × 10^03^	− 5.3781	− 2.9557	2.422	4.167	− 4.167	1.211	0.606	7.168	0.826	3.4404	8.026

The additional electronic charge is derived by the formula provided by Geerlings et al. and Pearson^33, 34^. and is equal to the maximum number of electrons moved in the chemical process (ΔN_max_).

Using the computation findings provided in Table 2:

- The gas phase energy decreases with the order: **2b > 2a > 2d > 2f > 4a > 2e > 2c > 4b > 4c > 4d**. This order indicates that the stability of **4d** is higher than that of other compounds.
- The *E*_HOMO_ and *E*_LUMO_ values are both negative, suggesting that the isolated compounds are stable^35, 36^.
- Hard molecules have larger HOMO–LUMO disparities, whereas soft and active molecules have smaller energy differences. The chemical potential ‘μ’ which quantifies electrons’ ability to escape from the equilibrium structure, is decreased as follows: **4a (− 3.835 eV) > 2a (− 3.835 eV) > 2b (− 3.925 eV) > 2c (− 4.021 eV) > 2e (− 4.162 eV) > 4d (− 4.167 eV) > 2f (− 4.214 eV) > 2d (– 4.378 eV) > 4b (− 4.445 eV) > 4c (− 4.482 eV)**.
- ΔN_max_ is the reaction index, which gauges the bond energy’s stability. The computation of ΔN_max_ reveals that **2f** has the greatest value (3.8900 e), which is greater than other compounds, showing its high electron acceptance.
- The dipole moment value of the **4a** is greater than other compounds’ values which may increase its hydrophilic nature and, as a result, its biological potency.

### Computation of vibrational frequency

A frequency calculation study was performed to determine the spectroscopic signature of substances (Fig. S34 in supplementary materials). Because the calculations were conducted for free molecules in a vacuum whereas the tests were performed for solid samples, there are modest discrepancies in theoretical and practical vibrational wavenumbers as displayed in (Fig. S34 in supplementary materials). Because of the poor symmetry of compounds, the modes of vibration are extremely complicated. Because of mixing with ring modes and substituent modes, in/out of plane, and torsion manners are the hardest to assign. Though, there are several strong frequencies in the IR spectrum that are important to define (Fig. S35 in supplementary materials) depicts the correlation graphic that described the agreement between theoretical and experimental wavenumbers. Table 3 shows the linear relationships between computed and experimental wavenumbers for all substances. For each figure, the correlation coefficient R^2^ was obtained, where R^2^ is a statistical number indicating how closely the regression line approximates the true data points. As a result, a good connection was discovered for the examined chemicals, as shown in (Fig. S35 in supplementary materials).

**Table 3:** Comparison of experimental and theoretical IR spectra of investigated compounds.

Comp. no	(FT–IR (cm^–1^))							Intercept	Slope	R^2^
	Method	νNH	H-νArom.	H-νAliph.	νP= O	C–O–vP	CH-νP			
2a	Exp.	3248	3029	2925	1254	1086	761	− 58.81 ± 128.60	0.025 ± 1.11	0.99734
	Calc.	3572	3201	3049	1270	1049	748			
2b	Exp.	3413	3025	2917	1265	1006	700	− 22.77 ± 34.05	0.009 ± 1.06	0.99957
	Calc.	3593	3201	3050	1271	1051	726			
2c	Exp.	3435	3029	2982	1193	1104	758	− 50.81 ± 80.41	0.021 ± 1.06	0.99793
	Calc.	3545	3203	3059	1256	1041	712			
2d	Exp.	3430	3068	2853	1230	1083	764	− 42.92 ± 91.13	0.018 ± 1.08	0.99855
	Calc.	3578	3207	3066	1235	1040	758			
2e	Exp.	3447	3162	2922	1239	1069	765	− 33.49 ± 42.90	0.014 ± 1.04	0.99908
	Calc.	3565	3205	3059	1262	1066	750			
2f.	Exp.	3422	3065	2919	1267	1011	701	− 35.82 ± 6.28	0.015 ± 1.029	0.99889
	Calc.	3520	3198	2997	1255	1043	773			
4a	Exp.	3432	3032	–	1234	1015	701	− 11.70 ± 26.63	0.005 ± 1.06	0.9999
	Calc.	3598	3205	–	1275	1052	717			
4b	Exp.	3432	3065	2925	1235	1025	765	− 22.55 ± 29.23	0.010 ± 1.04	0.99962
	Calc.	3543	3206	–	1238	1052	781			
4c	Exp.	3434	3063	2924	1237	1064	764	− 15.12 ± 83.96	0.006 ± 1.07	0.99984
	Calc.	3607	3202	–	1222	1057	755			
4d	Exp.	3464	3025	2921	1252	1080	732	− 49.80 ± 48.13	0.021 ± 1.04	0.99795
	Calc.	3556	3198	2987	1220	1057	773			

### Electrostatic potential (ESP) and average local ionization energy (ALIE) properties on the molecular surface

Electrostatic potential *V(r)* and average local ionization energy $\overline{I }$*(r)* of the investigated compounds have been demonstrated to be dynamic guides to its reactive performance^37^.

**Figure 9:**
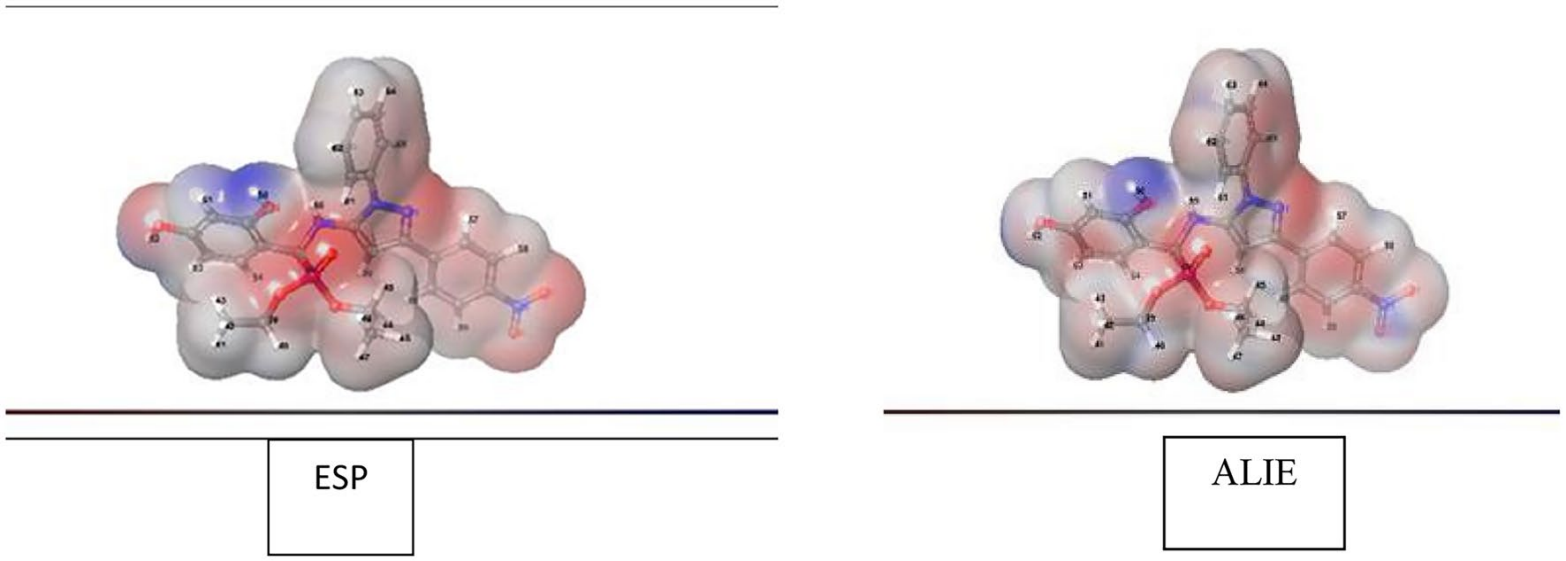
The surface structure of ESP and ALIE using DFT method for **2a**.

Figure 9 and supplementary material (Figs. S36 and S37) depicted the electrostatic potential V(r) and average local ionization energy $\overline{\mathrm{I} }$(r) of all compounds. Also, computed molecular surface data recorded in Table 3 and the following parameters are listed in this table:

- The most positive *V*_S*,*max_ and the most negative *V*_S*,*min_.
- The whole surface potential value $\overline{V }$_*S*_, with its positive averages ${\overline{V} }_{s}^{+}$ and negative averages ${\overline{V} }_{s}^{-}$.
- Most positive $\overline{I }$_S,max_ and most negative $\overline{I }$_S,min*,*_ and the average over the surface of the local ionization energy $\overline{I }$_S,ave*.*_
- Internal charge transfer (local polarity) Π, is derived as a sign of internal charge separation and is present even in molecules with zero dipole moment due to symmetry.
- The variances,${\sigma }_{+}^{2}$, ${\sigma }_{-}^{2}$ and ${\sigma }_{tot}^{2}$ describe the intensities and variations of the positive, negative, and overall surface potentials, respectively^38^.
- An electrostatic balancing parameter ν, that indicates the degree of equilibrium between positive and negative potentials; it has a maximum value of 0.25 when ${\sigma }_{+}^{2}$= ${\sigma }_{-}^{2}$.

Table 4 shows that **2a** has the largest Π with value 12.52 kcal/mol followed by **2b**. This is because to their strong polar structures. While **2c** exhibits lowest value of Π = 10.16 kcal/mol and may be related to its structural symmetry.

**Table 4:** Computed molecular surface properties (ESP) and (ALIE).

Compound	*V*_*s, min*_	*V*_*s, max*_	$\overline{V }$_*S*_	${\overline{V} }_{s}^{+}$	${\overline{V} }_{s}^{-}$	${\sigma }_{+}^{2}$	${\sigma }_{-}^{2}$	${\sigma }_{tot}^{2}$	ν	Π	$\overline{I }$_*S, min*_	$\overline{I }$_*S, max*_	$\overline{I }$_*S*_	$\overline{I }$_*S, ave*_
2a	− 39.10	61.78	0.78	12.33	− 12.84	117.43	107.86	225.29	0.250	12.52	189.27	381.58	267.27	22.79
2b	− 38.02	58.37	0.79	11.52	− 12.58	73.07	102.34	175.41	0.243	11.92	192.16	375.08	267.06	21.05
2c	− 37.36	28.3	1.46	10.11	− 10.69	38.38	95.32	133.69	0.205	10.16	193.92	351.37	266.53	20.70
2d	− 36.12	34.45	3.44	11.88	− 12.01	44.80	93.45	138.25	0.219	11.18	198.19	366.83	273.56	20.49
2e	− 37.86	28.56	2.42	11.06	− 11.66	41.59	115.89	157.48	0.194	10.88	197.39	350.82	267.00	20.99
2f.	− 36.81	29.96	1.08	10.23	− 13.03	33.47	67.45	100.92	0.222	11.12	200.02	372.03	270.14	21.46
4a	− 39.53	60.9	0.56	11.61	− 11.61	75.18	93.02	168.20	0.247	11.59	190.00	380.37	264.67	23.28
4b	− 35.03	37.36	3.99	12.1	− 11.68	48.15	90.56	138.71	0.227	11.04	205.19	363.89	272.38	21.14
4c	− 38.51	60.02	1.09	12.16	− 11.48	90.48	66.21	156.68	0.244	11.80	198.28	378.52	268.13	22.92
4d	− 31.64	44.58	1.45	11.6	− 11.48	49.65	57.73	107.38	0.249	11.42	199.24	367.48	267.99	21.19

Units: *V*_s, min,_ *V*_s, max,_ $\overline{V }$_S,_${\overline{V} }_{\mathrm{s}}^{+}$, ${\overline{V} }_{\mathrm{s}}^{-}$, Π, $\overline{I }$_S, min,_ $\overline{I }$_S, max,_ $\overline{I }$_S_ and $\overline{I }$_S, ave_ are in kcal/mol, ${\sigma }_{+}^{2}$, ${\sigma }_{-}^{2}$, ${\sigma }_{\mathrm{tot}}^{2}$ are in (kcal/mol)^2^; ν is unitless.

The positive surface potential (${\overline{V} }_{s}^{+}$) and negative surface potential (${\overline{V} }_{s}^{-}$) of **2a** are strong with balanced with ν = 0.250. While, the weaker surface potentials with ν = 0.194 is related to **2e**. Furthermore, the largest value of ${\sigma }_{tot}^{2}$ for **2a** shows the strong and variable + *ve* and *-ve* surface potentials.

Figure 9, is presented the *V*_S_(r) and $\overline{I }$_S_(r) on surfaces of **2a** as a representative example and shows the locations of the various most positive, *V*_S*,*max_ and most negative, *V*_S*,*min_ as well as the highest, $\overline{I }$_S,max_ and lowest, $\overline{I }$_S,min_. On a particular molecular surface, there are frequently many local minima and maxima of each attribute. The highest negative electrostatic potential on the surface of **2a** is related to the oxygen (O27), *V*_S,min_ = − 39.1 kcal/mol, followed by a weaker value of − 39.07 kcal/mol on the oxygen (O28). As a result, *V*_*S*_*(r)* incorrectly guess electrophilic attack to arise specially at the oxygen atom. However, the lowest value of $\overline{I }$_*S*_*(r)* is found on the (C20), with $\overline{I }$_S,min_ = 189.27 kcal/mol; as well as, there is an $\overline{I }$_S,min_ by the nitrogen (N31), but it is significantly higher, 198.28 kcal mol^–1^. Thus, $\overline{I }$_S_(r) demonstrates that the least-tightly-bound electrons, and most reactive are at (C20), correctly suggesting that these sites are more vulnerable to electrophiles. On contrast, the extremely significantly positive electrostatic potential of hydrogen (H50), *V*_S,max_ = 61.78 kcal/mol, and the *V*_S,min_ = 7.16 kcal/mol of the hydrogen (H41) show their proclivity for noncovalent H-bonding as a donor.

### Molecular docking

#### Molecular docking with VEGFR‐2 and FGFR1 proteins

**Figure 10:**
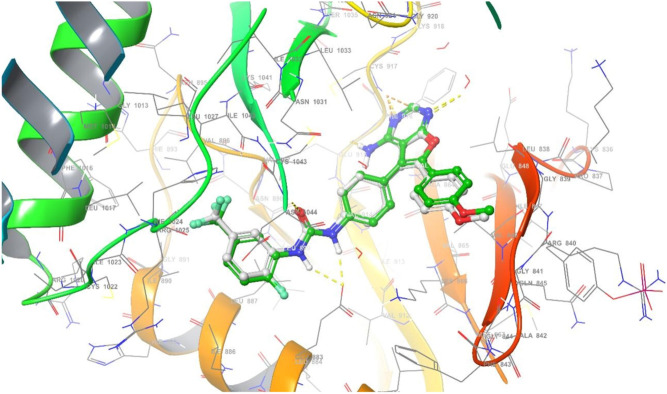
Superimposition of re-docked inhibitor (green) onto co-crystallized complex (grey) in the active site (RMSD = 0.792 Å) towards VEGFR‐ 2 receptor.

**Figure 11:**
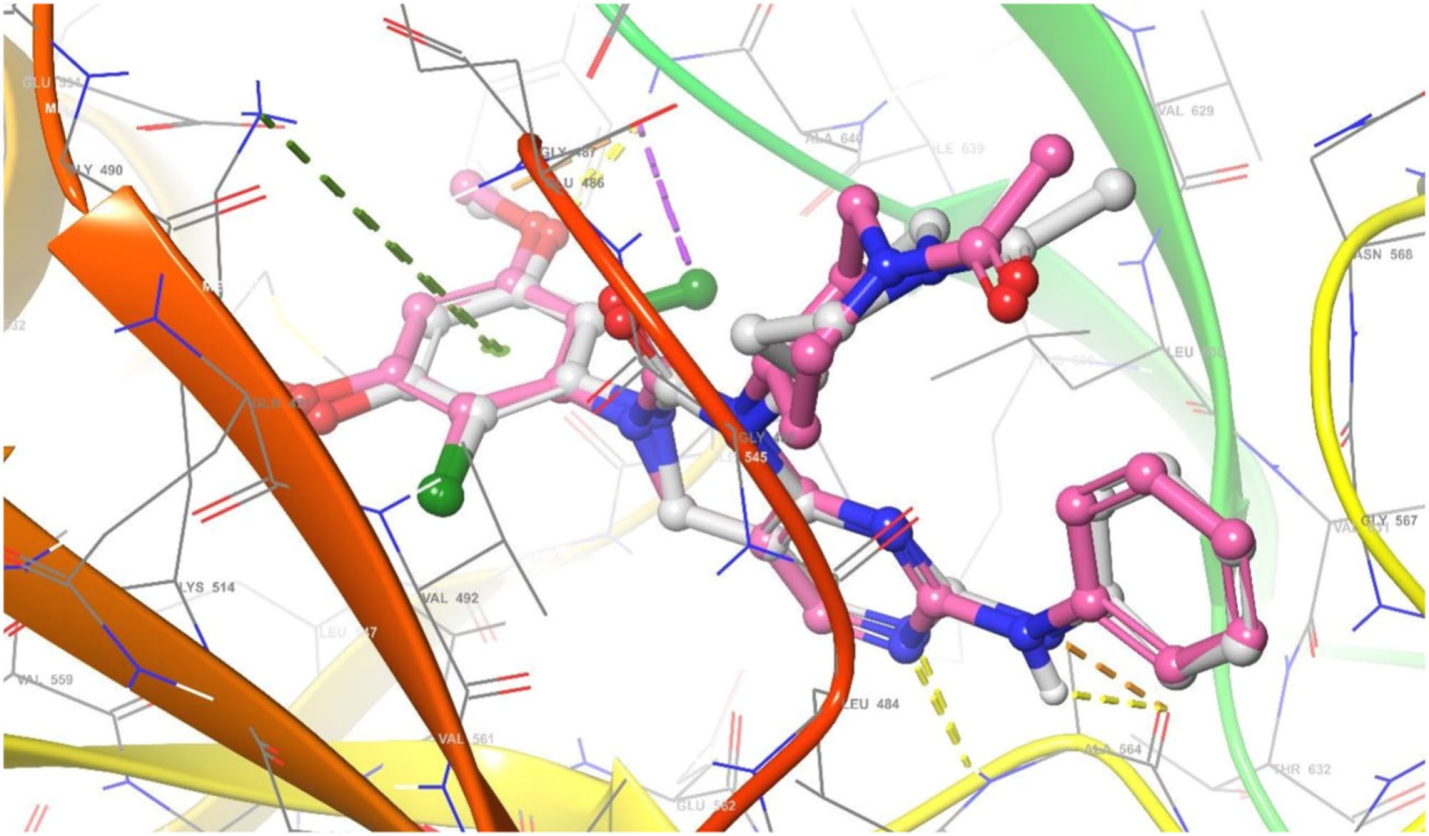
Superimposition of re-docked inhibitor (violet) onto co-crystallized complex (grey) in the active site (RMSD = 0.618 Å) towards FGFR1 receptor.

The relevance of molecular docking in drug development is well acknowledged. According to the Glide S-score, the Maestro programme was used to dock the most extensive docking pocket and hits (Tables 5 and 6). Higher Glide S-score and lower RMSD values were used to identify the best docking hit compounds. Thus, the results in Table 5, reveals that the standard drug Doxorubicin exhibits the highest inhibitory activity against both proteins. Furthermore, the compounds **2b** and **2a** have the strongest inhibitory activity of the VEGFR2 and FGFR1 proteins, respectively. The sequence of inhibitory activity to the VEGFR2 protein is **2b > 2f > 4a > 2a > 4b > 4d > 2e > 2c > 2d**. While the sequence of inhibitory action to the FGFR1 protein is **2a > 2b > 2f > 4d > 4a.** Furthermore, **4c** doesn’t exhibit any interactions with the VEGFR‐2 protein. Also compounds **4c, 2e, 2d, 4b**, and **2c** don’t show any interactions with the FGFR1 protein. From these data, one can notice that compound **4c** has no interaction with FGFR1 protein and agree with experimental data of in vitro cytotoxicity against WI38 (IC_50_ > 100). The examined substances demonstrate that H-donor and H-acceptor interactions are the most common kind of interaction with VEGFR2 and FGFR1 receptors (Figs. 10, 11 and Figs. S38–S45 in supplementary materials). The interactions of chemical **2a** with both proteins were shown as illustrative instances in (Figs. 10 and 11). From VEGFR‐2-**2a** interaction (Fig. 10), hydrogen donor interaction from the hydroxyl group of **2a** to water molecule with distance 1.83 Å, in addition, H-acceptor interactions from water molecule to (P-O) group, PTR1052 to (N=O) and LYS1053 to (N=O) with distances 1.97, 2.22 and 2.74 Å, respectively. While the interaction of **2a** with FGFR1 protein (Fig. 11) exhibits hydrogen donor interaction from the hydroxyl group of **2a** to ASP641 and hydrogen acceptor interaction from LYS514 to the same hydroxyl group of **2a** with distances 1.63 and 2.39 Å, respectively. The good interactions of **2a** with both receptors may be due to the highest positive and negative surface potentials of **2a** obtained from electrostatic potential calculations. The molecular docking result for the interactions of investigated compounds, with both proteins reveals that the most common types of interactions are hydrophobic interactions, such as π-cation, as well as H-acceptor, H-donor, halogen bond, and salt bridge interactions.

**Table 5:** Molecular docking glid G-scoring, RMSD, interaction results of the investigated compounds towards VEGFR‐2 receptor (PDB ID: 1YWN).

Compounds	Glid G-score	RMSD	Interaction	Type	Distance
Re-docked inhibitor	− 12.827	0.618	NH→GLU883	H-Bond	1.99, 2.23
			NH_2_→GLU915	H-Bond	2.08
			H_2_O→(C=N)	H-Bond	2.46
			ASP1044→(C=O)	H-Bond	2.23
Doxorubicin	− 4.988	1.667	OH→ASP1026	H-Bond	1.98
			OH→ASP1026	H-Bond	1.64
			OH→ASN1031	H-Bond	2.46
			AlA842→OH	H-Bond	1.93
2e	− 2.862	1.313	GLU883--(N^+^–O)	Salt bridge	3.41
			Cl→GLU915	Halogen bond	3.09
			CYS917→Cl	Halogen bond	2.46
2f.	− 4.466	0.915	ARG1030→(N=O)	H-Bond	1.95
			ASN921→(N=O^–^)	H-Bond	2.34
4d	− 3.014	1.451	ARG840→(N=O)	H-Bond	2.62
			ARG1030--(N=O^–^)	Salt bridge	3.40
4c	No interaction				
2d	− 2.774	0.982	GLU883--(N^+^–O)	Salt bridge	3.15
			LYS866--Ar.ring	π-cation	6.52
4b	− 3.637	1.331	LYS866--Ar. ring	π-cation	3.15
			GLU883--(N^+^–O)	Salt bridge	3.23
			ARG1030--(N=O^–^)	Salt bridge	3.91
			LYS1053--(N=O^–^)	Salt bridge	4.38
2a	− 4.132	1.454	OH→H_2_O	H-Bond	1.83
			H_2_O→(P–O)	H-Bond	1.97
			PTR1052→(N=O)	H-Bond	2.22
			LYS1053→(N=O)	H-Bond	2.74
2b	− 4.763	0.000	ASP1044→(C=N)	H-Bond	2.40
			OH→H_2_O→ASP1044	H-Bond via H_2_O molecule	1.86, 1.99
			OH→H_2_O→GLU883	H-Bond via H_2_O molecule	1.86, 2.17
			OH→H_2_O←LYS866	H-Bond via H_2_O molecule	1.86, 2.44
4a	− 4.303	1.077	OH→GLY844	H-Bond	2.09
			NH→LEU838	H-Bond	2.74
			ASP1026--(N^+^–O)	Salt bridge	5.00
2c	− 2.798	1.289	GLU883--(N^+^–O)	Salt bridge	3.41

Glide G-score: Kcal/mol, RMSD and distance: Å.

**Table 6:** Molecular docking glid G-scoring, RMSD, interaction results of the investigated compounds towards FGFR1 receptor (PDB ID: 5UR1).

Compounds	Glid G-score	RMSD	Interaction	Type	Distance
Re-docked inhibitor	− 6.290	0.792	NH→ALA564	H-Bond	2.34
			ASP641→(C–O)	H-Bond	2.17
			ALA564→(C = N)	H-Bond	2.34
			LYS514—Ar. ring	π-cation	5.51
			ASP641→Cl	halogen bond	2.92
Doxorubicin	− 5.760	0.565	OH→ASN628	H-Bond	1.97
			ASN568→(C=O)	H-Bond	2.58
			ASP641→(C=O)	H-Bond	2.63
			ASP641→(C–O)	H-Bond	2.60
			LYS514—Ar. ring	π-cation	5.19
2e	No interaction				
2f	− 3.015	0.814	ARG576→(N=O)	H-Bond	1.85
			ARG576—(N=O^–^)	Salt bridge	4.83
			LYS566—(N=O^–^)	Salt bridge	4.79
			LYS482—Ar. ring	π-cation	6.31
4d	− 2.002	0.789	LYS482—(N=O^–^)	Salt bridge	2.9
			LYS482—Ar. ring	π-cation	6.29
4c	No interaction				
2d	No interaction				
4b	No interaction				
2a	− 5.440	1.900	OH→ASP641	H-Bond	1.63
			LYS514→OH	H-Bond	2.39
2b	− 3.744	1.764	ASP641→OH	H-Bond	2.06
4a	− 1.978	1.925	NH→ASP641	H-Bond	2.1
2c	No interaction				

Glide G-score: Kcal/mol, RMSD and distance: Å.

One possible source of the difference between the theoretical and experimental data is the limitations of molecular docking computational calculations. These calculations rely on mathematical functions, algorithms, and active site sensitivity to predict the interactions between drugs and biological targets. However, these calculations do not consider the factors that affect the drug delivery method or mechanism, the experimental variations, or the biological target properties^39^.

#### Docking validation

**Figure 12:**
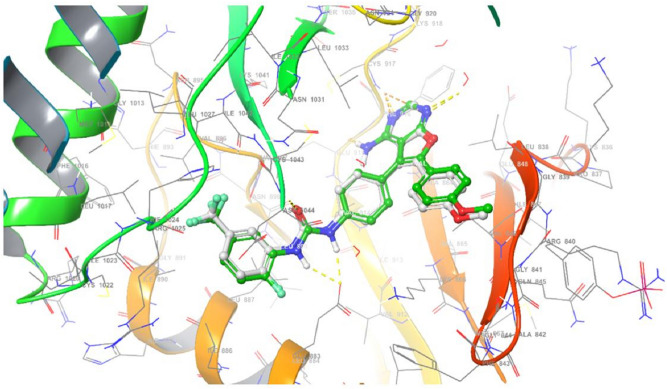
Superimposition of re-docked inhibitor (green) onto co-crystallized complex (grey) in the active site (RMSD = 0.792 Å) towards VEGFR‐2 receptor.

**Figure 13:**
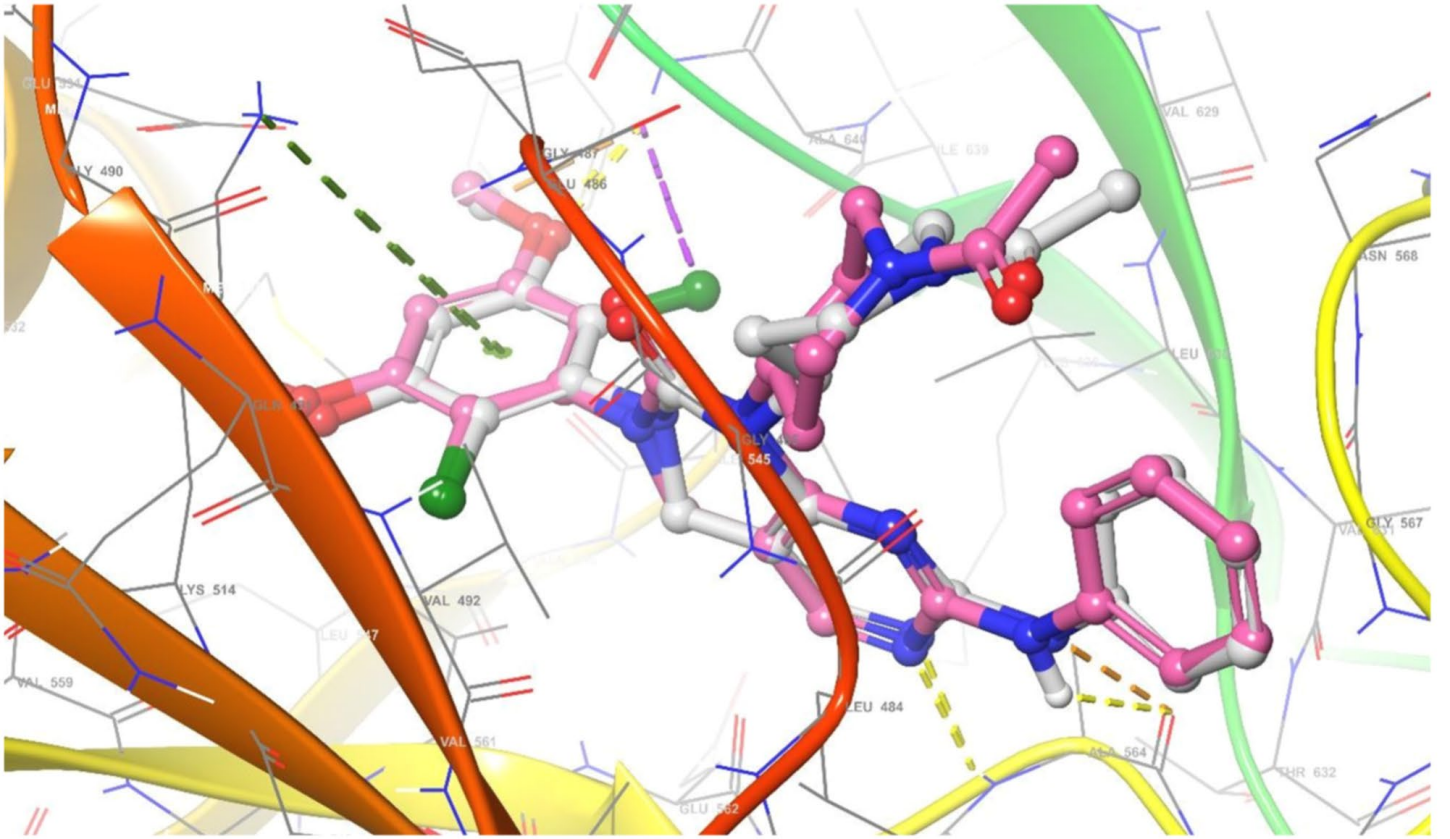
Superimposition of re-docked inhibitor (violet) onto co-crystallized complex (grey) in the active site (RMSD = 0.618 Å) towards FGFR1 receptor.

The re-docking was done to test the docking strategy and efficiency. The re-dockin approach used the same procedures as earlier. The peptide original inhibitor was precisely attached to the active site pocket of the VEGFR2 receptor by completely 5- hydrogen bonds were formed with distances (1.99, 2.23), 2.08, 2.46, and 2.23 Å. While the original inhibitor linked to the active site pocket of FGFR1 receptor through four hydrogen bonds with distances 2.34, 2.17, 2.34, and 2.92 Å as well as π-cation interaction between the aromatic ring and LYS514 with distance 5.51 Å. The re-docked original ligand was overlaid onto the native co-crystallized (Figs. 12 and 13). The re-docked inhibitor linked to VEGFR‐2 and FGFR1 receptors has molecular docking scoring (-12.827 and -6.290 kcal mol^−1^) and the RMSD of (0.618 and 0.792 Å), respectively).

### 3D- QSAR studies

To create the model, we employed the atom-based QSAR module. For all categories, we used four PLS factors. We used the cross-correlation coefficient^40^ to assess the models’ prediction abilities (Table 7). The comprehensive data for the QSAR investigation is shown in Table 8. Each set’s best model result is presented in this table.

**Table 7:** Prediction of QSAR models on drug compounds.

Compounds	VEGFR2 model			FGFR1 model		
	Experimental pIC_50_	Predicted pIC_50_	Prediction error	Experimental pIC_50_	Predicted pIC_50_	Prediction error
2a	5.022	4.902	− 0.120	4.475	4.431	− 0.044
2b	4.073	4.223	0.150	4.254	4.192	− 0.062
2c	4.340	4.437	0.097	4.528	4.459	− 0.069
2d	4.701	4.763	0.062	4.378	4.408	0.030
2e	4.242	4.193	− 0.049	4.064	4.148	0.084
2f	4.422	4.343	− 0.079	4.186	4.170	− 0.016
4a	4.135	4.143	0.008	4.327	4.342	0.015
4b	4.822	4.560	− 0.262	4.202	4.208	0.006
4c	4.290	4.487	0.197	4.000	4.027	0.027
4d	4.584	4.569	− 0.015	4.106	4.083	− 0.023
AFATINIB	4.390	4.357	− 0.033	4.390	4.370	− 0.020
AXITINIB	4.640	4.643	0.003	4.640	4.672	0.032
DACOMITINIB	5.160	5.137	− 0.023	5.160	5.168	0.008
Doxorubicin	5.281	5.290	0.009	5.173	5.177	0.004
ENTRECTINIB	5.030	5.039	0.009	5.030	4.717	− 0.313
ERDAFITINIB	4.180	4.174	− 0.006	4.180	4.197	0.017
GEFITINIB	4.280	4.256	− 0.024	4.280	4.269	− 0.011
OLMUTINIB	5.100	4.761	− 0.339	5.100	4.714	− 0.386
OSIMERTINIB	4.510	4.538	0.028	4.510	4.491	− 0.019
ROCILETINIB	4.780	4.810	0.030	4.780	4.763	− 0.018
TUCATINIB	5.090	5.098	0.008	5.090	5.105	0.015

**Table 8:** Statistical parameters of atom-based QSAR model.

Model	PLS factors	SD	R^2^	R^2^ CV	R^2^ scramble	Stability	F	P	RMSE	Q^2^	Pearson-r
VEGFR2	1	0.2626	0.5784	− 0.3838	0.6475	0.226	17.8	9.96 × 10^–04^	0.25	0.4519	0.805
	2	0.1454	0.8806	− 0.354	0.8538	− 0.252	44.3	2.89 × 10^–06^	0.24	0.5078	0.9345
	3	0.0794	0.9674	− 0.1024	0.9274	− 0.0795	108.7	1.86 × 10^–08^	0.21	0.6324	0.8979
	4	0.0585	0.9839	− 0.1924	0.969	− 0.185	152.7	6.41 × 10^–09^	0.2	0.6352	0.9086
FGFR1	1	0.2727	0.5421	0.1043	0.589	0.822	15.4	1.75 × 10^–03^	0.26	0.5515	0.9424
	2	0.1293	0.905	− 0.2142	0.8128	− 0.0954	57.2	7.35 × 10^–07^	0.24	0.6174	0.9542
	3	0.0544	0.9846	− 0.0246	0.897	0.00916	234.1	3.04 × 10^−10^	0.2	0.7334	0.9774
	4	0.0451	0.9903	− 0.097	0.9558	− 0.0759	256.5	4.98 × 10^–10^	0.2	0.7242	0.9812

**Figure 14:**
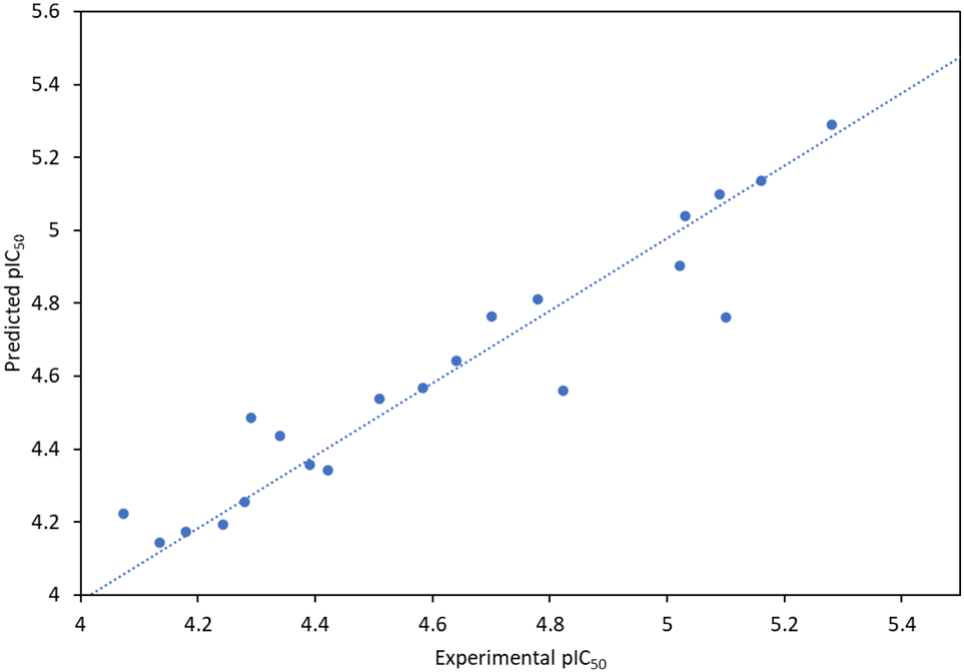
Experimental vs predicted activity of atom-based QSAR model for VEGFR2 model.

**Figure 15:**
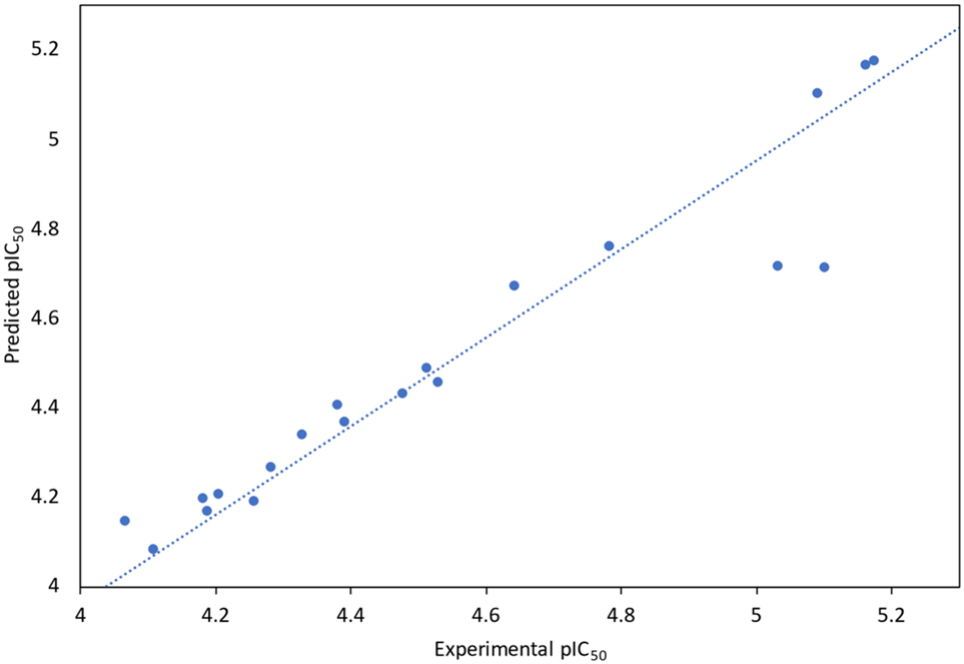
Experimental vs predicted activity of atom-based QSAR model for VEGFR2 model.

The models have R^2^, the regression coefficient, ~ 0.5–0.9, while the PLS factor 4 has R^2^ ~ 0.9, indicating the model's robustness. The cross-correlation coefficient and regression coefficient are close, indicating that the model is stable. The models have a very high F variance and low P values, indicating that they are statistically significant. The cross-correlation coefficient Q^2^ for the models is 0.635 and 0.724, respectively. This shows that the models are feasible. Figures 14 and 15 show the graphs for the models' training and test sets.

Blue cubes represent favorable locations toward inhibitory action in the models of VEGFR2 and FGFR1 receptors for substances (Fig. 16 and Figs. S42–S58, supplementary materials), whereas red cubes represent unfavorable regions. Compound **2a**, for example, demonstrates the hydrogen bond donor action of NH and both phenolic OH groups for the VEGFR2 model but is not very effective for FGFR1 model suppression. The hydrogen bond donor effect of NH is not as good as VEGFR2 inhibition, and the hydrogen bond donor effect of one of the hydroxyl groups for the FGFR1 model is reduced while the impact of the other OH group is vanished.

**Figure 16:**
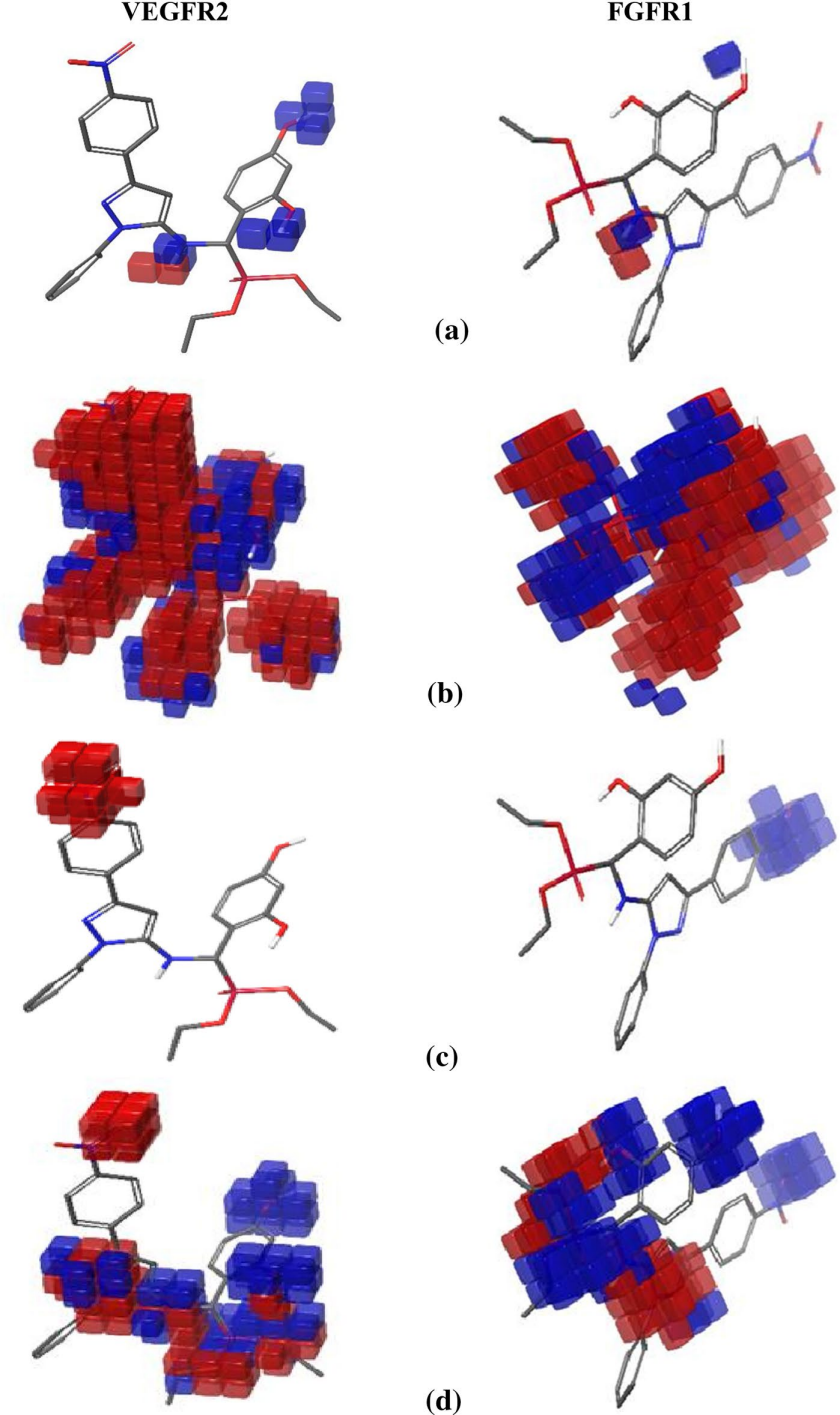
QSAR models of compound 2a with VEGFR2 and FGFR1 receptors inhibition for (**a**) hydrogen bond donor effect, (**b**) hydrophobic effect, (**c**) positive ionic effect, and (**d**) electron withdrawing effect.

The compound’s hydrophobic impact cannot be utilized to differentiate between VEGFR2 and FGFR1 inhibition. The majority of hydrophobic areas behave similarly to both systems.

The electron-drawing effect is likewise insufficient for distinguishing the inhibitory characteristics of VEGFR2 and FGFR1. Also, the electron-withdrawing effect of N(CH_3_)_2_ substituent is equally good for inhibiting both models. But the electron-withdrawing effect of (NO_2_) substituents shows good and poor inhibiting. Finally, the (Cl) substituent doesn’t exhibit any effect.

The positive ionic effect of (NO_2_) ion made compounds are not favorable for VEGFR2 inhibition. On the other hand, the positive ionic effect of (NO_2_) toward FGFR1 inhibition is good inhibitor except for the compound 2f and 4d. As a result, the compound’s positive ionic action on both inhibitory systems is very instructive in distinguishing between VEGFR2 and FGFR1 inhibition.

## Conclusion

In conclusion, this study is based on synthesized and evaluated new series of α-aminophosphonates **2a-f** and **4a-d** derivatives containing pyrazole moiety by reaction of various aromatic aldehydes with diethyl and/or diphenyl phosphites in presence of LiClO_4_ as a catalyst under convenient and efficient conditions via the Kabachnik-Fields reaction. The structure of the synthesized compounds was confirmed by elemental analysis, FT-IR, ^1^H NMR, ^13^C NMR, and MS spectral data. All of the synthesized compounds were evaluated for their in vitro antitumor activities, against colorectal carcinoma Colon cancer (HCT-116) and Epdermoid carcinoma (HEP2) and also Human lung fibroblast normal cell line (WI38). From the data, we could conclude that all these novel α-aminophosphonates could inhibit tumor cell lines (HCT-116 and HEP2) below 10 µM by the MTT assay. Moreover, Compounds **2a, 4b and 4d** exhibited more potent inhibitory activity for Epdermoid Carcinoma (HEP2 with IC50 values (0.9 ± 12.5, 1.40 ± 17.76, and 1.8 ± 21.28 μM), respectively, compared with doxorubicin (0.6 ± 8.54 μM), while the rest compounds have moderate to weak activity against the same cell line. For colon carcinoma cells (HCT-116) Compounds **2a, 2d and 4b** gave the strongest activity among all compounds with IC50 values (0.7 ± 9.51, 1.6 ± 19.90, and 1.1 ± 15.06 μM), respectively, compared with doxorubicin (0.3 ± 5.32 μM). Finally, all the studied phosphonate compounds have good to moderate cytotoxicity activity on the normal cell WI-38. The geometry optimization results using DFT exhibits that the stability of **4d** is higher than that of other compounds as well as **2f** has the highest electron acceptance while dipole moment of **4a** is greater than other compounds’ values which may increase hydrophilic nature of **4a**. From molecular docking interaction, the results reveals that compounds **2a** shows good interactions with VEGFR‐2 and FGFR1 proteins respectively, this behavior may be due to the highest positive and negative surface potentials of **2a** obtained from electrostatic potential calculations. On the other hand, compound **4c** doesn’t exhibit any interactions with the VEGFR‐2 protein. Also compounds **4c, 2e, 2d, 4b, and 2c** don’t show any interactions with the FGFR1 protein. From these data, one can notice that compound **4c** has no interaction with FGFR1 protein and agree with experimental data of in vitro cytotoxicity against WI38 with IC50 values (> 100 μM).

## Experimental

### Materials and instrumentation

All data of chemicals and instruments are available in the supplementary file (Sect. S1). In accordance with literature procedures, the compounds aryl substituted pyrazolamines^41^ were synthesized.

### Chemistry

#### Synthesis of α-aminophosphonate compounds 2a-e and 4a-d

To a stirred solution of 3-(4-nitrophenyl)-1-phenyl-1H-pyrazol-5-amine **1**/or 3-(4-nitrophenyl)-1-phenyl-1H-pyrazol-5-amine **2** (0.01 mol) and appropriate aldehyde derivatives (0.0012 mol) in dry dichloromethane CH_2_Cl_2_ (5 ml), triphenyl phosphite and/or triethyl phosphite (0.001 mol) and anhydrous lithium perchlorate LiClO_4_ (100 mol %) were added. The reaction mixture was stirred at room temperature (48 h) until the completion of the reaction as indicated by TLC. Then CH_2_Cl_2_ was evaporated and the α-amino phosphonates were precipitated using methanol. The precipitate was filtered off affording new α-amino phosphonates in good yield.

### Diethyl{(3-(4-nitrophenyl)-1-phenyl-1H-pyrazol-5-ylamino)(2,3-dihydroxyphenyl)}methyphosphonate (2a)

Isolated yield = 85%, Melting point: 277–279 °C, IR: ν/cm^–1^: 3248 (NH), 3029 (CH-Arom.), 2925 (CH-Aliph), 1254 (P=O), 1086 (P–O–C), 761 (P–CH). ^1^H NMR (400 MHz, DMSO-*d6*) δ 1.32 (m, 6H, 2CH_3_), 3.65 (m, 4H, 2CH_2_), 5.07 (s, 1H, CH-P), 6.94 (s, 1H, CH-Pyrazole), 7.14–8.00 (m, 7H, Ar–H), 5.70 (br, 1H, NH, exchangeable with D_2_O), 9.41, 9.31(2H, OH, exchangeable with D_2_O). ^13^C NMR (101 MHz, DMSO-*d6*) δ 12.23 (2CH_3_), 52.47 (2CH_2_), 54.04 (CH-P), 83.24 (CH-Pyrazole), 152.28 (C=N pyrazole), 148.63 (C–NH), 148.47 (C–NO_2_), 161.43(C–OH), 122.16–148.34 (Ar–C). MS (EI, 70 eV): m/z = 538.73 [M]^+^. Anal. Calcd. For C_26_H_27_N_4_O_7_P (538.16) C, 57.99; H, 5.05; N, 10.40. Found; C, 57.91; H, 4.98; N, 10.34.

### Diethyl{(3-(4-nitrophenyl)-1-phenyl-1H-pyrazol-5-ylamino)(3-hydroxyphenyl)}methylphosphonate (2b)

Isolated yield = 77%, Melting point: 252–254 °C. IR: ν/cm^–1^: 3413 (NH), 3025 (CH–Arom.), 2917 (CH–Aliph.), 1265 (P=O); 1006 (P–O–C), 700 (P–CH). ^1^H NMR (400 MHz, DMSO-*d6*) δ 1.23 (m, 6H, 2CH_3_), 4.25 (m, 4H, 2CH_2_), 5.29 (s, 1H, CH-P), 6.55 (s, 1H, CH-Pyrazole), 7.14–8.72 (m, 7H, Ar–H), 5.71 (br, 1H, NH, exchangeable with D_2_O), 9.06 (s, 1H, OH, exchangeable with D_2_O). ^13^C NMR (101 MHz, DMSO-*d6*) δ 12.23 (2CH_3_), 59.59 (2CH_2_), 66.81 (CH-P), 81.93 (CH-Pyrazole), 167.94 (C=N pyrazole), 146.24 (C-NH), 145.54 (C–NO_2_), 117.44–144.20 (Ar–C). MS (EI, 70 eV): m/z = 522.66 [M]^+^. Anal. Calcd. For C_26_H_27_N_4_O_6_P (522.17) C, 59.77; H, 5.21; N, 10.72. Found; C, 59.69; H, 5.16; N, 10.65.

### Diethyl{(3-(4-nitrophenyl)-1-phenyl-1H-pyrazol-5-ylamino)(3-chlorophenyl)}methylphosphonate (2c)

Isolated yield = 84%, Melting point: 150–152 °C. IR: ν/cm^–1^: 3435 (NH), 3029 (CH–Arom.), 2982 (CH–Aliph.), 1193 (P=O), 1104 (P–O–C), 758 (P–CH). ^1^H NMR (400 MHz, DMSO-*d6*) δ 1.26 (m, 6H, 2CH_3_), 3.62 (m, 4H, 2CH_2_), 5.35 (s, 1H, CH-P), 6.23 (s, 1H, CH-Pyrazole), 6.72–8.52 (m, 7H, Ar–H), 5.61 (br, 1H, NH,exchangeable with D_2_O). ^13^C NMR (101 MHz, DMSO-*d6*) δ 22.06 (2CH_3_), 64.01 (2CH_2_), 66.82 (CH-P), 85.49 (CH-Pyrazole), 146.42 (C=N pyrazole), 143.77 (C–NH), 139.33 (C-NO_2_), 119.72–137.88 (Ar–C). MS (EI, 70 eV): m/z = 540.13 [M] ^+^. Anal. Calcd. For C_26_H_26_ClN_4_O_5_P (540.12) C, 57.73; H, 4.84; N, 10.36. Found; C, 57.67; H, 4.79; N, 10.29.

### Diethyl{(3-(4-nitrophenyl)-1-phenyl-1H-pyrazol-5-ylamino)(4-nitrophenyl)}methylphosphonate (2d)

Isolated yield = 75%, Melting point: 234–235 °C. IR: ν/cm^-1^: 3430 (NH), 3068 (CH–Arom.), 2853 (CH–Aliph.), 1230 (P=O); 1083 (P–O–C), 764 (P–CH). ^1^H NMR (400 MHz, DMSO-*d6*) δ 1.39 (m, 6H, 2CH_3_), 4.32 (m, 4H, 2CH_2_), 5.52 (s, 1H, CH-P), 6.81 (s, 1H, CH-Pyrazole), 7.13–8.35 (m, 7H, Ar–H), 5.85 (br, 1H, NH,exchangeable with D_2_O). ^13^C NMR (101 MHz, DMSO-*d6*) δ 15.21 (2CH_3_), 58.29 (2CH_2_), 59.05 (CH–P), 89.37 (CH–Pyrazole), 148.97 (C = N pyrazole), 144.32 (C–NH), 142.92 (C–NO_2_), 116.39–135.83 (Ar–C). MS (EI, 70 eV): m/z = 549.12 [M^+^-2]. Anal. Calcd. For C_26_H_26_N_5_O_7_P (551.16) C, 56.62; H, 4.75; N, 20.31. Found; C, 56.54; H, 4.69; N, 20.24.

### Diethyl{(3-(4-nitrophenyl)-1-phenyl-1H-pyrazol-5-ylamino)(4-chlorophenyl)}methylphosphonate (2e)

Isolated yield = 77%, Melting point: 298–300 °C. IR: ν/cm^–1^: 3447 (NH), 3162 (CH–Arom.), 2922 (CH–Aliph.), 1239 (P=O), 1069 (P–O–C), 765 (P–CH). ^1^H NMR (400 MHz, DMSO-*d6*) δ 1.31 (m, 6H, 2CH_3_), 4.39 (m, 4H, 2CH_2_), 5.49 (s, 1H, CH-P), 6.42 (s, 1H, CH-Pyrazole), 6.72–8.95 (m, 7H, Ar–H), 5.92 (br, 1H, NH, exchangeable with D_2_O). ^13^C NMR (101 MHz, DMSO-*d6*) δ 16.97 (2CH_3_), 41.90 (2CH_2_), 66.82 (CH-P), 82.89 (CH-Pyrazole), 151.35 (C=N pyrazole), 150.40 (C–NH), 146.87 (C–NO_2_), 116.97–139.73 (Ar–C). MS (EI, 70 eV): m/z = 540.80 [M] ^+^. Anal. Calcd. For C_26_H_26_ClN_4_O_5_P (540.13) C, 57.73; H, 4.84; N, 10.36. Found; C, 57.65; H, 4.77; N, 10.29.

### Diethyl{(1-(2,4-dinitrophenyl)-3-phenyl-1H-pyrazol-5-ylamino)(4-N,Ndimethylaminophenyl)} methyl phosphonate (2f)

Isolated yield = 82%, Melting point: 244–246 °C. IR: ν/cm^–1^: 3422 (NH), 3065 (CH–Arom.), 2919 (CH–Aliph.), 1267 (P=O); 1011 (P–O–C), 701(P–CH). ^1^H NMR (400 MHz, DMSO-*d6*) δ 2.61 (s, 6H, 2CH_3_), 1.41 (m, 6H, 2CH_3_), 3.93 (m, 4H, 2CH_2_), 5.31 (s, 1H, CH-P), 6.94 (s, 1H, CH-Pyrazole), 7.14–9.41 (m, 7H, Ar–H), 5.99 (br, 1H, NH, exchangeable with D_2_O). ^13^C NMR (101 MHz, DMSO-*d6*) δ 20.61(2CH_3_), 12.63 (2CH_3_), 55.16 (2CH_2_), 64.82 (CH-P), 83.86 (CH-Pyrazole), 161.43 (C = N pyrazole), 152.28 (C–NH), 148.62, 148.47 (2 C–NO_2_), 119.26–148.34 (Ar–C). MS (EI, 70 eV): m/z = 594.67 [M] ^+^. Anal. Calcd. For C_28_H_31_N_6_O_7_P (594.20) C, 56.56; H, 5.26; N, 14.13. Found; C, 56.49; H, 5.21; N, 14.07.

### Diphenyl{(3-(4-nitrophenyl)-1-phenyl-1H-pyrazol-5-ylamino)(3-hydroxyphenyl)}methylphosphonate (4a)

Isolated yield = 81%, Melting point: 266–268 °C. IR: ν/cm^–1^: 3432 (NH), 3032 (CH–Arom.), 1234 (P=O), 1015 (P–O–C), 701 (P–CH). ^1^H NMR (400 MHz, DMSO-*d6*) δ 5.34 (s, 1H, CH-P), 6.84 (s, 1H, CH-Pyrazole), 6.32–8.64 (m, 7H, Ar–H), 5.74 (br, 1H, NH,exchangeable with D_2_O), 6.34 (s, 1H, OH, exchangeable with D_2_O). ^13^C NMR (101 MHz, DMSO-*d6*) δ 68.35 (CH–P), 89.37 (CH-Pyrazole), 159.05 (C=N) pyrazole, 158.29 (C–NH), 148.97 (C–NO_2_), 116.39–135.83 (Ar–C). MS (EI, 70 eV): m/z = 617.04 [M^+^-1]. found,.Anal. Calcd. For C_34_H_27_N_4_O_6_P (618.17) C, 66.02; H, 4.40; N, 9.06. Found; C, 65.96; H, 4.32; N, 8.99.

### Diphenyl{(3-(4-nitrophenyl)-1-phenyl-1H-pyrazol-5-ylamino)(4-nitrophenyl)}methylphosphonate (4b)

Isolated yield = 83%, Melting point: 167–168 °C. IR: ν/cm^–1^: 3432 (NH), 3065 (CH–Arom.), 2925 (CH–Aliph.), 1235 (P=O), 1025 (P–O–C), 765 (P–CH). ^1^H NMR (400 MHz, DMSO-*d6*) δ 5.49 (s, 1H, CH–P), 6.61 (s, 1H, CH–Pyrazole), 6.75–8.45 (m, 7H, Ar–H), 5.94 (br, 1H, NH, exchangeable with D_2_O). ^13^C NMR (101 MHz, DMSO-*d6*) δ 64.01 (CH–P), 83.77 (CH–Pyrazole), 146.42 (C=N pyrazole), 143.77 (C–NH), 139.33 (C–NO_2_), 119.72–137.88 (Ar–C). MS (EI, 70 eV): m/z = 647.25 [M] ^+^. Anal. Calcd. For C_34_H_26_N_5_O_7_P (647.16) C, 63.06; H, 4.05; N, 10.81. Found; C, 62.98; H, 3.97; N, 10.74.

### Diphenyl{(1-(2,4-dinitrophenyl)-3-phenyl-1H-pyrazol-5-ylamino)(3-nitrophenyl)}methyl phosphonate (4c)

Isolated yield = 79%, Melting point: 198–200 °C. IR: ν/cm^–1^: 3434 (NH), 3063 (CH–Arom.), 2924 (CH–Aliph.), 1237 (P=O), 1064 (P–O–C), 764 (P–CH). ^1^H NMR (400 MHz, DMSO-*d6*) δ 5.29 (s, 1H, CH–P), 7.12 (s, 1H, CH–Pyrazole), 7.31–8.73 (m, 7H, Ar–H), 5.75 (br, 1H, NH, exchangeable with D_2_O), 6.31 (s, 1H, OH, exchangeable with D_2_O). ^13^C NMR (101 MHz, DMSO-*d6*) δ 58.28 (CH-P), 89.39 (CH–Pyrazole), 158.28 (C=N pyrazole), 148.96 (C–NH), 147.26 (C–NO_2_), 160.41(C–OH), 114.75–140.75 (Ar–C). MS (EI, 70 eV): m/z = 663.91 [M] ^+^. Anal. Calcd. For C_34_H_26_N_5_O_8_P (663.15) C, 61.54; H, 3.95; N, 10.55. Found; C, 61.49; H, 3.89; N, 10.48.

### Diphenyl{(1-(2,4-dinitrophenyl)-3-phenyl-1H-pyrazol-5-ylamino)(4-N,N-dimethylaminophenyl)} methyl phosphonate (4d)

Isolated yield = 80%, Melting point: 212–214 °C. IR: ν/cm^–1^: 3464 (NH), 3025 (CH–Arom.), 2921 (CH–Aliph.), 1252 (P=O), 1080 (P–O–C), 732 (P–CH). ^1^H NMR (400 MHz, DMSO-*d6*) δ 2.51 (s, 6H, 2CH_3_), 5.76 (s, 1H, CH–P), 6.87 (s, 1H, CH–Pyrazole), 7.38–9.26 (m, 7H, Ar–H), 5.92 (br, 1H, NH, exchangeable with D_2_O). ^13^C NMR (101 MHz, DMSO-*d6*) δ 20.61 (2CH_3_), 65.16 (CH–P), 88.98 (CH–Pyrazole), 158.36 (C=N pyrazole), 156.46 (C–NH), 148.27, 146.46 (2 C–NO_2_), 144.35–143.74 (Ar–C). MS (EI, 70 eV): m/z = 690.81 [M] ^+^. Anal. Calcd. For C_36_H_31_N_6_O_7_P (690.20) C, 62.61; H, 4.52; N, 12.17. Found; C, 62.55; H, 4.46; N, 12.10.

### Antitumor screening

The in vitro antitumor activity of the compounds under study in this research was investigated cell growth using the MTT assay^30, 31^. The human tumor cell lines that used to evaluate the activity are Human lung fibroblast (WI38), Colorectal carcinoma Colon cancer (HCT-116) and Epdermoid Carcinoma (HEP2). The MTT assay is a standard colorimetric assay that is used to measure cell growth. It is used to determine cytotoxicity of potential medicinal agents and other toxic materials. Detailed procedures for the MTT assay method is presented in the supporting information (Sect. S2).

### Molecular modelling

The Jaguar 11.2 software^40, 41^ in Schrödinger’s suite^42^ was used to optimise the geometry of investigated compounds. The density functional theory (DFT) was utilised to simulate chemical processes and predict material characteristics using the hybrid density functional technique B3LYP-d3^43^ combination with a 6-31G** basis set for examined substances.

### Molecular docking

A molecular modeling technique called molecular docking is often used to examine how receptors and investigated compounds interact in detail^44^.

### Protein preparation

Vascular endothelial growth factor receptor 2 (VEGFR‐2) was found to be crucial for cell survival, which regulates endothelial differentiation in human colorectal carcinoma (HCT‐116)^45, 46^. Furthermore, Fibroblast growth factor receptors (FGFR1) amplification is commonly observed in 9–20% of squamous non-small cell lung cancer^47, 48^. Therefore, the three-dimensional complex structures of HCT-116 (PDB ID: 1YWN) and W-I38 (PDB ID: 5UR1) were retrieved from the Protein Data Bank^49, 50^ which they are related to the biological targets VEGFR‐2 and FGFR1, respectively. The protein structures were created with the Schrödinger suite’s protein preparation wizard^51, 52^ which eliminated water molecules (> 5A radius) and small molecules from the structural section, built disulfide connections, and added hydrogens to the PDB structures. Constrained minimization with default parameters was performed on the structure using the optimized potentials for liquid simulations (OPLS-2005) force field. The structures created were used to generate receptor grids for docking.

### Preparation of ligands

The standard drug Doxorubicin and the optimized target compounds using a basis set 6-31G** were intended to be used for docking and were organized using the default methodology of the Ligprep software^53^ in Schrödinger’s suite. The glide program^52^ in Schrödinger’s suite was used for docking studies. The glide dock XP procedure was used to dock all investigated compounds to the target protein. The G-score value is a letter grade that indicates how well a compound binds to a receptor. The RMSD may also be utilised to compare a binding conformation to a reference binding configuration. The G-score and RMSD values can be identified as potential inhibitors^54^.

### Docking validation

The docking procedure was confirmed by removing and redocking 1-{4-[4-Amino-6-(4-methoxyphenyl)furo[2,3-d]pyrimidin-5-yl]phenyl}-3-[2-fluoro-5-(trifluoromethyl)phenyl]urea inhibitor from the VEGFR‐2 receptor (PDB ID: 1YWN) and 3-(2,6-dichloro-3,5-dimethoxyphenyl)-1-{1-[4-(dimethylamino)but-2-enoyl]piperidin-4-yl}-7-(phenylamino)-3,4-dihydropyrimido[4,5-d]pyrimidin-2(1H)-one (PDB ID: 5UR1) inhibitor from the FGFR1 receptor into the active sites using Schrödinger’s suite software using the same approach including function parameters were unaltered in the process. This was done to ensure that the inhibitor binds to the active site pocket accurately and with less variance than the true co-crystallized complex.

### Atom-based QSAR model

Three-dimensional QSAR analysis is a high-dimensional QSAR approach in which the descriptors are three-dimensional^55^. Schrodinger software’s phase module was used to create atom-based QSAR models for the two sets. The dataset should be extremely well aligned with each other^56^ to derive a statistically valid 3D QSAR model.

The training set for model creation was chosen at random, with 70% of the molecules in the training set and 30% in the test set for developing models for VEGFR2 and FGFR1 receptors. For regression analysis, the partial least squares regression approach with a PLS factor of four was utilized^57^ The PLS factor establishes the relationship between the dependent and independent variables. The same collection of training and test set molecules is used for QSAR model creation of VEGFR2 and FGFR1 receptors inhibition. The models’ dependability and utility were validated by utilizing an external collection of 10 active medicinal compounds retrieved from the ChEMBL drug. (https://www.ebi.ac.uk/chembl/).

### Supplementary Information

Supplementary Information.

## Data Availability

The cell lines were provided from the American Type Culture Collection (ATCC) via VACSERA, Cairo, Egypt. The datasets generated and/or analyzed during the current study are available in: Macromolecule protein structure, can be deposited in the worldwide protein data bank repository, (https://www.rcsb.org/structure/1YWN and https://www.rcsb.org/structure/5UR1). The Jaguar 11.2 software in Schrödinger’s suite was used to optimize the geometry of investigated compounds. The density functional theory (DFT) was utilized to simulate chemical processes and predict material characteristics using the hybrid density functional technique B3LYP-d3 combination with a 6-31G** basis set for examined substances**.** The optimized target compounds using a basis set 6-31G** were intended to be used for docking and were organized using the default methodology of the Ligprep software in Schrödinger’s suite. The glide program in Schrödinger’s suite was used for docking studies.
