# Supplementary material for: A new class of anticancer activity with computational studies for a novel bioactive aminophosphonates based on pyrazole moiety
Source: Sci Rep. 2023 Sep 6;13:14680. doi: 10.1038/s41598-023-40265-8 (PMC10482913; doi:10.1038/s41598-023-40265-8)
Supplement: Supplementary file 1 — Supplementary Information. [file 41598_2023_40265_MOESM1_ESM.pdf]

Supplementry Material

A new class of anticancer activity with computational studies and molecular docking simulation for a novel bioactive aminophosphonates based on pyrazole moiety

Mohamed H. Baren[a], Seham A.Ibrahim[a*], Munirah M. Al-Rooqi[b], Saleh A. Ahmed [b], Mohammed M. El-Gamil[c], Hend A. Hekal[a]**

[a] Chemistry Department, Faculty of Science, Tanta University, 31527, Egypt
[b] Department of Chemistry, Faculty of Applied Sciences, Umm Al-Qura University, Makkah 21955, Saudi Arabia
[c] Department of Toxic and Narcotic Drug, Forensic Medicine, Mansoura Laboratory, Medico legal Organization, Ministry of Justice, Mansoura, Egypt

Materials and instrumentation (Section S1)

Unless otherwise stated, all reagents of analytical grade were purchased from Sigma-Aldrich and were used without further purification. All melting points were measured without corrections on a Gallenkamp melting point apparatus. The Fourier transform infrared spectroscopy (FTIR) spectra were recorded on a Perkin-Elmer FTIR 1430 spectrophotometer using KBr disk technique. The ^{1}H nuclear magnetic resonance (NMR) spectra were recorded on a Bruker AC spectrometer (400 MHz) at 25°C in DMSO-d6 with TMS as an internal standard, and chemical shifts are reported in parts per million as d values; ^{13}C NMR was set at 101 MHz. Mass spectra were measured on a Finnigan MAT 8222 EX electron impact mass spectrometer (EIMS) at 70 eV. Elemental analyses for C, H, and N were also carried out at the Regional Center for Mycology and Biotechnology, Azhar University, and the values were found to be within 0.4% of the theoretical ones unless otherwise indicated. Reaction progress was monitored by thin-layer chromatography using benzene/ethylacetate (3/1 by volume) as eluent.

Cytotoxicity Assay (Section S2)

The cell lines Human lung fibroblast (WI38), Colorectal carcinoma Colon cancer (HCT-116) and Epdermoid Carcinoma (HEP2) were used to determine the inhibitory effects of compounds on cell growth using the MTT assay. This colorimetric assay is based on the conversion of the yellow tetrazolium bromide (MTT) to a purple formazan derivative by mitochondrial succinate dehydrogenase in viable cells. Cell lines were cultured in RPMI-1640 medium with 10% fetal bovine serum. Antibiotics added were 100 units/ml penicillin and 100µg/ml streptomycin at 37 C in a 5% Co2 incubator. The cell lines were seedes in a 96-well plate at a density of 1.0x104 cells/well. At 37 C for 48 h under 5% CO2. After incubation the cells were treated with different concentration of compounds and incubated for 24 h. After 24 h of drug treatment, 20 µl of MTT solution at

5mg/ml was added and incubated for 4 h. Dimethyl sulfoxide (DMSO) in volume of 100 µl is added into each well to dissolve the purple formazan formed. The colorimetric assay is measured and recorded at absorbance of 570 nm using a plate reader (EXL 800, USA). The relative cell viability in percentage was calculated as (A570 of treated samples/A570 of untreated sample) X 100.

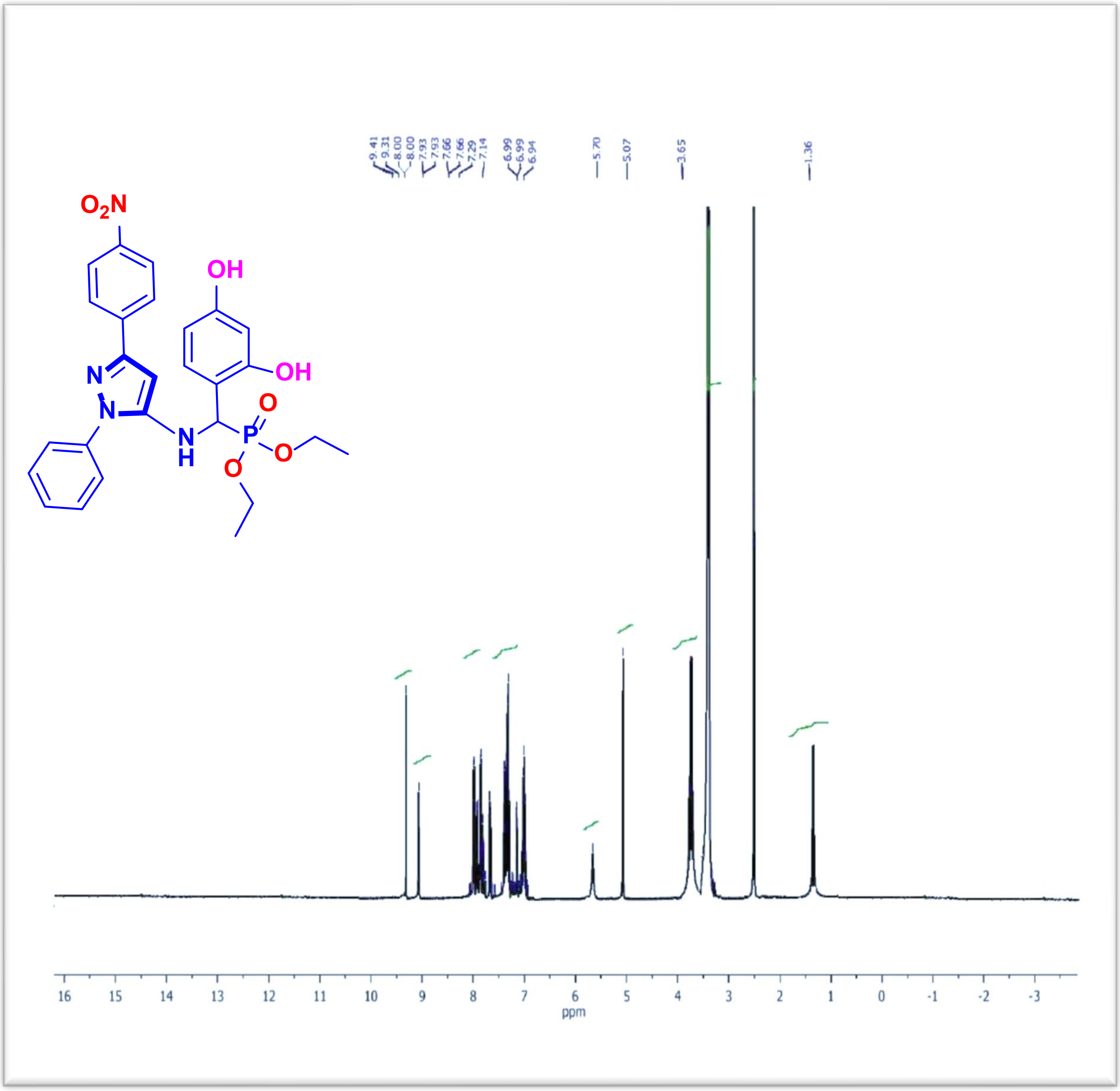

Fig. S1. ^{1}H-NMR spectrum of α-aminophosphonate **2a**.

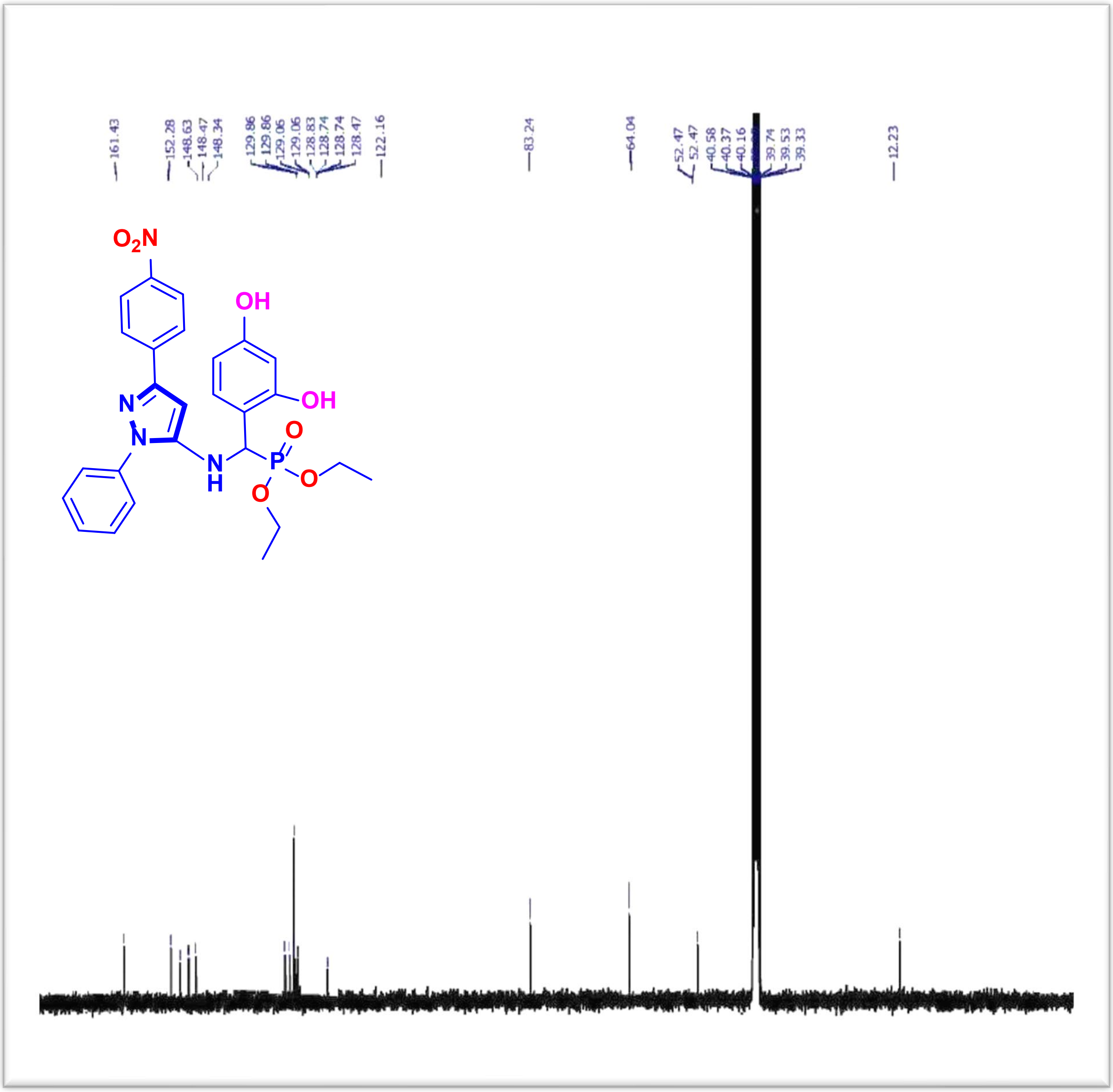

Fig. S2. ^{13}C-NMR spectrum of α-aminophosphonate **2a**.

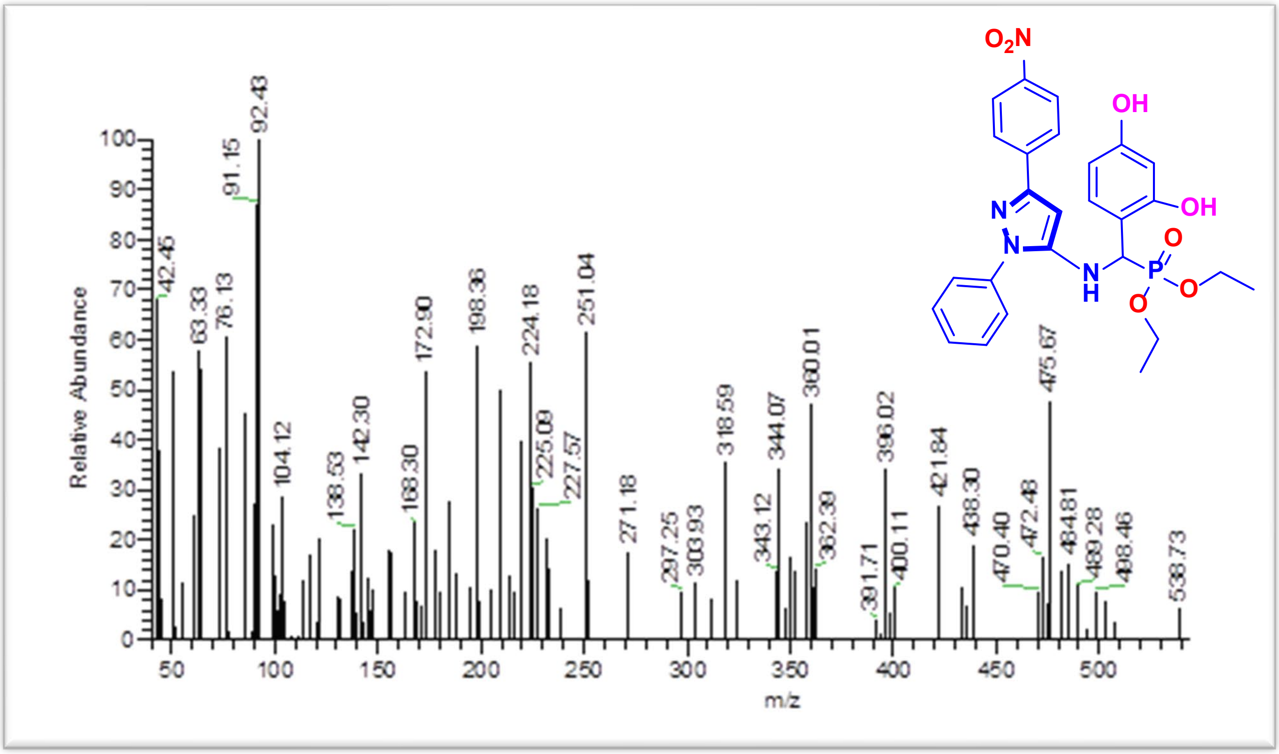

Fig. S3. Mass spectrum of α-aminophosphonate **2a**

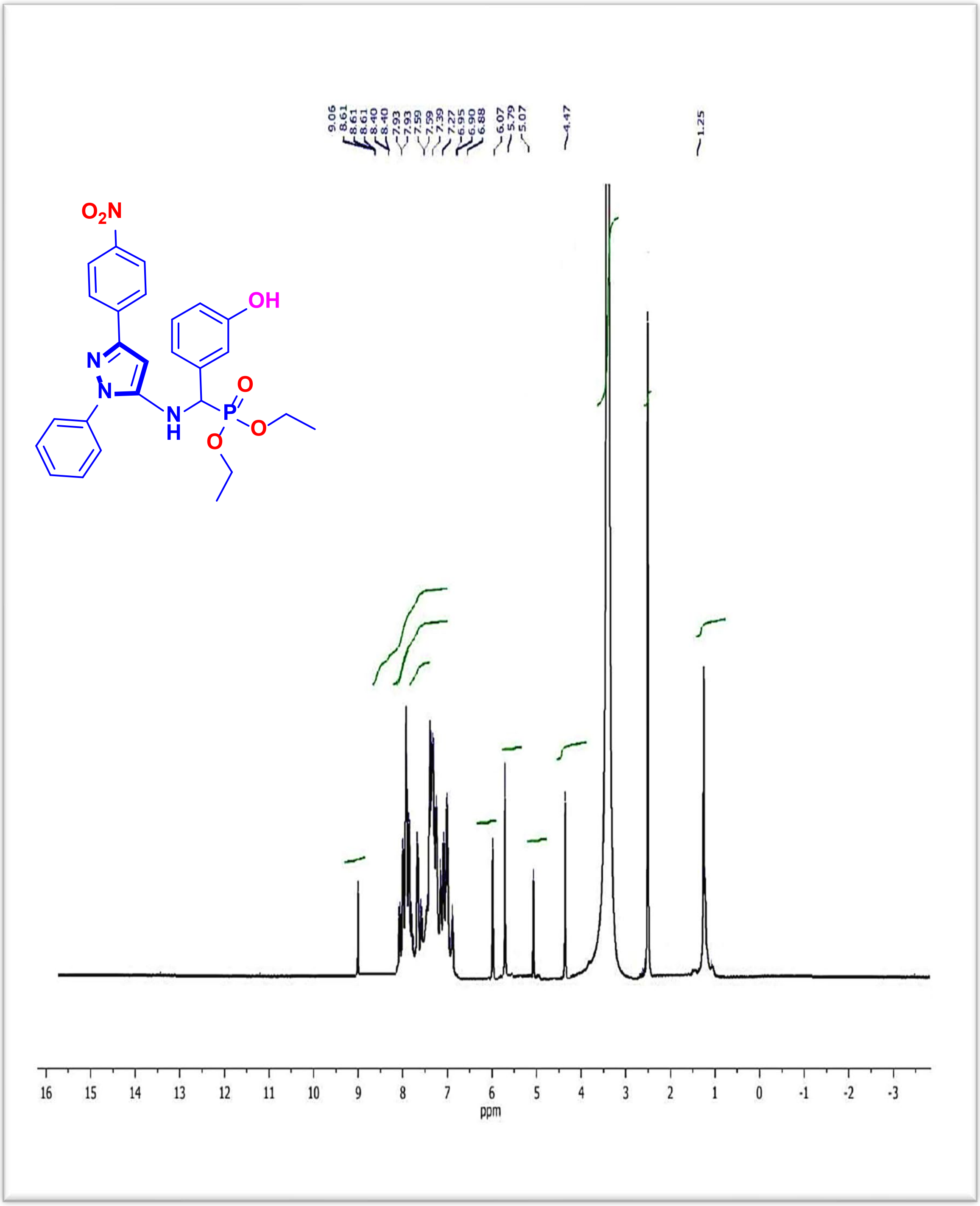

Fig. S4. ^{1}H-NMR spectrum of α-aminophosphonate **2b**.

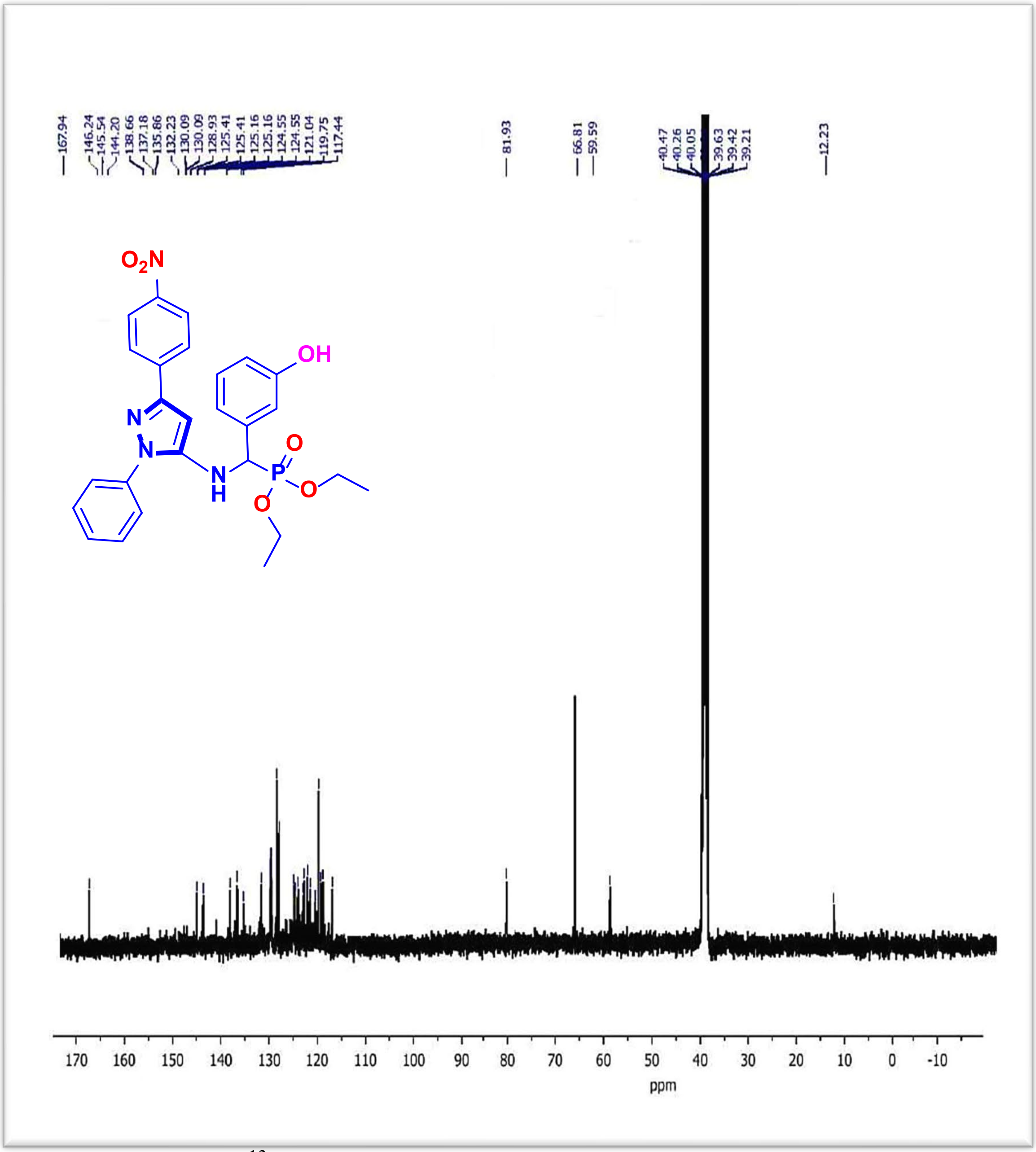

Fig. S5. ^{13}C-NMR spectrum of α-aminophosphonate **2b**.

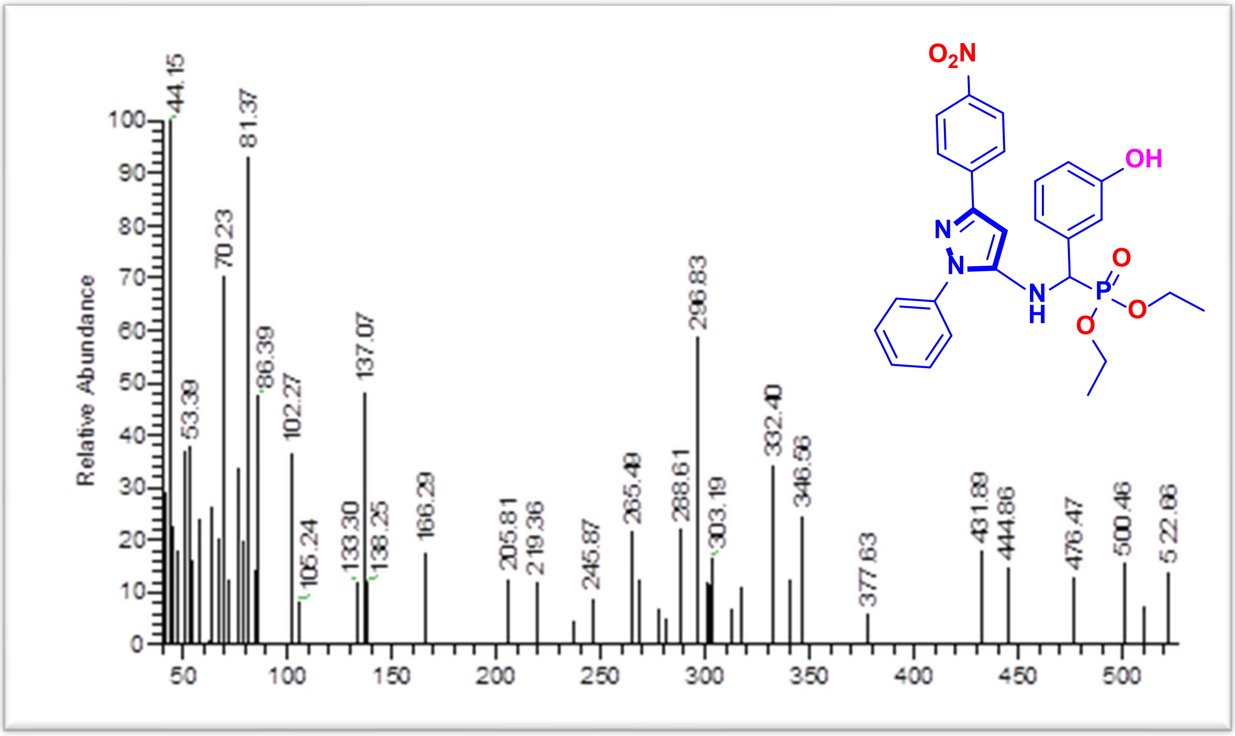

Fig. S6. Mass spectrum of α-aminophosphonate **2b.**

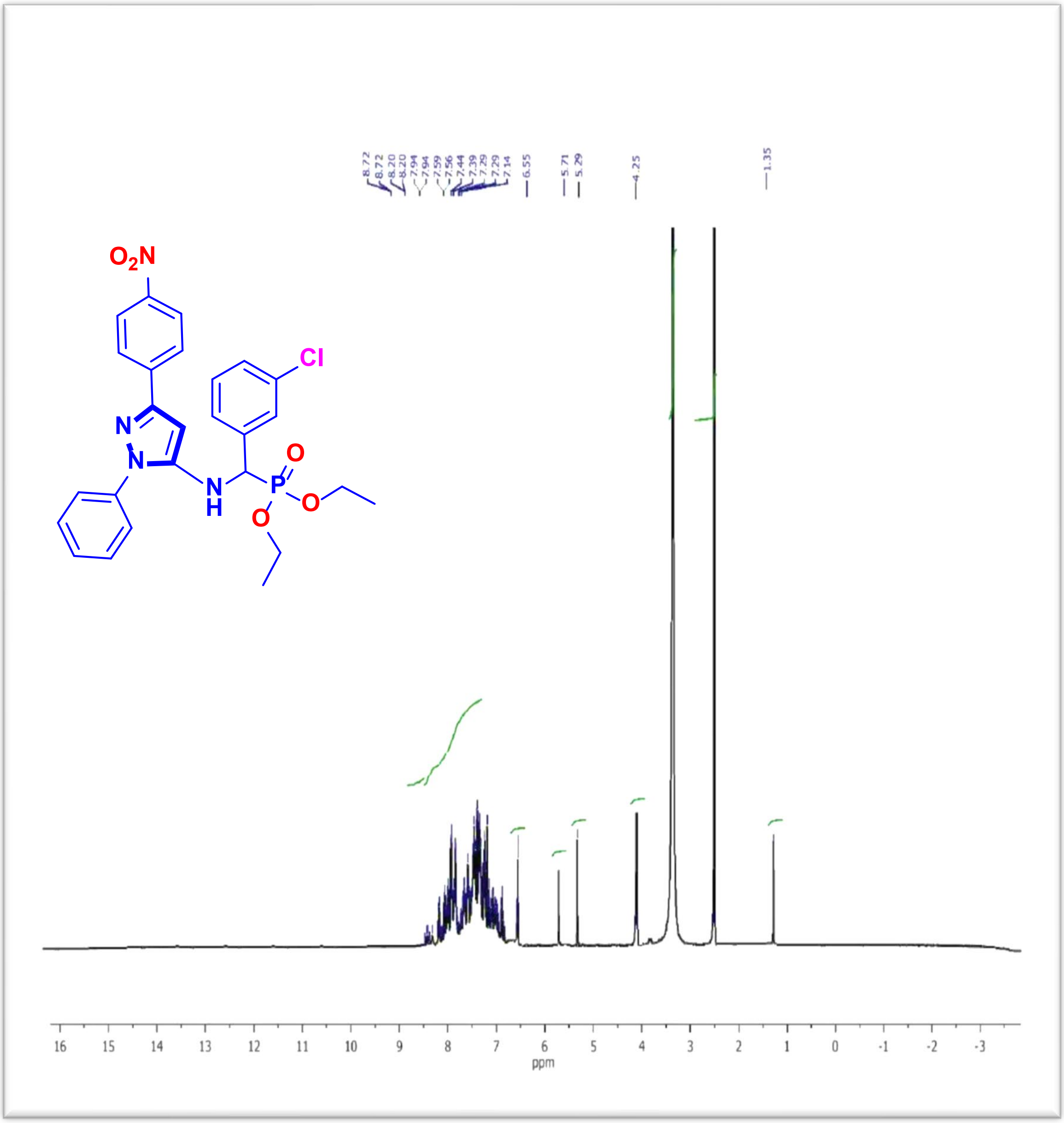

Fig. S7. ^{1}H-NMR spectrum of α-aminophosphonate **2c**.

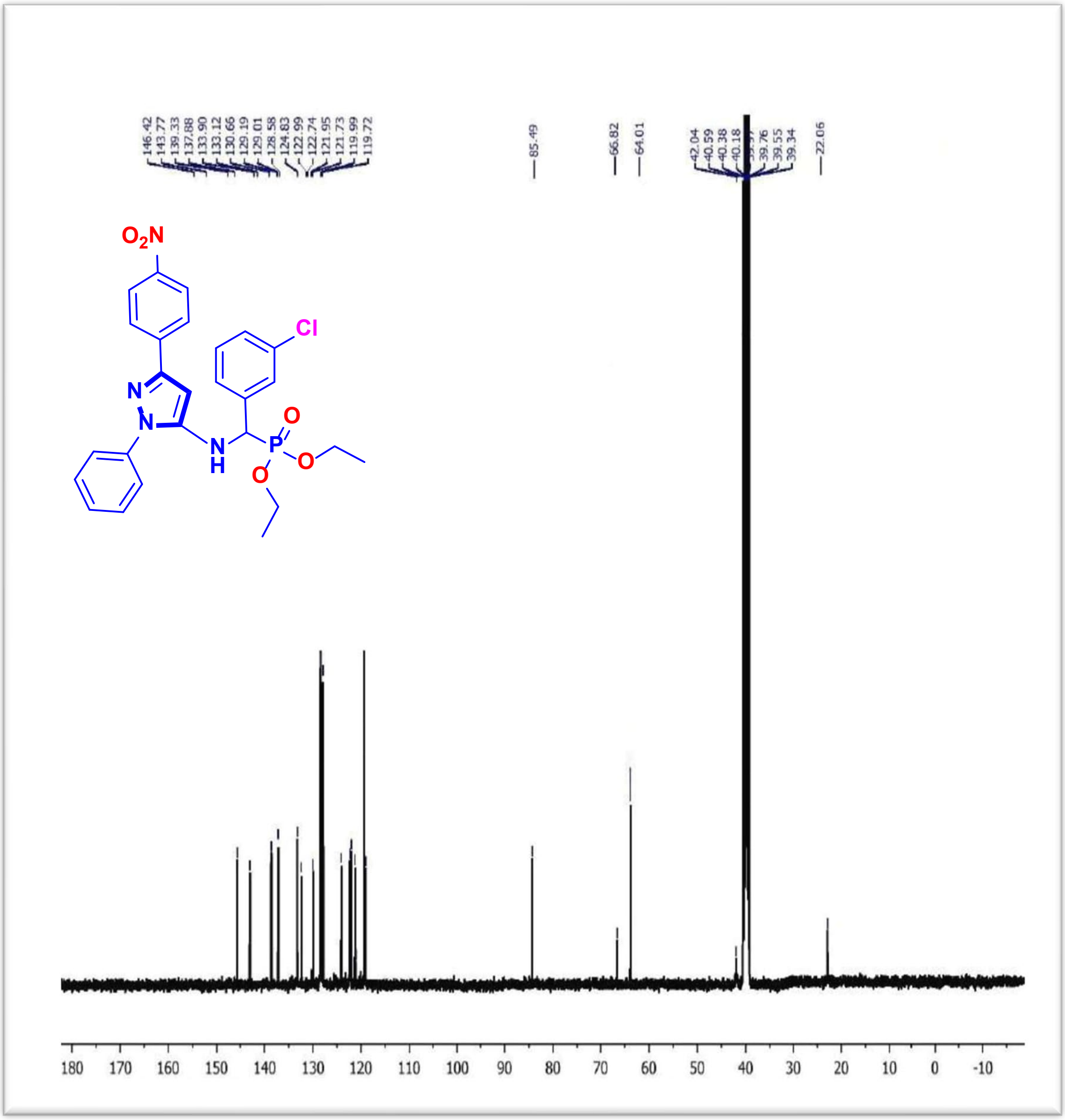

Fig. S8. ^{13}C-NMR spectrum of α-aminophosphonate **2c**.

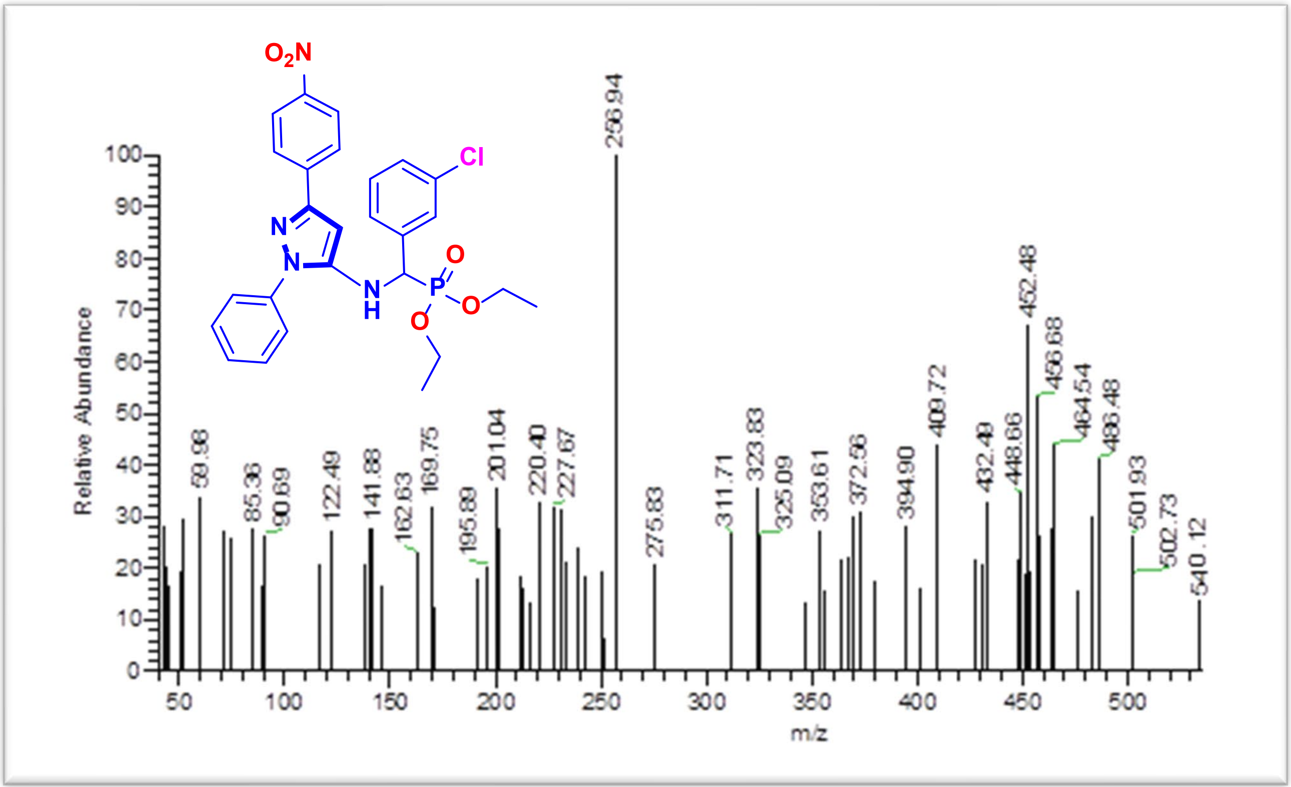

Fig. S9. Mass spectrum of α-aminophosphonate **2c**

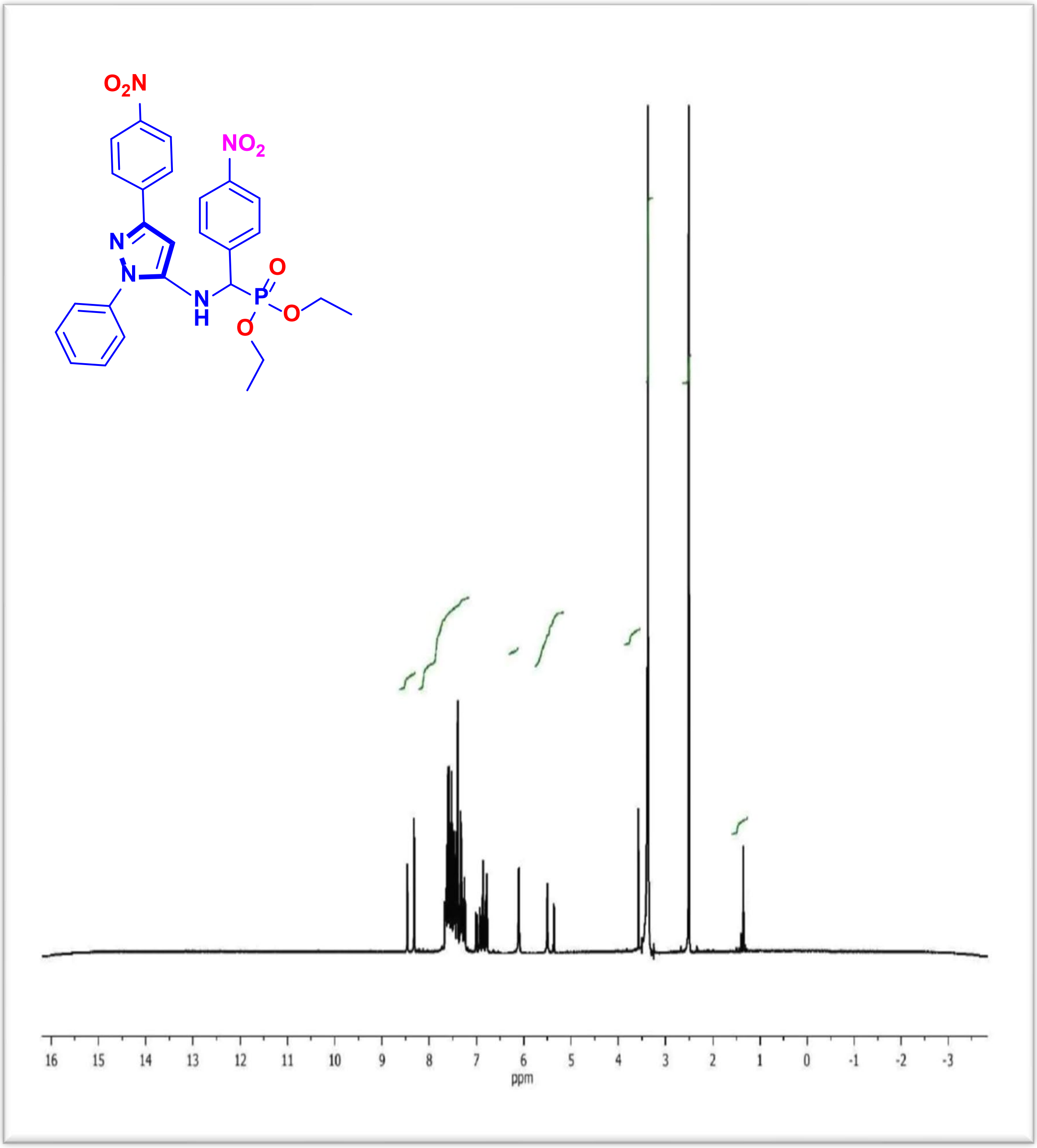

Fig. S10. ^{1}H-NMR spectrum of α-aminophosphonate **2d**.

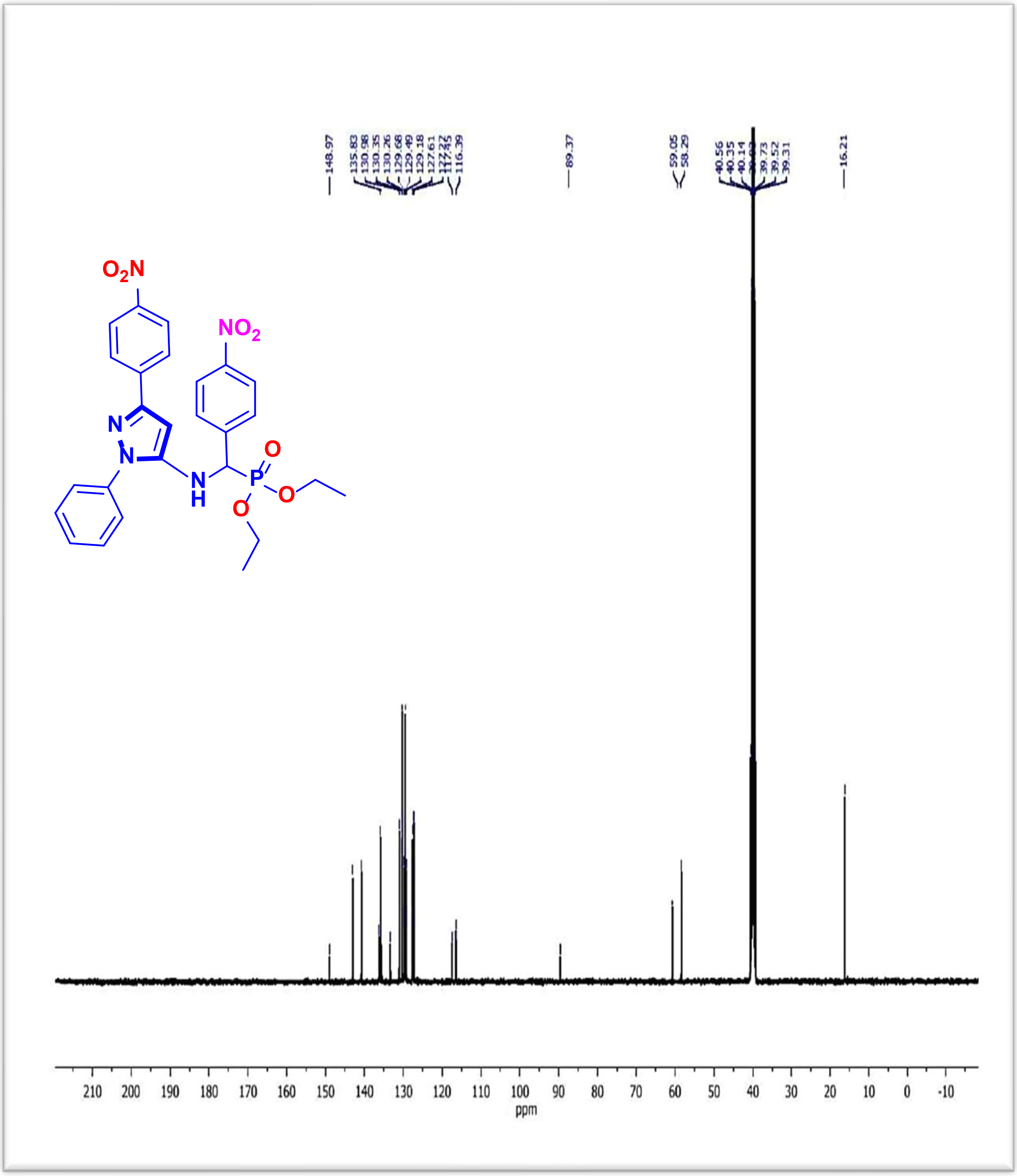

Fig. S11. ^{13}C-NMR spectrum of α-aminophosphonate **2d**.

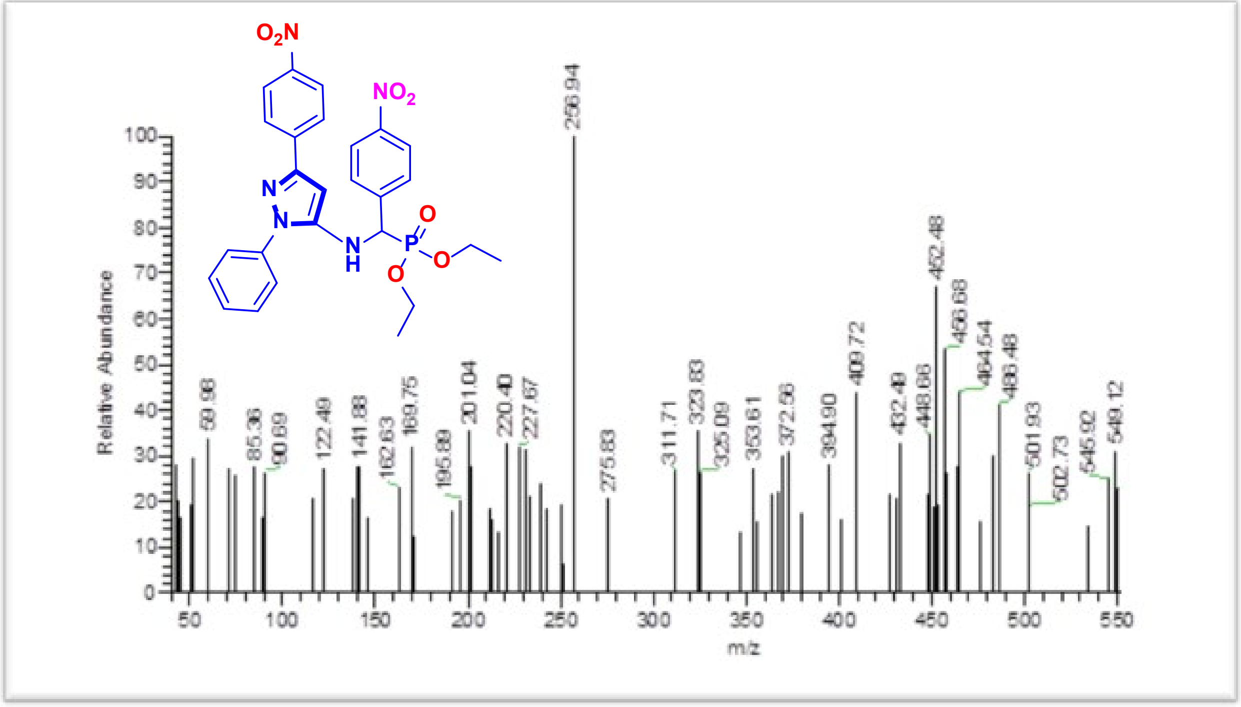

Fig. S12. Mass spectrum of α-aminophosphonate **2d**

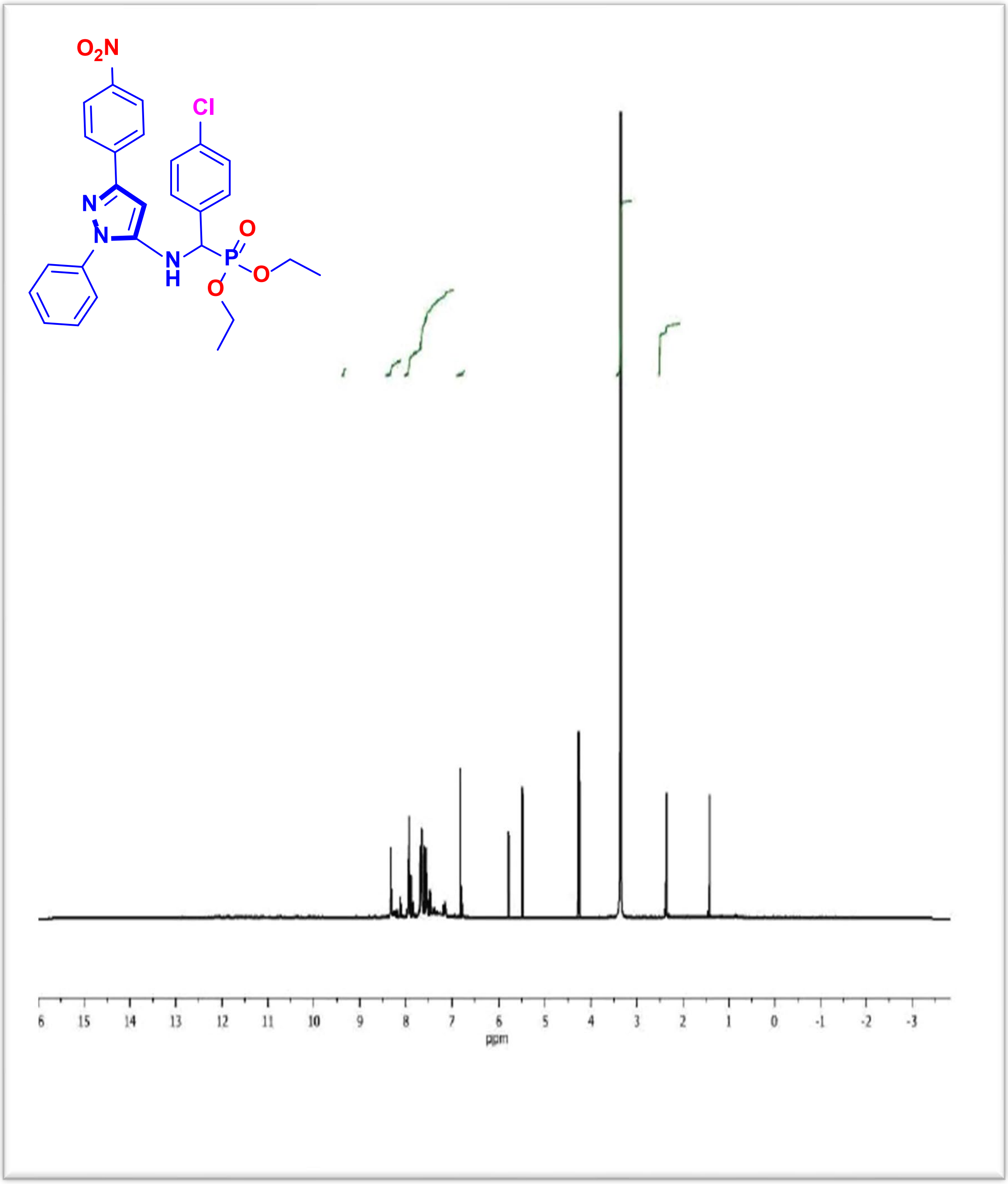

Fig. S13. ^{1}H-NMR spectrum of α-aminophosphonate **2e**.

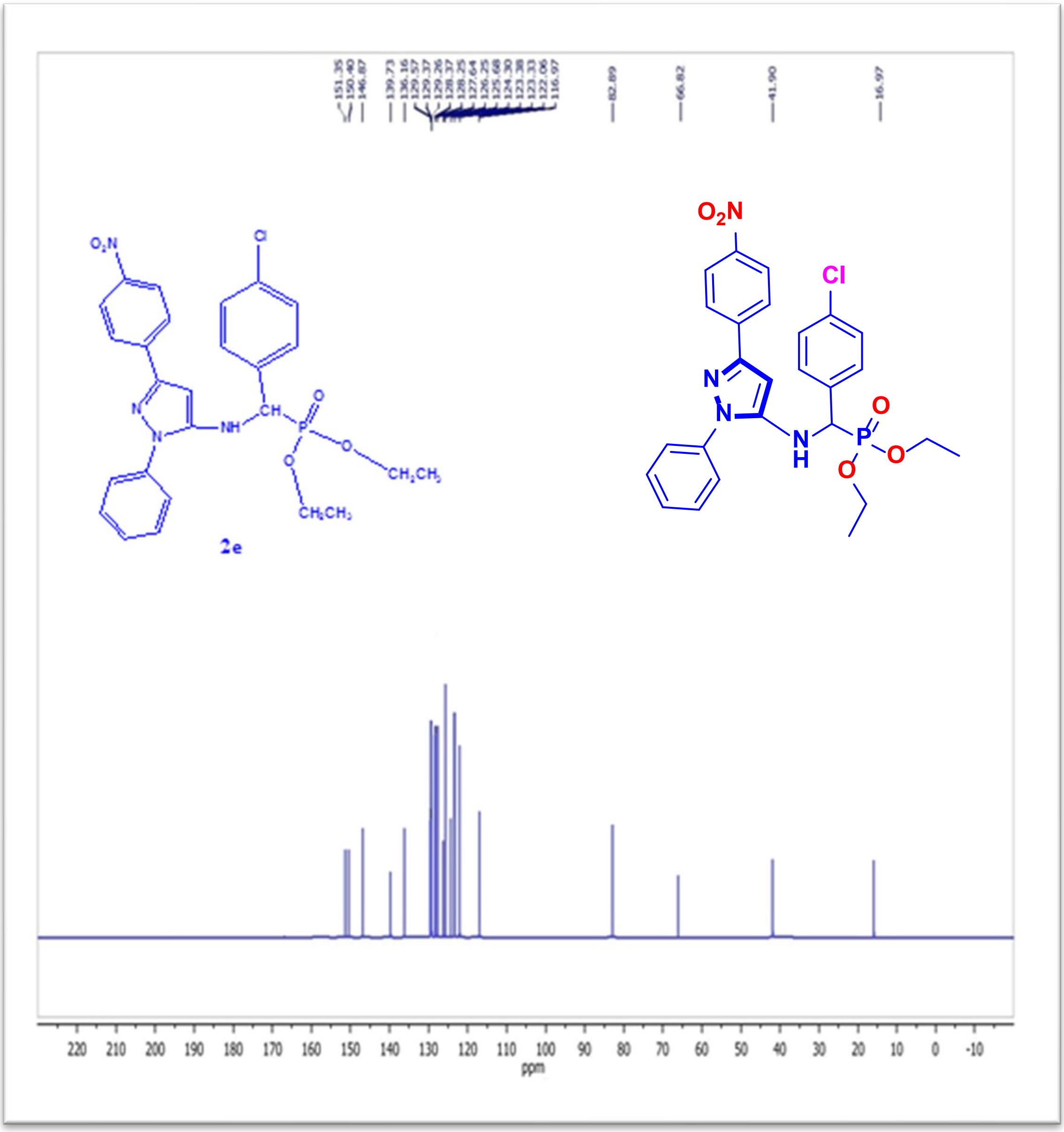

Fig. S14. ^{13}C-NMR spectrum of α-aminophosphonate **2e**.

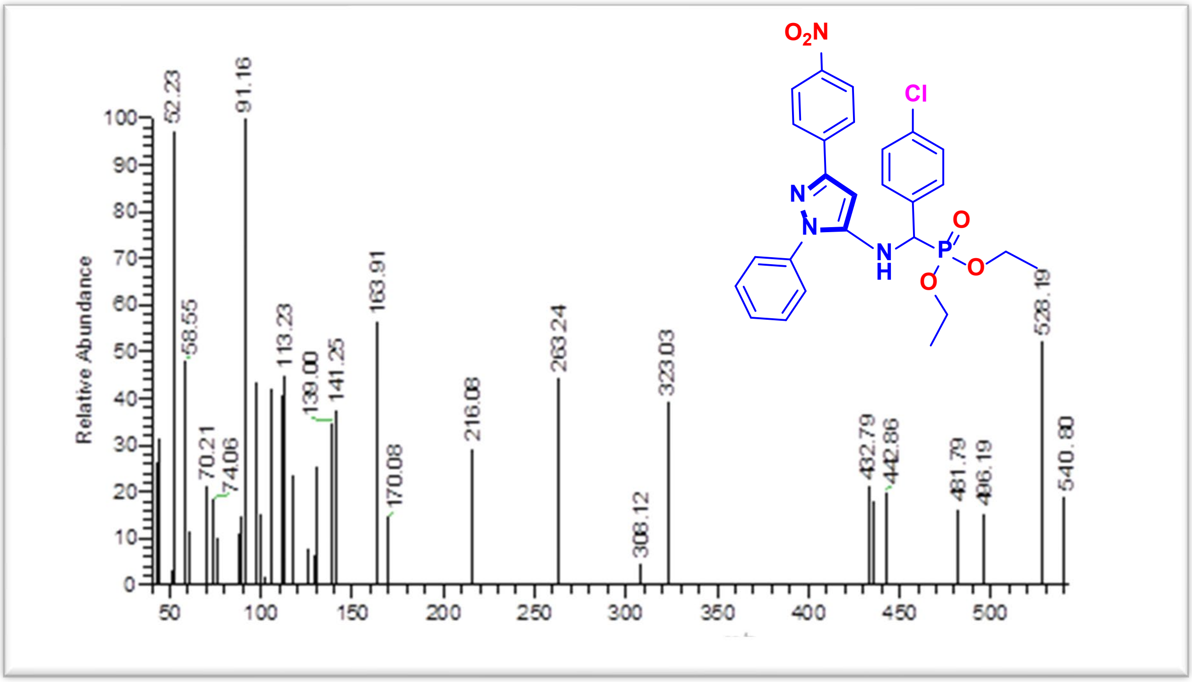

Fig. S15. Mass spectrum of α-aminophosphonate **2e**

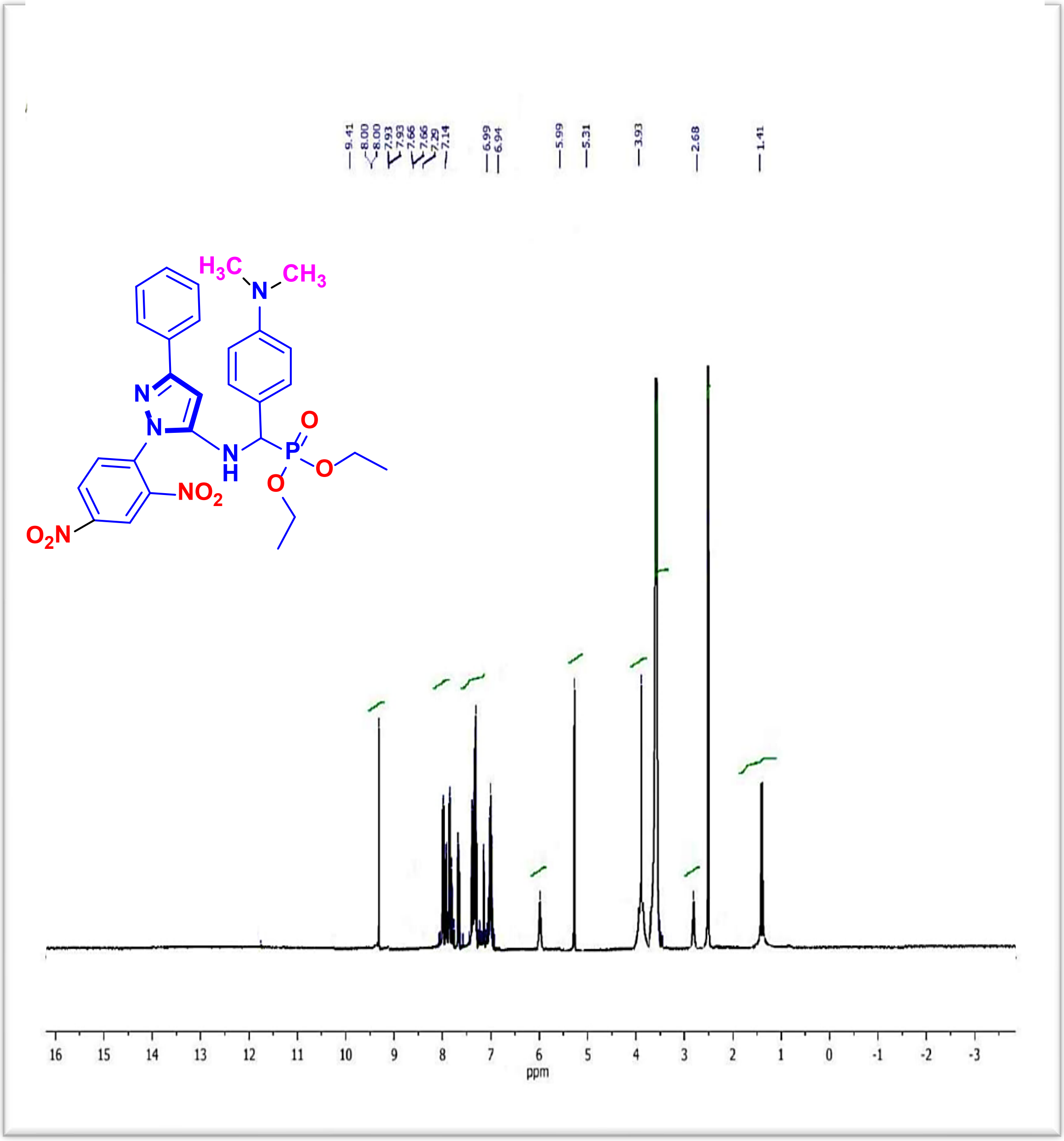

Fig. S16. ^{1}H-NMR spectrum of α-aminophosphonate **2f**.

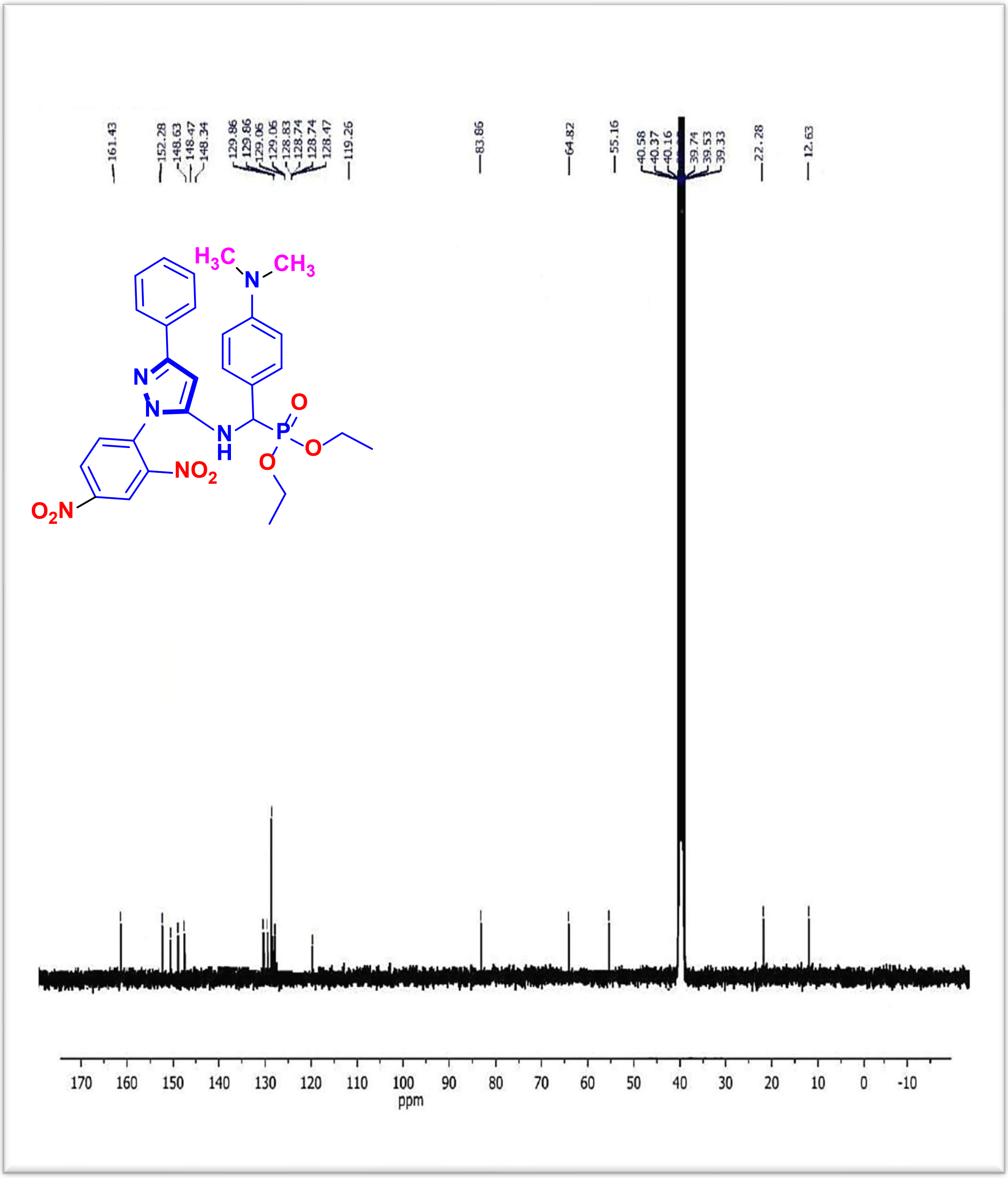

Fig. S17. ^{13}C-NMR spectrum of α-aminophosphonate **2f**.

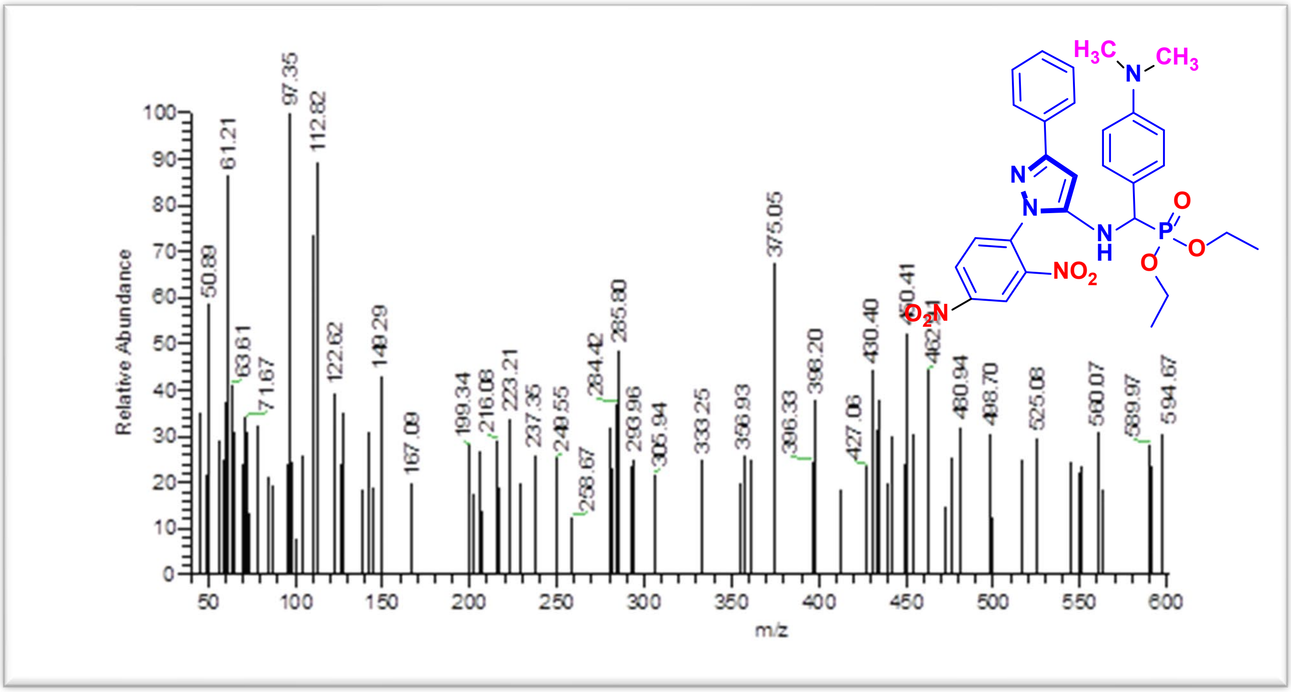

Fig. S18. Mass spectrum of α-aminophosphonate **2f**

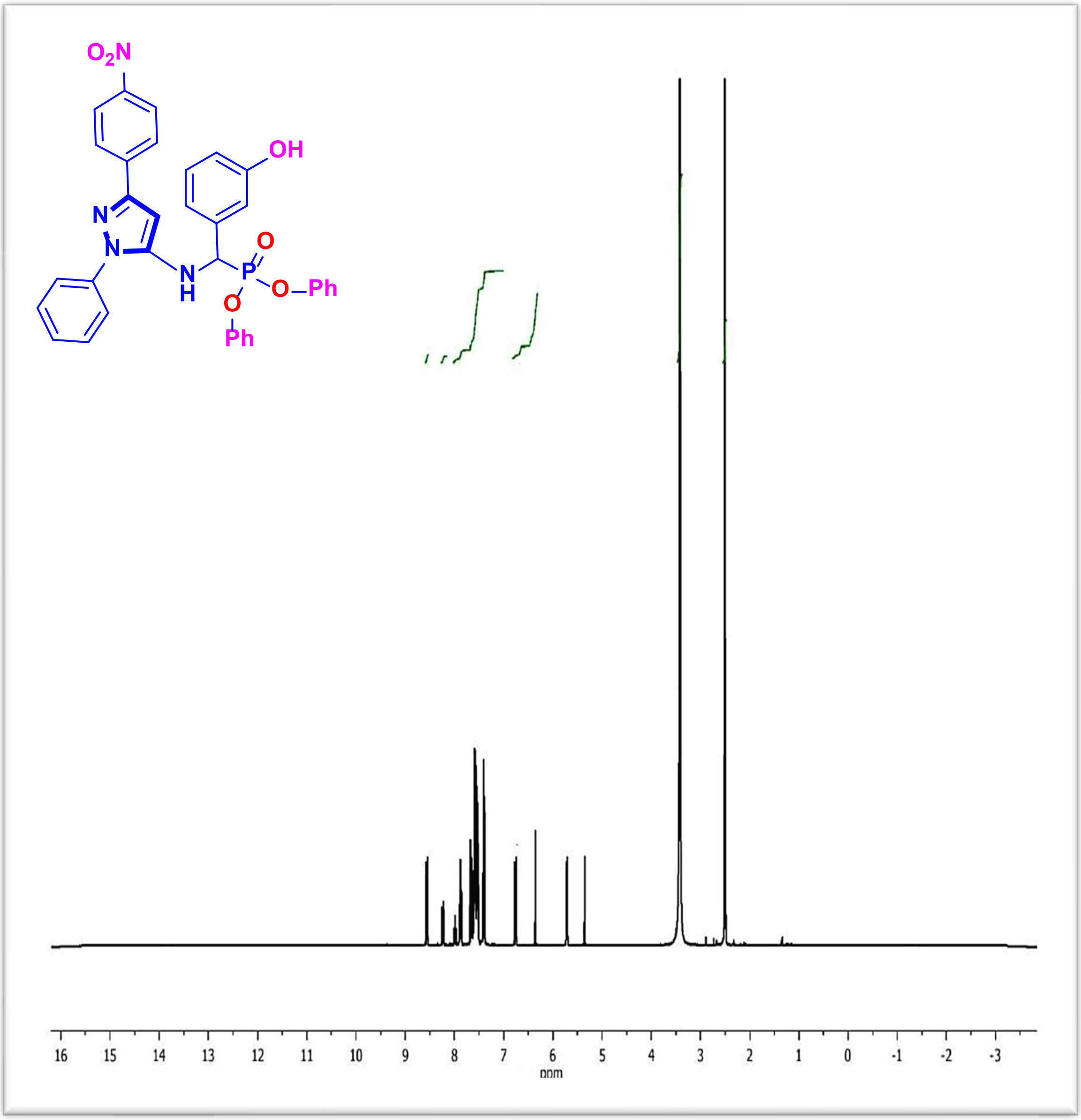

Fig. S19. ^{1}H-NMR spectrum of α-aminophosphonate **4a**.

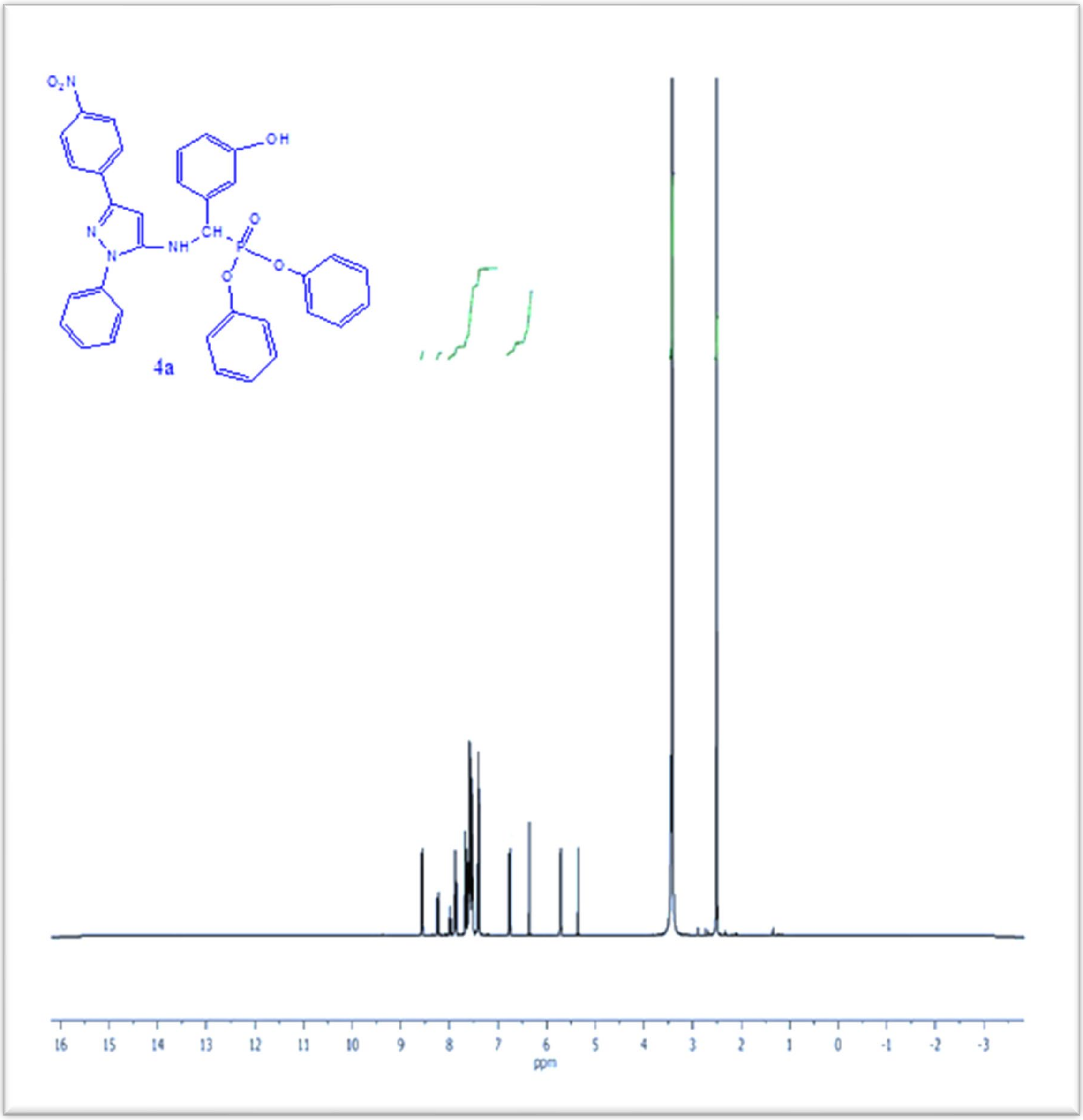

Fig. S20 ^{13}C-NMR spectrum of α-aminophosphonate **4a**.

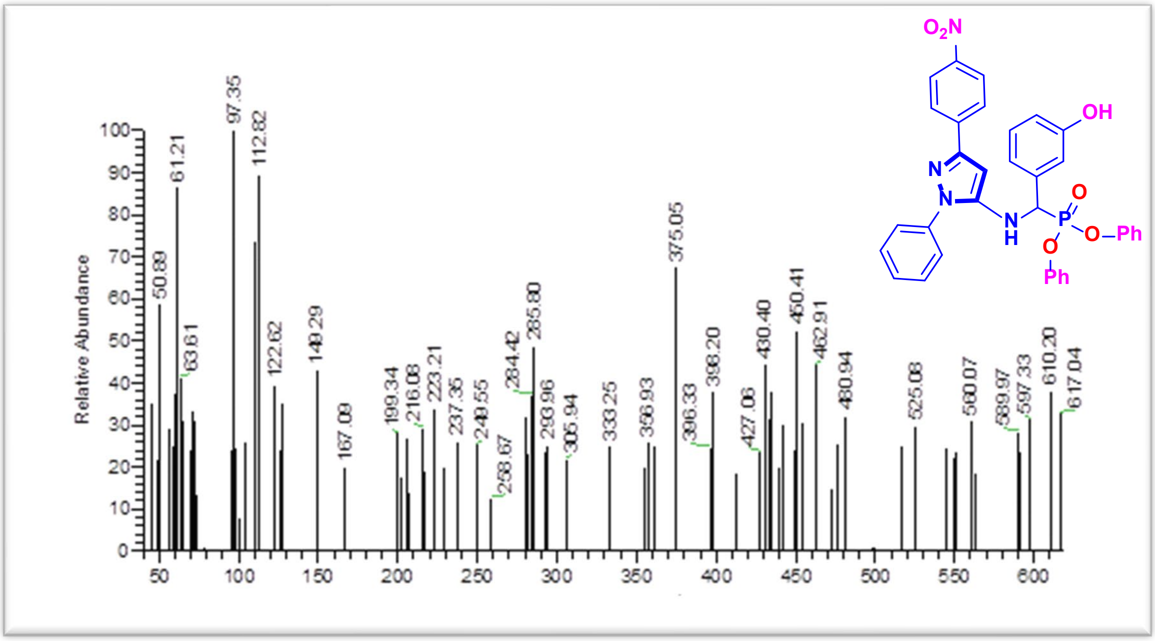

Fig. S21. Mass spectrum of α-aminophosphonate **4a**

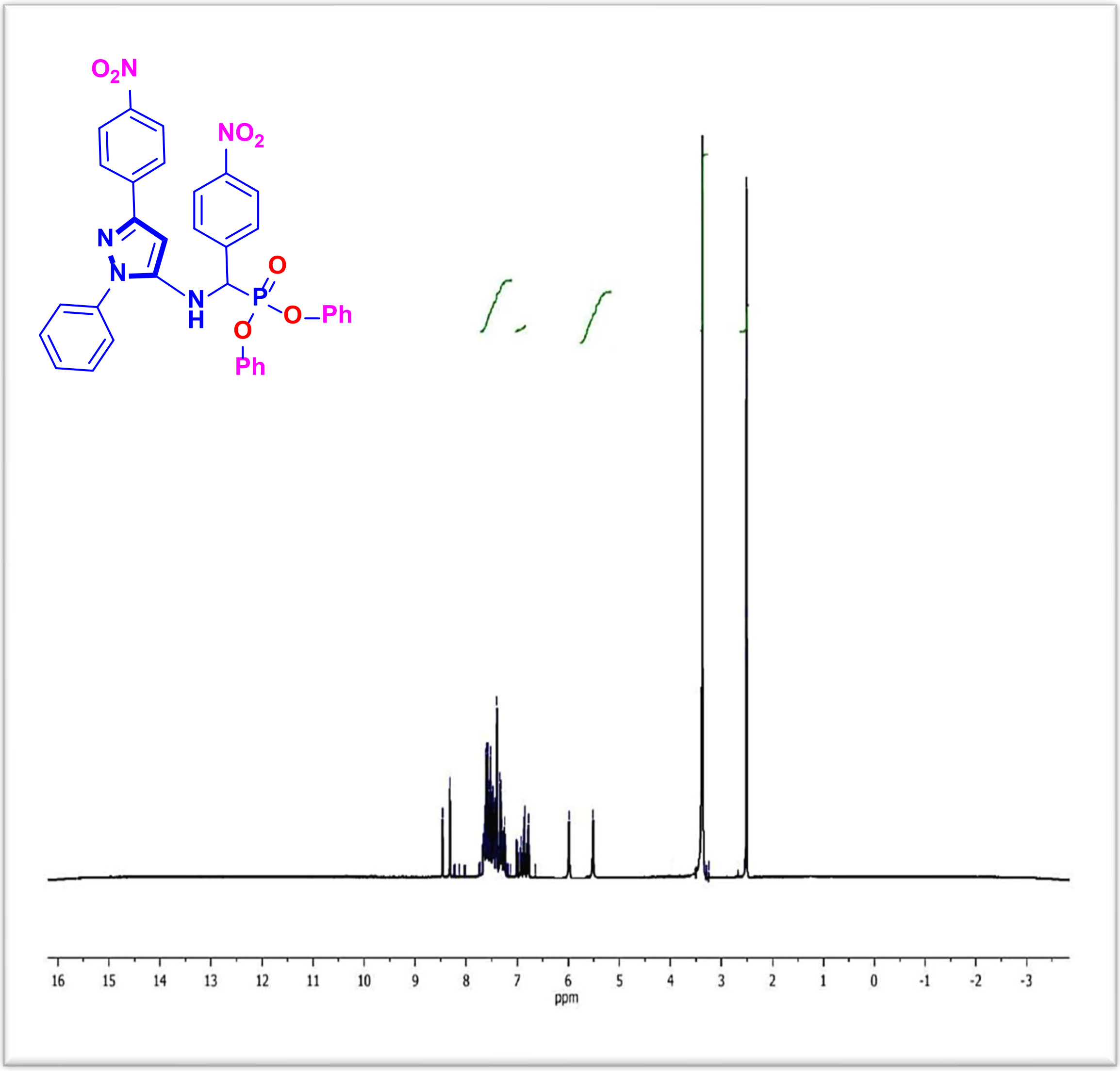

Fig. S22. ^{1}H-NMR spectrum of α-aminophosphonate **4b**.

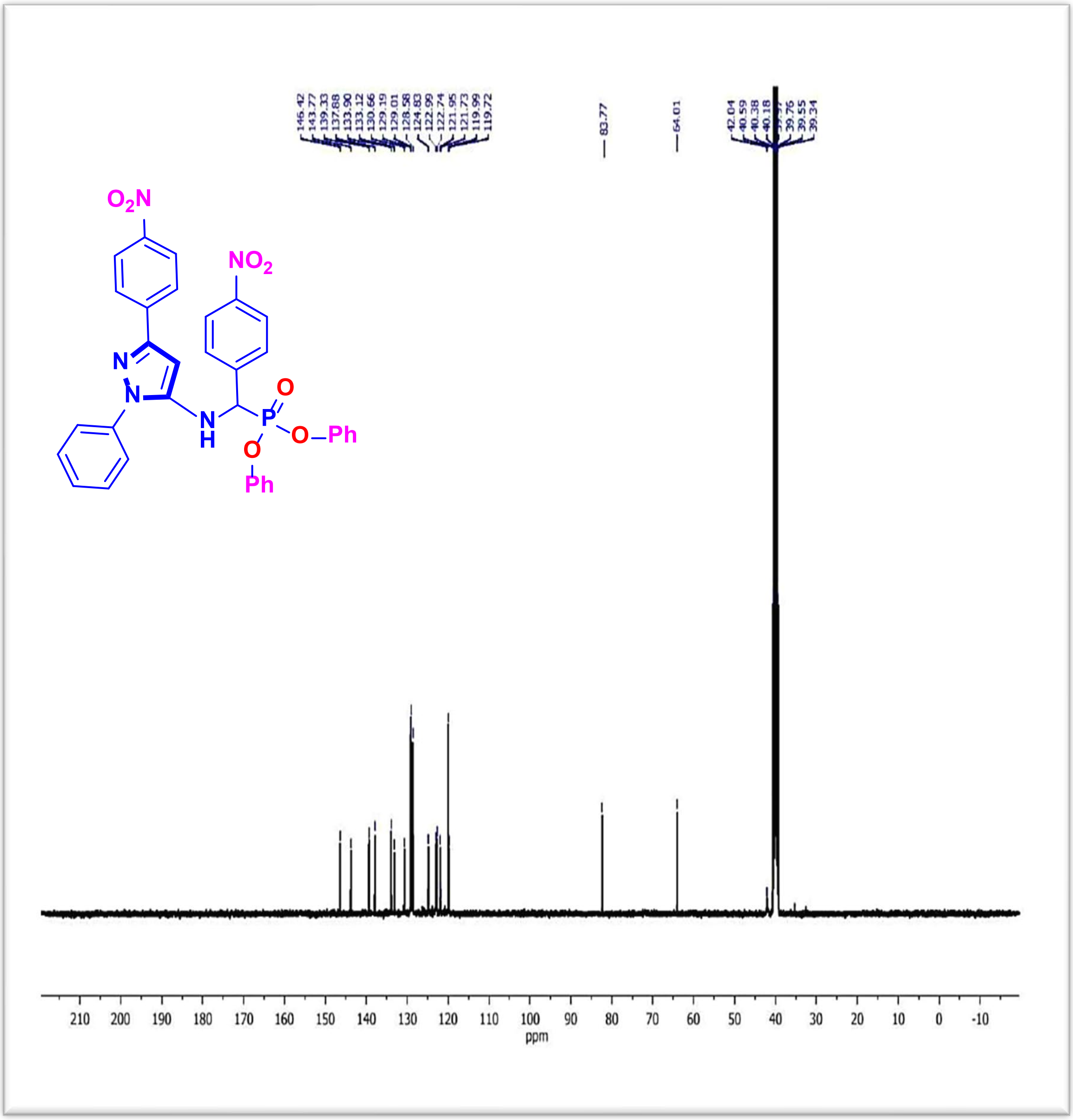

Fig. S23. ^{13}C-NMR spectrum of α-aminophosphonate **4b**.

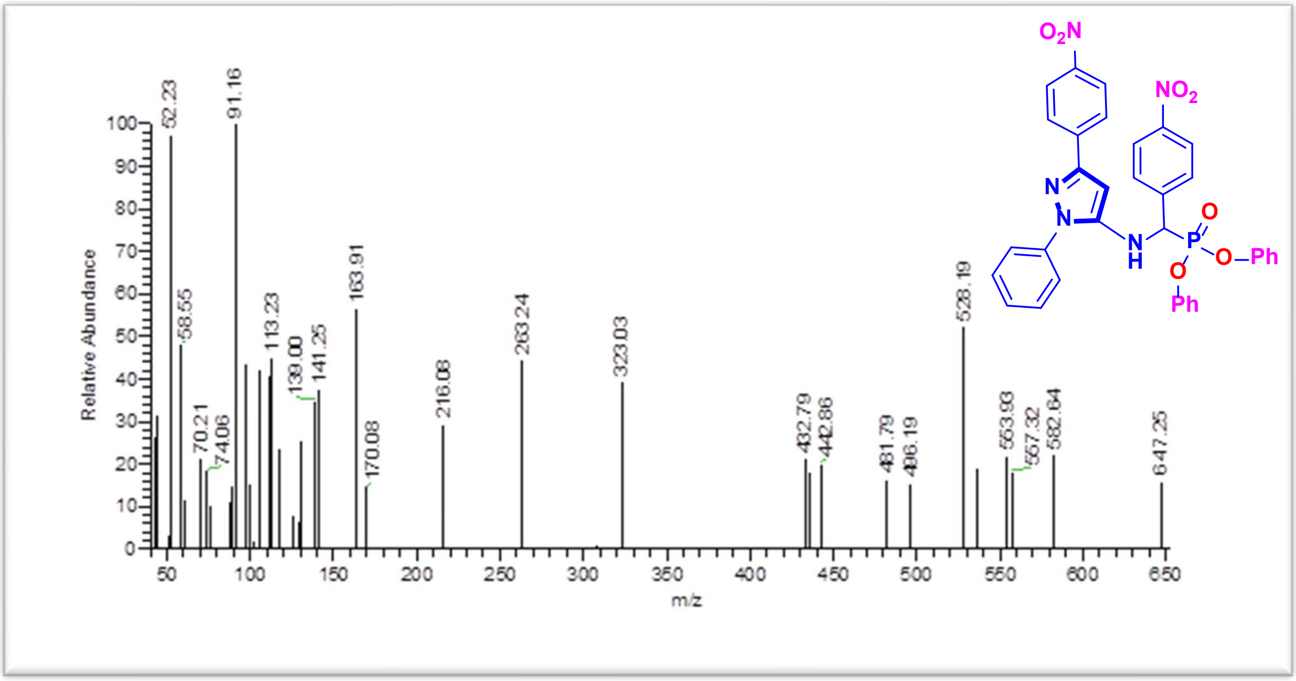

Fig. S24. Mass spectrum of α-aminophosphonate **4b**

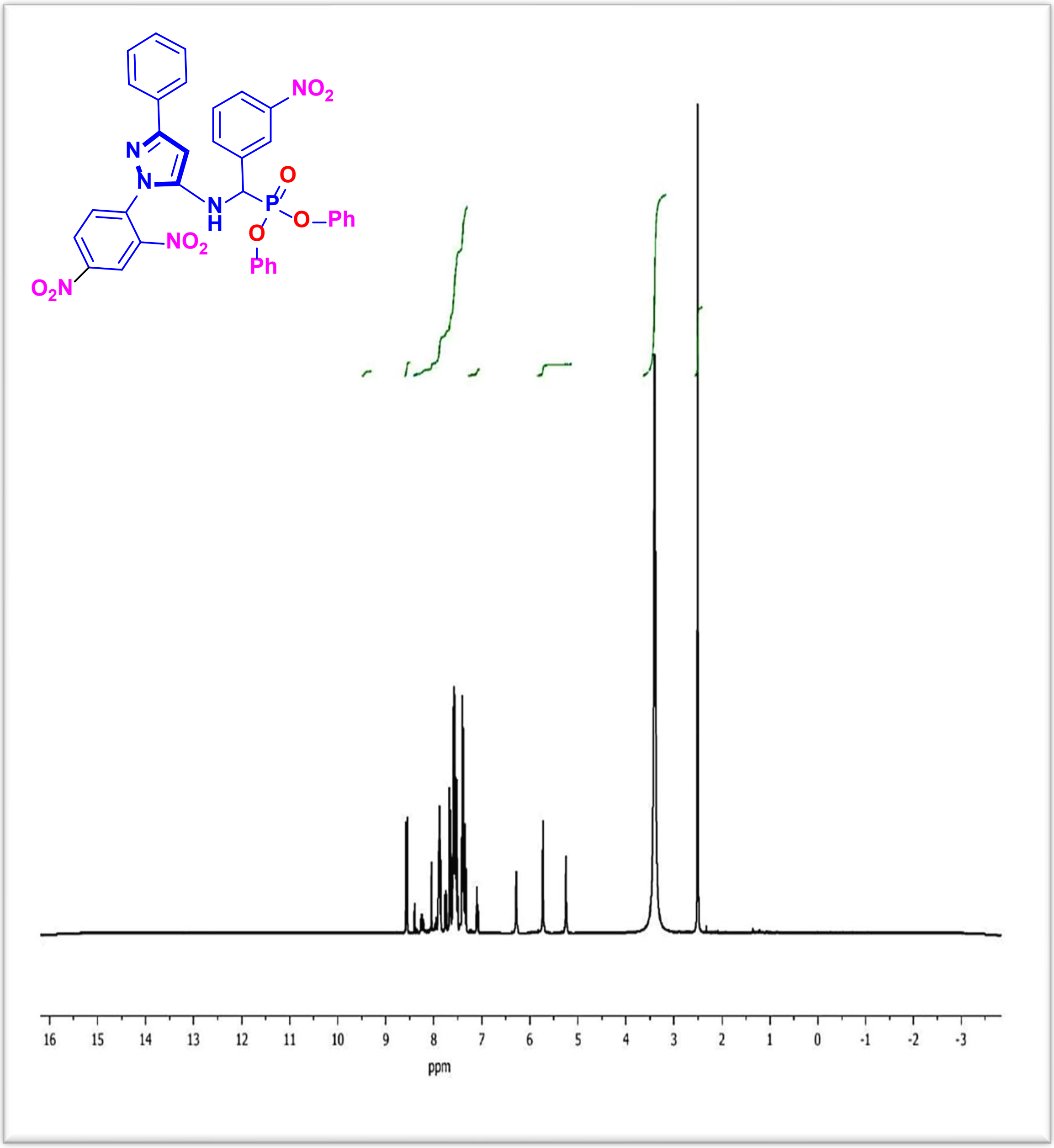

Fig. S25. ^{1}H-NMR spectrum of α-aminophosphonate **4c**.

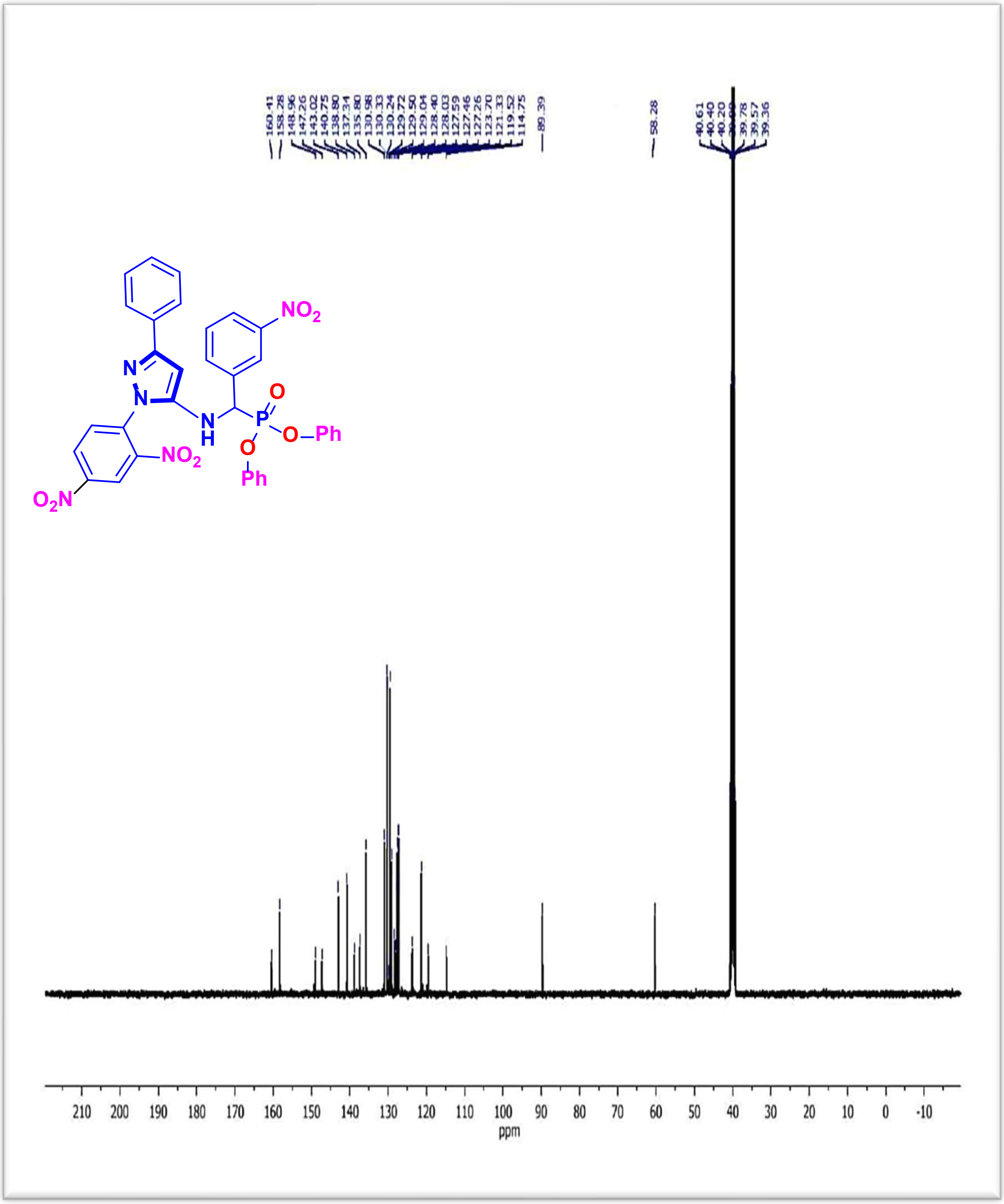

Fig. S26. ^{13}C-NMR spectrum of α-aminophosphonate **4c**.

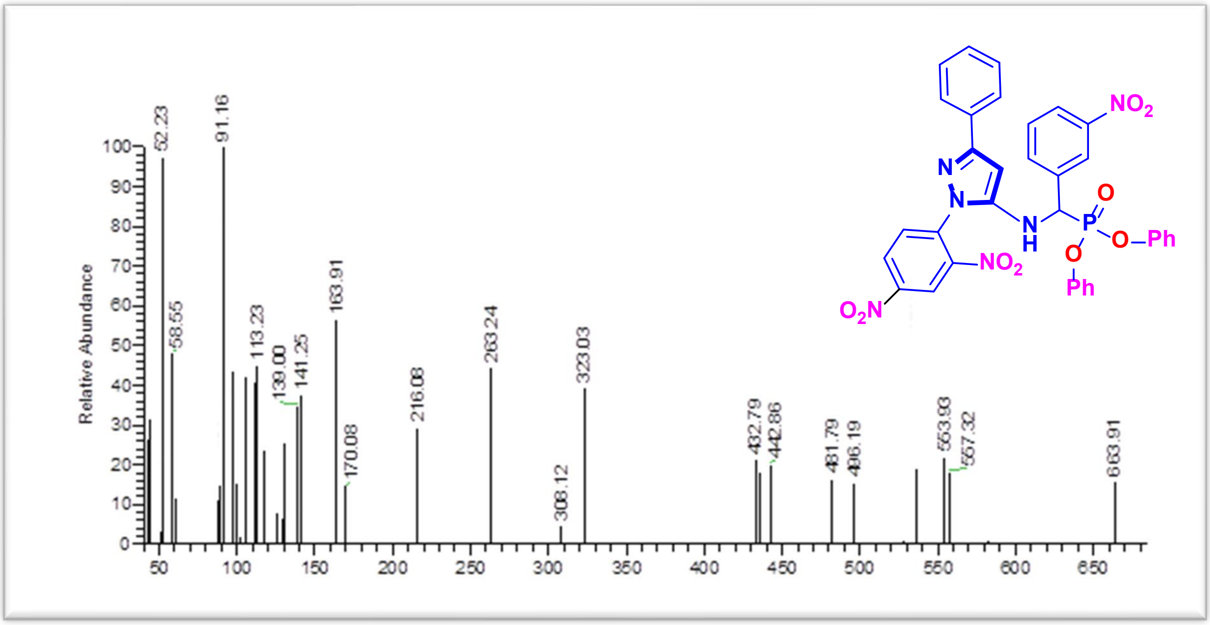

Fig. S27. Mass spectrum of α-aminophosphonate **4c**

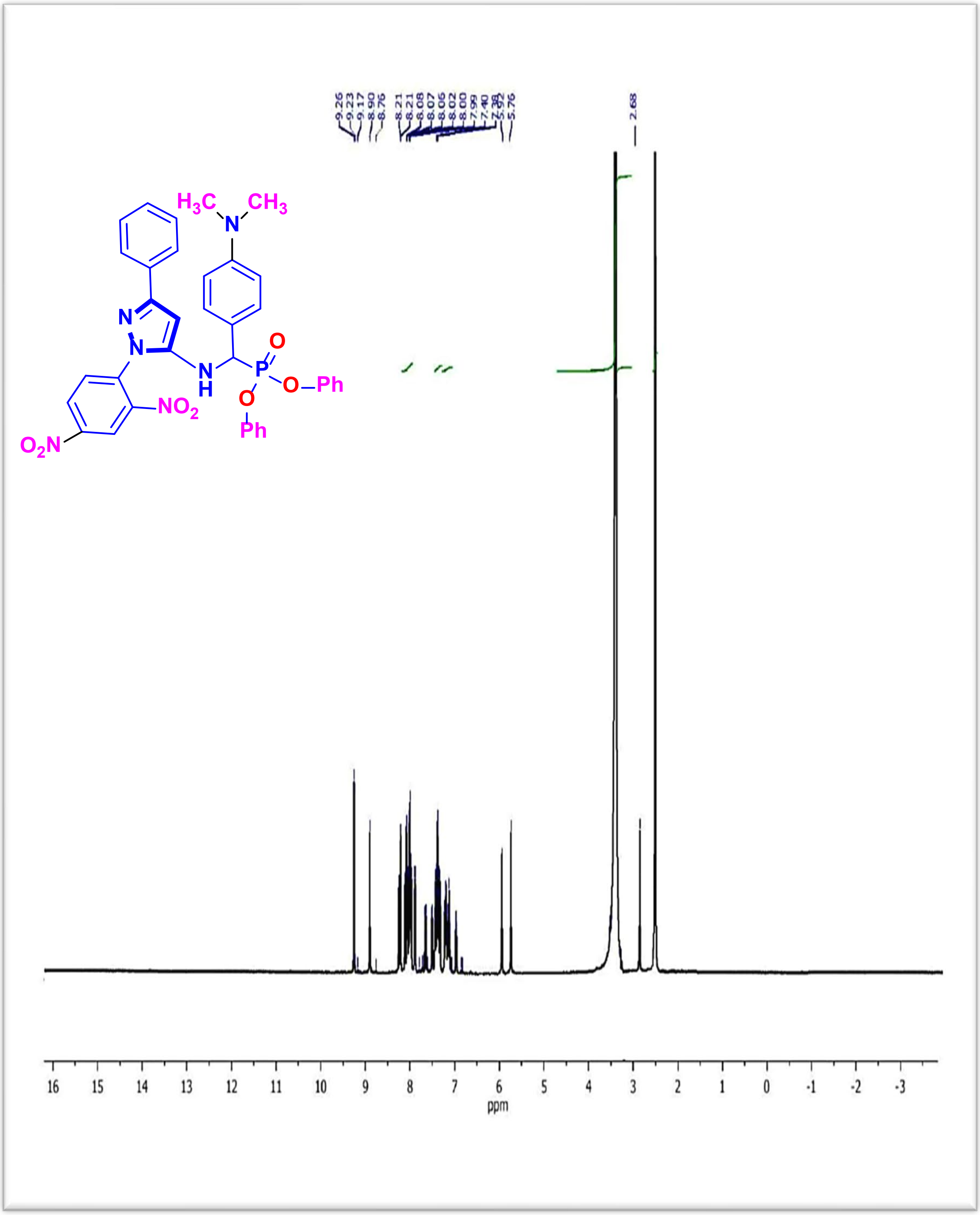

Fig. S28. ^{1}H-NMR spectrum of α-aminophosphonate **4d**.

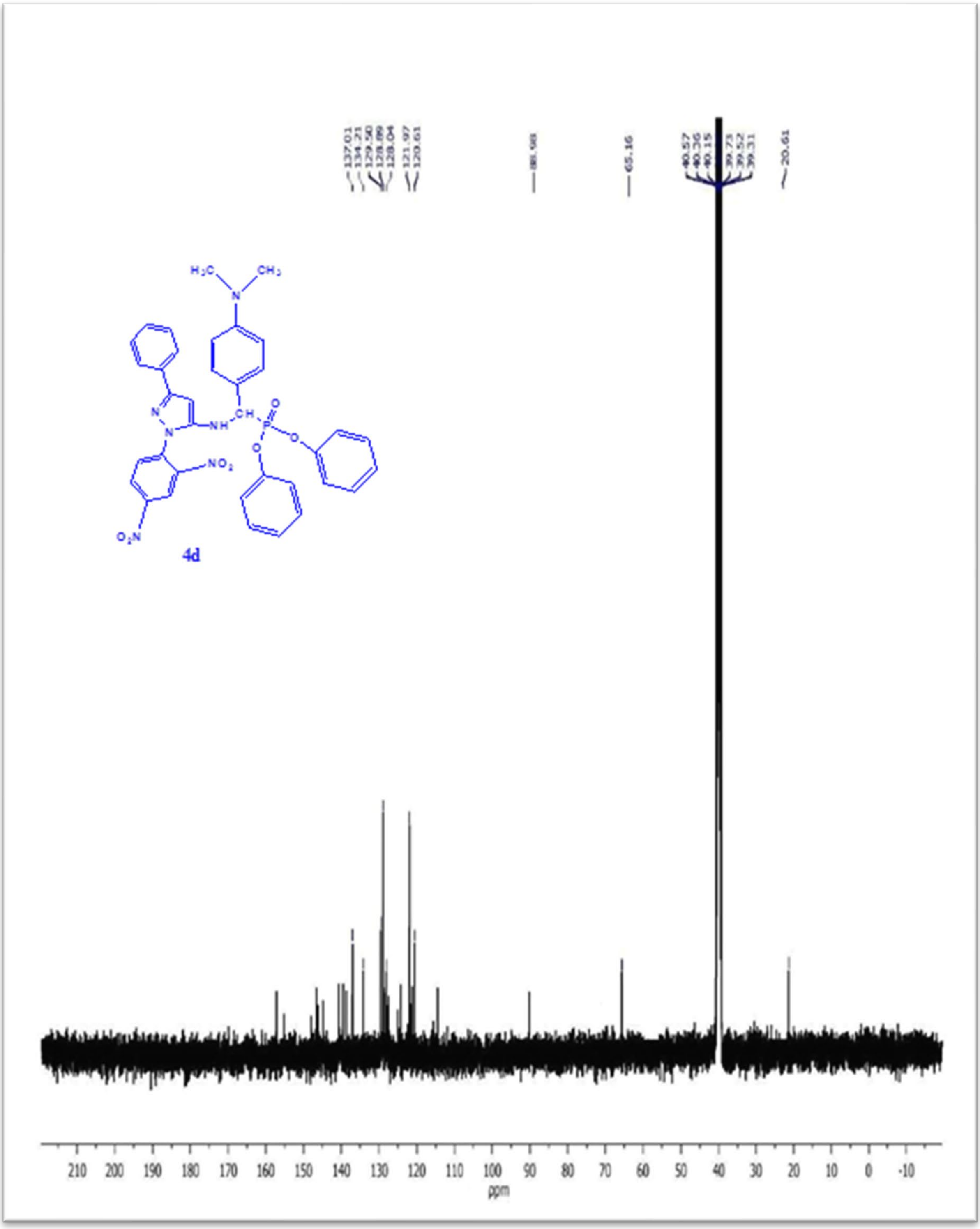

Fig. S29. ^{13}C-NMR spectrum of α-aminophosphonate **4d**.

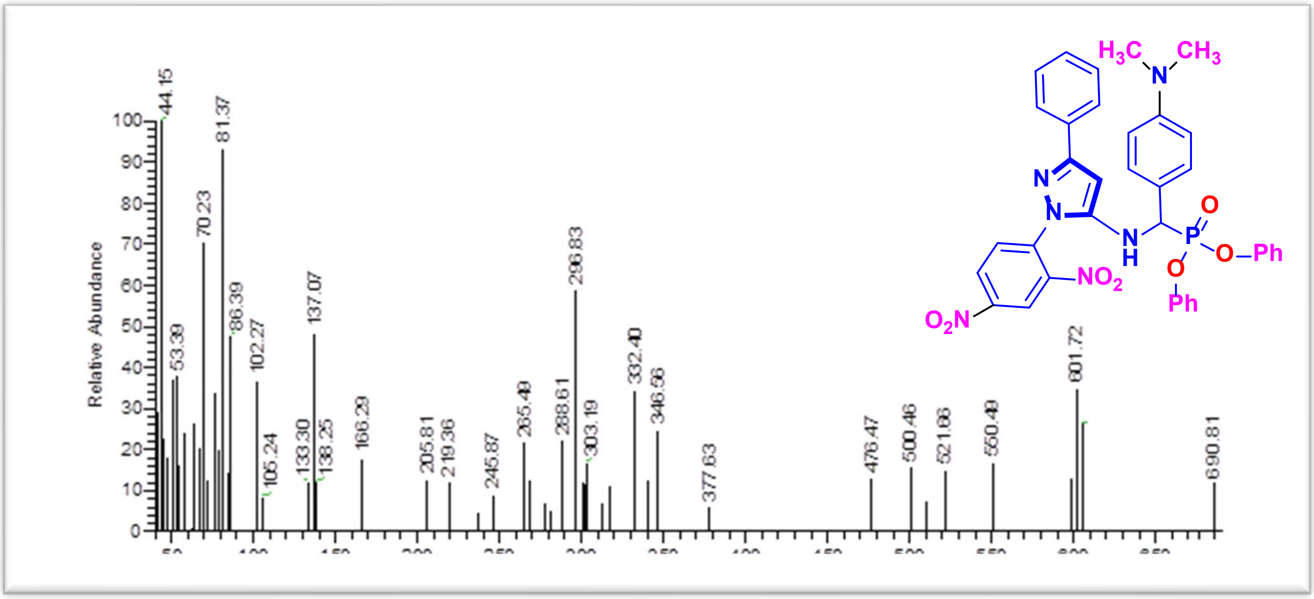

Fig. S30. Mass spectrum of α-aminophosphonate **4d**

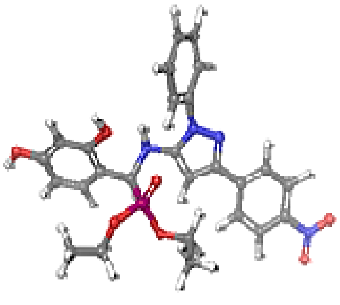

2a

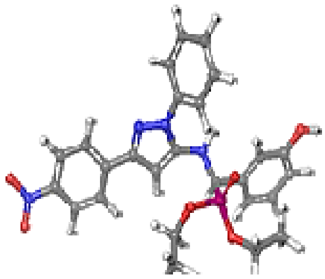

2b

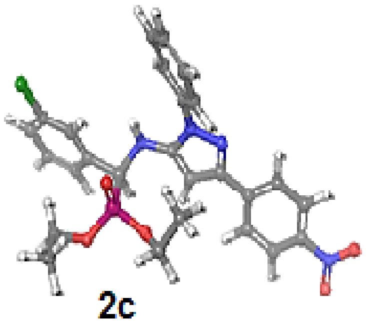

2c

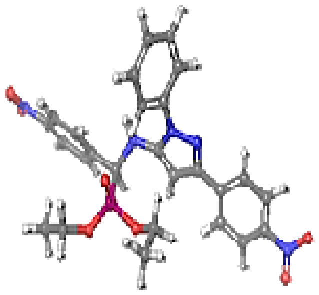

2d

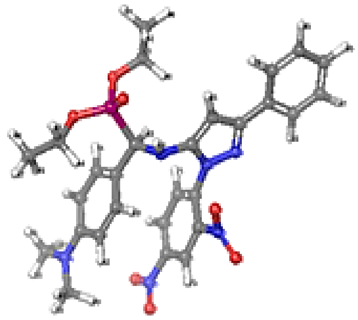

2f

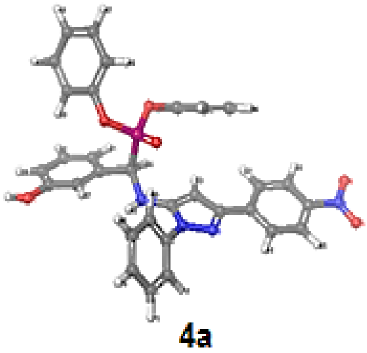

4a

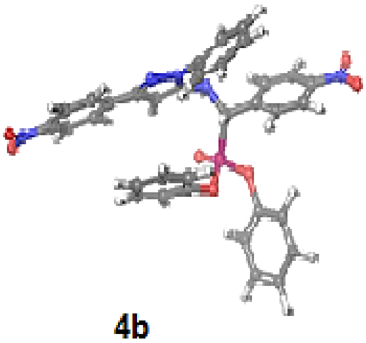

4b

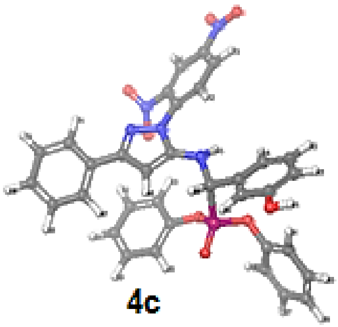

4c

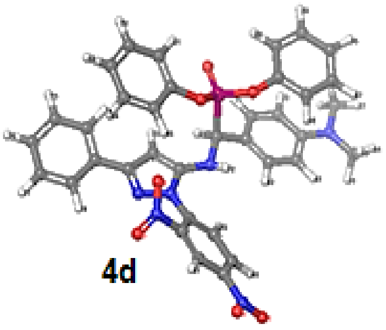

4d

Fig.S31. Optimized molecular structure of α-aminophosphonates.

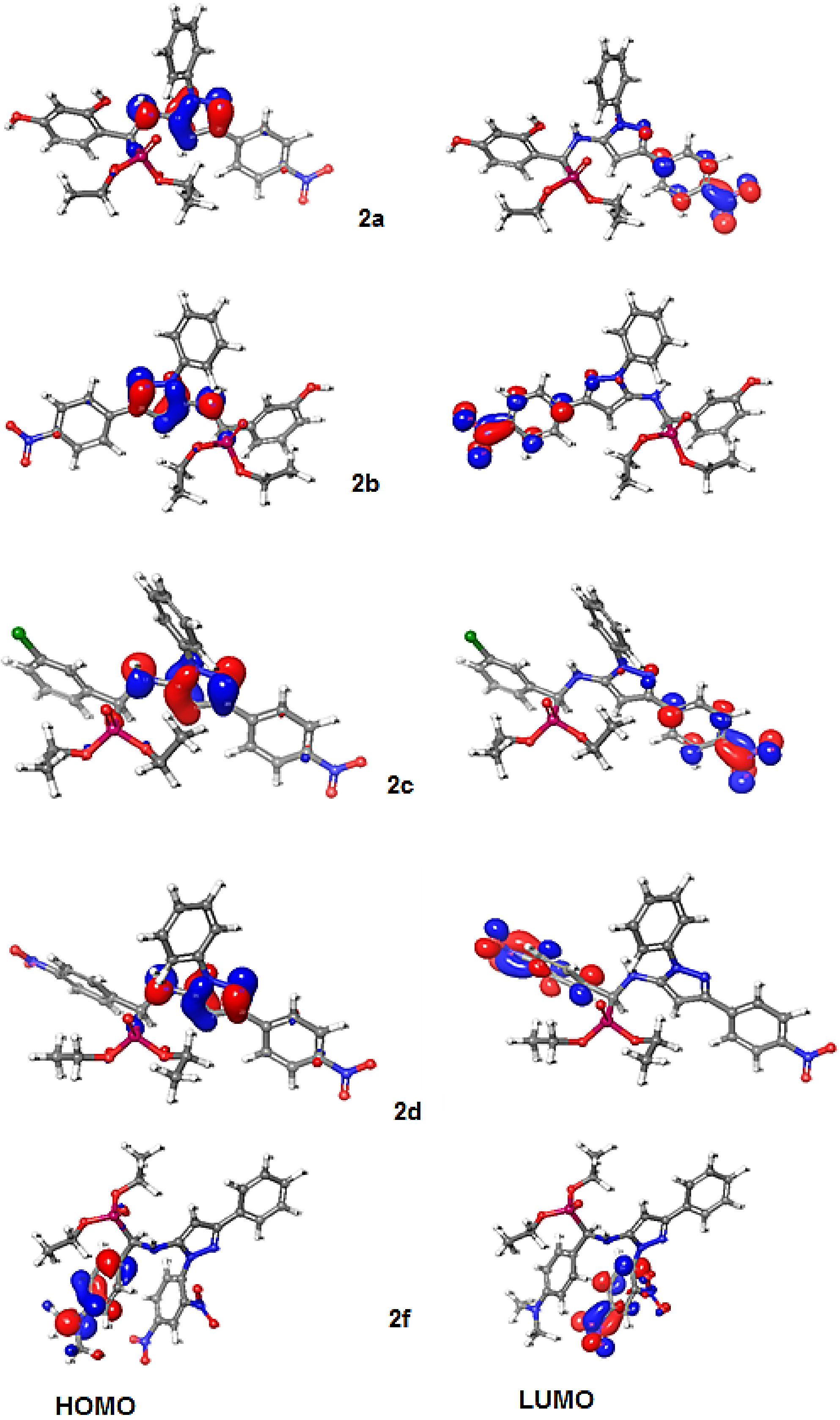

Fig. S32. 3D plots frontier orbital energies HOMO and LUMO using DFT method for α-aminophosphonate **2a-2f**.

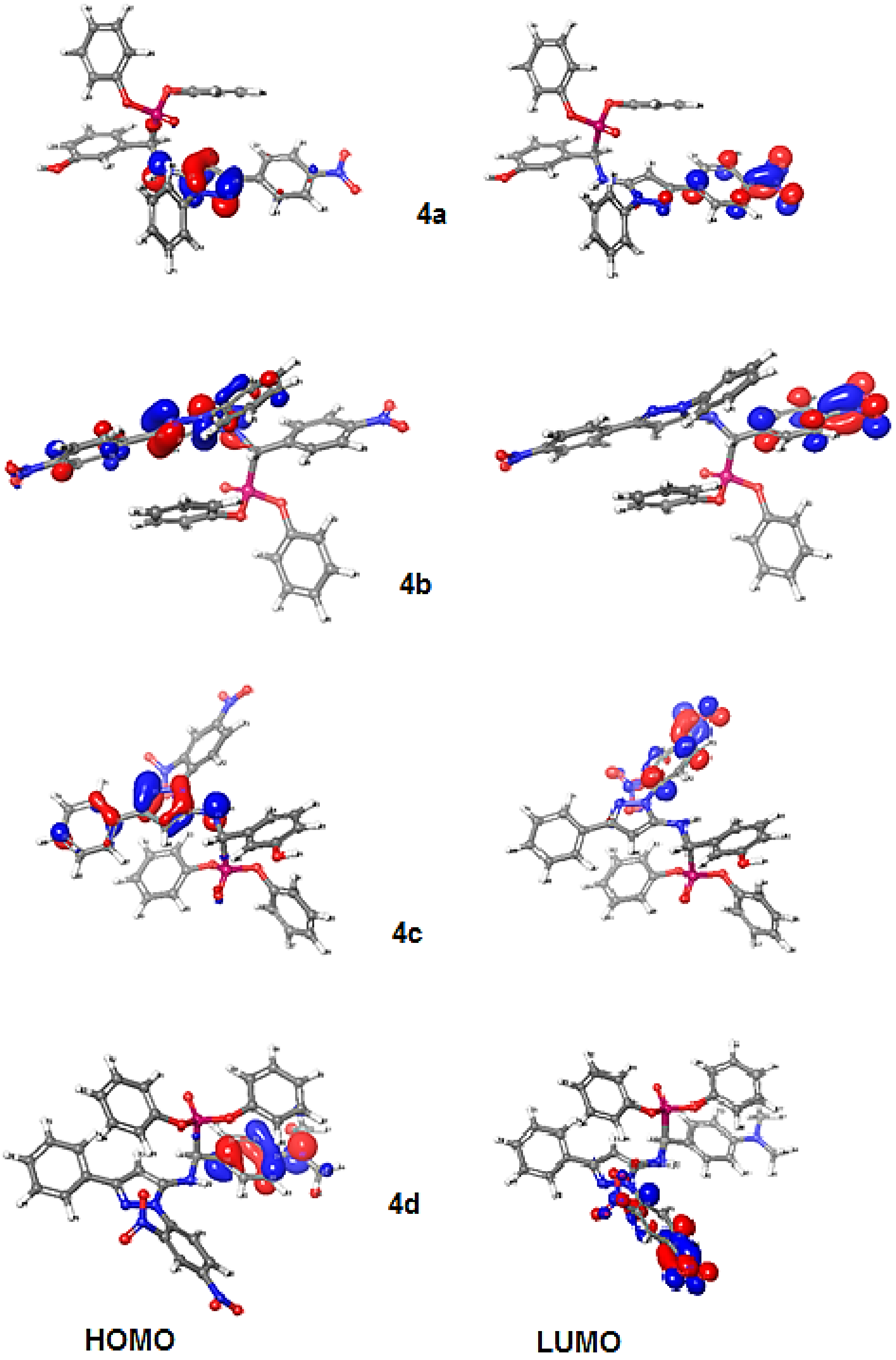

Fig. S33. 3D plots frontier orbital energies HOMO and LUMO using DFT method for α-aminophosphonate **4a-4d**.

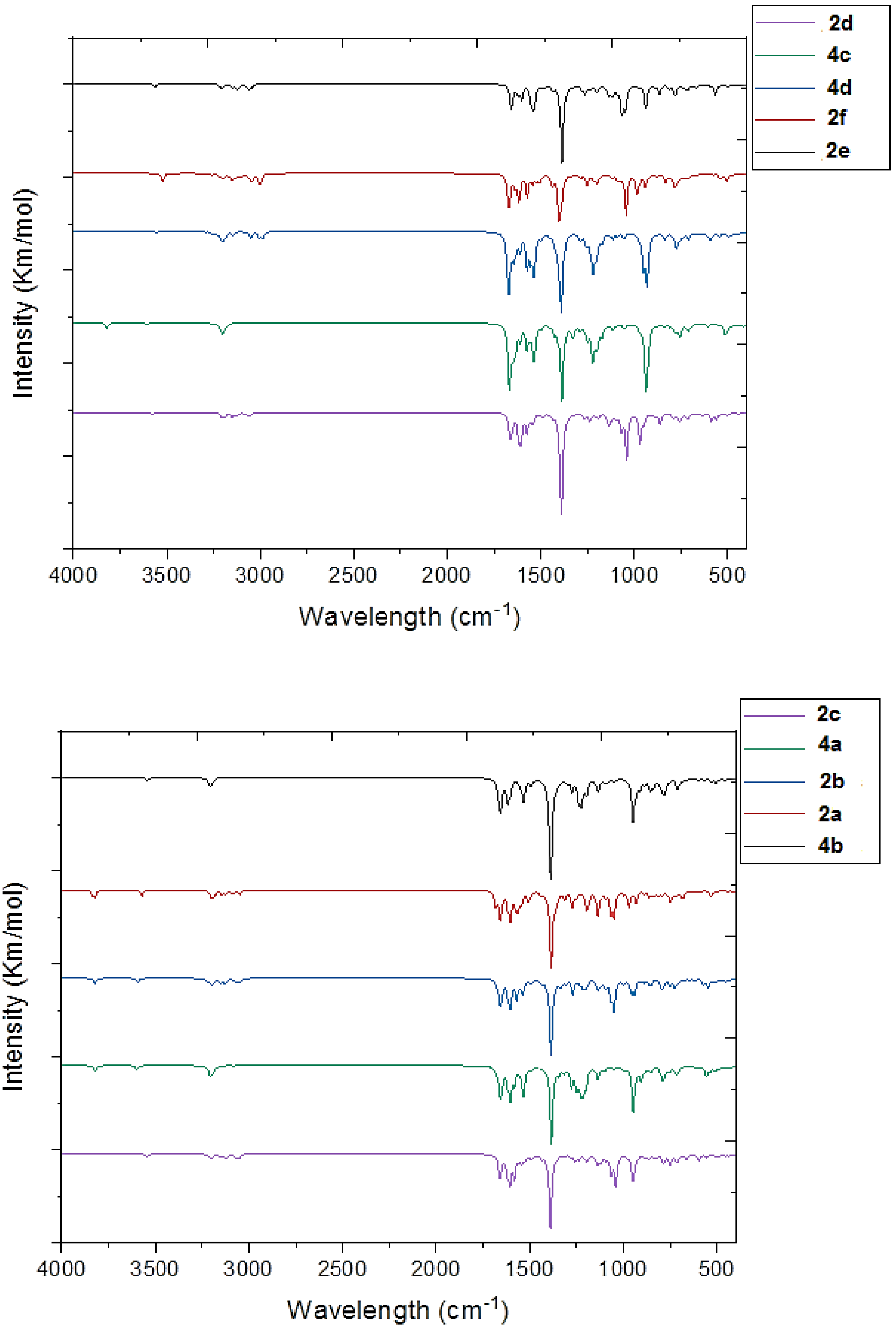

Fig. S34. the calculated IR spectra of α-aminophosphonates **2a-2f** and **4a-4d**.

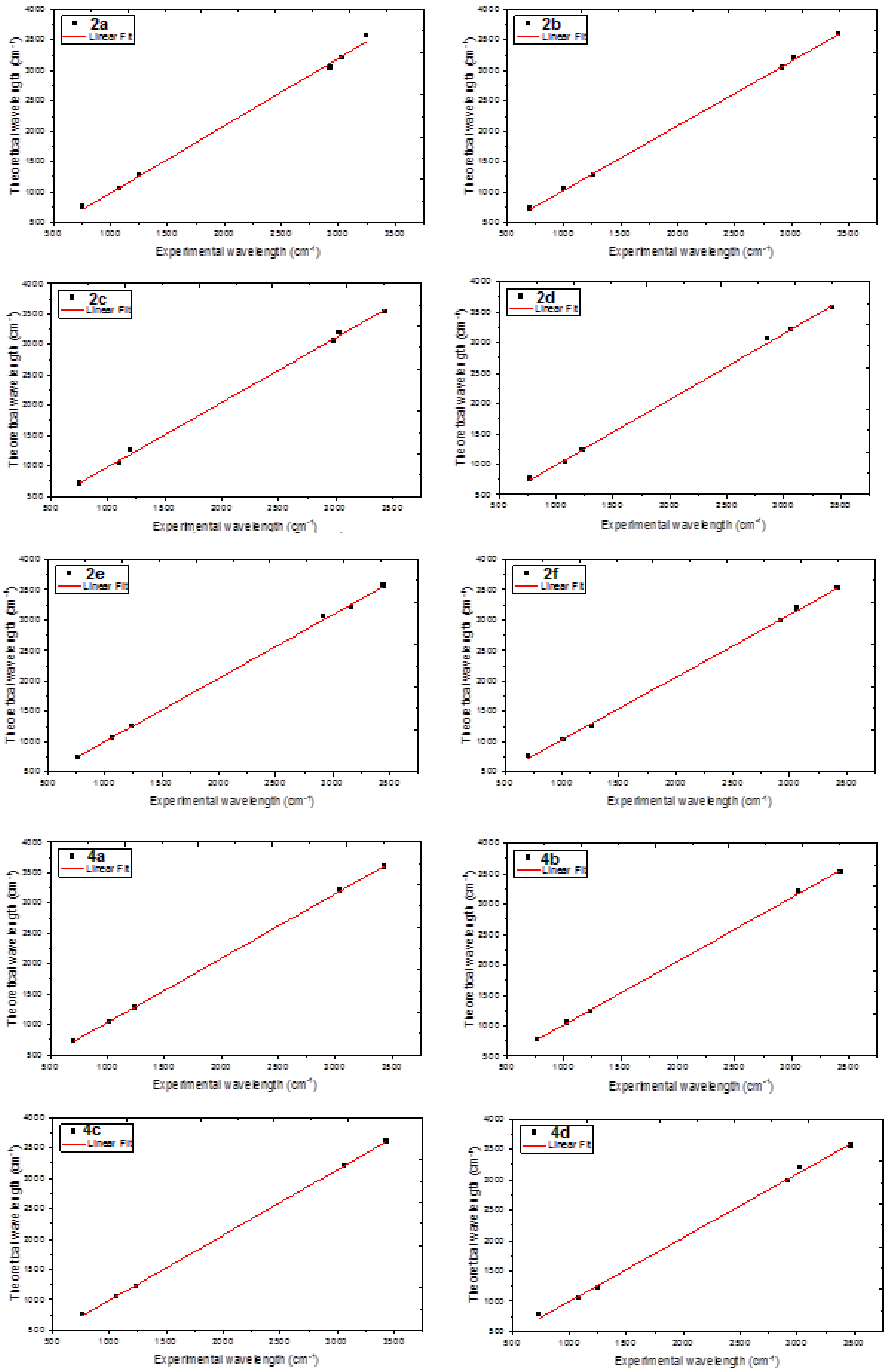

Fig. S35. The linear regression between the experimental and theoretical frequencies of α-aminophosphonates **2a-2f** and **4a-4d**.

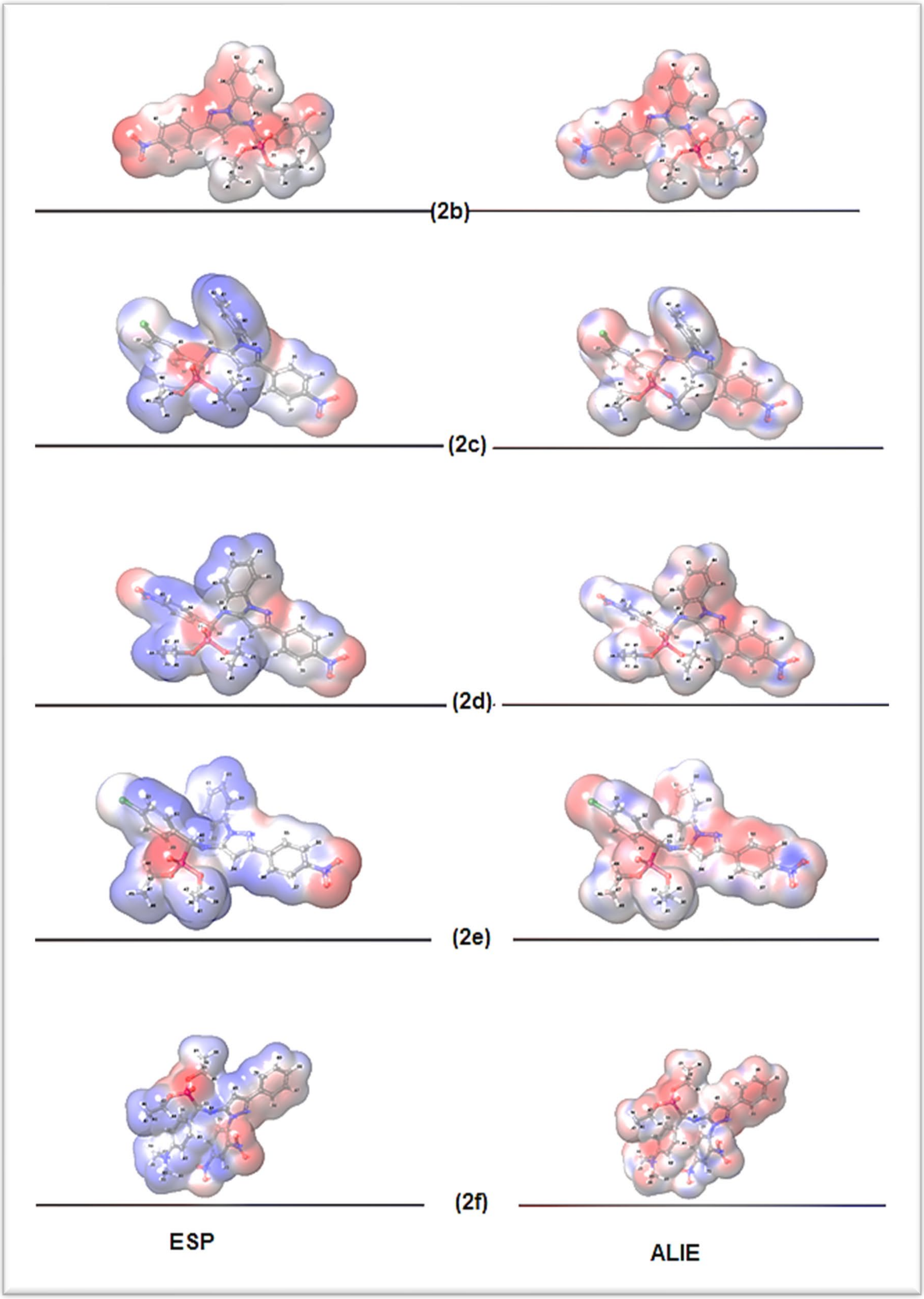

Fig. S36. Surface structure of ESP and ALIE using DFT method for α-aminophosphonates **2b-2f**.

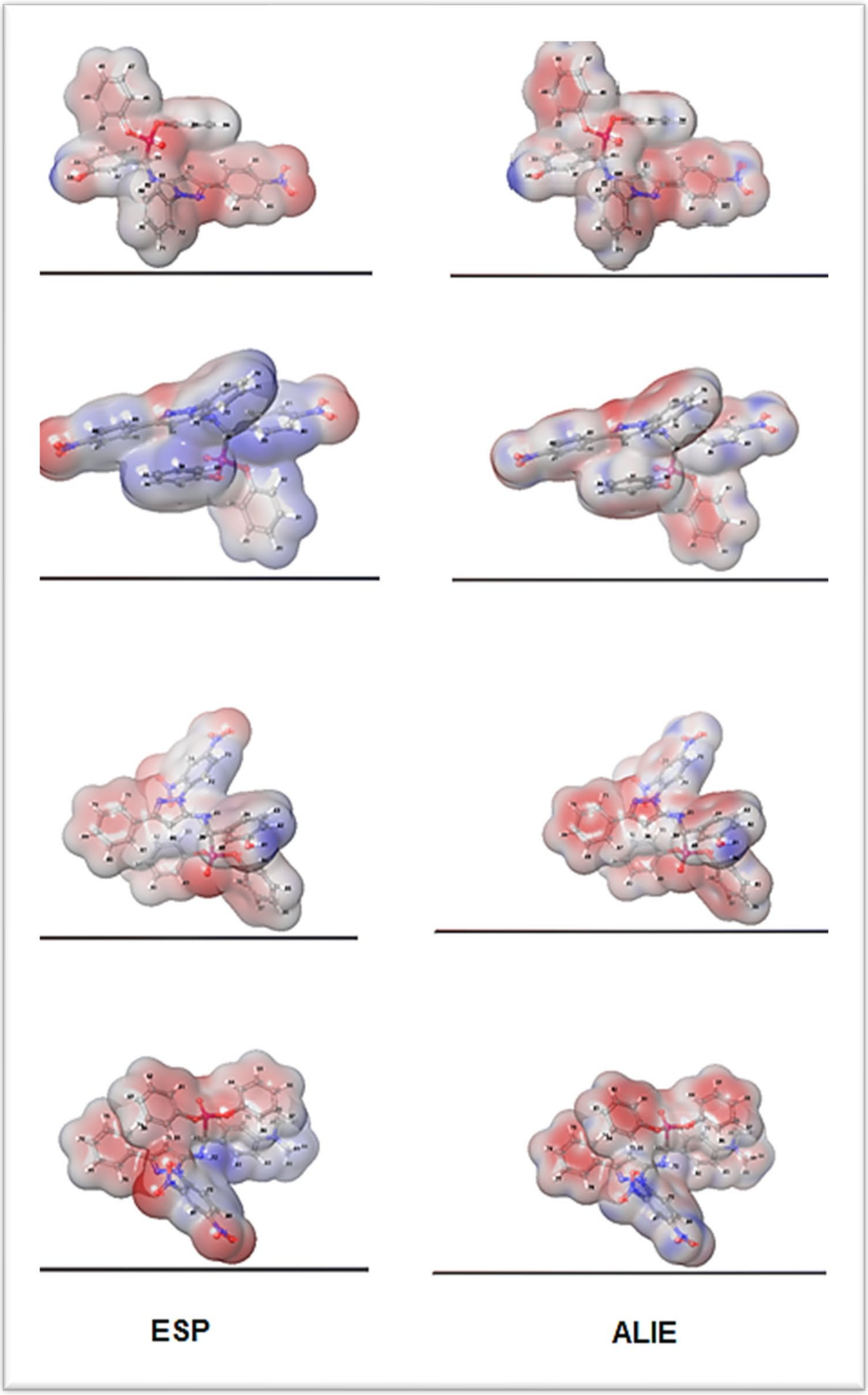

Fig. S37. Surface structure of ESP and ALIE using DFT method for α-aminophosphonates **4a-4d**.

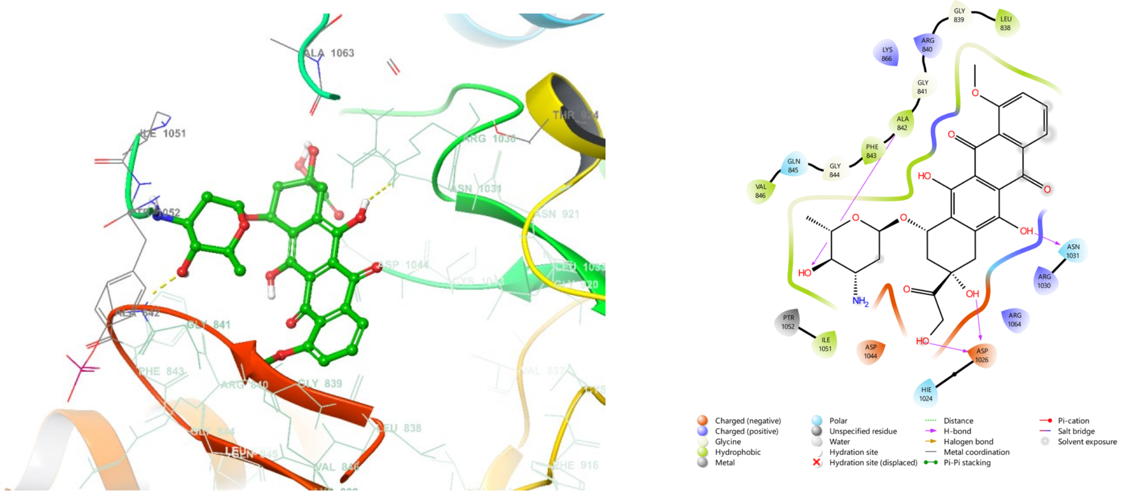

Fig. S38. 3D and 2D molecular interaction of **Doxorubicin** to inhibitory activity to the VEGFR-2 protein.

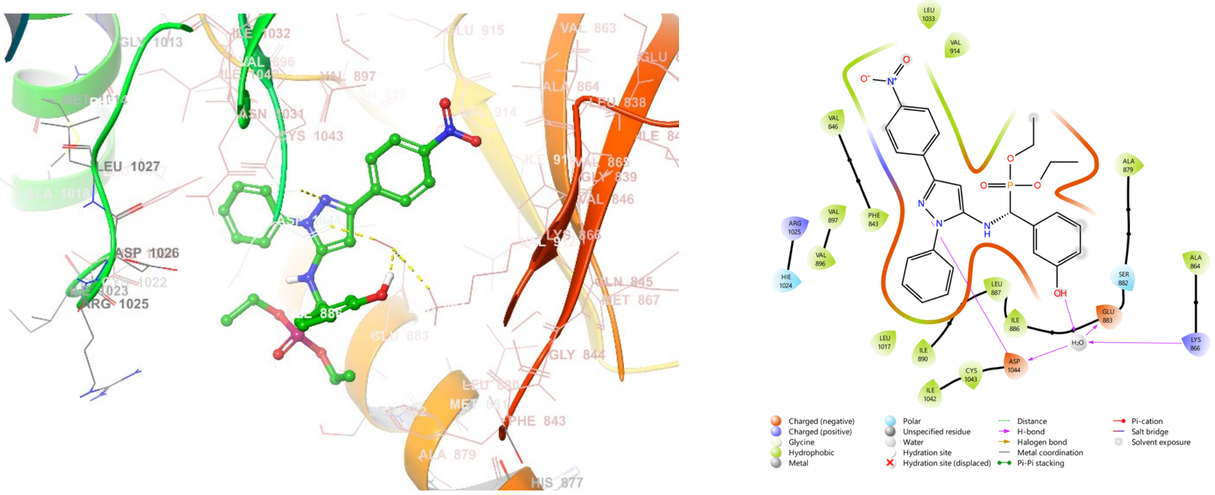

Fig. S39. 3D and 2D molecular interaction of **2b** to inhibitory activity to the VEGFR-2 protein.

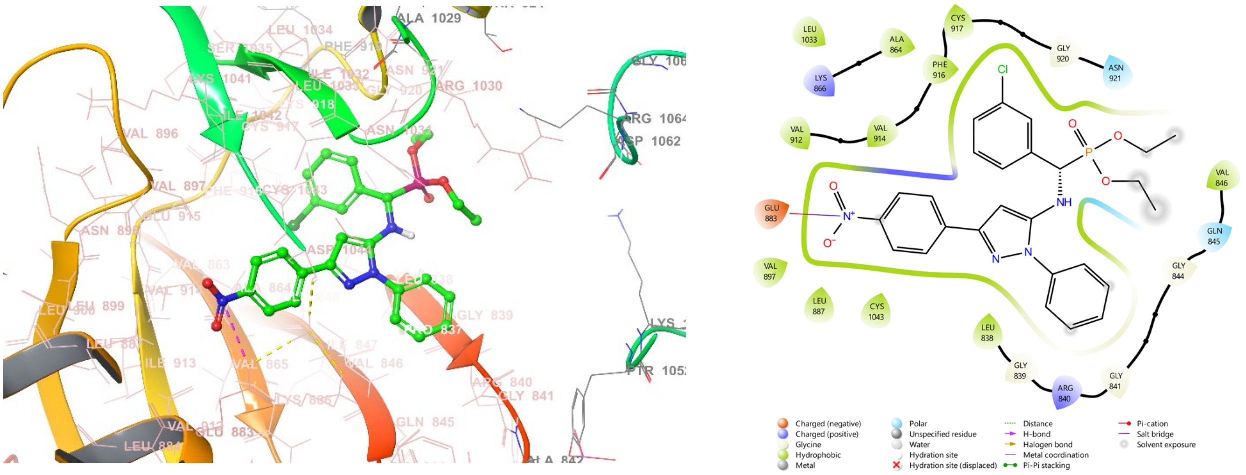

Fig. S40. 3D and 2D molecular interaction of **2c** to inhibitory activity to the VEGFR-2 protein.

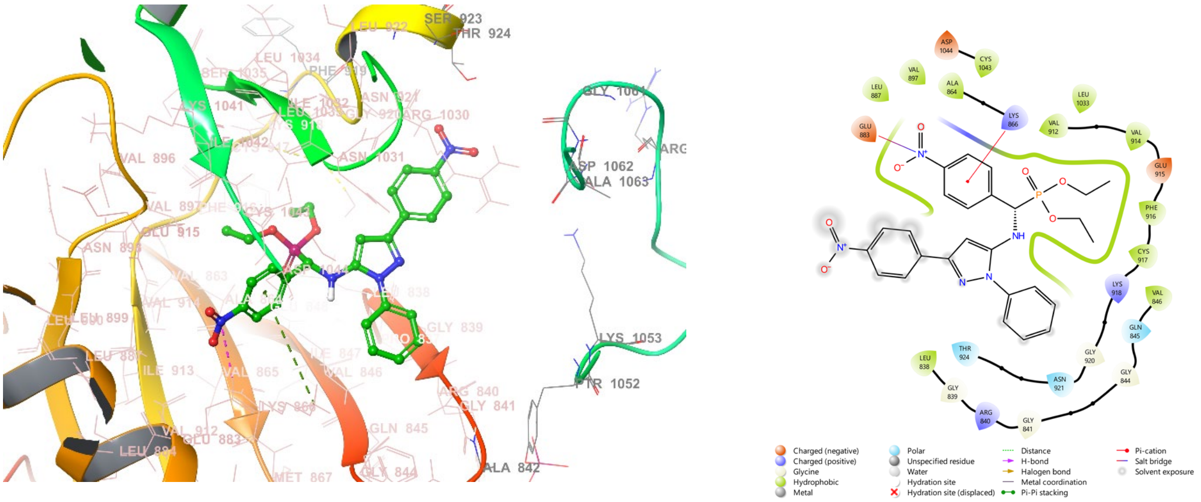

Fig. S41. 3D and 2D molecular interaction of **2d** to inhibitory activity to the VEGFR-2 protein.

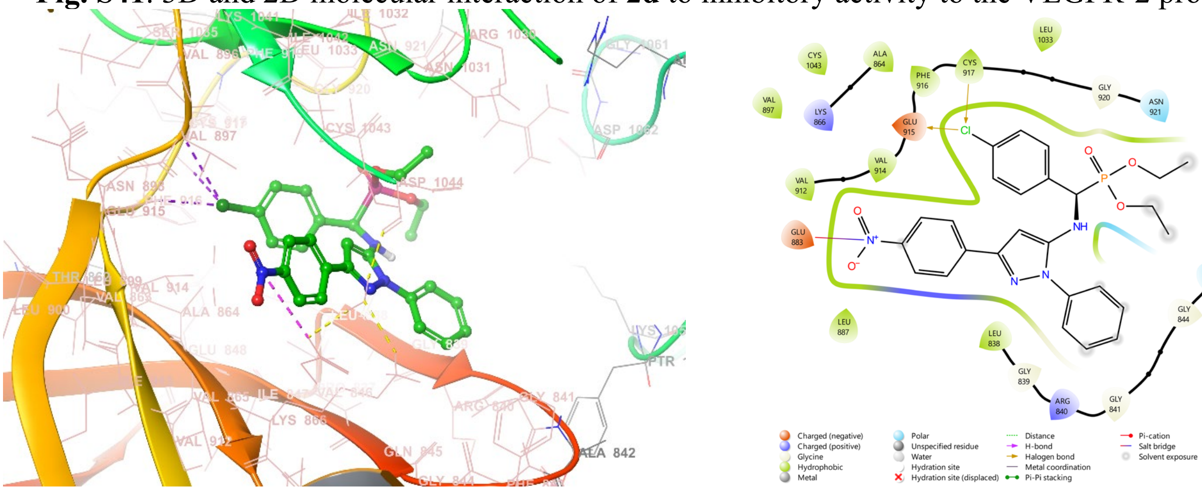

Fig. S42. 3D and 2D molecular interaction of **2e** to inhibitory activity to the VEGFR-2 protein.

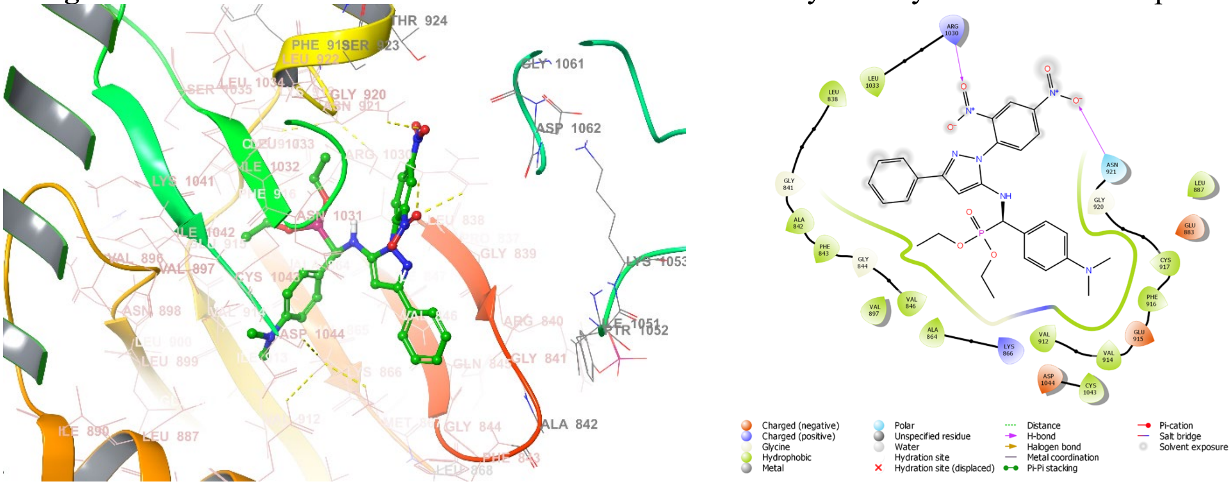

Fig. 43. 3D and 2D molecular interaction of **2f** to inhibitory activity to the VEGFR-2 protein.

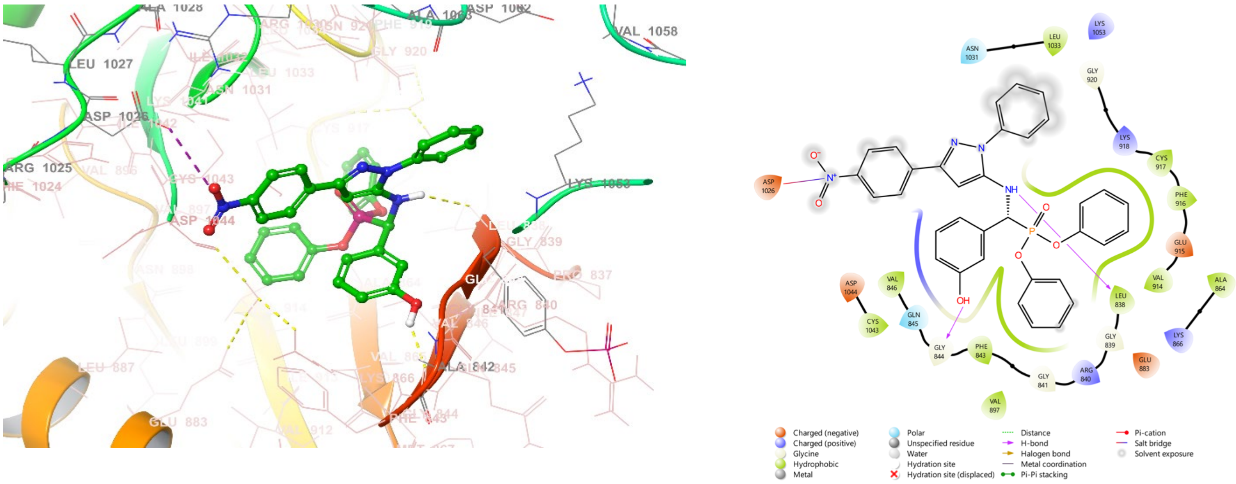

Fig. S44. 3D and 2D molecular interaction of **4a** to inhibitory activity to the VEGFR-2 protein.

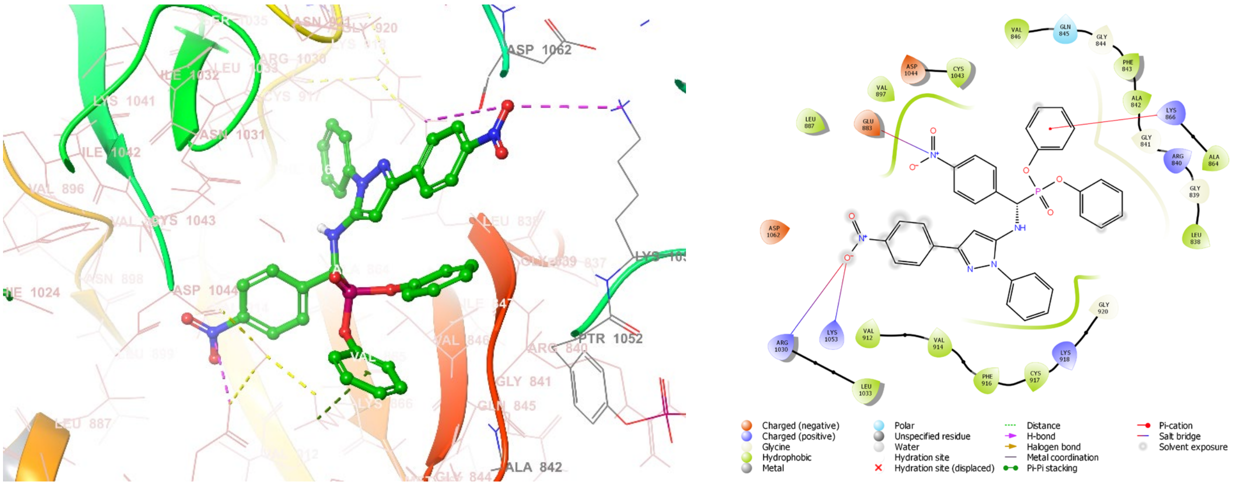

Fig. S45. 3D and 2D molecular interaction of **4b** to inhibitory activity to the VEGFR-2 protein

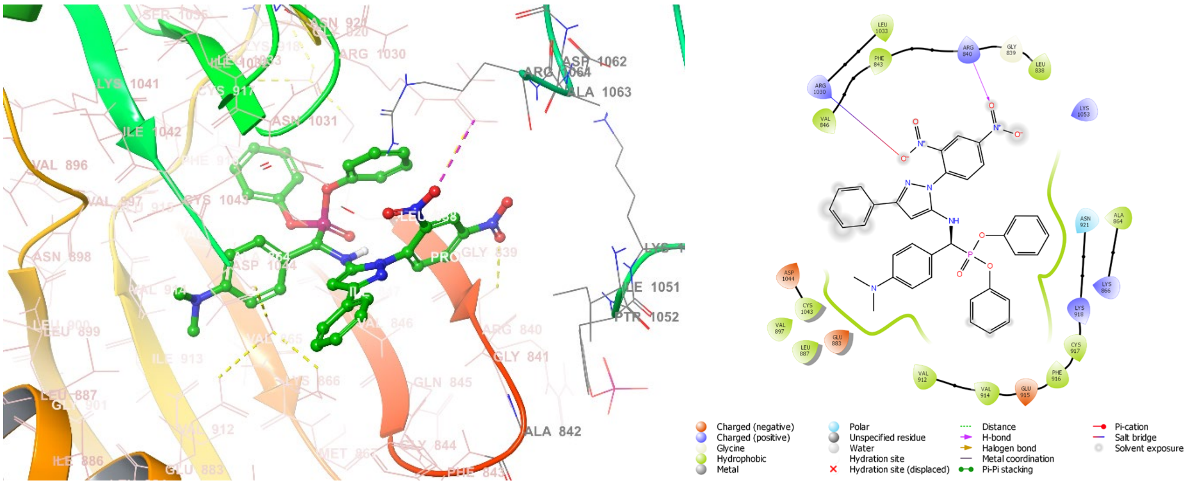

Fig. S46. 3D and 2D molecular interaction of **4d** to inhibitory activity to the VEGFR-2 protein.

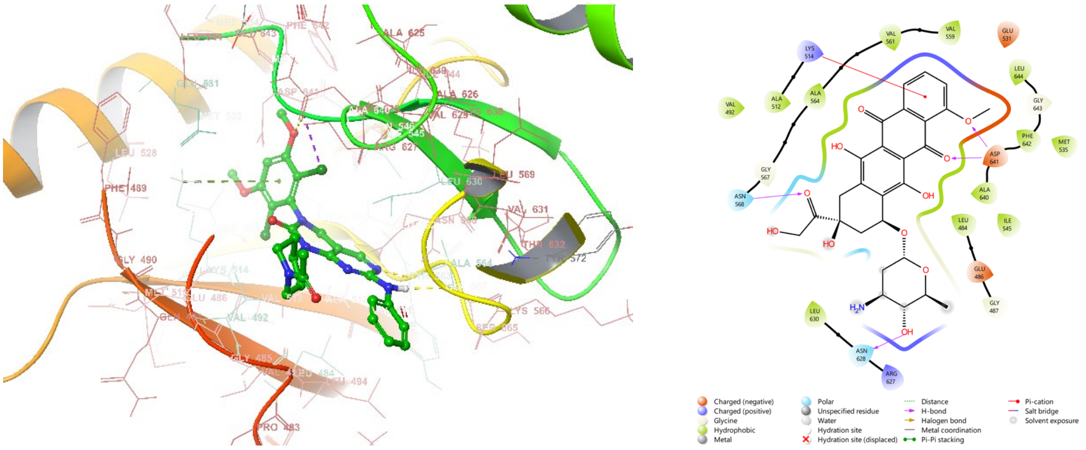

Fig. S47. 3D and 2D molecular interaction of **Doxorubicin** to inhibitory activity to the FGFR1 protein

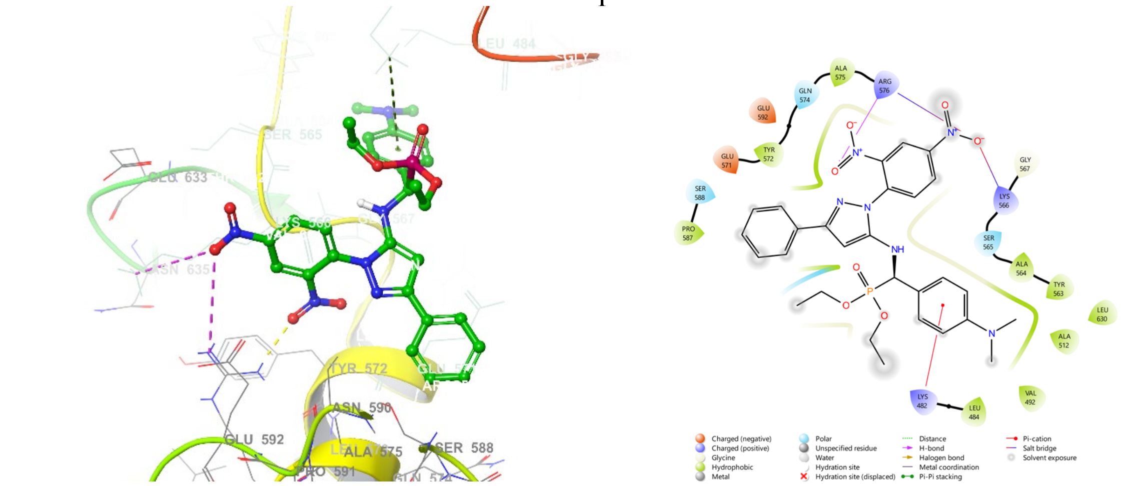

Fig. S48. 3D and 2D molecular interaction of **2f** to inhibitory activity to the FGFR1 protein

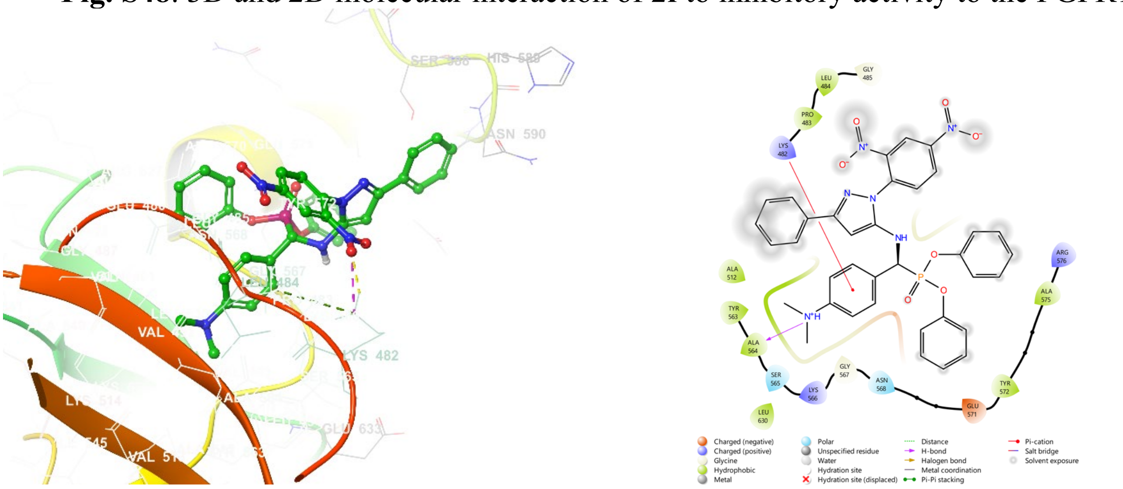

Fig. S49. 3D and 2D molecular interaction of **4d** to inhibitory activity to the FGFR1 protein

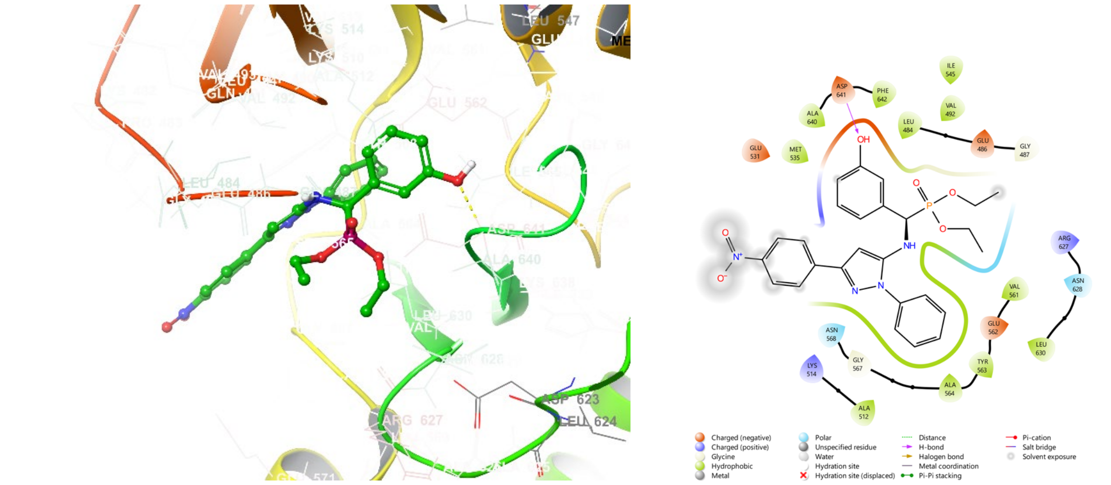

Fig. S50. 3D and 2D molecular interaction of **2b** to inhibitory activity to the FGFR1 protein

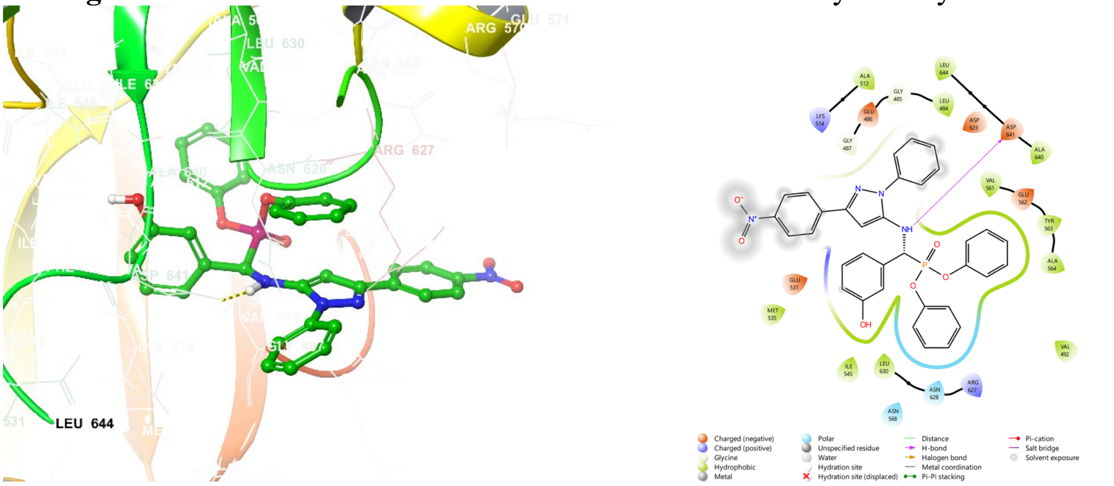

Fig. S51. 3D and 2D molecular interaction of **4a** to inhibitory activity to the FGFR1 protein

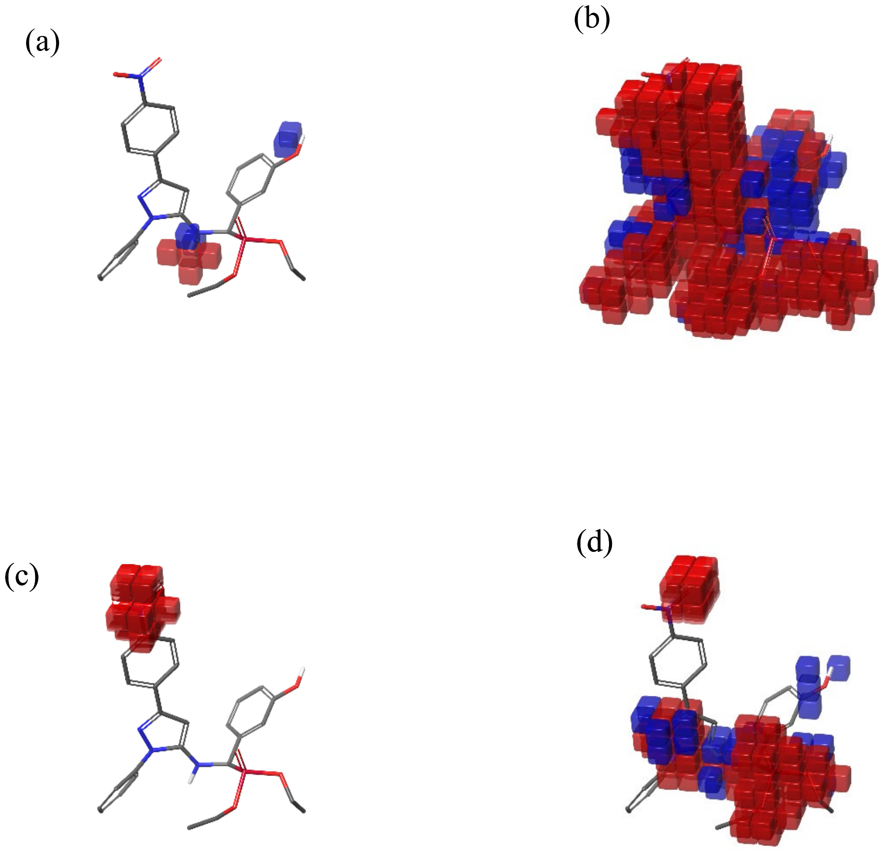

Fig. S52. QSAR models of compound 2b with VEGFR2 receptor inhibition for (a) hydrogen bond donor effect, (b) hydrophobic effect, (c) positive ionic effect, and (d) electron withdrawing effect.

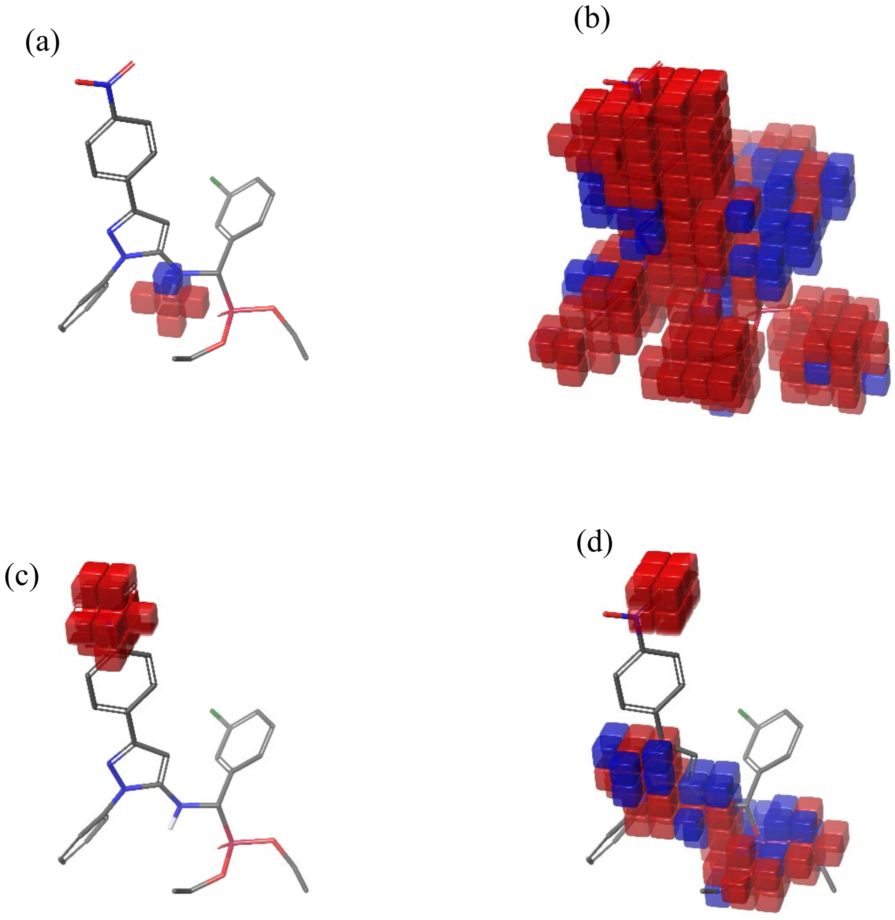

Fig. S53. QSAR models of compound 2c with VEGFR2 receptor inhibition for (a) hydrogen bond donor effect, (b) hydrophobic effect, (c) positive ionic effect, and (d) electron withdrawing effect.

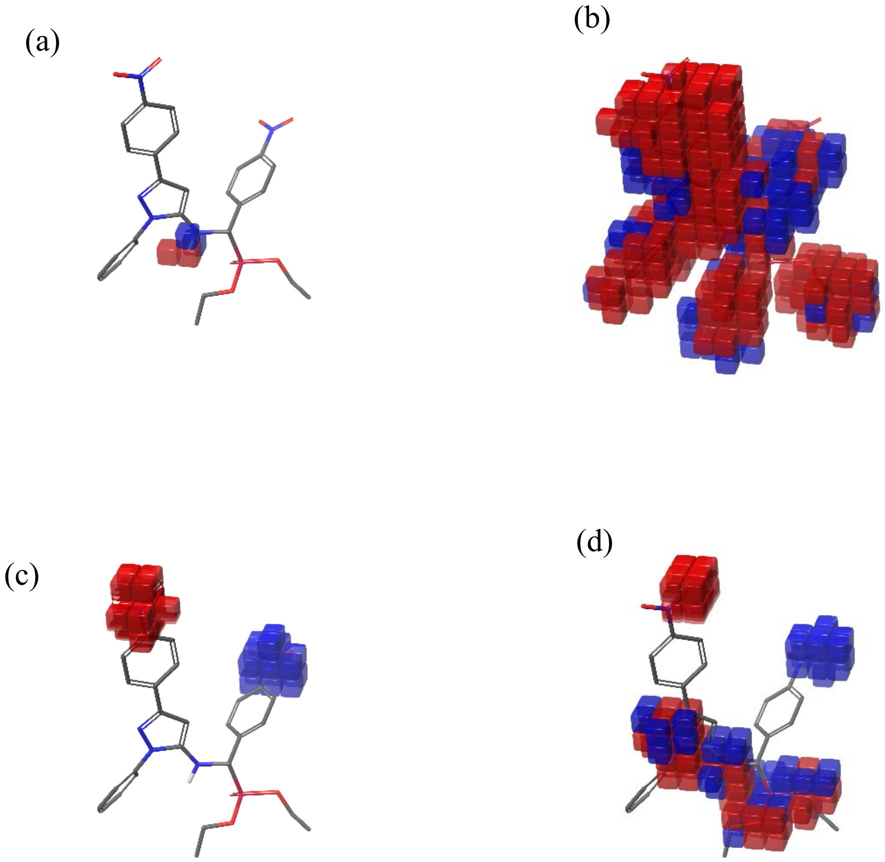

Fig. S54. QSAR models of compound 2d with VEGFR2 receptor inhibition for (a) hydrogen bond donor effect, (b) hydrophobic effect, (c) positive ionic effect, and (d) electron withdrawing effect.

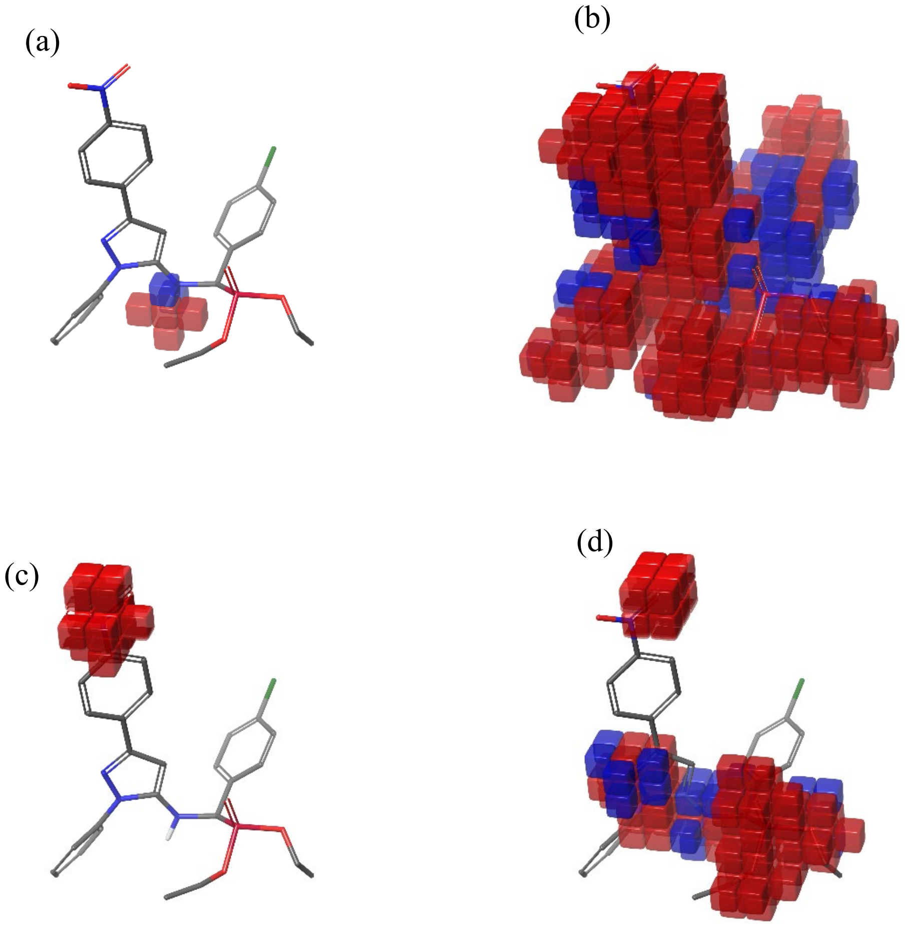

Fig. S55. QSAR models of compound 2e with VEGFR2 receptor inhibition for (a) hydrogen bond donor effect, (b) hydrophobic effect, (c) positive ionic effect, and (d) electron withdrawing effect.

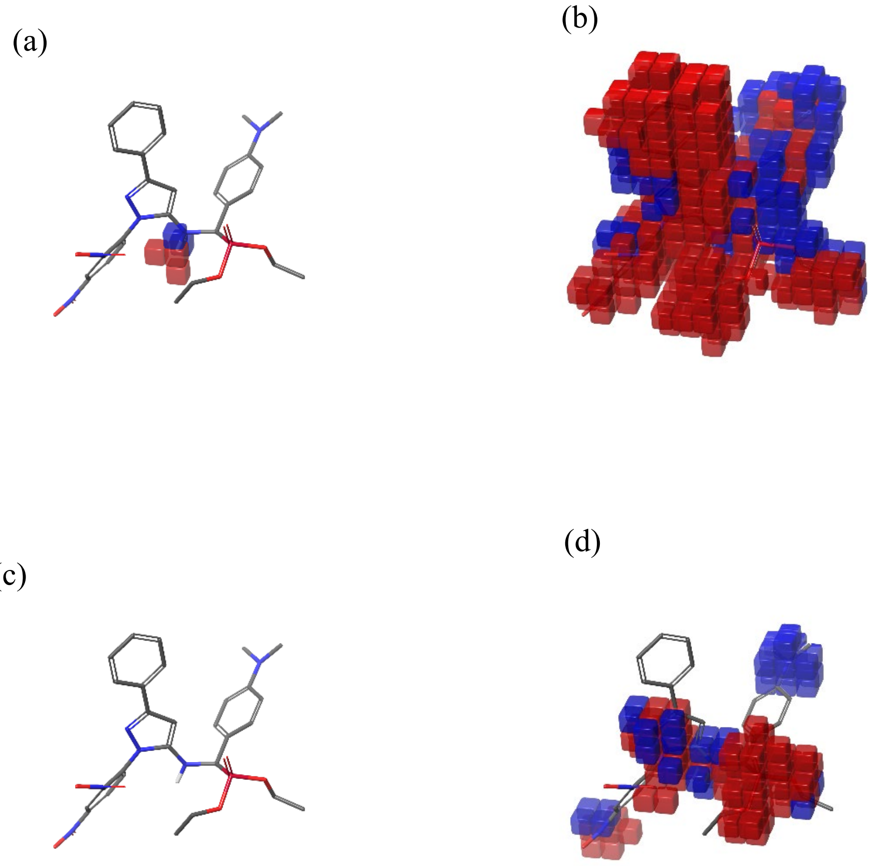

Fig. S56. QSAR models of compound 2f with VEGFR2 receptor inhibition for (a) hydrogen bond donor effect, (b) hydrophobic effect, (c) positive ionic effect, and (d) electron withdrawing effect.

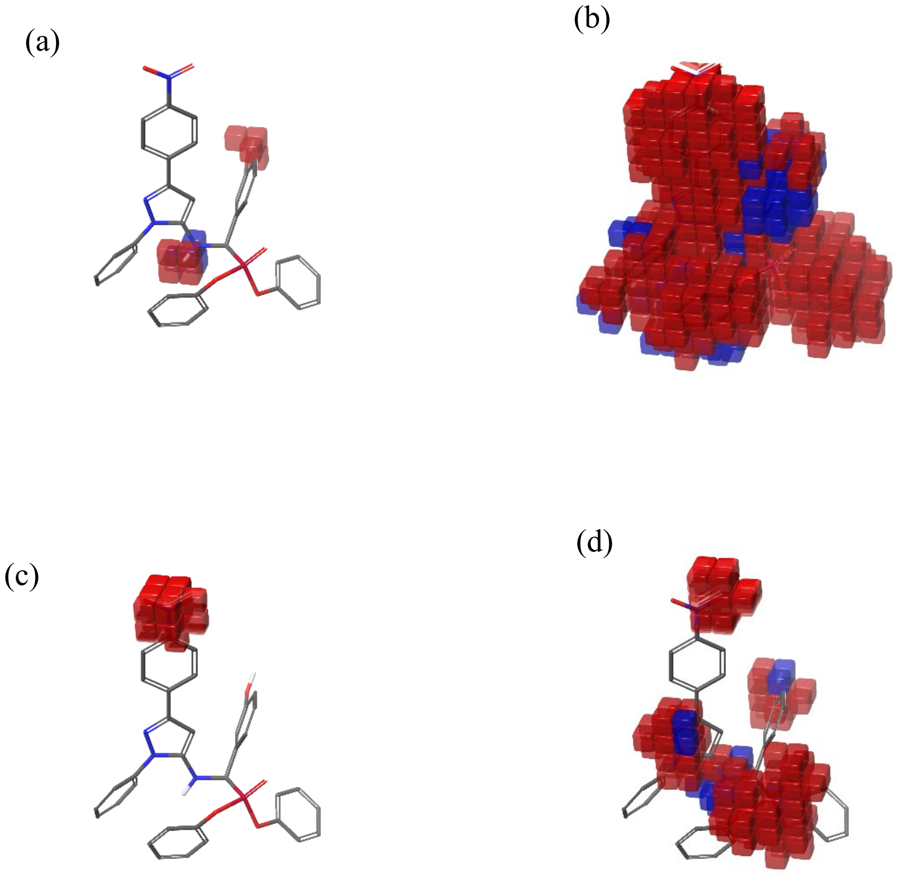

Fig. S57. QSAR models of compound 4a with VEGFR2 receptor inhibition for (a) hydrogen bond donor effect, (b) hydrophobic effect, (c) positive ionic effect, and (d) electron withdrawing effect.

(a) (b)

(c) (d)

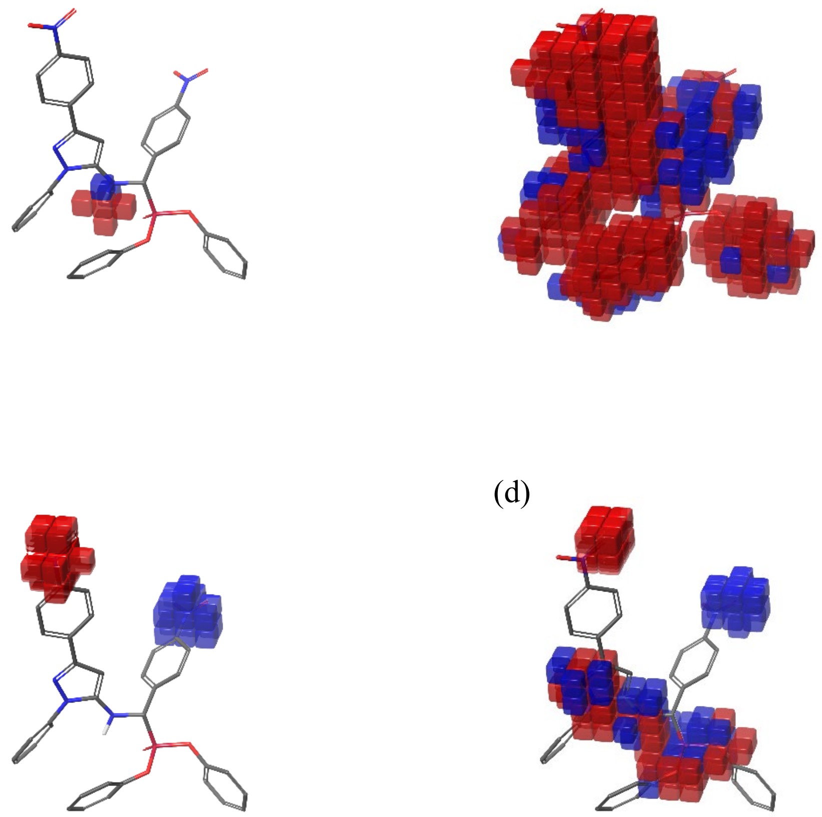

Fig. S58. QSAR models of compound 4b with VEGFR2 receptor inhibition for (a) hydrogen bond donor effect, (b) hydrophobic effect, (c) positive ionic effect, and (d) electron withdrawing effect.

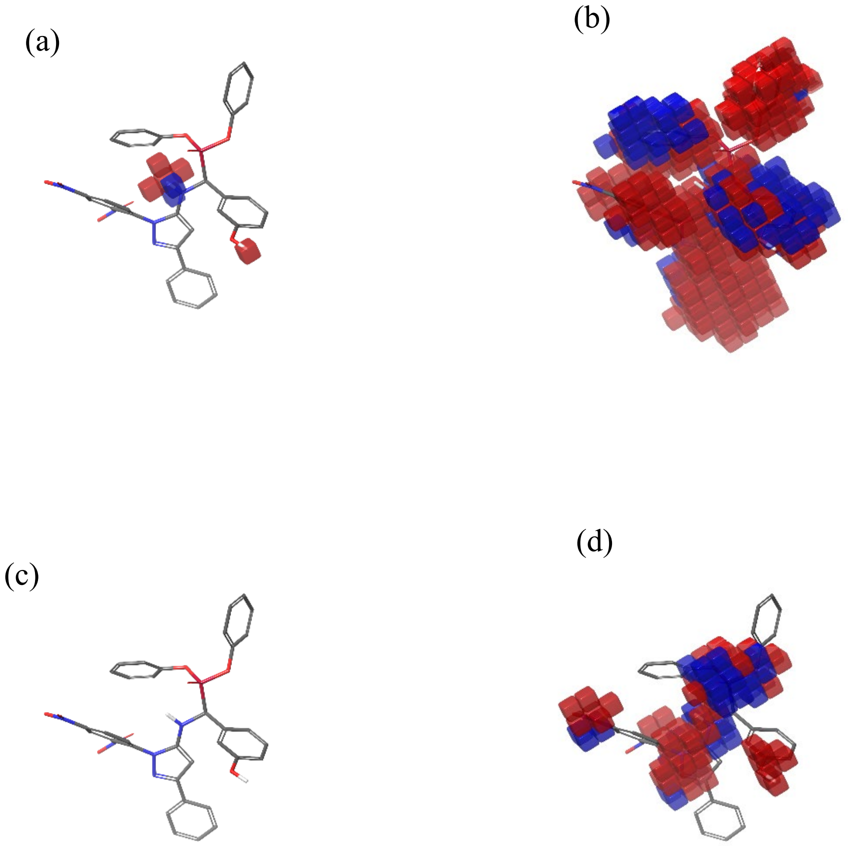

Fig. S59. QSAR models of compound 4c with VEGFR2 receptor inhibition for (a) hydrogen bond donor effect, (b) hydrophobic effect, (c) positive ionic effect, and (d) electron withdrawing effect.

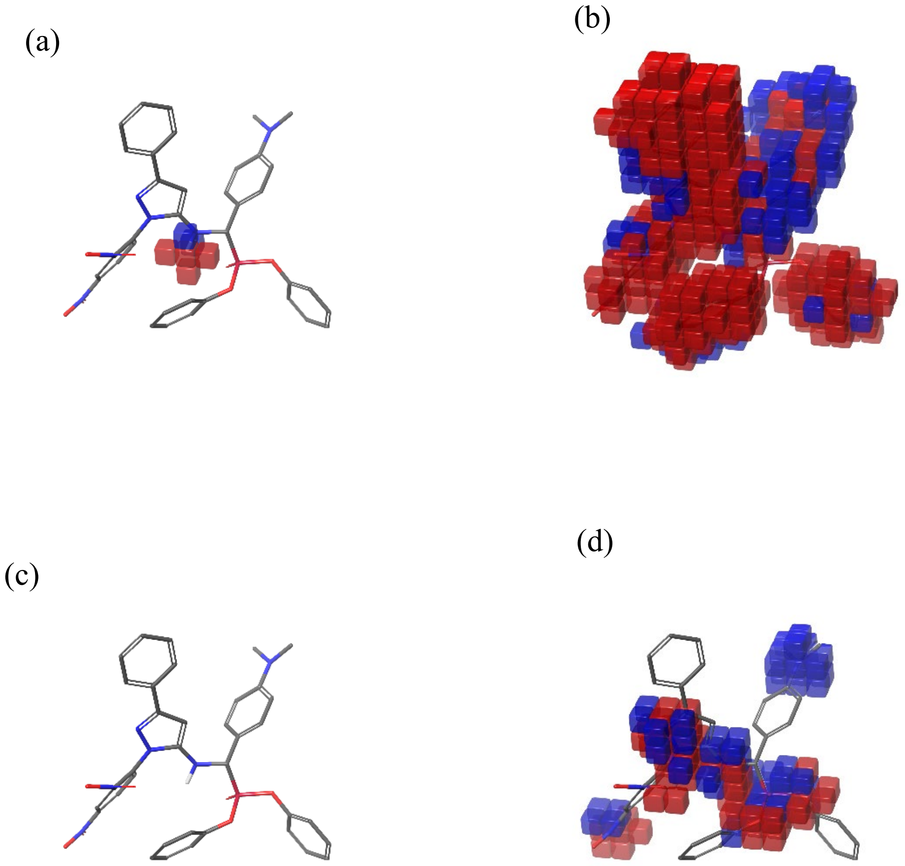

Fig. S60. QSAR models of compound 4d with VEGFR2 receptor inhibition for (a) hydrogen bond donor effect, (b) hydrophobic effect, (c) positive ionic effect, and (d) electron withdrawing effect.

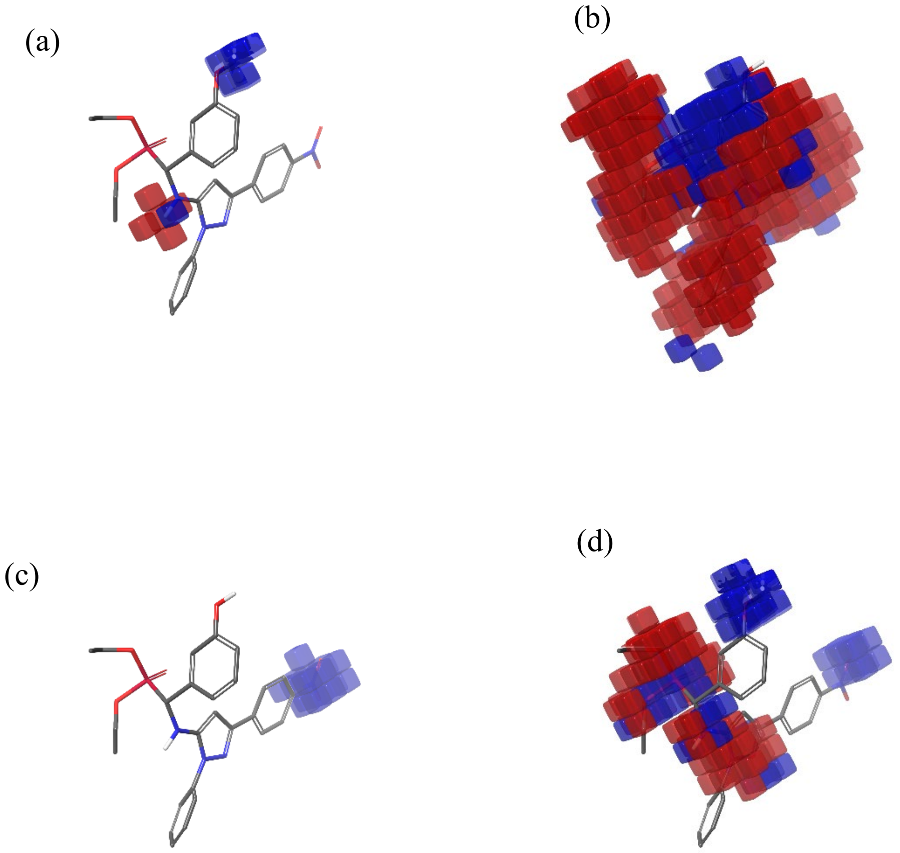

Fig. S61. QSAR models of compound 2b with FGFR1 receptor inhibition for (a) hydrogen bond donor effect, (b) hydrophobic effect, (c) positive ionic effect, and (d) electron withdrawing effect.

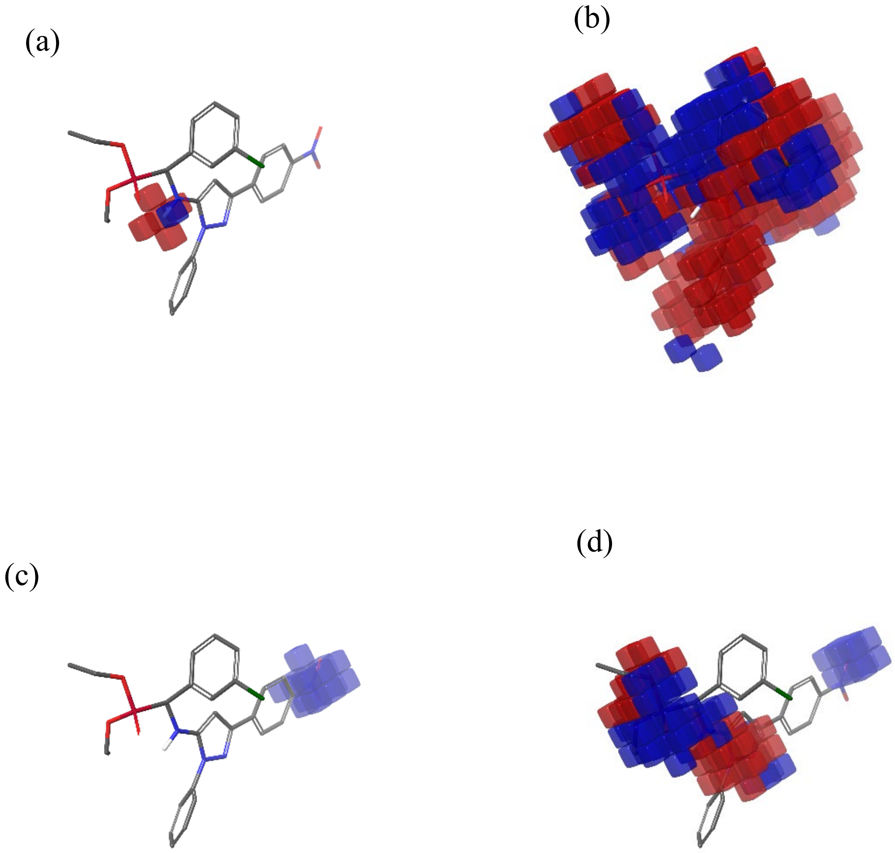

Fig. S62. QSAR models of compound 2c with FGFR1 receptor inhibition for (a) hydrogen bond donor effect, (b) hydrophobic effect, (c) positive ionic effect, and (d) electron withdrawing effect.

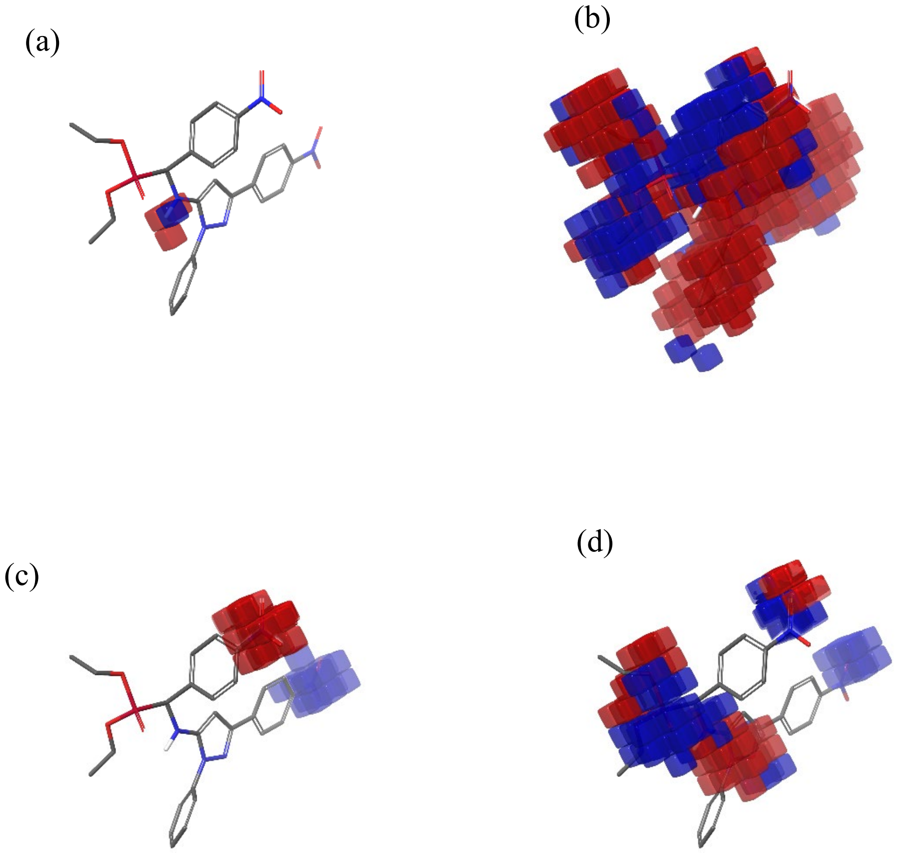

Fig. S63. QSAR models of compound 2d with FGFR1 receptor inhibition for (a) hydrogen bond donor effect, (b) hydrophobic effect, (c) positive ionic effect, and (d) electron withdrawing effect.

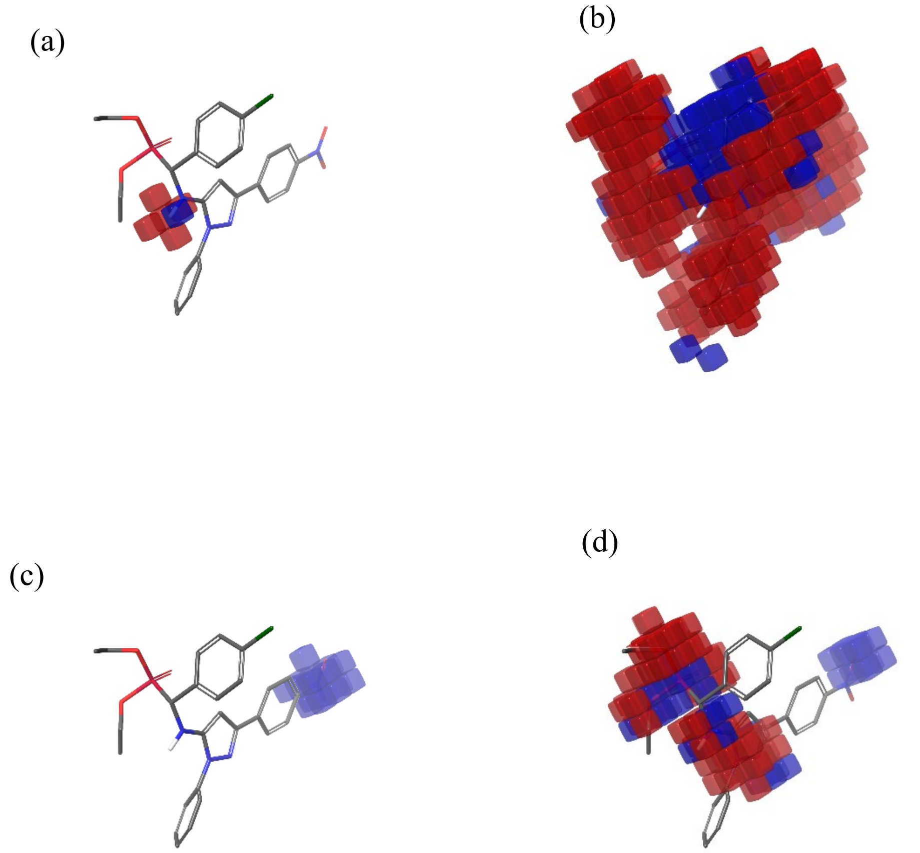

Fig. S64. QSAR models of compound 2e with FGFR1 receptor inhibition for (a) hydrogen bond donor effect, (b) hydrophobic effect, (c) positive ionic effect, and (d) electron withdrawing effect.

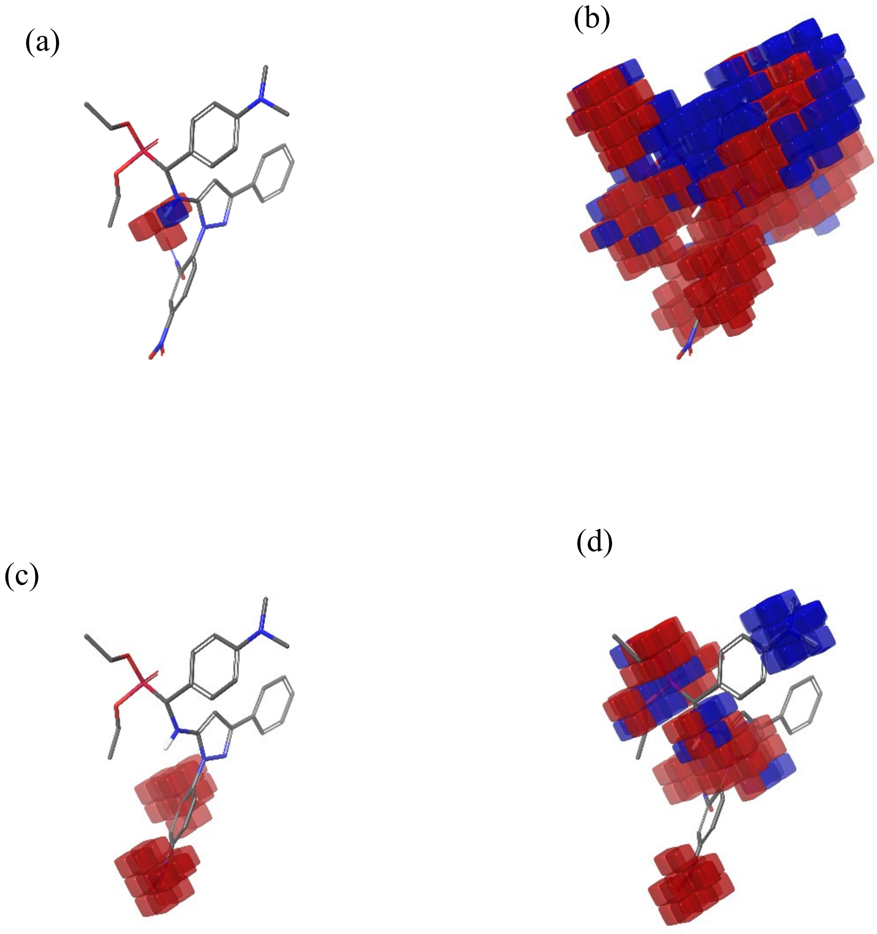

Fig. S65. QSAR models of compound 2f with FGFR1 receptor inhibition for (a) hydrogen bond donor effect, (b) hydrophobic effect, (c) positive ionic effect, and (d) electron withdrawing effect.

(a) (b)

(c) (d)

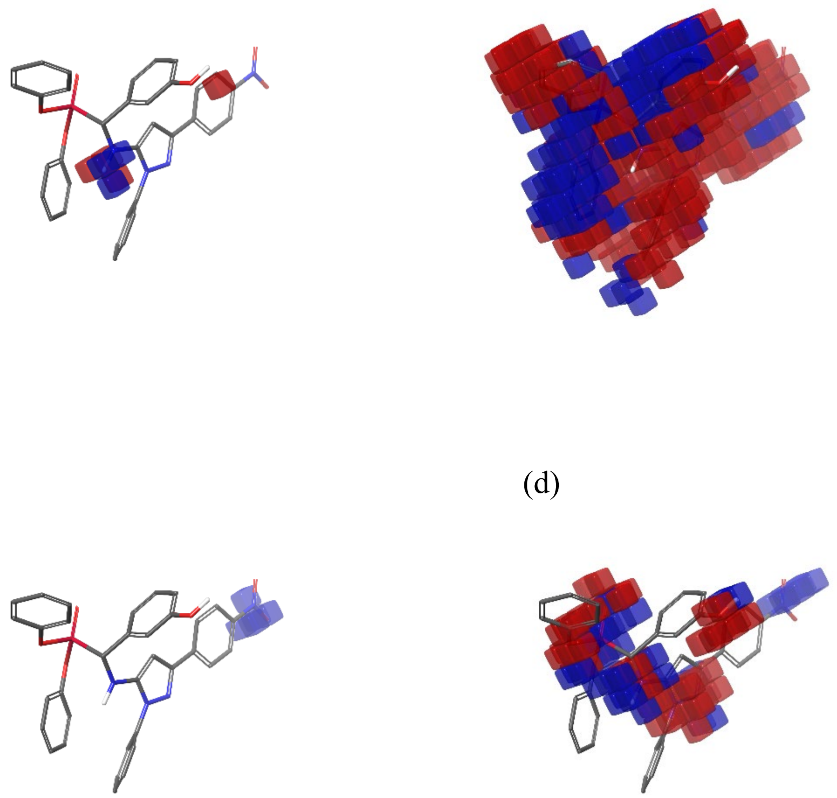

Fig. S66. QSAR models of compound 4a with FGFR1 receptor inhibition for (a) hydrogen bond donor effect, (b) hydrophobic effect, (c) positive ionic effect, and (d) electron withdrawing effect.

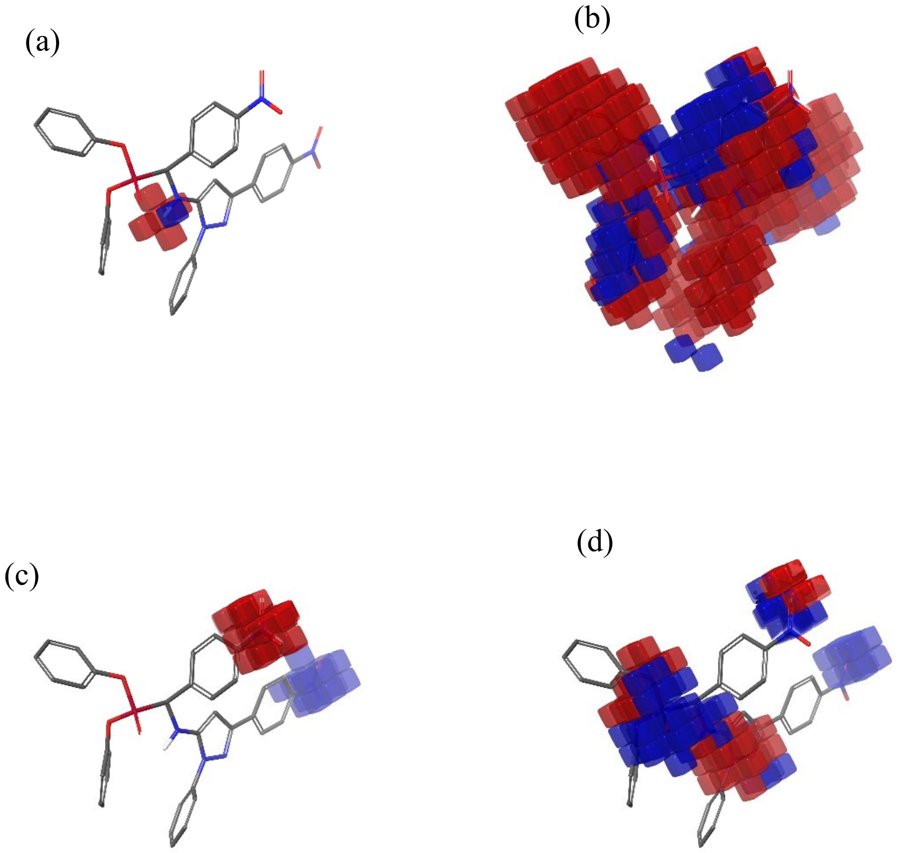

Fig. S67. QSAR models of compound 4b with FGFR1 receptor inhibition for (a) hydrogen bond donor effect, (b) hydrophobic effect, (c) positive ionic effect, and (d) electron withdrawing effect.

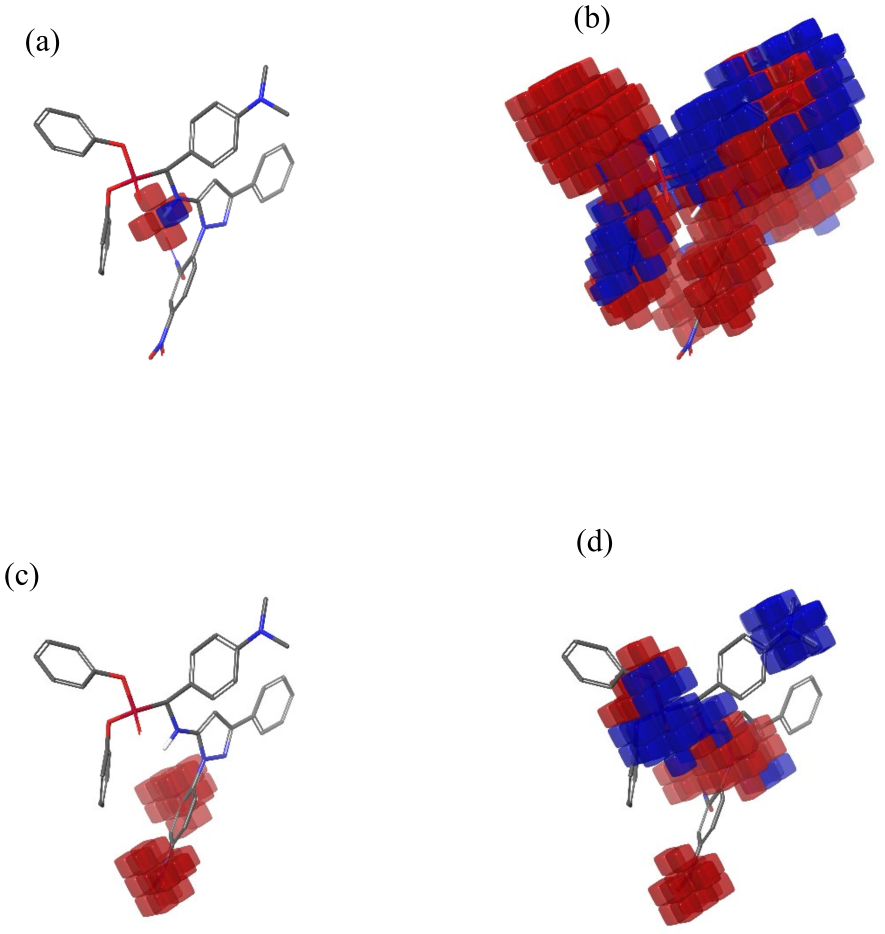

Fig. S68. QSAR models of compound 4d with FGFR1 receptor inhibition for (a) hydrogen bond donor effect, (b) hydrophobic effect, (c) positive ionic effect, and (d) electron withdrawing effect.

Table S1: Selected bond length (Å) of **2a**

Bond	Length(Å)	Bond	Length(Å)	Bond	Length(Å)
C(٣٨)-H(٦٥)	١,٠٨٤	C(٣٧)-H(٦٤)	١,٠٨٦	C(٣٧)-C(٣٨)	١,٣٩٣
C(٣٦)-H(٦٣)	١,٠٨٦	C(٣٦)-C(٣٧)	١,٣٩٧	C(٣٥)-H(٦٢)	١,٠٨٦
C(٣٥)-C(٣٦)	١,٣٩٥	C(٣٤)-H(٦١)	١,٠٨٧	C(٣٤)-C(٣٥)	١,٣٩٦
C(٣٣)-C(٣٤)	١,٣٩٨	C(٣٣)-C(٣٨)	١,٣٩٦	N(٣٢)-C(٣٣)	١,٤٢٤
N(٣١)-N(٣٢)	١,٣٦٢	C(٣٠)-H(٦٠)	١,٠٨٥	C(٢٩)-H(٥٩)	١,٠٨٣
C(٢٩)-C(٣٠)	١,٣٨٩	N(٢٦)-O(٢٨)	١,٢٣٤	N(٢٦)-O(٢٧)	١,٢٣٢
C(٢٥)-C(٢٩)	١,٣٩٤	C(٢٥)-N(٢٦)	١,٤٦٦	C(٢٤)-H(٥٨)	١,٠٨٢
C(٢٤)-C(٢٥)	١,٣٩٥	C(٢٣)-H(٥٧)	١,٠٨٣	C(٢٣)-C(٢٤)	١,٣٨٦
C(٢٢)-C(٢٣)	١,٤٠٨	C(٢٢)-C(٣٠)	١,٤٠٦	C(٢١)-N(٣١)	١,٣٣٦
C(٢١)-C(٢٢)	١,٤٦٧	C(٢٠)-H(٥٦)	١,٠٧٧	C(٢٠)-C(٢١)	١,٤٢٠
C(١٩)-C(٢٠)	١,٣٨٤	C(١٩)-N(٣٢)	١,٣٧٣	N(١٨)-H(٥٥)	١,٠١٤
N(١٨)-C(١٩)	١,٣٨٦	C(١٧)-H(٥٤)	١,٠٨٧	C(١٦)-H(٥٣)	١,٠٨٦
C(١٦)-C(١٧)	١,٣٩٥	O(١٥)-H(٥٢)	٠,٩٦٦	C(١٤)-C(١٦)	١,٣٩٨
C(١٤)-O(١٥)	١,٣٦٣	C(١٣)-H(٥١)	١,٠٨٦	C(١٣)-C(١٤)	١,٣٩٨
O(١٢)-H(٥٠)	٠,٩٦٥	C(١١)-C(١٣)	١,٣٩١	C(١١)-O(١٢)	١,٣٦٦
C(١٠)-C(١١)	١,٤٠٩	C(١٠)-C(١٧)	١,٣٩٤	C(٩)-H(٤٩)	١,٠٩٤
C(٩)-N(١٨)	١,٤٦٤	C(٩)-C(١٠)	١,٥١١	C(٨)-H(٤٨)	١,٠٩٥
C(٨)-H(٤٧)	١,٠٩٤	C(٨)-H(٤٦)	١,٠٩٣	C(٧)-H(٤٥)	١,٠٩٣
C(٧)-H(٤٤)	١,٠٩٣	C(٧)-C(٨)	١,٥١٨	O(٦)-C(٧)	١,٤٥٨
C(٥)-H(٤٣)	١,٠٩٣	C(٥)-H(٤٢)	١,٠٩٣	C(٥)-H(٤١)	١,٠٩٥
C(٤)-H(٤٠)	١,٠٩٥	C(٤)-H(٣٩)	١,٠٩٦	C(٤)-C(٥)	١,٥١٥
O(٣)-C(٤)	١,٤٤٩	P(٢)-C(٩)	١,٨٩٠	P(٢)-O(٦)	١,٦٢٥
P(٢)-O(٣)	١,٦١٤	O(١)-P(٢)	١,٤٨٥		

Table S2: Selected bond angle (°) of **2a**.

Angle	(°) Degree	Angle	(°) Degree	Angle	(°) Degree
H(٦٥)-C(٣٨)-C(٣٧)	١٢١,٥٢٣	H(٦٥)-C(٣٨)-C(٣٣)	١١٩,١٨٠	C(٣٧)-C(٣٨)-C(٣٣)	١١٩,٢٩٥
H(٦٤)-C(٣٧)-C(٣٨)	١١٩,٦٦٨	H(٦٤)-C(٣٧)-C(٣٦)	١٢٠,٠٩٧	C(٣٨)-C(٣٧)-C(٣٦)	١٢٠,٢٣٣
H(٦٣)-C(٣٦)-C(٣٧)	١١٩,٩٩٠	H(٦٣)-C(٣٦)-C(٣٥)	١٢٠,١٠٠	C(٣٧)-C(٣٦)-C(٣٥)	١١٩,٩٠٨
H(٦٢)-C(٣٥)-C(٣٦)	١٢٠,١٣٠	H(٦٢)-C(٣٥)-C(٣٤)	١١٩,٢٩٠	C(٣٦)-C(٣٥)-C(٣٤)	١٢٠,٥٦٢
H(٦١)-C(٣٤)-C(٣٥)	١٢١,٣٤٦	H(٦١)-C(٣٤)-C(٣٣)	١١٩,٨٢٧	C(٣٥)-C(٣٤)-C(٣٣)	١١٨,٨٠٧
C(٣٤)-C(٣٣)-C(٣٨)	١٢١,١٧١	C(٣٤)-C(٣٣)-N(٣٢)	١١٩,٥٩١	C(٣٨)-C(٣٣)-N(٣٢)	١١٩,٢٣٣
C(٣٣)-N(٣٢)-N(٣١)	١٢٠,١١٧	C(٣٣)-N(٣٢)-C(١٩)	١٢٧,٦٥٥	N(٣١)-N(٣٢)-C(١٩)	١١٢,٢١٧
N(٣٢)-N(٣١)-C(٢١)	١٠٤,٨٦٨	H(٦٠)-C(٣٠)-C(٢٩)	١١٩,٠٤٠	H(٦٠)-C(٣٠)-C(٢٢)	١١٩,٨٩٩
C(٢٩)-C(٣٠)-C(٢٢)	١٢١,٠٦٠	H(٥٩)-C(٢٩)-C(٣٠)	١٢١,٦٨٩	H(٥٩)-C(٢٩)-C(٢٥)	١١٩,٤٤٥
C(٣٠)-C(٢٩)-C(٢٥)	١١٨,٨٦٦	O(٢٨)-N(٢٦)-O(٢٧)	١٢٤,٣٤٢	O(٢٨)-N(٢٦)-C(٢٥)	١١٧,٨٠٢
O(٢٧)-N(٢٦)-C(٢٥)	١١٧,٨٥٦	C(٢٩)-C(٢٥)-N(٢٦)	١١٩,٢١٣	C(٢٩)-C(٢٥)-C(٢٤)	١٢١,٥١٨
N(٢٦)-C(٢٥)-C(٢٤)	١١٩,٢٦٨	H(٥٨)-C(٢٤)-C(٢٥)	١١٩,٤٠٧	H(٥٨)-C(٢٤)-C(٢٣)	١٢١,٥٧٠
C(٢٥)-C(٢٤)-C(٢٣)	١١٩,٠٢٣	H(٥٧)-C(٢٣)-C(٢٤)	١٢٠,٣٢٩	H(٥٧)-C(٢٣)-C(٢٢)	١١٨,٦٩٧
C(٢٤)-C(٢٣)-C(٢٢)	١٢٠,٩٧٤	C(٢٣)-C(٢٢)-C(٣٠)	١١٨,٥٥٩	C(٢٣)-C(٢٢)-C(٢١)	١٢٠,٣٢١
C(٣٠)-C(٢٢)-C(٢١)	١٢١,١١١	N(٣١)-C(٢١)-C(٢٢)	١٢٠,١٤٩	N(٣١)-C(٢١)-C(٢٠)	١١١,٥٧٠
C(٢٢)-C(٢١)-C(٢٠)	١٢٨,٢٥٠	H(٥٦)-C(٢٠)-C(٢١)	١٢٨,٤٩٦	H(٥٦)-C(٢٠)-C(١٩)	١٢٦,٥٤٤
C(٢١)-C(٢٠)-C(١٩)	١٠٤,٩٥٦	C(٢٠)-C(١٩)-N(٣٢)	١٠٦,٣٦٨	C(٢٠)-C(١٩)-N(١٨)	١٣٠,٩٠٦
N(٣٢)-C(١٩)-N(١٨)	١٢٢,٦٥٩	H(٥٥)-N(١٨)-C(١٩)	١١٣,١٩٢	H(٥٥)-N(١٨)-C(٩)	١١١,٣٦٧
C(١٩)-N(١٨)-C(٩)	١١٧,٤٩١	H(٥٤)-C(١٧)-C(١٦)	١١٨,٩٦٤	H(٥٤)-C(١٧)-C(١٠)	١١٨,٩٠٠
C(١٦)-C(١٧)-C(١٠)	١٢٢,١٣٦	H(٥٣)-C(١٦)-C(١٧)	١٢٠,٤٩٨	H(٥٣)-C(١٦)-C(١٤)	١٢٠,٣٣٠
C(١٧)-C(١٦)-C(١٤)	١١٩,١٧١	H(٥٢)-O(١٥)-C(١٤)	١٠٩,٤١٣	C(١٦)-C(١٤)-O(١٥)	١٢٣,١٦٨
C(١٦)-C(١٤)-C(١٣)	١٢٠,١٤٢	O(١٥)-C(١٤)-C(١٣)	١١٦,٦٨٩	H(٥١)-C(١٣)-C(١٤)	١١٩,١٩٣
H(٥١)-C(١٣)-C(١١)	١٢١,٢٧٥	C(١٤)-C(١٣)-C(١١)	١١٩,٥٣١	H(٥٠)-O(١٢)-C(١١)	١١٠,٨٩٧
C(١٣)-C(١١)-O(١٢)	١٢٢,١٦١	C(١٣)-C(١١)-C(١٠)	١٢١,٦١١	O(١٢)-C(١١)-C(١٠)	١١٦,٢٢٧
C(١١)-C(١٠)-C(١٧)	١١٧,٤٠٦	C(١١)-C(١٠)-C(٩)	١٢١,١٣٩	C(١٧)-C(١٠)-C(٩)	١٢١,٤٢٣
H(٤٩)-C(٩)-N(١٨)	١٠٨,٠٩٦	H(٤٩)-C(٩)-C(١٠)	١٠٨,٠٣٤	H(٤٩)-C(٩)-P(٢)	١٠٥,٢٠٧
N(١٨)-C(٩)-C(١٠)	١١١,٩١٦	N(١٨)-C(٩)-P(٢)	١١٠,٢٥٤	C(١٠)-C(٩)-P(٢)	١١٢,٩٧٣
H(٤٨)-C(٨)-H(٤٧)	١٠٨,٤٩٣	H(٤٨)-C(٨)-H(٤٦)	١٠٨,٣١٢	H(٤٨)-C(٨)-C(٧)	١٠٩,٨٧٣
H(٤٧)-C(٨)-H(٤٦)	١٠٩,٠٣٥	H(٤٧)-C(٨)-C(٧)	١١٠,٨١٢	H(٤٦)-C(٨)-C(٧)	١١٠,٢٥٧
H(٤٥)-C(٧)-H(٤٤)	١٠٨,٣٦٧	H(٤٥)-C(٧)-C(٨)	١١١,٤٩٤	H(٤٥)-C(٧)-O(٦)	١٠٨,٩٧٣
H(٤٤)-C(٧)-C(٨)	١١١,٨٩٣	H(٤٤)-C(٧)-O(٦)	١٠٤,٧٠٧	C(٨)-C(٧)-O(٦)	١١١,١٣٤
C(٧)-O(٦)-P(٢)	١١٩,٤١١	H(٤٣)-C(٥)-H(٤٢)	١٠٨,٧٥٨	H(٤٣)-C(٥)-H(٤١)	١٠٨,٧٩٥
H(٤٣)-C(٥)-C(٤)	١١٠,٠٤٥	H(٤٢)-C(٥)-H(٤١)	١٠٨,٦١٤	H(٤٢)-C(٥)-C(٤)	١١٠,٣٩٩
H(٤١)-C(٥)-C(٤)	١١٠,١٨٦	H(٤٠)-C(٤)-H(٣٩)	١٠٧,٨٧٤	H(٤٠)-C(٤)-C(٥)	١١٢,٣٥١
H(٤٠)-C(٤)-O(٣)	١٠٨,١٨٧	H(٣٩)-C(٤)-C(٥)	١١٠,٦٣٧	H(٣٩)-C(٤)-O(٣)	١١٠,٣١٨
C(٥)-C(٤)-O(٣)	١٠٧,٤٥٣	C(٤)-O(٣)-P(٢)	١٢١,٧٧٣	C(٩)-P(٢)-O(٦)	١٠٢,١٧٤
C(٩)-P(٢)-O(٣)	١٠٦,٣١٦	C(٩)-P(٢)-O(١)	١١٤,٩٧٩	O(٦)-P(٢)-O(٣)	١٠٤,١٨٥
O(٦)-P(٢)-O(١)	١١٥,٠٣٣	O(٣)-P(٢)-O(١)	١١٢,٩٤٠		

Table S3: Selected bond length (Å) of **2b**.

Bond	Length(Å)	Bond	Length(Å)	Bond	Length(Å)
C(٣٧)-H(٦٤)	١,٠٨٤	C(٣٦)-H(٦٣)	١,٠٨٦	C(٣٦)-C(٣٧)	١,٣٩٢
C(٣٥)-H(٦٢)	١,٠٨٧	C(٣٥)-C(٣٦)	١,٣٩٨	C(٣٤)-H(٦١)	١,٠٨٦
C(٣٤)-C(٣٥)	١,٣٩٦	C(٣٣)-H(٦٠)	١,٠٨٥	C(٣٣)-C(٣٤)	١,٣٩٦
C(٣٢)-C(٣٣)	١,٣٩٨	C(٣٢)-C(٣٧)	١,٣٩٦	N(٣١)-C(٣٢)	١,٤٢٣
N(٣٠)-N(٣١)	١,٣٦٠	C(٢٩)-H(٥٩)	١,٠٨٥	C(٢٨)-H(٥٨)	١,٠٨٣
C(٢٨)-C(٢٩)	١,٣٩٠	N(٢٥)-O(٢٧)	١,٢٣٢	N(٢٥)-O(٢٦)	١,٢٣٣
C(٢٤)-C(٢٨)	١,٣٩٤	C(٢٤)-N(٢٥)	١,٤٦٦	C(٢٣)-H(٥٧)	١,٠٨٣
C(٢٣)-C(٢٤)	١,٣٩٥	C(٢٢)-H(٥٦)	١,٠٨٣	C(٢٢)-C(٢٣)	١,٣٨٦
C(٢١)-C(٢٢)	١,٤٠٨	C(٢١)-C(٢٩)	١,٤٠٧	C(٢٠)-N(٣٠)	١,٣٣٦
C(٢٠)-C(٢١)	١,٤٦٧	C(١٩)-H(٥٥)	١,٠٧٩	C(١٩)-C(٢٠)	١,٤٢١
C(١٨)-C(١٩)	١,٣٨٤	C(١٨)-N(٣١)	١,٣٧٥	N(١٧)-H(٥٤)	١,٠١٢
N(١٧)-C(١٨)	١,٣٨٥	C(١٦)-H(٥٣)	١,٠٨٦	C(١٥)-H(٥٢)	١,٠٨٦
C(١٥)-C(١٦)	١,٣٩٤	C(١٤)-H(٥١)	١,٠٨٨	C(١٤)-C(١٥)	١,٣٩٤
O(١٣)-H(٥٠)	٠,٩٦٦	C(١٢)-C(١٤)	١,٣٩٩	C(١٢)-O(١٣)	١,٣٦٥
C(١١)-H(٤٩)	١,٠٨٥	C(١١)-C(١٢)	١,٣٩٧	C(١٠)-C(١١)	١,٣٩٨
C(١٠)-C(١٦)	١,٣٩٩	C(٩)-H(٤٨)	١,٠٩٤	C(٩)-N(١٧)	١,٤٦٥
C(٩)-C(١٠)	١,٥١٦	C(٨)-H(٤٧)	١,٠٩٣	C(٨)-H(٤٦)	١,٠٩٥
C(٨)-H(٤٥)	١,٠٩٣	C(٧)-H(٤٤)	١,٠٩٢	C(٧)-H(٤٣)	١,٠٩٦
C(٧)-C(٨)	١,٥١٧	O(٦)-C(٧)	١,٤٥٠	C(٥)-H(٤٢)	١,٠٩٥
C(٥)-H(٤١)	١,٠٩٤	C(٥)-H(٤٠)	١,٠٩٢	C(٤)-H(٣٩)	١,٠٩٤
C(٤)-H(٣٨)	١,٠٩٣	C(٤)-C(٥)	١,٥١٩	O(٣)-C(٤)	١,٤٥٨
P(٢)-C(٩)	١,٨٦٢	P(٢)-O(٦)	١,٦١١	P(٢)-O(٣)	١,٦١٧
O(١)-P(٢)	١,٤٨٧				

Table S4: Selected bond angle (°) of **2b**.

Angle	(°) Degree	Angle	(°) Degree	Angle	(°) Degree
H(٦٤)-C(٣٧)-C(٣٦)	١٢١,٤٠٣	H(٦٤)-C(٣٧)-C(٣٢)	١١٩,١٥٣	C(٣٦)-C(٣٧)-C(٣٢)	١١٩,٤٤٣
H(٦٣)-C(٣٦)-C(٣٧)	١١٩,٦٨٣	H(٦٣)-C(٣٦)-C(٣٥)	١٢٠,٠٦٧	C(٣٧)-C(٣٦)-C(٣٥)	١٢٠,٢٤٥
H(٦٢)-C(٣٥)-C(٣٦)	١٢٠,٠٤٢	H(٦٢)-C(٣٥)-C(٣٤)	١٢٠,١٤٢	C(٣٦)-C(٣٥)-C(٣٤)	١١٩,٨١٦
H(٦١)-C(٣٤)-C(٣٥)	١٢٠,٣٣١	H(٦١)-C(٣٤)-C(٣٣)	١١٩,١٦٦	C(٣٥)-C(٣٤)-C(٣٣)	١٢٠,٤٩٦
H(٦٠)-C(٣٣)-C(٣٤)	١١٩,٧٧٩	H(٦٠)-C(٣٣)-C(٣٢)	١٢١,١٥٩	C(٣٤)-C(٣٣)-C(٣٢)	١١٩,٠٥٦
C(٣٣)-C(٣٢)-C(٣٧)	١٢٠,٩١٤	C(٣٣)-C(٣٢)-N(٣١)	١٢٠,٢٢٩	C(٣٧)-C(٣٢)-N(٣١)	١١٨,٨٥٧
C(٣٢)-N(٣١)-N(٣٠)	١٢٠,٢٥٧	C(٣٢)-N(٣١)-C(١٨)	١٢٧,٥١٦	N(٣٠)-N(٣١)-C(١٨)	١١٢,١٤٢
N(٣١)-N(٣٠)-C(٢٠)	١٠٤,٩٥٧	H(٥٩)-C(٢٩)-C(٢٨)	١١٩,١٤٠	H(٥٩)-C(٢٩)-C(٢١)	١١٩,٨٧٥
C(٢٨)-C(٢٩)-C(٢١)	١٢٠,٩٨٤	H(٥٨)-C(٢٨)-C(٢٩)	١٢١,٦٨٣	H(٥٨)-C(٢٨)-C(٢٤)	١١٩,٤٦٤
C(٢٩)-C(٢٨)-C(٢٤)	١١٨,٨٥٢	O(٢٧)-N(٢٥)-O(٢٦)	١٢٤,٣١٩	O(٢٧)-N(٢٥)-C(٢٤)	١١٧,٨٩٨
O(٢٦)-N(٢٥)-C(٢٤)	١١٧,٧٨٣	C(٢٨)-C(٢٤)-N(٢٥)	١١٩,١٢٣	C(٢٨)-C(٢٤)-C(٢٣)	١٢١,٦٠٥
N(٢٥)-C(٢٤)-C(٢٣)	١١٩,٢٧٠	H(٥٧)-C(٢٣)-C(٢٤)	١١٩,٤٣٦	H(٥٧)-C(٢٣)-C(٢٢)	١٢١,٥٨٨
C(٢٤)-C(٢٣)-C(٢٢)	١١٨,٩٧٦	H(٥٦)-C(٢٢)-C(٢٣)	١٢٠,٣٧٧	H(٥٦)-C(٢٢)-C(٢١)	١١٨,٦٨٢
C(٢٣)-C(٢٢)-C(٢١)	١٢٠,٩٤١	C(٢٢)-C(٢١)-C(٢٩)	١١٨,٦٤١	C(٢٢)-C(٢١)-C(٢٠)	١٢٠,١٨٦
C(٢٩)-C(٢١)-C(٢٠)	١٢١,١٦٩	N(٣٠)-C(٢٠)-C(٢١)	١١٩,٩٨٧	N(٣٠)-C(٢٠)-C(١٩)	١١١,٥٦٥
C(٢١)-C(٢٠)-C(١٩)	١٢٨,٤٤٣	H(٥٥)-C(١٩)-C(٢٠)	١٢٨,٤٨٧	H(٥٥)-C(١٩)-C(١٨)	١٢٦,٥٨٧
C(٢٠)-C(١٩)-C(١٨)	١٠٤,٨٧٩	C(١٩)-C(١٨)-N(٣١)	١٠٦,٤٢٣	C(١٩)-C(١٨)-N(١٧)	١٣٠,٩١٢
N(٣١)-C(١٨)-N(١٧)	١٢٢,٥٥٤	H(٥٤)-N(١٧)-C(١٨)	١١٣,٨٣٣	H(٥٤)-N(١٧)-C(٩)	١١٣,١٥١
C(١٨)-N(١٧)-C(٩)	١٢٠,١٦٩	H(٥٣)-C(١٦)-C(١٥)	١٢٠,١٦٠	H(٥٣)-C(١٦)-C(١٠)	١١٩,٩٩٢
C(١٥)-C(١٦)-C(١٠)	١١٩,٨٤٧	H(٥٢)-C(١٥)-C(١٦)	١١٩,٩٢٠	H(٥٢)-C(١٥)-C(١٤)	١١٩,٤٣٨
C(١٦)-C(١٥)-C(١٤)	١٢٠,٦٤١	H(٥١)-C(١٤)-C(١٥)	١٢٠,٥٣٠	H(٥١)-C(١٤)-C(١٢)	١٢٠,٠٢٥
C(١٥)-C(١٤)-C(١٢)	١١٩,٤٤٥	H(٥٠)-O(١٣)-C(١٢)	١٠٩,٤٠٩	C(١٤)-C(١٢)-O(١٣)	١٢٢,٧٨٧
C(١٤)-C(١٢)-C(١١)	١٢٠,٢٤٤	O(١٣)-C(١٢)-C(١١)	١١٦,٩٦٦	H(٤٩)-C(١١)-C(١٢)	١١٨,٤١٦
H(٤٩)-C(١١)-C(١٠)	١٢١,٥٤٠	C(١٢)-C(١١)-C(١٠)	١١٩,٩٨٤	C(١١)-C(١٠)-C(١٦)	١١٩,٨٣١
C(١١)-C(١٠)-C(٩)	١١٩,٩١٦	C(١٦)-C(١٠)-C(٩)	١٢٠,٢٤٤	H(٤٨)-C(٩)-N(١٧)	١٠٨,٢٥٧
H(٤٨)-C(٩)-C(١٠)	١٠٩,٠٧٨	H(٤٨)-C(٩)-P(٢)	١٠٧,٦٧٣	N(١٧)-C(٩)-C(١٠)	١١٠,٧١١
N(١٧)-C(٩)-P(٢)	١١١,٢٩٢	C(١٠)-C(٩)-P(٢)	١٠٩,٧٤٢	H(٤٧)-C(٨)-H(٤٦)	١٠٨,٦١٠
H(٤٧)-C(٨)-H(٤٥)	١٠٨,٧٢٦	H(٤٧)-C(٨)-C(٧)	١١٠,٢٥٩	H(٤٦)-C(٨)-H(٤٥)	١٠٨,٧٠٠
H(٤٦)-C(٨)-C(٧)	١١٠,١٨٣	H(٤٥)-C(٨)-C(٧)	١١٠,٣١٦	H(٤٤)-C(٧)-H(٤٣)	١٠٨,٠٧١
H(٤٤)-C(٧)-C(٨)	١١٢,١١٦	H(٤٤)-C(٧)-O(٦)	١٠٥,٣٩٧	H(٤٣)-C(٧)-C(٨)	١١٠,٨٩٩
H(٤٣)-C(٧)-O(٦)	١١٠,٣٩٤	C(٨)-C(٧)-O(٦)	١٠٩,٨١٩	C(٧)-O(٦)-P(٢)	١٢٣,١٣٢
H(٤٢)-C(٥)-H(٤١)	١٠٨,٨٢٩	H(٤٢)-C(٥)-H(٤٠)	١٠٨,٦١٩	H(٤٢)-C(٥)-C(٤)	١٠٩,٦٤٢
H(٤١)-C(٥)-H(٤٠)	١٠٨,٥٢٢	H(٤١)-C(٥)-C(٤)	١١٠,٦٨٢	H(٤٠)-C(٥)-C(٤)	١١٠,٤٩٨
H(٣٩)-C(٤)-H(٣٨)	١٠٨,٧٢٧	H(٣٩)-C(٤)-C(٥)	١١١,٢٤٤	H(٣٩)-C(٤)-O(٣)	١٠٩,٢٧٨
H(٣٨)-C(٤)-C(٥)	١١١,٧٤٨	H(٣٨)-C(٤)-O(٣)	١٠٤,٤٧٤	C(٥)-C(٤)-O(٣)	١١١,١٢٢
C(٤)-O(٣)-P(٢)	١٢٠,٦٣٣	C(٩)-P(٢)-O(٦)	١٠٦,٠٠٥	C(٩)-P(٢)-O(٣)	١٠٢,٤٣٥
C(٩)-P(٢)-O(١)	١١٣,٦٧٨	O(٦)-P(٢)-O(٣)	١٠٥,٤٣٩	O(٦)-P(٢)-O(١)	١١١,٤١٩
O(٣)-P(٢)-O(١)	١١٦,٨٤٠				

Table S5: Selected bond length (Å) of **2c**.

Bond	Length(Å)	Bond	Length(Å)	Bond	Length(Å)
C(٣٧)-H(٦٣)	١,٠٨٥	C(٣٦)-H(٦٢)	١,٠٨٦	C(٣٦)-C(٣٧)	١,٣٩٥
C(٣٥)-H(٦١)	١,٠٨٦	C(٣٥)-C(٣٦)	١,٣٩٣	C(٣٤)-H(٦٠)	١,٠٨٦
C(٣٤)-C(٣٥)	١,٣٩٧	C(٣٣)-H(٥٩)	١,٠٨٣	C(٣٣)-C(٣٤)	١,٣٩١
C(٣٢)-C(٣٣)	١,٣٩٨	C(٣٢)-C(٣٧)	١,٣٩٩	N(٣١)-C(٣٢)	١,٤٢٠
N(٣٠)-N(٣١)	١,٣٦٢	C(٢٩)-H(٥٨)	١,٠٨٥	C(٢٨)-H(٥٧)	١,٠٨٣
C(٢٨)-C(٢٩)	١,٣٨٩	N(٢٥)-O(٢٧)	١,٢٣٣	N(٢٥)-O(٢٦)	١,٢٣٢
C(٢٤)-C(٢٨)	١,٣٩٣	C(٢٤)-N(٢٥)	١,٤٦٦	C(٢٣)-H(٥٦)	١,٠٨٣
C(٢٣)-C(٢٤)	١,٣٩٥	C(٢٢)-H(٥٥)	١,٠٨٤	C(٢٢)-C(٢٣)	١,٣٨٦
C(٢١)-C(٢٢)	١,٤٠٧	C(٢١)-C(٢٩)	١,٤٠٥	C(٢٠)-N(٣٠)	١,٣٣٦
C(٢٠)-C(٢١)	١,٤٦٨	C(١٩)-H(٥٤)	١,٠٧٧	C(١٩)-C(٢٠)	١,٤١٩
C(١٨)-C(١٩)	١,٣٨٢	C(١٨)-N(٣١)	١,٣٧٧	N(١٧)-H(٥٣)	١,٠١٥
N(١٧)-C(١٨)	١,٣٩٠	C(١٦)-H(٥٢)	١,٠٨٦	C(١٥)-H(٥١)	١,٠٨٦
C(١٥)-C(١٦)	١,٣٩٥	C(١٤)-H(٥٠)	١,٠٨٤	C(١٤)-C(١٥)	١,٣٩٣
C(١٢)-C(١٤)	١,٣٩٤	C(١٢)-Cl(١٣)	١,٧٥٩	C(١١)-H(٤٩)	١,٠٨٥
C(١١)-C(١٢)	١,٣٩١	C(١٠)-C(١١)	١,٤٠٣	C(١٠)-C(١٦)	١,٣٩٦
C(٩)-H(٤٨)	١,٠٩٢	C(٩)-N(١٧)	١,٤٦٨	C(٩)-C(١٠)	١,٥١٧
C(٨)-H(٤٧)	١,٠٩٣	C(٨)-H(٤٦)	١,٠٩٤	C(٨)-H(٤٥)	١,٠٩٦
C(٧)-H(٤٤)	١,٠٩٤	C(٧)-H(٤٣)	١,٠٩٣	C(٧)-C(٨)	١,٥١٨
O(٦)-C(٧)	١,٤٥٧	C(٥)-H(٤٢)	١,٠٩٣	C(٥)-H(٤١)	١,٠٩٦
C(٥)-H(٤٠)	١,٠٩٣	C(٤)-H(٣٩)	١,٠٩٣	C(٤)-H(٣٨)	١,٠٩٥
C(٤)-C(٥)	١,٥١٩	O(٣)-C(٤)	١,٤٥٩	P(٢)-C(٩)	١,٨٥٧
P(٢)-O(٦)	١,٦١٦	P(٢)-O(٣)	١,٦٠٥	O(١)-P(٢)	١,٤٩١

Table S6: Selected bond angle (°) of **2c**.

**Angle**	**(°) Degree**	**Angle**	**(°) Degree**	**Angle**	**Degree (°)**
H(٦٣)-C(٣٧)-C(٣٦)	١٢٠,٥٩٧	H(٦٣)-C(٣٧)-C(٣٢)	١١٩,٩٤٤	C(٣٦)-C(٣٧)-C(٣٢)	١١٩,٤٤٧
H(٦٢)-C(٣٦)-C(٣٧)	١١٩,٤٩٢	H(٦٢)-C(٣٦)-C(٣٥)	١٢٠,٢٠٣	C(٣٧)-C(٣٦)-C(٣٥)	١٢٠,٣٠٠
H(٦١)-C(٣٥)-C(٣٦)	١٢٠,١١٤	H(٦١)-C(٣٥)-C(٣٤)	١٢٠,٠٤٧	C(٣٦)-C(٣٥)-C(٣٤)	١١٩,٨٣٨
H(٦٠)-C(٣٤)-C(٣٥)	١٢٠,٠٥٦	H(٦٠)-C(٣٤)-C(٣٣)	١١٩,٤٩٢	C(٣٥)-C(٣٤)-C(٣٣)	١٢٠,٤٤٦
H(٥٩)-C(٣٣)-C(٣٤)	١٢١,٦٣٣	H(٥٩)-C(٣٣)-C(٣٢)	١١٨,٩٧٤	C(٣٤)-C(٣٣)-C(٣٢)	١١٩,٣٩٣
C(٣٣)-C(٣٢)-C(٣٧)	١٢٠,٥٤٩	C(٣٣)-C(٣٢)-N(٣١)	١١٩,٠٥٦	C(٣٧)-C(٣٢)-N(٣١)	١٢٠,٣٩٠
C(٣٢)-N(٣١)-N(٣٠)	١٢٠,٠٥٥	C(٣٢)-N(٣١)-C(١٨)	١٢٧,٥٢٠	N(٣٠)-N(٣١)-C(١٨)	١١٢,٠٤٠
N(٣١)-N(٣٠)-C(٢٠)	١٠٤,٨٩٨	H(٥٨)-C(٢٩)-C(٢٨)	١١٩,٠٦٣	H(٥٨)-C(٢٩)-C(٢١)	١١٩,٩٤٨
C(٢٨)-C(٢٩)-C(٢١)	١٢٠,٩٨٢	H(٥٧)-C(٢٨)-C(٢٩)	١٢١,٦٣٨	H(٥٧)-C(٢٨)-C(٢٤)	١١٩,٤٢٨
C(٢٩)-C(٢٨)-C(٢٤)	١١٨,٩٣٢	O(٢٧)-N(٢٥)-O(٢٦)	١٢٤,٢٣٦	O(٢٧)-N(٢٥)-C(٢٤)	١١٧,٨٧٧
O(٢٦)-N(٢٥)-C(٢٤)	١١٧,٨٨٧	C(٢٨)-C(٢٤)-N(٢٥)	١١٩,٢١٨	C(٢٨)-C(٢٤)-C(٢٣)	١٢١,٥١٣
N(٢٥)-C(٢٤)-C(٢٣)	١١٩,٢٦٧	H(٥٦)-C(٢٣)-C(٢٤)	١١٩,٤٤٣	H(٥٦)-C(٢٣)-C(٢٢)	١٢١,٥٩٦
C(٢٤)-C(٢٣)-C(٢٢)	١١٨,٩٦٠	H(٥٥)-C(٢٢)-C(٢٣)	١٢٠,٢٧٨	H(٥٥)-C(٢٢)-C(٢١)	١١٨,٧٣٦
C(٢٣)-C(٢٢)-C(٢١)	١٢٠,٩٨٥	C(٢٢)-C(٢١)-C(٢٩)	١١٨,٦٢٧	C(٢٢)-C(٢١)-C(٢٠)	١٢٠,١٦٥
C(٢٩)-C(٢١)-C(٢٠)	١٢١,١٩٣	N(٣٠)-C(٢٠)-C(٢١)	١١٩,٨٧٠	N(٣٠)-C(٢٠)-C(١٩)	١١١,٦٢٨
C(٢١)-C(٢٠)-C(١٩)	١٢٨,٤٨٦	H(٥٤)-C(١٩)-C(٢٠)	١٢٨,٣٤٣	H(٥٤)-C(١٩)-C(١٨)	١٢٦,٦١٠
C(٢٠)-C(١٩)-C(١٨)	١٠٤,٩٩٦	C(١٩)-C(١٨)-N(٣١)	١٠٦,٤١٩	C(١٩)-C(١٨)-N(١٧)	١٣١,٨٥٩
N(٣١)-C(١٨)-N(١٧)	١٢١,٧٢٠	H(٥٣)-N(١٧)-C(١٨)	١١١,٦٨٤	H(٥٣)-N(١٧)-C(٩)	١١٠,٧٤٢
C(١٨)-N(١٧)-C(٩)	١١٩,٢٣٣	H(٥٢)-C(١٦)-C(١٥)	١١٩,٩٦٩	H(٥٢)-C(١٦)-C(١٠)	١١٩,٧٠٥
C(١٥)-C(١٦)-C(١٠)	١٢٠,٣٢٦	H(٥١)-C(١٥)-C(١٦)	١١٩,٨٩٦	H(٥١)-C(١٥)-C(١٤)	١١٩,٤٧٣
C(١٦)-C(١٥)-C(١٤)	١٢٠,٦٣١	H(٥٠)-C(١٤)-C(١٥)	١٢١,٢٢٢	H(٥٠)-C(١٤)-C(١٢)	١٢٠,١٢٤
C(١٥)-C(١٤)-C(١٢)	١١٨,٦٥٣	C(١٤)-C(١٢)-Cl(١٣)	١١٩,٣٦٦	C(١٤)-C(١٢)-C(١١)	١٢١,٥٣٨
Cl(١٣)-C(١٢)-C(١١)	١١٩,٠٩٠	H(٤٩)-C(١١)-C(١٢)	١١٩,٦٠١	H(٤٩)-C(١١)-C(١٠)	١٢٠,٩٠٦
C(١٢)-C(١١)-C(١٠)	١١٩,٤٢٩	C(١١)-C(١٠)-C(١٦)	١١٩,٤٢٠	C(١١)-C(١٠)-C(٩)	١١٩,٨٠٣

C(١٦)-C(١٠)-C(٩)	١٢٠,٧٤١	H(٤٨)-C(٩)-N(١٧)	١٠٩,١٠٤	H(٤٨)-C(٩)-C(١٠)	١٠٩,٦٦٤
H(٤٨)-C(٩)-P(٢)	١٠٨,٤٨٩	N(١٧)-C(٩)-C(١٠)	١١٠,٦٢٤	N(١٧)-C(٩)-P(٢)	١١٠,٢٤٣
C(١٠)-C(٩)-P(٢)	١٠٨,٦٨٣	H(٤٧)-C(٨)-H(٤٦)	١٠٩,١٦٥	H(٤٧)-C(٨)-H(٤٥)	١٠٨,٥٨٣
H(٤٧)-C(٨)-C(٧)	١٠٩,٩٣٦	H(٤٦)-C(٨)-H(٤٥)	١٠٨,٥٤٥	H(٤٦)-C(٨)-C(٧)	١١٠,٥٤٦
H(٤٥)-C(٨)-C(٧)	١١٠,٠٢٤	H(٤٤)-C(٧)-H(٤٣)	١٠٨,٣٤١	H(٤٤)-C(٧)-C(٨)	١١١,١٩٥
H(٤٤)-C(٧)-O(٦)	١٠٩,٨٥٣	H(٤٣)-C(٧)-C(٨)	١١١,٩٨٦	H(٤٣)-C(٧)-O(٦)	١٠٤,٦٤١
C(٨)-C(٧)-O(٦)	١١٠,٦٠٣	C(٧)-O(٦)-P(٢)	١٢٠,٥٣٤	H(٤٢)-C(٥)-H(٤١)	١٠٨,٦٩٣
H(٤٢)-C(٥)-H(٤٠)	١٠٨,٩٧١	H(٤٢)-C(٥)-C(٤)	١١٠,١٧٩	H(٤١)-C(٥)-H(٤٠)	١٠٨,٥٨٠
H(٤١)-C(٥)-C(٤)	١٠٩,٦٩٨	H(٤٠)-C(٥)-C(٤)	١١٠,٦٧٦	H(٣٩)-C(٤)-H(٣٨)	١٠٨,٢٥٣
H(٣٩)-C(٤)-C(٥)	١١١,٨٠٣	H(٣٩)-C(٤)-O(٣)	١٠٤,٥٣١	H(٣٨)-C(٤)-C(٥)	١١١,٤٧٤
H(٣٨)-C(٤)-O(٣)	١٠٩,٥٨٣	C(٥)-C(٤)-O(٣)	١١٠,٩٣٦	C(٤)-O(٣)-P(٢)	١١٩,٩٩٠
C(٩)-P(٢)-O(٦)	١٠٥,٦٧٨	C(٩)-P(٢)-O(٣)	١٠٢,٠٢١	C(٩)-P(٢)-O(١)	١١٢,١٢٧
O(٦)-P(٢)-O(٣)	١٠٢,٩٣١	O(٦)-P(٢)-O(١)	١١٥,٤٧٥	O(٣)-P(٢)-O(١)	١١٧,٠٧٩

Table S7: Selected bond length (Å) of **2d**.

Bond	Length(Å)	Bond	Length(Å)	Bond	Length(Å)
C(٣٩)-H(٦٥)	١,٠٨٤	C(٣٨)-H(٦٤)	١,٠٨٦	C(٣٨)-C(٣٩)	١,٣٩٢
C(٣٧)-H(٦٣)	١,٠٨٦	C(٣٧)-C(٣٨)	١,٣٩٧	C(٣٦)-H(٦٢)	١,٠٨٦
C(٣٦)-C(٣٧)	١,٣٩٥	C(٣٥)-H(٦١)	١,٠٨٦	C(٣٥)-C(٣٦)	١,٣٩٦
C(٣٤)-C(٣٥)	١,٣٩٩	C(٣٤)-C(٣٩)	١,٣٩٧	N(٣٣)-C(٣٤)	١,٤٢٥
N(٣٢)-N(٣٣)	١,٣٦٢	C(٣١)-H(٦٠)	١,٠٨٤	C(٣٠)-H(٥٩)	١,٠٨٢
C(٣٠)-C(٣١)	١,٣٨٩	N(٢٧)-O(٢٩)	١,٢٣٢	N(٢٧)-O(٢٨)	١,٢٣٣
C(٢٦)-C(٣٠)	١,٣٩٣	C(٢٦)-N(٢٧)	١,٤٦٧	C(٢٥)-H(٥٨)	١,٠٨٣
C(٢٥)-C(٢٦)	١,٣٩٥	C(٢٤)-H(٥٧)	١,٠٨٤	C(٢٤)-C(٢٥)	١,٣٨٧
C(٢٣)-C(٢٤)	١,٤٠٨	C(٢٣)-C(٣١)	١,٤٠٥	C(٢٢)-N(٣٢)	١,٣٣٦
C(٢٢)-C(٢٣)	١,٤٦٧	C(٢١)-H(٥٦)	١,٠٧٧	C(٢١)-C(٢٢)	١,٤١٨
C(٢٠)-C(٢١)	١,٣٨٢	C(٢٠)-N(٣٣)	١,٣٧٥	N(١٩)-H(٥٥)	١,٠١٣
N(١٩)-C(٢٠)	١,٣٨٨	C(١٨)-H(٥٤)	١,٠٨٦	C(١٧)-H(٥٣)	١,٠٨٣
C(١٧)-C(١٨)	١,٣٩١	N(١٤)-O(١٦)	١,٢٢٩	N(١٤)-O(١٥)	١,٢٣١
C(١٣)-C(١٧)	١,٣٩٣	C(١٣)-N(١٤)	١,٤٧٤	C(١٢)-H(٥٢)	١,٠٨٣
C(١٢)-C(١٣)	١,٣٩٢	C(١١)-H(٥١)	١,٠٨٥	C(١١)-C(١٢)	١,٣٩٢
C(١٠)-C(١١)	١,٣٩٩	C(١٠)-C(١٨)	١,٤٠٤	C(٩)-H(٥٠)	١,٠٩٣
C(٩)-N(١٩)	١,٤٦٦	C(٩)-C(١٠)	١,٥١٦	C(٨)-H(٤٩)	١,٠٩٥
C(٨)-H(٤٨)	١,٠٩٤	C(٨)-H(٤٧)	١,٠٩٣	C(٧)-H(٤٦)	١,٠٩٣
C(٧)-H(٤٥)	١,٠٩١	C(٧)-C(٨)	١,٥١٧	O(٦)-C(٧)	١,٤٦١
C(٥)-H(٤٤)	١,٠٩٥	C(٥)-H(٤٣)	١,٠٩٣	C(٥)-H(٤٢)	١,٠٩٢
C(٤)-H(٤١)	١,٠٩٣	C(٤)-H(٤٠)	١,٠٩٤	C(٤)-C(٥)	١,٥١٦
O(٣)-C(٤)	١,٤٥٨	P(٢)-C(٩)	١,٨٦٥	P(٢)-O(٦)	١,٦٠٤
P(٢)-O(٣)	١,٦٠٨	O(١)-P(٢)	١,٤٩٤		

Table S8: Selected bond angle (°) of **2d**.

Angle	(°) Degree	Angle	(°) Degree	Angle	(°) Degree
H(٦٥)-C(٣٩)-C(٣٨)	١٢١,٥٥٥	H(٦٥)-C(٣٩)-C(٣٤)	١١٩,١٤١	C(٣٨)-C(٣٩)-C(٣٤)	١١٩,٣٠٢
H(٦٤)-C(٣٨)-C(٣٩)	١١٩,٦١٧	H(٦٤)-C(٣٨)-C(٣٧)	١٢٠,١٠٤	C(٣٩)-C(٣٨)-C(٣٧)	١٢٠,٢٧٨
H(٦٣)-C(٣٧)-C(٣٨)	١١٩,٩٨٩	H(٦٣)-C(٣٧)-C(٣٦)	١٢٠,٠٨٩	C(٣٨)-C(٣٧)-C(٣٦)	١١٩,٩٢٠
H(٦٢)-C(٣٦)-C(٣٧)	١٢٠,٢٤١	H(٦٢)-C(٣٦)-C(٣٥)	١١٩,٢٣٩	C(٣٧)-C(٣٦)-C(٣٥)	١٢٠,٥٠٩
H(٦١)-C(٣٥)-C(٣٦)	١٢٠,٤٢٥	H(٦١)-C(٣٥)-C(٣٤)	١٢٠,٦٣٤	C(٣٦)-C(٣٥)-C(٣٤)	١١٨,٩٣٣
C(٣٥)-C(٣٤)-C(٣٩)	١٢١,٠٤١	C(٣٥)-C(٣٤)-N(٣٣)	١٢٠,٠٣٥	C(٣٩)-C(٣٤)-N(٣٣)	١١٨,٩١٨
C(٣٤)-N(٣٣)-N(٣٢)	١١٩,٨٤٤	C(٣٤)-N(٣٣)-C(٢٠)	١٢٨,١٢٠	N(٣٢)-N(٣٣)-C(٢٠)	١١١,٩٩٦
N(٣٣)-N(٣٢)-C(٢٢)	١٠٤,٩١٠	H(٦٠)-C(٣١)-C(٣٠)	١١٩,٠٨٤	H(٦٠)-C(٣١)-C(٢٣)	١١٩,٩١١
C(٣٠)-C(٣١)-C(٢٣)	١٢٠,٩٩٩	H(٥٩)-C(٣٠)-C(٣١)	١٢١,٦٩٦	H(٥٩)-C(٣٠)-C(٢٦)	١١٩,٤٩١
C(٣١)-C(٣٠)-C(٢٦)	١١٨,٨١٢	O(٢٩)-N(٢٧)-O(٢٨)	١٢٤,٣٨٢	O(٢٩)-N(٢٧)-C(٢٦)	١١٧,٨٥٨
O(٢٨)-N(٢٧)-C(٢٦)	١١٧,٧٥٩	C(٣٠)-C(٢٦)-N(٢٧)	١١٩,٠٩٦	C(٣٠)-C(٢٦)-C(٢٥)	١٢١,٦٥٦
N(٢٧)-C(٢٦)-C(٢٥)	١١٩,٢٤٨	H(٥٨)-C(٢٥)-C(٢٦)	١١٩,٤٤١	H(٥٨)-C(٢٥)-C(٢٤)	١٢١,٦٢٣
C(٢٦)-C(٢٥)-C(٢٤)	١١٨,٩٣٥	H(٥٧)-C(٢٤)-C(٢٥)	١٢٠,٣٠٢	H(٥٧)-C(٢٤)-C(٢٣)	١١٨,٨٥٢
C(٢٥)-C(٢٤)-C(٢٣)	١٢٠,٨٤٥	C(٢٤)-C(٢٣)-C(٣١)	١١٨,٧٥٢	C(٢٤)-C(٢٣)-C(٢٢)	١٢٠,٢٣٧
C(٣١)-C(٢٣)-C(٢٢)	١٢١,٠٠١	N(٣٢)-C(٢٢)-C(٢٣)	١١٩,٩٧٢	N(٣٢)-C(٢٢)-C(٢١)	١١١,٦٠٧
C(٢٣)-C(٢٢)-C(٢١)	١٢٨,٤٠٥	H(٥٦)-C(٢١)-C(٢٢)	١٢٨,٤٩٤	H(٥٦)-C(٢١)-C(٢٠)	١٢٦,٥٦١
C(٢٢)-C(٢١)-C(٢٠)	١٠٤,٩٤٤	C(٢١)-C(٢٠)-N(٣٣)	١٠٦,٥١١	C(٢١)-C(٢٠)-N(١٩)	١٣١,٠٧٥
N(٣٣)-C(٢٠)-N(١٩)	١٢٢,٣٥٥	H(٥٥)-N(١٩)-C(٢٠)	١١٣,٢٧٥	H(٥٥)-N(١٩)-C(٩)	١١٢,٦٣٣
C(٢٠)-N(١٩)-C(٩)	١١٩,٤١٨	H(٥٤)-C(١٨)-C(١٧)	١١٩,٦٢٢	H(٥٤)-C(١٨)-C(١٠)	١١٩,٧٧٢
C(١٧)-C(١٨)-C(١٠)	١٢٠,٥٧٥	H(٥٣)-C(١٧)-C(١٨)	١٢١,٦٦٧	H(٥٣)-C(١٧)-C(١٣)	١١٩,٥٧٩
C(١٨)-C(١٧)-C(١٣)	١١٨,٧٥٢	O(١٦)-N(١٤)-O(١٥)	١٢٤,٨٧٢	O(١٦)-N(١٤)-C(١٣)	١١٧,٦٩٠
O(١٥)-N(١٤)-C(١٣)	١١٧,٤٣٨	C(١٧)-C(١٣)-N(١٤)	١١٩,٠٥٩	C(١٧)-C(١٣)-C(١٢)	١٢١,٩٦٩
N(١٤)-C(١٣)-C(١٢)	١١٨,٩٧٢	H(٥٢)-C(١٢)-C(١٣)	١١٩,٥٨٠	H(٥٢)-C(١٢)-C(١١)	١٢١,٨١٠
C(١٣)-C(١٢)-C(١١)	١١٨,٦١٠	H(٥١)-C(١١)-C(١٢)	١١٩,٤٢٤	H(٥١)-C(١١)-C(١٠)	١١٩,٧٤٠
C(١٢)-C(١١)-C(١٠)	١٢٠,٨٣٦	C(١١)-C(١٠)-C(١٨)	١١٩,٢٥٤	C(١١)-C(١٠)-C(٩)	١٢٠,٣٩٤
C(١٨)-C(١٠)-C(٩)	١٢٠,٣٥٠	H(٥٠)-C(٩)-N(١٩)	١٠٨,٥٦٩	H(٥٠)-C(٩)-C(١٠)	١٠٩,٤١١
H(٥٠)-C(٩)-P(٢)	١٠٧,١٨٢	N(١٩)-C(٩)-C(١٠)	١٠٩,٥٣٣	N(١٩)-C(٩)-P(٢)	١١١,٣٣٦
C(١٠)-C(٩)-P(٢)	١١٠,٧٤٣	H(٤٩)-C(٨)-H(٤٨)	١٠٨,٥٦٣	H(٤٩)-C(٨)-H(٤٧)	١٠٨,٤٣٩
H(٤٩)-C(٨)-C(٧)	١٠٩,٦٧٩	H(٤٨)-C(٨)-H(٤٧)	١٠٩,١٣٢	H(٤٨)-C(٨)-C(٧)	١١٠,٦٤٢
H(٤٧)-C(٨)-C(٧)	١١٠,٣٣٥	H(٤٦)-C(٧)-H(٤٥)	١٠٧,٩٧٥	H(٤٦)-C(٧)-C(٨)	١١١,٥٣٢
H(٤٦)-C(٧)-O(٦)	١٠٩,٤٦٧	H(٤٥)-C(٧)-C(٨)	١١٢,٣٤٨	H(٤٥)-C(٧)-O(٦)	١٠٤,١٩٩
C(٨)-C(٧)-O(٦)	١١١,٠١٩	C(٧)-O(٦)-P(٢)	١٢١,١٣٥	H(٤٤)-C(٥)-H(٤٣)	١٠٨,٤٦٠
H(٤٤)-C(٥)-H(٤٢)	١٠٨,٥٤٥	H(٤٤)-C(٥)-C(٤)	١٠٩,٧٨٩	H(٤٣)-C(٥)-H(٤٢)	١٠٨,٦١٧
H(٤٣)-C(٥)-C(٤)	١١٠,٦٩٦	H(٤٢)-C(٥)-C(٤)	١١٠,٦٧٢	H(٤١)-C(٤)-H(٤٠)	١٠٨,١٧٥
H(٤١)-C(٤)-C(٥)	١١٢,٠٣٥	H(٤١)-C(٤)-O(٣)	١٠٩,٣٦٢	H(٤٠)-C(٤)-C(٥)	١١١,٧٢٦
H(٤٠)-C(٤)-O(٣)	١٠٦,٧٠٦	C(٥)-C(٤)-O(٣)	١٠٨,٦٨١	C(٤)-O(٣)-P(٢)	١٢١,٣٣٨
C(٩)-P(٢)-O(٦)	١٠٣,٩٧١	C(٩)-P(٢)-O(٣)	١٠٤,٣٨٥	C(٩)-P(٢)-O(١)	١١٢,٠٥٥
O(٦)-P(٢)-O(٣)	١٠٢,٣٦٧	O(٦)-P(٢)-O(١)	١١٦,٢٥٩	O(٣)-P(٢)-O(١)	١١٦,٣٠٥
H(٥٣)-C(٩)-C(٤)	١١٩,٥٤٣	C(٨)-C(٩)-C(٤)	١١٩,٢٤٢	H(٥٢)-C(٨)-C(٩)	١١٨,٩٠٨
H(٥٢)-C(٨)-C(٧)	١٢١,٠٦٣	C(٩)-C(٨)-C(٧)	١٢٠,٠٠٨	H(٥١)-C(٧)-C(٨)	١٢٠,١٠٥
H(٥١)-C(٧)-C(٦)	١٢٠,٠٧٦	C(٨)-C(٧)-C(٦)	١١٩,٨١٦	H(٥٠)-C(٦)-C(٧)	١٢٠,٠٤٠
H(٥٠)-C(٦)-C(٥)	١١٩,٠٥٨	C(٧)-C(٦)-C(٥)	١٢٠,٩٠٢	H(٤٩)-C(٥)-C(٦)	١٢١,٧٠٨
H(٤٩)-C(٥)-C(٤)	١٢٠,٢٤٢	C(٦)-C(٥)-C(٤)	١١٨,٠٤٣	C(٥)-C(٤)-C(٩)	١٢١,٩٧١
C(٥)-C(٤)-O(٣)	١٠٨,٦٨١	C(٩)-C(٤)-O(٣)	١١٥,٤٠٧	C(٤)-O(٣)-P(٢)	١٢١,٣٣٨
C(١٧)-P(٢)-O(١٠)	١٠١,٦٤٨	C(١٧)-P(٢)-O(٣)	١٠٧,٦٢١	C(١٧)-P(٢)-O(١)	١١٢,٥٩٣
O(١٠)-P(٢)-O(٣)	٩٧,٠٤٠	O(١٠)-P(٢)-O(١)	١١٧,٨٦٠	O(٣)-P(٢)-O(١)	١١٦,٣٠٥

H(٥٤)-C(٨)-C(٧)	١٢٠,٨٦٥	C(٩)-C(٨)-C(٧)	١٢٠,٠٠٨	H(٥٣)-C(٧)-C(٨)	١٢٠,١٤٤
H(٥٣)-C(٧)-C(٦)	١٢٠,١٥٠	C(٨)-C(٧)-C(٦)	١١٩,٨١٦	H(٥٢)-C(٦)-C(٧)	١٢٠,٠٦٥
H(٥٢)-C(٦)-C(٥)	١١٨,٩٨٧	C(٧)-C(٦)-C(٥)	١٢٠,٩٠٢	H(٥١)-C(٥)-C(٦)	١٢١,٤٦٨
H(٥١)-C(٥)-C(٤)	١٢٠,٤٤٣	C(٦)-C(٥)-C(٤)	١١٨,٠٤٣	C(٥)-C(٤)-C(٩)	١٢١,٩٧١
C(٥)-C(٤)-O(٣)	١٠٨,٦٨١	C(٩)-C(٤)-O(٣)	١١٥,٤٠٧	C(٤)-O(٣)-P(٢)	١٢١,٣٣٨
C(١٧)-P(٢)-O(١٠)	١٠١,٦٤٨	C(١٧)-P(٢)-O(٣)	١٠٧,٦٢١	C(١٧)-P(٢)-O(١)	١١٢,٥٩٣
O(١٠)-P(٢)-O(٣)	٩٧,٠٤٠	O(١٠)-P(٢)-O(١)	١١٧,٨٦٠	O(٣)-P(٢)-O(١)	١١٦,٣٠٥

Table S9: Selected bond length (Å) of **2e**

Bond	Length(Å)	Bond	Length(Å)	Bond	Length(Å)
C(٣٧)-H(٦٣)	١,٠٨٦	C(٣٦)-H(٦٢)	١,٠٨٦	C(٣٦)-C(٣٧)	١,٣٩٥
C(٣٥)-H(٦١)	١,٠٨٦	C(٣٥)-C(٣٦)	١,٣٩٤	C(٣٤)-H(٦٠)	١,٠٨٦
C(٣٤)-C(٣٥)	١,٣٩٧	C(٣٣)-H(٥٩)	١,٠٨٤	C(٣٣)-C(٣٤)	١,٣٩٢
C(٣٢)-C(٣٣)	١,٣٩٧	C(٣٢)-C(٣٧)	١,٣٩٨	N(٣١)-C(٣٢)	١,٤٢٠
N(٣٠)-N(٣١)	١,٣٥٤	C(٢٩)-H(٥٨)	١,٠٨٥	C(٢٨)-H(٥٧)	١,٠٨٣
C(٢٨)-C(٢٩)	١,٣٨٩	N(٢٥)-O(٢٧)	١,٢٣٣	N(٢٥)-O(٢٦)	١,٢٣٣
C(٢٤)-C(٢٨)	١,٣٩٣	C(٢٤)-N(٢٥)	١,٤٦٦	C(٢٣)-H(٥٦)	١,٠٨٣
C(٢٣)-C(٢٤)	١,٣٩٥	C(٢٢)-H(٥٥)	١,٠٨٤	C(٢٢)-C(٢٣)	١,٣٨٦
C(٢١)-C(٢٢)	١,٤٠٨	C(٢١)-C(٢٩)	١,٤٠٦	C(٢٠)-N(٣٠)	١,٣٣٨
C(٢٠)-C(٢١)	١,٤٦٨	C(١٩)-H(٥٤)	١,٠٧٥	C(١٩)-C(٢٠)	١,٤٢٠
C(١٨)-C(١٩)	١,٣٨٠	C(١٨)-N(٣١)	١,٣٨٣	N(١٧)-H(٥٣)	١,٠١٣
N(١٧)-C(١٨)	١,٣٩٩	C(١٦)-H(٥٢)	١,٠٨٥	C(١٥)-H(٥١)	١,٠٨٤
C(١٥)-C(١٦)	١,٣٩٣	C(١٣)-C(١٥)	١,٣٩٤	C(١٣)-Cl(١٤)	١,٧٥٩
C(١٢)-H(٥٠)	١,٠٨٤	C(١٢)-C(١٣)	١,٣٩٣	C(١١)-H(٤٩)	١,٠٨٥
C(١١)-C(١٢)	١,٣٩٣	C(١٠)-C(١١)	١,٤٠٢	C(١٠)-C(١٦)	١,٣٩٩
C(٩)-H(٤٨)	١,٠٩٧	C(٩)-N(١٧)	١,٤٧٧	C(٩)-C(١٠)	١,٥١٨
C(٨)-H(٤٧)	١,٠٩٣	C(٨)-H(٤٦)	١,٠٩٥	C(٨)-H(٤٥)	١,٠٩٥
C(٧)-H(٤٤)	١,٠٩٤	C(٧)-H(٤٣)	١,٠٩٣	C(٧)-C(٨)	١,٥١٨
O(٦)-C(٧)	١,٤٥٥	C(٥)-H(٤٢)	١,٠٩٢	C(٥)-H(٤١)	١,٠٩٥
C(٥)-H(٤٠)	١,٠٩٤	C(٤)-H(٣٩)	١,٠٩٣	C(٤)-H(٣٨)	١,٠٩٥
C(٤)-C(٥)	١,٥٢٠	O(٣)-C(٤)	١,٤٥٤	P(٢)-C(٩)	١,٨٥٢
P(٢)-O(٦)	١,٦٢٠	P(٢)-O(٣)	١,٦١٠	O(١)-P(٢)	١,٤٨٨

Table S10: Selected bond angle (°) of **2e**

Angle	(°) Degree	Angle	(°) Degree	Angle	(°) Degree
H(٦٣)-C(٣٧)-C(٣٦)	١٢٠,٦٤٣	H(٦٣)-C(٣٧)-C(٣٢)	١١٩,٧٩٩	C(٣٦)-C(٣٧)-C(٣٢)	١١٩,٥٢٩
H(٦٢)-C(٣٦)-C(٣٧)	١١٩,٥٧٨	H(٦٢)-C(٣٦)-C(٣٥)	١٢٠,٢٥٨	C(٣٧)-C(٣٦)-C(٣٥)	١٢٠,١٥٢
H(٦١)-C(٣٥)-C(٣٦)	١٢٠,٠٦٦	H(٦١)-C(٣٥)-C(٣٤)	١٢٠,٠٣٢	C(٣٦)-C(٣٥)-C(٣٤)	١١٩,٩٠١
H(٦٠)-C(٣٤)-C(٣٥)	١٢٠,٠٤٥	H(٦٠)-C(٣٤)-C(٣٣)	١١٩,٥٥١	C(٣٥)-C(٣٤)-C(٣٣)	١٢٠,٣٩٨
H(٥٩)-C(٣٣)-C(٣٤)	١٢١,٦٢٩	H(٥٩)-C(٣٣)-C(٣٢)	١١٩,٠٠٣	C(٣٤)-C(٣٣)-C(٣٢)	١١٩,٣٦٨
C(٣٣)-C(٣٢)-C(٣٧)	١٢٠,٦٠٧	C(٣٣)-C(٣٢)-N(٣١)	١١٩,١٩٨	C(٣٧)-C(٣٢)-N(٣١)	١٢٠,١٨٨
C(٣٢)-N(٣١)-N(٣٠)	١٢٠,١٣٣	C(٣٢)-N(٣١)-C(١٨)	١٢٧,٦١٨	N(٣٠)-N(٣١)-C(١٨)	١١٢,٢٣٧
N(٣١)-N(٣٠)-C(٢٠)	١٠٥,٠٢١	H(٥٨)-C(٢٩)-C(٢٨)	١١٩,١٢٨	H(٥٨)-C(٢٩)-C(٢١)	١١٩,٨٦٠
C(٢٨)-C(٢٩)-C(٢١)	١٢١,٠١١	H(٥٧)-C(٢٨)-C(٢٩)	١٢١,٦٤٩	H(٥٧)-C(٢٨)-C(٢٤)	١١٩,٤٧٨
C(٢٩)-C(٢٨)-C(٢٤)	١١٨,٨٧٢	O(٢٧)-N(٢٥)-O(٢٦)	١٢٤,٢٧٣	O(٢٧)-N(٢٥)-C(٢٤)	١١٧,٨٨٦
O(٢٦)-N(٢٥)-C(٢٤)	١١٧,٨٤٠	C(٢٨)-C(٢٤)-N(٢٥)	١١٩,٢٧٠	C(٢٨)-C(٢٤)-C(٢٣)	١٢١,٥٨٥
N(٢٥)-C(٢٤)-C(٢٣)	١١٩,١٤٥	H(٥٦)-C(٢٣)-C(٢٤)	١١٩,٤٧٣	H(٥٦)-C(٢٣)-C(٢٢)	١٢١,٥٥٥
C(٢٤)-C(٢٣)-C(٢٢)	١١٨,٩٧٠	H(٥٥)-C(٢٢)-C(٢٣)	١٢٠,٢٨٠	H(٥٥)-C(٢٢)-C(٢١)	١١٨,٧٦٢
C(٢٣)-C(٢٢)-C(٢١)	١٢٠,٩٥٦	C(٢٢)-C(٢١)-C(٢٩)	١١٨,٥٩٧	C(٢٢)-C(٢١)-C(٢٠)	١٢٠,٠٦٠
C(٢٩)-C(٢١)-C(٢٠)	١٢١,٣٣٨	N(٣٠)-C(٢٠)-C(٢١)	١١٩,٧٣٣	N(٣٠)-C(٢٠)-C(١٩)	١١١,٤٣١
C(٢١)-C(٢٠)-C(١٩)	١٢٨,٧٩٧	H(٥٤)-C(١٩)-C(٢٠)	١٢٩,٤٣٢	H(٥٤)-C(١٩)-C(١٨)	١٢٥,٣٤٧
C(٢٠)-C(١٩)-C(١٨)	١٠٥,٢٢٠	C(١٩)-C(١٨)-N(٣١)	١٠٦,٠٨٨	C(١٩)-C(١٨)-N(١٧)	١٣١,٩١٤
N(٣١)-C(١٨)-N(١٧)	١٢١,٩٩٣	H(٥٣)-N(١٧)-C(١٨)	١٠٩,٧٣٦	H(٥٣)-N(١٧)-C(٩)	١٠٨,١٨٣
C(١٨)-N(١٧)-C(٩)	١١٧,٧٣٤	H(٥٢)-C(١٦)-C(١٥)	١١٩,٤٠٤	H(٥٢)-C(١٦)-C(١٠)	١١٩,٦٧٩
C(١٥)-C(١٦)-C(١٠)	١٢٠,٩١١	H(٥١)-C(١٥)-C(١٦)	١٢٠,٧٤٧	H(٥١)-C(١٥)-C(١٣)	١٢٠,٠٤٩
C(١٦)-C(١٥)-C(١٣)	١١٩,٢٠٤	C(١٥)-C(١٣)-Cl(١٤)	١١٩,٥٣٥	C(١٥)-C(١٣)-C(١٢)	١٢١,٠١٩
Cl(١٤)-C(١٣)-C(١٢)	١١٩,٤٤٦	H(٥٠)-C(١٢)-C(١٣)	١٢٠,٠٨٩	H(٥٠)-C(١٢)-C(١١)	١٢٠,٧٧٦
C(١٣)-C(١٢)-C(١١)	١١٩,١٣٠	H(٤٩)-C(١١)-C(١٢)	١١٩,١٤٦	H(٤٩)-C(١١)-C(١٠)	١١٩,٩١٢
C(١٢)-C(١١)-C(١٠)	١٢٠,٩١٩	C(١١)-C(١٠)-C(١٦)	١١٨,٨١١	C(١١)-C(١٠)-C(٩)	١٢٠,٦٤٩
C(١٦)-C(١٠)-C(٩)	١٢٠,٤٧٦	H(٤٨)-C(٩)-N(١٧)	١١٠,٩٨٥	H(٤٨)-C(٩)-C(١٠)	١٠٩,٨١٤
H(٤٨)-C(٩)-P(٢)	١٠٥,٦٦٧	N(١٧)-C(٩)-C(١٠)	١١١,١٨٩	N(١٧)-C(٩)-P(٢)	١١٢,٨٣٣
C(١٠)-C(٩)-P(٢)	١٠٦,٠٩٧	H(٤٧)-C(٨)-H(٤٦)	١٠٩,٢٧٩	H(٤٧)-C(٨)-H(٤٥)	١٠٨,٥٨٩
H(٤٧)-C(٨)-C(٧)	١٠٩,٩٣٩	H(٤٦)-C(٨)-H(٤٥)	١٠٨,٣٥٧	H(٤٦)-C(٨)-C(٧)	١١٠,٥٩١
H(٤٥)-C(٨)-C(٧)	١١٠,٠٤١	H(٤٤)-C(٧)-H(٤٣)	١٠٨,٥٢٣	H(٤٤)-C(٧)-C(٨)	١١١,٠٣٨
H(٤٤)-C(٧)-O(٦)	١٠٩,٧٥٢	H(٤٣)-C(٧)-C(٨)	١١١,٧٧١	H(٤٣)-C(٧)-O(٦)	١٠٤,٥٨٩
C(٨)-C(٧)-O(٦)	١١٠,٩٥١	C(٧)-O(٦)-P(٢)	١١٩,٨٧٢	H(٤٢)-C(٥)-H(٤١)	١٠٩,١٥٥
H(٤٢)-C(٥)-H(٤٠)	١٠٨,٢١٠	H(٤٢)-C(٥)-C(٤)	١١٠,٧٣٣	H(٤١)-C(٥)-H(٤٠)	١٠٨,٥٩٥
H(٤١)-C(٥)-C(٤)	١٠٩,٧٧٧	H(٤٠)-C(٥)-C(٤)	١١٠,٣٢٢	H(٣٩)-C(٤)-H(٣٨)	١٠٧,٩٧٥
H(٣٩)-C(٤)-C(٥)	١١١,٦١٧	H(٣٩)-C(٤)-O(٣)	١٠٤,٦٣٣	H(٣٨)-C(٤)-C(٥)	١١٢,٠٠٥
H(٣٨)-C(٤)-O(٣)	١٠٨,٧٦٧	C(٥)-C(٤)-O(٣)	١١١,٥١١	C(٤)-O(٣)-P(٢)	١٢٠,٢٧٣
C(٩)-P(٢)-O(٦)	١٠٥,٦٦١	C(٩)-P(٢)-O(٣)	١٠٢,١٤٨	C(٩)-P(٢)-O(١)	١١٢,٩٤٣
O(٦)-P(٢)-O(٣)	١٠٢,٣٨٣	O(٦)-P(٢)-O(١)	١١٤,٣٤٣	O(٣)-P(٢)-O(١)	١١٧,٨١٨

Table S11: Selected bond length (Å) of **2f**

Bond	Length(Å)	Bond	Length(Å)	Bond	Length(Å)
N(٤٠)-O(٤٢)	١,٢٣٠	N(٤٠)-O(٤١)	١,٢٢٥	C(٣٩)-N(٤٠)	١,٤٧٥
C(٣٨)-H(٧٣)	١,٠٨١	C(٣٨)-C(٣٩)	١,٣٨٧	N(٣٥)-O(٣٧)	١,٢٣٢
N(٣٥)-O(٣٦)	١,٢٢٩	C(٣٤)-C(٣٨)	١,٣٨٨	C(٣٤)-N(٣٥)	١,٤٧٢
C(٣٣)-H(٧٢)	١,٠٨٢	C(٣٣)-C(٣٤)	١,٣٩٠	C(٣٢)-H(٧١)	١,٠٨٤
C(٣٢)-C(٣٣)	١,٣٩٠	C(٣١)-C(٣٢)	١,٣٩٧	C(٣١)-C(٣٩)	١,٤٠٣
N(٣٠)-C(٣١)	١,٤١٧	N(٢٩)-N(٣٠)	١,٣٦٣	C(٢٨)-H(٧٠)	١,٠٨٥
C(٢٧)-H(٦٩)	١,٠٨٦	C(٢٧)-C(٢٨)	١,٣٩٣	C(٢٦)-H(٦٨)	١,٠٨٦
C(٢٦)-C(٢٧)	١,٣٩٥	C(٢٥)-H(٦٧)	١,٠٨٦	C(٢٥)-C(٢٦)	١,٣٩٧
C(٢٤)-H(٦٦)	١,٠٨٥	C(٢٤)-C(٢٥)	١,٣٩٢	C(٢٣)-C(٢٤)	١,٤٠٤
C(٢٣)-C(٢٨)	١,٤٠٢	C(٢٢)-N(٢٩)	١,٣٣٧	C(٢٢)-C(٢٣)	١,٤٧١
C(٢١)-H(٦٥)	١,٠٧٩	C(٢١)-C(٢٢)	١,٤٢٣	C(٢٠)-C(٢١)	١,٣٧٧
C(٢٠)-N(٣٠)	١,٣٧٤	N(١٩)-H(٦٤)	١,٠١٧	N(١٩)-C(٢٠)	١,٣٩٤
C(١٨)-H(٦٣)	١,٠٨٥	C(١٧)-H(٦٢)	١,٠٨٣	C(١٧)-C(١٨)	١,٣٩٢
C(١٦)-H(٦١)	١,٠٩٩	C(١٦)-H(٦٠)	١,٠٩٧	C(١٦)-H(٥٩)	١,٠٩٠
C(١٥)-H(٥٨)	١,٠٩٠	C(١٥)-H(٥٧)	١,١٠٠	C(١٥)-H(٥٦)	١,٠٩٨
N(١٤)-C(١٦)	١,٤٥٤	N(١٤)-C(١٥)	١,٤٥١	C(١٣)-C(١٧)	١,٤١٥
C(١٣)-N(١٤)	١,٣٧٩	C(١٢)-H(٥٥)	١,٠٨٣	C(١٢)-C(١٣)	١,٤١٧
C(١١)-H(٥٤)	١,٠٨٨	C(١١)-C(١٢)	١,٣٨٨	C(١٠)-C(١١)	١,٣٩٩
C(١٠)-C(١٨)	١,٣٩٦	C(٩)-H(٥٣)	١,٠٩٧	C(٩)-N(١٩)	١,٤٧٤
C(٩)-C(١٠)	١,٥١٧	C(٨)-H(٥٢)	١,٠٩٢	C(٨)-H(٥١)	١,٠٩٣
C(٨)-H(٥٠)	١,٠٩٥	C(٧)-H(٤٩)	١,٠٩٩	C(٧)-H(٤٨)	١,٠٩٣
C(٧)-C(٨)	١,٥١٩	O(٦)-C(٧)	١,٤٥٥	C(٥)-H(٤٧)	١,٠٩٥
C(٥)-H(٤٦)	١,٠٩٤	C(٥)-H(٤٥)	١,٠٩٣	C(٤)-H(٤٤)	١,٠٩٥
C(٤)-H(٤٣)	١,٠٩٤	C(٤)-C(٥)	١,٥١٤	O(٣)-C(٤)	١,٤٥٦
P(٢)-C(٩)	١,٨٧٩	P(٢)-O(٦)	١,٦١٣	P(٢)-O(٣)	١,٦٠٤
O(١)-P(٢)	١,٤٩٢				

Table S12: Selected bond angle (°) of **٢f**

Angle	(°) Degree	Angle	(°) Degree	Angle	(°) Degree
O(٤٢)-N(٤٠)-O(٤١)	١٢٥,٩٣٦	O(٤٢)-N(٤٠)-C(٣٩)	١١٦,٠٣٨	O(٤١)-N(٤٠)-C(٣٩)	١١٨,٠٠٠
N(٤٠)-C(٣٩)-C(٣٨)	١١٦,٥٩٢	N(٤٠)-C(٣٩)-C(٣١)	١٢١,٩٥١	C(٣٨)-C(٣٩)-C(٣١)	١٢١,٤٤٠
H(٧٣)-C(٣٨)-C(٣٩)	١٢٠,٨٢٨	H(٧٣)-C(٣٨)-C(٣٤)	١٢١,١٣٩	C(٣٩)-C(٣٨)-C(٣٤)	١١٨,٠٠٦
O(٣٧)-N(٣٥)-O(٣٦)	١٢٤,٧٢١	O(٣٧)-N(٣٥)-C(٣٤)	١١٧,٤٤٥	O(٣٦)-N(٣٥)-C(٣٤)	١١٧,٨٠٤
C(٣٨)-C(٣٤)-N(٣٥)	١١٨,٤٥٢	C(٣٨)-C(٣٤)-C(٣٣)	١٢٢,٣٤٤	N(٣٥)-C(٣٤)-C(٣٣)	١١٩,١٢٠
H(٧٢)-C(٣٣)-C(٣٤)	١١٩,٨٢٨	H(٧٢)-C(٣٣)-C(٣٢)	١٢١,٦٨٥	C(٣٤)-C(٣٣)-C(٣٢)	١١٨,٤٢٦
H(٧١)-C(٣٢)-C(٣٣)	١٢٠,٦٥٦	H(٧١)-C(٣٢)-C(٣١)	١١٨,٣٠٠	C(٣٣)-C(٣٢)-C(٣١)	١٢٠,٩٦٥
C(٣٢)-C(٣١)-C(٣٩)	١١٨,٦١١	C(٣٢)-C(٣١)-N(٣٠)	١١٩,٣١٧	C(٣٩)-C(٣١)-N(٣٠)	١٢٢,٠٢٦
C(٣١)-N(٣٠)-N(٢٩)	١٢١,٨٧٢	C(٣١)-N(٣٠)-C(٢٠)	١٢٥,٢٣٨	N(٢٩)-N(٣٠)-C(٢٠)	١١٢,٧٣٤
N(٣٠)-N(٢٩)-C(٢٢)	١٠٤,٤٣٥	H(٧٠)-C(٢٨)-C(٢٧)	١١٩,٢٧٠	H(٧٠)-C(٢٨)-C(٢٣)	١٢٠,٠٠١
C(٢٧)-C(٢٨)-C(٢٣)	١٢٠,٧٢٩	H(٦٩)-C(٢٧)-C(٢٨)	١١٩,٦٤٩	H(٦٩)-C(٢٧)-C(٢٦)	١٢٠,١٥٢
C(٢٨)-C(٢٧)-C(٢٦)	١٢٠,١٩٩	H(٦٨)-C(٢٦)-C(٢٧)	١٢٠,٢٣٤	H(٦٨)-C(٢٦)-C(٢٥)	١٢٠,٢٥٥
C(٢٧)-C(٢٦)-C(٢٥)	١١٩,٥١١	H(٦٧)-C(٢٥)-C(٢٦)	١٢٠,٠١٦	H(٦٧)-C(٢٥)-C(٢٤)	١١٩,٦٠٨
C(٢٦)-C(٢٥)-C(٢٤)	١٢٠,٣٧٦	H(٦٦)-C(٢٤)-C(٢٥)	١٢٠,٥٦١	H(٦٦)-C(٢٤)-C(٢٣)	١١٨,٩٠٩
C(٢٥)-C(٢٤)-C(٢٣)	١٢٠,٥٣٠	C(٢٤)-C(٢٣)-C(٢٨)	١١٨,٦٥٦	C(٢٤)-C(٢٣)-C(٢٢)	١٢٠,٥٥٨
C(٢٨)-C(٢٣)-C(٢٢)	١٢٠,٧٨٦	N(٢٩)-C(٢٢)-C(٢٣)	١٢٠,٦٧٦	N(٢٩)-C(٢٢)-C(٢١)	١١١,٤٦٠
C(٢٣)-C(٢٢)-C(٢١)	١٢٧,٨٥٠	H(٦٥)-C(٢١)-C(٢٢)	١٢٩,٠٥٢	H(٦٥)-C(٢١)-C(٢٠)	١٢٥,٦٠٧
C(٢٢)-C(٢١)-C(٢٠)	١٠٥,٣٤١	C(٢١)-C(٢٠)-N(٣٠)	١٠٦,٠٢٥	C(٢١)-C(٢٠)-N(١٩)	١٣٣,٥٦٣
N(٣٠)-C(٢٠)-N(١٩)	١٢٠,٤١١	H(٦٤)-N(١٩)-C(٢٠)	١١٠,٤٦٢	H(٦٤)-N(١٩)-C(٩)	١١٠,٣٩٦
C(٢٠)-N(١٩)-C(٩)	١١٥,١٨٤	H(٦٣)-C(١٨)-C(١٧)	١١٩,٧٤٦	H(٦٣)-C(١٨)-C(١٠)	١١٨,٧٨٧
C(١٧)-C(١٨)-C(١٠)	١٢١,٤٣١	H(٦٢)-C(١٧)-C(١٨)	١١٨,٥٣٥	H(٦٢)-C(١٧)-C(١٣)	١٢٠,٢٤٨
C(١٨)-C(١٧)-C(١٣)	١٢١,٢١٤	H(٦١)-C(١٦)-H(٦٠)	١٠٨,٢٣٣	H(٦١)-C(١٦)-H(٥٩)	١٠٨,١٢٦
H(٦١)-C(١٦)-N(١٤)	١١٢,١٣٠	H(٦٠)-C(١٦)-H(٥٩)	١٠٧,١١١	H(٦٠)-C(١٦)-N(١٤)	١١١,٨٩٤
H(٥٩)-C(١٦)-N(١٤)	١٠٩,١٥٨	H(٥٨)-C(١٥)-H(٥٧)	١٠٧,٨٢٠	H(٥٨)-C(١٥)-H(٥٦)	١٠٧,٧٧٥
H(٥٨)-C(١٥)-N(١٤)	١٠٩,٤٠٧	H(٥٧)-C(١٥)-H(٥٦)	١٠٧,٨٧١	H(٥٧)-C(١٥)-N(١٤)	١١٢,١٧٠
H(٥٦)-C(١٥)-N(١٤)	١١١,٦٢٩	C(١٦)-N(١٤)-C(١٥)	١١٩,٧١١	C(١٦)-N(١٤)-C(١٣)	١٢٠,٣٤٣
C(١٥)-N(١٤)-C(١٣)	١١٩,٩٤٥	C(١٧)-C(١٣)-N(١٤)	١٢١,٧٣٦	C(١٧)-C(١٣)-C(١٢)	١١٦,٩٤٤
N(١٤)-C(١٣)-C(١٢)	١٢١,٣١٥	H(٥٥)-C(١٢)-C(١٣)	١٢٠,٤٨٥	H(٥٥)-C(١٢)-C(١١)	١١٨,٥٤٢
C(١٣)-C(١٢)-C(١١)	١٢٠,٩٧١	H(٥٤)-C(١١)-C(١٢)	١١٨,٧٧٧	H(٥٤)-C(١١)-C(١٠)	١١٩,٤٧٩
C(١٢)-C(١١)-C(١٠)	١٢١,٧٤١	C(١١)-C(١٠)-C(١٨)	١١٧,٦٩٤	C(١١)-C(١٠)-C(٩)	١١٩,٧٨٠
C(١٨)-C(١٠)-C(٩)	١٢٢,٤٧٨	H(٥٣)-C(٩)-N(١٩)	١٠٨,٣٣٣	H(٥٣)-C(٩)-C(١٠)	١٠٧,٥٣٧
H(٥٣)-C(٩)-P(٢)	١٠٣,٤٧٧	N(١٩)-C(٩)-C(١٠)	١١٤,٢٢٨	N(١٩)-C(٩)-P(٢)	١٠٧,٩٦٧
C(١٠)-C(٩)-P(٢)	١١٤,٦٠٠	H(٥٢)-C(٨)-H(٥١)	١٠٨,٦٨٩	H(٥٢)-C(٨)-H(٥٠)	١٠٩,١٠٨
H(٥٢)-C(٨)-C(٧)	١١٠,٣٥٥	H(٥١)-C(٨)-H(٥٠)	١٠٨,٦١٥	H(٥١)-C(٨)-C(٧)	١١٠,٣١١
H(٥٠)-C(٨)-C(٧)	١٠٩,٧٢٥	H(٤٩)-C(٧)-H(٤٨)	١٠٧,٨٠٧	H(٤٩)-C(٧)-C(٨)	١١٠,٨٥٤
H(٤٩)-C(٧)-O(٦)	١١٠,٣٠٦	H(٤٨)-C(٧)-C(٨)	١١١,٦٨٢	H(٤٨)-C(٧)-O(٦)	١٠٤,٦٤١
C(٨)-C(٧)-O(٦)	١١١,٣٣٣	C(٧)-O(٦)-P(٢)	١١٨,٣٨٢	H(٤٧)-C(٥)-H(٤٦)	١٠٨,٥٨٥
H(٤٧)-C(٥)-H(٤٥)	١٠٨,٧٨٢	H(٤٧)-C(٥)-C(٤)	١٠٩,٨٢٥	H(٤٦)-C(٥)-H(٤٥)	١٠٨,٩٦٤
H(٤٦)-C(٥)-C(٤)	١١٠,٥٥٨	H(٤٥)-C(٥)-C(٤)	١١٠,٠٨٦	H(٤٤)-C(٤)-H(٤٣)	١٠٨,٤٣٨
H(٤٤)-C(٤)-C(٥)	١١٠,٥٧٦	H(٤٤)-C(٤)-O(٣)	١٠٩,٨٨٦	H(٤٣)-C(٤)-C(٥)	١١٢,٥١٥
H(٤٣)-C(٤)-O(٣)	١٠٨,٠٢٥	C(٥)-C(٤)-O(٣)	١٠٧,٣٥٣	C(٤)-O(٣)-P(٢)	١١٧,٩٨٨
C(٩)-P(٢)-O(٦)	١٠٤,٩١٠	C(٩)-P(٢)-O(٣)	١٠٨,٣٢١	C(٩)-P(٢)-O(١)	١٠٩,٩٩٠
O(٦)-P(٢)-O(٣)	٩٧,٩٦٣	O(٦)-P(٢)-O(١)	١١٧,٣٩١	O(٣)-P(٢)-O(١)	١١٧,٠٣٣

Table S13: Selected bond length (Å) of **4a**

Bond	Length(Å)	Bond	Length(Å)	Bond	Length(Å)
C(٤٥)-H(٧٢)	١,٠٨٤	C(٤٤)-H(٧١)	١,٠٨٦	C(٤٤)-C(٤٥)	١,٣٩٣
C(٤٣)-H(٧٠)	١,٠٨٦	C(٤٣)-C(٤٤)	١,٣٩٧	C(٤٢)-H(٦٩)	١,٠٨٦
C(٤٢)-C(٤٣)	١,٣٩٥	C(٤١)-H(٦٨)	١,٠٨٧	C(٤١)-C(٤٢)	١,٣٩٥
C(٤٠)-C(٤١)	١,٤٠٠	C(٤٠)-C(٤٥)	١,٣٩٥	N(٣٩)-C(٤٠)	١,٤٢٢
N(٣٨)-N(٣٩)	١,٣٦١	C(٣٧)-H(٦٧)	١,٠٨٣	C(٣٦)-H(٦٦)	١,٠٨٣
C(٣٦)-C(٣٧)	١,٣٨٨	N(٣٣)-O(٣٥)	١,٢٣٣	N(٣٣)-O(٣٤)	١,٢٣٤
C(٣٢)-C(٣٦)	١,٣٩٣	C(٣٢)-N(٣٣)	١,٤٦٥	C(٣١)-H(٦٥)	١,٠٨٣
C(٣١)-C(٣٢)	١,٣٩٦	C(٣٠)-H(٦٤)	١,٠٨٤	C(٣٠)-C(٣١)	١,٣٨٧
C(٢٩)-C(٣٠)	١,٤٠٨	C(٢٩)-C(٣٧)	١,٤٠٥	C(٢٨)-N(٣٨)	١,٣٣٥
C(٢٨)-C(٢٩)	١,٤٦٧	C(٢٧)-H(٦٣)	١,٠٧٨	C(٢٧)-C(٢٨)	١,٤٢٢
C(٢٦)-C(٢٧)	١,٣٨٣	C(٢٦)-N(٣٩)	١,٣٧٢	N(٢٥)-H(٦٢)	١,٠١٢
N(٢٥)-C(٢٦)	١,٣٨٦	C(٢٤)-H(٦١)	١,٠٨٦	C(٢٣)-H(٦٠)	١,٠٨٦
C(٢٣)-C(٢٤)	١,٣٩٤	C(٢٢)-H(٥٩)	١,٠٨٧	C(٢٢)-C(٢٣)	١,٣٩٣
O(٢١)-H(٥٨)	٠,٩٦٦	C(٢٠)-C(٢٢)	١,٤٠١	C(٢٠)-O(٢١)	١,٣٦٤
C(١٩)-H(٥٧)	١,٠٨٦	C(١٩)-C(٢٠)	١,٣٩٥	C(١٨)-C(١٩)	١,٣٩٦
C(١٨)-C(٢٤)	١,٣٩٨	C(١٧)-H(٥٦)	١,٠٩٤	C(١٧)-N(٢٥)	١,٤٤٧
C(١٧)-C(١٨)	١,٥١٤	C(١٦)-H(٥٥)	١,٠٨٣	C(١٥)-H(٥٤)	١,٠٨٥
C(١٥)-C(١٦)	١,٣٩٤	C(١٤)-H(٥٣)	١,٠٨٦	C(١٤)-C(١٥)	١,٣٩٧
C(١٣)-H(٥٢)	١,٠٨٦	C(١٣)-C(١٤)	١,٣٩٦	C(١٢)-H(٥١)	١,٠٨٥
C(١٢)-C(١٣)	١,٣٩٦	C(١١)-C(١٢)	١,٣٩٣	C(١١)-C(١٦)	١,٣٩٤
O(١٠)-C(١١)	١,٣٩٦	C(٩)-H(٥٠)	١,٠٨٤	C(٨)-H(٤٩)	١,٠٨٦
C(٨)-C(٩)	١,٣٩٥	C(٧)-H(٤٨)	١,٠٨٦	C(٧)-C(٨)	١,٣٩٦
C(٦)-H(٤٧)	١,٠٨٦	C(٦)-C(٧)	١,٣٩٦	C(٥)-H(٤٦)	١,٠٨٤
C(٥)-C(٦)	١,٣٩٤	C(٤)-C(٥)	١,٣٩٢	C(٤)-C(٩)	١,٣٩١
O(٣)-C(٤)	١,٣٩٧	P(٢)-C(١٧)	١,٨٨٨	P(٢)-O(١٠)	١,٦٣٥
P(٢)-O(٣)	١,٦١٥	O(١)-P(٢)	١,٤٨٤		

Table S14: Selected bond angle (°) of **4a**

Angle	(°) Degree	Angle	(°) Degree	Angle	(°) Degree
H(٧٢)-C(٤٥)-C(٤٤)	١٢١,٦٦٩	H(٧٢)-C(٤٥)-C(٤٠)	١١٩,١٧٧	C(٤٤)-C(٤٥)-C(٤٠)	١١٩,١٥٤
H(٧١)-C(٤٤)-C(٤٥)	١١٩,٦٣٩	H(٧١)-C(٤٤)-C(٤٣)	١٢٠,٠٥٧	C(٤٥)-C(٤٤)-C(٤٣)	١٢٠,٣٠١
H(٧٠)-C(٤٣)-C(٤٤)	١١٩,٩٤٩	H(٧٠)-C(٤٣)-C(٤٢)	١٢٠,٠٠٥	C(٤٤)-C(٤٣)-C(٤٢)	١٢٠,٠٤٤
H(٦٩)-C(٤٢)-C(٤٣)	١٢٠,٢٣١	H(٦٩)-C(٤٢)-C(٤١)	١١٩,٤٤٦	C(٤٣)-C(٤٢)-C(٤١)	١٢٠,٣١١
H(٦٨)-C(٤١)-C(٤٢)	١٢١,٥٦٥	H(٦٨)-C(٤١)-C(٤٠)	١١٩,٤١٤	C(٤٢)-C(٤١)-C(٤٠)	١١٨,٩٩٧
C(٤١)-C(٤٠)-C(٤٥)	١٢١,١٦٢	C(٤١)-C(٤٠)-N(٣٩)	١١٩,٠٣١	C(٤٥)-C(٤٠)-N(٣٩)	١١٩,٨٠٦
C(٤٠)-N(٣٩)-N(٣٨)	١٢٠,٧٨٤	C(٤٠)-N(٣٩)-C(٢٦)	١٢٦,٨٤٩	N(٣٨)-N(٣٩)-C(٢٦)	١١٢,٢٦٦
N(٣٩)-N(٣٨)-C(٢٨)	١٠٤,٦٨٢	H(٦٧)-C(٣٧)-C(٣٦)	١١٩,١٤٢	H(٦٧)-C(٣٧)-C(٢٩)	١١٩,٨٦٢
C(٣٦)-C(٣٧)-C(٢٩)	١٢٠,٩٨٠	H(٦٦)-C(٣٦)-C(٣٧)	١٢١,٦٠٩	H(٦٦)-C(٣٦)-C(٣٢)	١١٩,٤٢٠
C(٣٧)-C(٣٦)-C(٣٢)	١١٨,٩٦٩	O(٣٥)-N(٣٣)-O(٣٤)	١٢٤,٢٩٧	O(٣٥)-N(٣٣)-C(٣٢)	١١٧,٨٦٨
O(٣٤)-N(٣٣)-C(٣٢)	١١٧,٨٣٥	C(٣٦)-C(٣٢)-N(٣٣)	١١٩,٢٤٥	C(٣٦)-C(٣٢)-C(٣١)	١٢١,٤٧١
N(٣٣)-C(٣٢)-C(٣١)	١١٩,٢٨٣	H(٦٥)-C(٣١)-C(٣٢)	١١٩,٤٣٣	H(٦٥)-C(٣١)-C(٣٠)	١٢١,٥٦٥
C(٣٢)-C(٣١)-C(٣٠)	١١٩,٠٠١	H(٦٤)-C(٣٠)-C(٣١)	١٢٠,٣٢٣	H(٦٤)-C(٣٠)-C(٢٩)	١١٨,٧٦٩
C(٣١)-C(٣٠)-C(٢٩)	١٢٠,٩٠٨	C(٣٠)-C(٢٩)-C(٣٧)	١١٨,٦٦١	C(٣٠)-C(٢٩)-C(٢٨)	١٢٠,٢٥٥
C(٣٧)-C(٢٩)-C(٢٨)	١٢١,٠٧٦	N(٣٨)-C(٢٨)-C(٢٩)	١١٩,٧٥١	N(٣٨)-C(٢٨)-C(٢٧)	١١١,٨١٠
C(٢٩)-C(٢٨)-C(٢٧)	١٢٨,٤٣٣	H(٦٣)-C(٢٧)-C(٢٨)	١٢٨,١٧١	H(٦٣)-C(٢٧)-C(٢٦)	١٢٧,٢١٣
C(٢٨)-C(٢٧)-C(٢٦)	١٠٤,٦٠٤	C(٢٧)-C(٢٦)-N(٣٩)	١٠٦,٦١٢	C(٢٧)-C(٢٦)-N(٢٥)	١٣١,٨٩٠
N(٣٩)-C(٢٦)-N(٢٥)	١٢١,٢٦٩	H(٦٢)-N(٢٥)-C(٢٦)	١١٣,٥٥٩	H(٦٢)-N(٢٥)-C(١٧)	١١٤,٧٣٩
C(٢٦)-N(٢٥)-C(١٧)	١١٩,٧٣٦	H(٦١)-C(٢٤)-C(٢٣)	١٢٠,١٥٢	H(٦١)-C(٢٤)-C(١٨)	١١٩,٩٦٤
C(٢٣)-C(٢٤)-C(١٨)	١١٩,٨٨٤	H(٦٠)-C(٢٣)-C(٢٤)	١١٩,٨٧٨	H(٦٠)-C(٢٣)-C(٢٢)	١١٩,٥٠١
C(٢٤)-C(٢٣)-C(٢٢)	١٢٠,٦٢١	H(٥٩)-C(٢٢)-C(٢٣)	١٢٠,٥٤٦	H(٥٩)-C(٢٢)-C(٢٠)	١٢٠,٠٠٩
C(٢٣)-C(٢٢)-C(٢٠)	١١٩,٤٤٥	H(٥٨)-O(٢١)-C(٢٠)	١٠٩,٥١١	C(٢٢)-C(٢٠)-O(٢١)	١٢٢,٨٥٣
C(٢٢)-C(٢٠)-C(١٩)	١٢٠,١٢٩	O(٢١)-C(٢٠)-C(١٩)	١١٧,٠١٦	H(٥٧)-C(١٩)-C(٢٠)	١١٧,٧١٤
H(٥٧)-C(١٩)-C(١٨)	١٢٢,٠٨٥	C(٢٠)-C(١٩)-C(١٨)	١٢٠,١٧٤	C(١٩)-C(١٨)-C(٢٤)	١١٩,٧٤٥
C(١٩)-C(١٨)-C(١٧)	١٢٠,٥٧٥	C(٢٤)-C(١٨)-C(١٧)	١١٩,٦١٧	H(٥٦)-C(١٧)-N(٢٥)	١٠٧,٧٧١
H(٥٦)-C(١٧)-C(١٨)	١٠٨,٥٩١	H(٥٦)-C(١٧)-P(٢)	١٠٣,٥٥٣	N(٢٥)-C(١٧)-C(١٨)	١١٢,٦٤٦
N(٢٥)-C(١٧)-P(٢)	١١٢,٥٨٨	C(١٨)-C(١٧)-P(٢)	١١١,١٥٧	H(٥٥)-C(١٦)-C(١٥)	١٢١,٨١٠
H(٥٥)-C(١٦)-C(١١)	١١٩,٥٨٨	C(١٥)-C(١٦)-C(١١)	١١٨,٥٩٠	H(٥٤)-C(١٥)-C(١٦)	١١٩,٣٨٥
H(٥٤)-C(١٥)-C(١٤)	١٢٠,٠٠٥	C(١٦)-C(١٥)-C(١٤)	١٢٠,٦٠٧	H(٥٣)-C(١٤)-C(١٥)	١١٩,٩٩٠
H(٥٣)-C(١٤)-C(١٣)	١٢٠,٠٨٠	C(١٥)-C(١٤)-C(١٣)	١١٩,٩٣٠	H(٥٢)-C(١٣)-C(١٤)	١٢٠,٢٣٢
H(٥٢)-C(١٣)-C(١٢)	١١٩,٦٢٢	C(١٤)-C(١٣)-C(١٢)	١٢٠,١٤٦	H(٥١)-C(١٢)-C(١٣)	١٢١,٨٣٦
H(٥١)-C(١٢)-C(١١)	١١٩,١٣٦	C(١٣)-C(١٢)-C(١١)	١١٩,٠١٩	C(١٢)-C(١١)-C(١٦)	١٢١,٧٠٦
C(١٢)-C(١١)-O(١٠)	١١٧,٢٤٧	C(١٦)-C(١١)-O(١٠)	١٢١,٠٢٩	C(١١)-O(١٠)-P(٢)	١٢٠,٩٨٦
H(٥٠)-C(٩)-C(٨)	١٢٢,٠١١	H(٥٠)-C(٩)-C(٤)	١١٩,٢٦٩	C(٨)-C(٩)-C(٤)	١١٨,٧١٩
H(٤٩)-C(٨)-C(٩)	١١٩,٤٧١	H(٤٩)-C(٨)-C(٧)	١٢٠,١٤٢	C(٩)-C(٨)-C(٧)	١٢٠,٣٨٥
H(٤٨)-C(٧)-C(٨)	١٢٠,١١١	H(٤٨)-C(٧)-C(٦)	١٢٠,٠٠٩	C(٨)-C(٧)-C(٦)	١١٩,٨٨٠
H(٤٧)-C(٦)-C(٧)	١٢٠,١٦٧	H(٤٧)-C(٦)-C(٥)	١١٩,٤٢٠	C(٧)-C(٦)-C(٥)	١٢٠,٤١٣
H(٤٦)-C(٥)-C(٦)	١٢١,٤٥٨	H(٤٦)-C(٥)-C(٤)	١١٩,٨٥٤	C(٦)-C(٥)-C(٤)	١١٨,٦٨٦
C(٥)-C(٤)-C(٩)	١٢١,٩١٣	C(٥)-C(٤)-O(٣)	١٢٠,٨١٩	C(٩)-C(٤)-O(٣)	١١٧,٢٣٣
C(٤)-O(٣)-P(٢)	١٢٧,٦٧٦	C(١٧)-P(٢)-O(١٠)	١٠٣,٧٧٠	C(١٧)-P(٢)-O(٣)	١٠٦,٣١٦
C(١٧)-P(٢)-O(١)	١١٣,٨١٨	O(١٠)-P(٢)-O(٣)	١٠٠,٠٨٥	O(١٠)-P(٢)-O(١)	١١٦,٨٧٧
O(٣)-P(٢)-O(١)	١١٤,٤٠٧				

Table S15: Selected bond length (Å) of **4b**

Bond	Length(Å)	Bond	Length(Å)	Bond	Length(Å)
C(٤٧)-H(٧٣)	١,٠٨٤	C(٤٦)-H(٧٢)	١,٠٨٦	C(٤٦)-C(٤٧)	١,٣٩٣
C(٤٥)-H(٧١)	١,٠٨٦	C(٤٥)-C(٤٦)	١,٣٩٧	C(٤٤)-H(٧٠)	١,٠٨٦
C(٤٤)-C(٤٥)	١,٣٩٦	C(٤٣)-H(٦٩)	١,٠٨٣	C(٤٣)-C(٤٤)	١,٣٩٤
C(٤٢)-C(٤٣)	١,٣٩٨	C(٤٢)-C(٤٧)	١,٣٩٨	N(٤١)-C(٤٢)	١,٤٢٢
N(٤٠)-N(٤١)	١,٣٥٧	C(٣٩)-H(٦٨)	١,٠٨٤	C(٣٨)-H(٦٧)	١,٠٨٣
C(٣٨)-C(٣٩)	١,٣٨٩	N(٣٥)-O(٣٧)	١,٢٣٣	N(٣٥)-O(٣٦)	١,٢٣٢
C(٣٤)-C(٣٨)	١,٣٩٣	C(٣٤)-N(٣٥)	١,٤٦٧	C(٣٣)-H(٦٦)	١,٠٨٣
C(٣٣)-C(٣٤)	١,٣٩٥	C(٣٢)-H(٦٥)	١,٠٨٤	C(٣٢)-C(٣٣)	١,٣٨٧
C(٣١)-C(٣٢)	١,٤٠٨	C(٣١)-C(٣٩)	١,٤٠٦	C(٣٠)-N(٤٠)	١,٣٣٧
C(٣٠)-C(٣١)	١,٤٦٧	C(٢٩)-H(٦٤)	١,٠٧٨	C(٢٩)-C(٣٠)	١,٤٢٠
C(٢٨)-C(٢٩)	١,٣٧٦	C(٢٨)-N(٤١)	١,٣٧٨	N(٢٧)-H(٦٣)	١,٠١٦
N(٢٧)-C(٢٨)	١,٤٠٣	C(٢٦)-H(٦٢)	١,٠٨٥	C(٢٥)-H(٦١)	١,٠٨٣
C(٢٥)-C(٢٦)	١,٣٩١	N(٢٢)-O(٢٤)	١,٢٣١	N(٢٢)-O(٢٣)	١,٢٣١
C(٢١)-C(٢٥)	١,٣٩٣	C(٢١)-N(٢٢)	١,٤٧٣	C(٢٠)-H(٦٠)	١,٠٨٣
C(٢٠)-C(٢١)	١,٣٩٣	C(١٩)-H(٥٩)	١,٠٨٦	C(١٩)-C(٢٠)	١,٣٩٠
C(١٨)-C(١٩)	١,٤٠٠	C(١٨)-C(٢٦)	١,٣٩٩	C(١٧)-H(٥٨)	١,٠٩٢
C(١٧)-N(٢٧)	١,٤٧٤	C(١٧)-C(١٨)	١,٥١٠	C(١٦)-H(٥٧)	١,٠٨٣
C(١٥)-H(٥٦)	١,٠٨٥	C(١٥)-C(١٦)	١,٣٩٥	C(١٤)-H(٥٥)	١,٠٨٥
C(١٤)-C(١٥)	١,٣٩٦	C(١٣)-H(٥٤)	١,٠٨٦	C(١٣)-C(١٤)	١,٣٩٥
C(١٢)-H(٥٣)	١,٠٨٥	C(١٢)-C(١٣)	١,٣٩٥	C(١١)-C(١٢)	١,٣٩٢
C(١١)-C(١٦)	١,٣٩٣	O(١٠)-C(١١)	١,٤٠٢	C(٩)-H(٥٢)	١,٠٨٥
C(٨)-H(٥١)	١,٠٨٦	C(٨)-C(٩)	١,٣٩٥	C(٧)-H(٥٠)	١,٠٨٦
C(٧)-C(٨)	١,٣٩٦	C(٦)-H(٤٩)	١,٠٨٦	C(٦)-C(٧)	١,٣٩٦
C(٥)-H(٤٨)	١,٠٨٥	C(٥)-C(٦)	١,٣٩٥	C(٤)-C(٥)	١,٣٩١
C(٤)-C(٩)	١,٣٩٠	O(٣)-C(٤)	١,٤٠٢	P(٢)-C(١٧)	١,٨٧٤
P(٢)-O(١٠)	١,٦٢٨	P(٢)-O(٣)	١,٦١٢	O(١)-P(٢)	١,٤٨٥

Table S16: Selected bond angle (°) of **4b**

Angle	(°) Degree	Angle	(°) Degree	Angle	(°) Degree
H(٧٣)-C(٤٧)-C(٤٦)	١٢١,٧٣٤	H(٧٣)-C(٤٧)-C(٤٢)	١١٨,٨٥٢	C(٤٦)-C(٤٧)-C(٤٢)	١١٩,٤١٤
H(٧٢)-C(٤٦)-C(٤٧)	١١٩,٤٥٣	H(٧٢)-C(٤٦)-C(٤٥)	١٢٠,١٠٤	C(٤٧)-C(٤٦)-C(٤٥)	١٢٠,٤٤١
H(٧١)-C(٤٥)-C(٤٦)	١٢٠,٢١١	H(٧١)-C(٤٥)-C(٤٤)	١٢٠,١٧٤	C(٤٦)-C(٤٥)-C(٤٤)	١١٩,٦١٥
H(٧٠)-C(٤٤)-C(٤٥)	١٢٠,١٦٨	H(٧٠)-C(٤٤)-C(٤٣)	١١٩,٢٥٥	C(٤٥)-C(٤٤)-C(٤٣)	١٢٠,٥٧٦
H(٦٩)-C(٤٣)-C(٤٤)	١٢٠,٩٢٥	H(٦٩)-C(٤٣)-C(٤٢)	١١٩,٧٧٠	C(٤٤)-C(٤٣)-C(٤٢)	١١٩,٢٩٠
C(٤٣)-C(٤٢)-C(٤٧)	١٢٠,٦٥٦	C(٤٣)-C(٤٢)-N(٤١)	١٢٠,٥٧٩	C(٤٧)-C(٤٢)-N(٤١)	١١٨,٧٥٢
C(٤٢)-N(٤١)-N(٤٠)	١١٩,٩٩٤	C(٤٢)-N(٤١)-C(٢٨)	١٢٨,١٠٩	N(٤٠)-N(٤١)-C(٢٨)	١١١,٥٥٦
N(٤١)-N(٤٠)-C(٣٠)	١٠٥,٣٩٦	H(٦٨)-C(٣٩)-C(٣٨)	١١٩,٠٣٦	H(٦٨)-C(٣٩)-C(٣١)	١١٩,٩٠٤
C(٣٨)-C(٣٩)-C(٣١)	١٢١,٠٥٩	H(٦٧)-C(٣٨)-C(٣٩)	١٢١,٦٧٦	H(٦٧)-C(٣٨)-C(٣٤)	١١٩,٤٧٦
C(٣٩)-C(٣٨)-C(٣٤)	١١٨,٨٤٨	O(٣٧)-N(٣٥)-O(٣٦)	١٢٤,٤٥٤	O(٣٧)-N(٣٥)-C(٣٤)	١١٧,٧١٨
O(٣٦)-N(٣٥)-C(٣٤)	١١٧,٨٢٨	C(٣٨)-C(٣٤)-N(٣٥)	١١٩,١٢٨	C(٣٨)-C(٣٤)-C(٣٣)	١٢١,٥٧٨
N(٣٥)-C(٣٤)-C(٣٣)	١١٩,٢٩٣	H(٦٦)-C(٣٣)-C(٣٤)	١١٩,٤٣٣	H(٦٦)-C(٣٣)-C(٣٢)	١٢١,٦٠٠
C(٣٤)-C(٣٣)-C(٣٢)	١١٨,٩٦٦	H(٦٥)-C(٣٢)-C(٣٣)	١٢٠,٢٨٧	H(٦٥)-C(٣٢)-C(٣١)	١١٨,٧٧٨
C(٣٣)-C(٣٢)-C(٣١)	١٢٠,٩٣١	C(٣٢)-C(٣١)-C(٣٩)	١١٨,٦١٣	C(٣٢)-C(٣١)-C(٣٠)	١٢٠,٤٤٧
C(٣٩)-C(٣١)-C(٣٠)	١٢٠,٩٠٦	N(٤٠)-C(٣٠)-C(٣١)	١٢٠,٥٠٨	N(٤٠)-C(٣٠)-C(٢٩)	١١١,٢٢٣
C(٣١)-C(٣٠)-C(٢٩)	١٢٨,١٧٧	H(٦٤)-C(٢٩)-C(٣٠)	١٢٩,٤١٨	H(٦٤)-C(٢٩)-C(٢٨)	١٢٥,٣٢٣
C(٣٠)-C(٢٩)-C(٢٨)	١٠٤,٩٩٧	C(٢٩)-C(٢٨)-N(٤١)	١٠٦,٧٩٩	C(٢٩)-C(٢٨)-N(٢٧)	١٣٠,٩٠٧
N(٤١)-C(٢٨)-N(٢٧)	١٢٢,٢٦٧	H(٦٣)-N(٢٧)-C(٢٨)	١٠٨,١١٥	H(٦٣)-N(٢٧)-C(١٧)	١١٠,٠٠٦
C(٢٨)-N(٢٧)-C(١٧)	١١٤,٤٩١	H(٦٢)-C(٢٦)-C(٢٥)	١١٩,٤٢١	H(٦٢)-C(٢٦)-C(١٨)	١٢٠,٠٢٢
C(٢٥)-C(٢٦)-C(١٨)	١٢٠,٥٥٦	H(٦١)-C(٢٥)-C(٢٦)	١٢١,٧٤١	H(٦١)-C(٢٥)-C(٢١)	١١٩,٤٧٦
C(٢٦)-C(٢٥)-C(٢١)	١١٨,٧٨١	O(٢٤)-N(٢٢)-O(٢٣)	١٢٤,٧٥٤	O(٢٤)-N(٢٢)-C(٢١)	١١٧,٦٥٣
O(٢٣)-N(٢٢)-C(٢١)	١١٧,٥٩٣	C(٢٥)-C(٢١)-N(٢٢)	١١٩,١٣٥	C(٢٥)-C(٢١)-C(٢٠)	١٢١,٩٢٠
N(٢٢)-C(٢١)-C(٢٠)	١١٨,٩٤٤	H(٦٠)-C(٢٠)-C(٢١)	١١٩,٦٢٦	H(٦٠)-C(٢٠)-C(١٩)	١٢١,٨٦٦
C(٢١)-C(٢٠)-C(١٩)	١١٨,٥٠٨	H(٥٩)-C(١٩)-C(٢٠)	١١٩,٥٠١	H(٥٩)-C(١٩)-C(١٨)	١١٩,٦٧٠
C(٢٠)-C(١٩)-C(١٨)	١٢٠,٨٢٨	C(١٩)-C(١٨)-C(٢٦)	١١٩,٣٩٦	C(١٩)-C(١٨)-C(١٧)	١١٩,٠٩٨
C(٢٦)-C(١٨)-C(١٧)	١٢١,٤٥٧	H(٥٨)-C(١٧)-N(٢٧)	١٠٧,٩٦٩	H(٥٨)-C(١٧)-C(١٨)	١٠٨,١٣٦
H(٥٨)-C(١٧)-P(٢)	١٠٤,٣٢٦	N(٢٧)-C(١٧)-C(١٨)	١١٤,١٧٨	N(٢٧)-C(١٧)-P(٢)	١٠٩,٢١٢
C(١٨)-C(١٧)-P(٢)	١١٢,٤٥٣	H(٥٧)-C(١٦)-C(١٥)	١٢١,٩٤٥	H(٥٧)-C(١٦)-C(١١)	١١٩,٤٦٠
C(١٥)-C(١٦)-C(١١)	١١٨,٥٨١	H(٥٦)-C(١٥)-C(١٦)	١١٩,٥٥١	H(٥٦)-C(١٥)-C(١٤)	١١٩,٩٢١
C(١٦)-C(١٥)-C(١٤)	١٢٠,٥٢٢	H(٥٥)-C(١٤)-C(١٥)	١١٩,٨٩٥	H(٥٥)-C(١٤)-C(١٣)	١٢٠,١٥١
C(١٥)-C(١٤)-C(١٣)	١١٩,٩٤٦	H(٥٤)-C(١٣)-C(١٤)	١٢٠,١٨٧	H(٥٤)-C(١٣)-C(١٢)	١١٩,٥٧٢
C(١٤)-C(١٣)-C(١٢)	١٢٠,٢٤١	H(٥٣)-C(١٢)-C(١٣)	١٢١,٦٧٠	H(٥٣)-C(١٢)-C(١١)	١١٩,٤٦٣
C(١٣)-C(١٢)-C(١١)	١١٨,٨٦٦	C(١٢)-C(١١)-C(١٦)	١٢١,٨٤٤	C(١٢)-C(١١)-O(١٠)	١١٧,٤٧٩
C(١٦)-C(١١)-O(١٠)	١٢٠,٦٥٩	C(١١)-O(١٠)-P(٢)	١٢٠,٨٣٨	H(٥٢)-C(٩)-C(٨)	١٢٢,١٠٤
H(٥٢)-C(٩)-C(٤)	١١٩,١٦٦	C(٨)-C(٩)-C(٤)	١١٨,٧٢٩	H(٥١)-C(٨)-C(٩)	١١٩,٥٧٨
H(٥١)-C(٨)-C(٧)	١٢٠,١٦٨	C(٩)-C(٨)-C(٧)	١٢٠,٢٥٣	H(٥٠)-C(٧)-C(٨)	١٢٠,٠٢٩
H(٥٠)-C(٧)-C(٦)	١٢٠,٠٠٢	C(٨)-C(٧)-C(٦)	١١٩,٩٦٩	H(٤٩)-C(٦)-C(٧)	١٢٠,١٢٣
H(٤٩)-C(٦)-C(٥)	١١٩,٤٥٩	C(٧)-C(٦)-C(٥)	١٢٠,٤١٨	H(٤٨)-C(٥)-C(٦)	١٢١,٦٢٧
H(٤٨)-C(٥)-C(٤)	١١٩,٨٥٤	C(٦)-C(٥)-C(٤)	١١٨,٥١٨	C(٥)-C(٤)-C(٩)	١٢٢,١٠٥
C(٥)-C(٤)-O(٣)	١٢٠,٧٩٣	C(٩)-C(٤)-O(٣)	١١٧,٠٨٢	C(٤)-O(٣)-P(٢)	١٢٤,٩٣٧
C(١٧)-P(٢)-O(١٠)	١٠٥,٢١٥	C(١٧)-P(٢)-O(٣)	١٠٤,٨٢٠	C(١٧)-P(٢)-O(١)	١١١,٨٦٢
O(١٠)-P(٢)-O(٣)	٩٩,٩٣٠	O(١٠)-P(٢)-O(١)	١١٦,٩٠٠	O(٣)-P(٢)-O(١)	١١٦,٥٧٧

Table S17: Selected bond length (Å) of **4c**

**Bond**	**Length(Å)**	**Bond**	**Length(Å)**	**Bond**	**Length(Å)**
N(٤٦)-O(٤٨)	١,٢٢٥	N(٤٦)-O(٤٧)	١,٢٢٨	C(٤٥)-N(٤٦)	١,٤٧٦
C(٤٤)-H(٧٤)	١,٠٨٢	C(٤٤)-C(٤٥)	١,٣٨٧	N(٤١)-O(٤٣)	١,٢٣٠
N(٤١)-O(٤٢)	١,٢٣١	C(٤٠)-C(٤٤)	١,٣٨٩	C(٤٠)-N(٤١)	١,٤٦٨
C(٣٩)-H(٧٣)	١,٠٨٣	C(٣٩)-C(٤٠)	١,٣٩٣	C(٣٨)-H(٧٢)	١,٠٨٣
C(٣٨)-C(٣٩)	١,٣٨٧	C(٣٧)-C(٣٨)	١,٤٠٦	C(٣٧)-C(٤٥)	١,٤١٠
N(٣٦)-C(٣٧)	١,٣٩٥	N(٣٥)-N(٣٦)	١,٣٧٨	C(٣٤)-H(٧١)	١,٠٨٥
C(٣٣)-H(٧٠)	١,٠٨٦	C(٣٣)-C(٣٤)	١,٣٩١	C(٣٢)-H(٦٩)	١,٠٨٦
C(٣٢)-C(٣٣)	١,٣٩٧	C(٣١)-H(٦٨)	١,٠٨٦	C(٣١)-C(٣٢)	١,٣٩٥
C(٣٠)-H(٦٧)	١,٠٨٥	C(٣٠)-C(٣١)	١,٣٩٣	C(٢٩)-C(٣٠)	١,٤٠٢
C(٢٩)-C(٣٤)	١,٤٠٣	C(٢٨)-N(٣٥)	١,٣٢٥	C(٢٨)-C(٢٩)	١,٤٦٨
C(٢٧)-H(٦٦)	١,٠٧٧	C(٢٧)-C(٢٨)	١,٤٢٦	C(٢٦)-C(٢٧)	١,٣٧٥
C(٢٦)-N(٣٦)	١,٣٨٨	N(٢٥)-H(٦٥)	١,٠١١	N(٢٥)-C(٢٦)	١,٣٨١
C(٢٤)-H(٦٤)	١,٠٨٥	C(٢٣)-H(٦٣)	١,٠٨٦	C(٢٣)-C(٢٤)	١,٣٩١
C(٢٢)-H(٦٢)	١,٠٨٧	C(٢٢)-C(٢٣)	١,٣٩٦	O(٢١)-H(٦١)	٠,٩٦٦
C(٢٠)-C(٢٢)	١,٣٩٨	C(٢٠)-O(٢١)	١,٣٦٤	C(١٩)-H(٦٠)	١,٠٨٥
C(١٩)-C(٢٠)	١,٣٩٩	C(١٨)-C(١٩)	١,٣٩٥	C(١٨)-C(٢٤)	١,٤٠١
C(١٧)-H(٥٩)	١,٠٩٢	C(١٧)-N(٢٥)	١,٤٥١	C(١٧)-C(١٨)	١,٥١٧
C(١٦)-H(٥٨)	١,٠٨٥	C(١٥)-H(٥٧)	١,٠٨٦	C(١٥)-C(١٦)	١,٣٩٥
C(١٤)-H(٥٦)	١,٠٨٦	C(١٤)-C(١٥)	١,٣٩٦	C(١٣)-H(٥٥)	١,٠٨٦
C(١٣)-C(١٤)	١,٣٩٦	C(١٢)-H(٥٤)	١,٠٨٤	C(١٢)-C(١٣)	١,٣٩٥
C(١١)-C(١٢)	١,٣٩٠	C(١١)-C(١٦)	١,٣٩١	O(١٠)-C(١١)	١,٣٩٩
C(٩)-H(٥٣)	١,٠٨٢	C(٨)-H(٥٢)	١,٠٨٥	C(٨)-C(٩)	١,٣٩٢
C(٧)-H(٥١)	١,٠٨٥	C(٧)-C(٨)	١,٣٩٦	C(٦)-H(٥٠)	١,٠٨٦
C(٦)-C(٧)	١,٣٩٥	C(٥)-H(٤٩)	١,٠٨٣	C(٥)-C(٦)	١,٣٩٧
C(٤)-C(٥)	١,٣٩٣	C(٤)-C(٩)	١,٣٨٩	O(٣)-C(٤)	١,٤٠٢
P(٢)-C(١٧)	١,٨٧٩	P(٢)-O(١٠)	١,٦٢١	P(٢)-O(٣)	١,٦٢٣
O(١)-P(٢)	١,٤٨٣				

Table S18: Selected bond angle (°) of **4c**

Angle	(°) Degree	Angle	(°) Degree	Angle	(°) Degree
O(٤٨)-N(٤٦)-O(٤٧)	١٢٥,٩١٠	O(٤٨)-N(٤٦)-C(٤٥)	١١٦,٩١٧	O(٤٧)-N(٤٦)-C(٤٥)	١١٧,٠٣٩
N(٤٦)-C(٤٥)-C(٤٤)	١١٥,٧٠٧	N(٤٦)-C(٤٥)-C(٣٧)	١٢٢,٥٨٣	C(٤٤)-C(٤٥)-C(٣٧)	١٢١,٦٢٢
H(٧٤)-C(٤٤)-C(٤٥)	١٢٠,٥٣٤	H(٧٤)-C(٤٤)-C(٤٠)	١٢٠,٨١٤	C(٤٥)-C(٤٤)-C(٤٠)	١١٨,٦٤١
O(٤٣)-N(٤١)-O(٤٢)	١٢٥,٠٣٦	O(٤٣)-N(٤١)-C(٤٠)	١١٧,٦٢٨	O(٤٢)-N(٤١)-C(٤٠)	١١٧,٣٣٦
C(٤٤)-C(٤٠)-N(٤١)	١١٩,١٢٤	C(٤٤)-C(٤٠)-C(٣٩)	١٢١,٥١٩	N(٤١)-C(٤٠)-C(٣٩)	١١٩,٣٥٦
H(٧٣)-C(٣٩)-C(٤٠)	١١٩,٤٩٧	H(٧٣)-C(٣٩)-C(٣٨)	١٢١,٢٥٠	C(٤٠)-C(٣٩)-C(٣٨)	١١٩,٢١٩
H(٧٢)-C(٣٨)-C(٣٩)	١١٩,٩٨٩	H(٧٢)-C(٣٨)-C(٣٧)	١١٨,٩١١	C(٣٩)-C(٣٨)-C(٣٧)	١٢١,٠٥٢
C(٣٨)-C(٣٧)-C(٤٥)	١١٧,٩٠٥	C(٣٨)-C(٣٧)-N(٣٦)	١٢٠,٣٣٨	C(٤٥)-C(٣٧)-N(٣٦)	١٢١,٦٠١
C(٣٧)-N(٣٦)-N(٣٥)	١١٨,٧٢١	C(٣٧)-N(٣٦)-C(٢٦)	١٢٩,٦١٣	N(٣٥)-N(٣٦)-C(٢٦)	١١١,٥٨٦
N(٣٦)-N(٣٥)-C(٢٨)	١٠٤,٩١٣	H(٧١)-C(٣٤)-C(٣٣)	١٢٠,٦٥٢	H(٧١)-C(٣٤)-C(٢٩)	١١٩,٠٠٢
C(٣٣)-C(٣٤)-C(٢٩)	١٢٠,٣٤٦	H(٧٠)-C(٣٣)-C(٣٤)	١١٩,٦٤٠	H(٧٠)-C(٣٣)-C(٣٢)	١٢٠,٠٨٤
C(٣٤)-C(٣٣)-C(٣٢)	١٢٠,٢٧٧	H(٦٩)-C(٣٢)-C(٣٣)	١٢٠,١٤٨	H(٦٩)-C(٣٢)-C(٣١)	١٢٠,١٥١
C(٣٣)-C(٣٢)-C(٣١)	١١٩,٧٠١	H(٦٨)-C(٣١)-C(٣٢)	١٢٠,١٧٠	H(٦٨)-C(٣١)-C(٣٠)	١١٩,٦٥٢
C(٣٢)-C(٣١)-C(٣٠)	١٢٠,١٧٧	H(٦٧)-C(٣٠)-C(٣١)	١١٩,٥٣٩	H(٦٧)-C(٣٠)-C(٢٩)	١١٩,٩٨١
C(٣١)-C(٣٠)-C(٢٩)	١٢٠,٤٤٠	C(٣٠)-C(٢٩)-C(٣٤)	١١٩,٠٥٧	C(٣٠)-C(٢٩)-C(٢٨)	١٢٠,٥٥٣
C(٣٤)-C(٢٩)-C(٢٨)	١٢٠,٣٨٣	N(٣٥)-C(٢٨)-C(٢٩)	١٢٠,٨٥٥	N(٣٥)-C(٢٨)-C(٢٧)	١١١,٨٠٠
C(٢٩)-C(٢٨)-C(٢٧)	١٢٧,٣٤٠	H(٦٦)-C(٢٧)-C(٢٨)	١٢٧,٢٨٤	H(٦٦)-C(٢٧)-C(٢٦)	١٢٧,٠٩٨
C(٢٨)-C(٢٧)-C(٢٦)	١٠٥,٥٠٤	C(٢٧)-C(٢٦)-N(٣٦)	١٠٦,١٩٣	C(٢٧)-C(٢٦)-N(٢٥)	١٣١,٠٧٠
N(٣٦)-C(٢٦)-N(٢٥)	١٢٢,٧٣٧	H(٦٥)-N(٢٥)-C(٢٦)	١١٥,٧٤٧	H(٦٥)-N(٢٥)-C(١٧)	١١٥,٧٣٣
C(٢٦)-N(٢٥)-C(١٧)	١١٨,٨٥٥	H(٦٤)-C(٢٤)-C(٢٣)	١١٩,٤١١	H(٦٤)-C(٢٤)-C(١٨)	١٢٠,٨٢١
C(٢٣)-C(٢٤)-C(١٨)	١١٩,٧٣٣	H(٦٣)-C(٢٣)-C(٢٤)	١١٩,٨٢١	H(٦٣)-C(٢٣)-C(٢٢)	١١٩,٣٦٤
C(٢٤)-C(٢٣)-C(٢٢)	١٢٠,٨١٠	H(٦٢)-C(٢٢)-C(٢٣)	١٢٠,٤٦٩	H(٦٢)-C(٢٢)-C(٢٠)	١٢٠,١٣١
C(٢٣)-C(٢٢)-C(٢٠)	١١٩,٤٠٠	H(٦١)-O(٢١)-C(٢٠)	١٠٩,٥٢٠	C(٢٢)-C(٢٠)-O(٢١)	١٢٣,٠٢٩
C(٢٢)-C(٢٠)-C(١٩)	١٢٠,٠٤٣	O(٢١)-C(٢٠)-C(١٩)	١١٦,٩٢٧	H(٦٠)-C(١٩)-C(٢٠)	١١٨,٥٥٨
H(٦٠)-C(١٩)-C(١٨)	١٢١,٢٠٤	C(٢٠)-C(١٩)-C(١٨)	١٢٠,٢٢٦	C(١٩)-C(١٨)-C(٢٤)	١١٩,٧٨٦
C(١٩)-C(١٨)-C(١٧)	١١٨,٤٣٥	C(٢٤)-C(١٨)-C(١٧)	١٢١,٦٧٩	H(٥٩)-C(١٧)-N(٢٥)	١٠٧,١٥٥
H(٥٩)-C(١٧)-C(١٨)	١٠٧,٦٠٦	H(٥٩)-C(١٧)-P(٢)	١٠٢,٨٠٣	N(٢٥)-C(١٧)-C(١٨)	١١٣,٨٢٥
N(٢٥)-C(١٧)-P(٢)	١١٥,٦٣٣	C(١٨)-C(١٧)-P(٢)	١٠٨,٩٣٢	H(٥٨)-C(١٦)-C(١٥)	١٢١,٨٧٧
H(٥٨)-C(١٦)-C(١١)	١١٩,٤٠٤	C(١٥)-C(١٦)-C(١١)	١١٨,٧١٩	H(٥٧)-C(١٥)-C(١٦)	١١٩,٥٨٩
H(٥٧)-C(١٥)-C(١٤)	١٢٠,١٨٧	C(١٦)-C(١٥)-C(١٤)	١٢٠,٢٢٤	H(٥٦)-C(١٤)-C(١٥)	١١٩,٩٥٧
H(٥٦)-C(١٤)-C(١٣)	١٢٠,٠١٧	C(١٥)-C(١٤)-C(١٣)	١٢٠,٠٢٦	H(٥٥)-C(١٣)-C(١٤)	١٢٠,٠٨٨
H(٥٥)-C(١٣)-C(١٢)	١١٩,٥٤٣	C(١٤)-C(١٣)-C(١٢)	١٢٠,٣٦٨	H(٥٤)-C(١٢)-C(١٣)	١٢٢,٠٦٤
H(٥٤)-C(١٢)-C(١١)	١١٩,٣٣١	C(١٣)-C(١٢)-C(١١)	١١٨,٦٠٤	C(١٢)-C(١١)-C(١٦)	١٢٢,٠٥٨
C(١٢)-C(١١)-O(١٠)	١١٨,٣٨٩	C(١٦)-C(١١)-O(١٠)	١١٩,٥٢١	C(١١)-O(١٠)-P(٢)	١٢١,٧٧٧
H(٥٣)-C(٩)-C(٨)	١٢١,١٧٣	H(٥٣)-C(٩)-C(٤)	١١٩,٥٤٣	C(٨)-C(٩)-C(٤)	١١٩,٢٤٢
H(٥٢)-C(٨)-C(٩)	١١٨,٩٠٨	H(٥٢)-C(٨)-C(٧)	١٢١,٠٦٣	C(٩)-C(٨)-C(٧)	١٢٠,٠٠٨
H(٥١)-C(٧)-C(٨)	١٢٠,١٠٥	H(٥١)-C(٧)-C(٦)	١٢٠,٠٧٦	C(٨)-C(٧)-C(٦)	١١٩,٨١٦
H(٥٠)-C(٦)-C(٧)	١٢٠,٠٤٠	H(٥٠)-C(٦)-C(٥)	١١٩,٠٥٨	C(٧)-C(٦)-C(٥)	١٢٠,٩٠٢
H(٤٩)-C(٥)-C(٦)	١٢١,٧٠٨	H(٤٩)-C(٥)-C(٤)	١٢٠,٢٤٢	C(٦)-C(٥)-C(٤)	١١٨,٠٤٣
C(٥)-C(٤)-C(٩)	١٢١,٩٧١	C(٥)-C(٤)-O(٣)	١٢٢,٦٠٣	C(٩)-C(٤)-O(٣)	١١٥,٤٠٧
C(٤)-O(٣)-P(٢)	١٢٧,٢٢١	C(١٧)-P(٢)-O(١٠)	١٠١,٦٤٨	C(١٧)-P(٢)-O(٣)	١٠٧,٦٢١
C(١٧)-P(٢)-O(١)	١١٢,٥٩٣	O(١٠)-P(٢)-O(٣)	٩٧,٠٤٠	O(١٠)-P(٢)-O(١)	١١٧,٨٦٠
O(٣)-P(٢)-O(١)	١١٧,٨٩٧	H(٥٤)-C(٨)-C(٧)	١٢٠,٨٦٥	C(٩)-C(٨)-C(٧)	١٢٠,٠٠٨
H(٥٣)-C(٧)-C(٨)	١٢٠,١٤٤	H(٥٣)-C(٧)-C(٦)	١٢٠,١٥٠	C(٨)-C(٧)-C(٦)	١١٩,٨١٦
H(٥٢)-C(٦)-C(٧)	١٢٠,٠٦٥	H(٥٢)-C(٦)-C(٥)	١١٨,٩٨٧	C(٧)-C(٦)-C(٥)	١٢٠,٩٠٢
H(٥١)-C(٥)-C(٦)	١٢١,٤٦٨	H(٥١)-C(٥)-C(٤)	١٢٠,٤٤٣	C(٦)-C(٥)-C(٤)	١١٨,٠٤٣
C(٥)-C(٤)-C(٩)	١٢١,٩٧١	C(٥)-C(٤)-O(٣)	١٢٢,٦٠٣	C(٩)-C(٤)-O(٣)	١١٥,٤٠٧
C(٤)-O(٣)-P(٢)	١٢٧,٢٢١	C(١٧)-P(٢)-O(١٠)	١٠١,٦٤٨	C(١٧)-P(٢)-O(٣)	١٠٧,٦٢١
C(١٧)-P(٢)-O(١)	١١٢,٥٩٣	O(١٠)-P(٢)-O(٣)	٩٧,٠٤٠	O(١٠)-P(٢)-O(١)	١١٧,٨٦٠
O(٣)-P(٢)-O(١)	١١٧,٨٩٧				

Table S19: Selected bond length (Å) of **4d**

Bond	Length(Å)	Bond	Length(Å)	Bond	Length(Å)
N(٤٨)-O(٥٠)	١,٢٢٥	N(٤٨)-O(٤٩)	١,٢٢٦	C(٤٧)-N(٤٨)	١,٤٧٦
C(٤٦)-H(٨١)	١,٠٨٢	C(٤٦)-C(٤٧)	١,٣٨٦	N(٤٣)-O(٤٥)	١,٢٣٠
N(٤٣)-O(٤٤)	١,٢٣٠	C(٤٢)-C(٤٦)	١,٣٩٠	C(٤٢)-N(٤٣)	١,٤٦٩
C(٤١)-H(٨٠)	١,٠٨٣	C(٤١)-C(٤٢)	١,٣٩٣	C(٤٠)-H(٧٩)	١,٠٨٣
C(٤٠)-C(٤١)	١,٣٨٧	C(٣٩)-C(٤٠)	١,٤٠٦	C(٣٩)-C(٤٧)	١,٤١٠
N(٣٨)-C(٣٩)	١,٣٩٥	N(٣٧)-N(٣٨)	١,٣٧٩	C(٣٦)-H(٧٨)	١,٠٨٥
C(٣٥)-H(٧٧)	١,٠٨٦	C(٣٥)-C(٣٦)	١,٣٩٢	C(٣٤)-H(٧٦)	١,٠٨٦
C(٣٤)-C(٣٥)	١,٣٩٧	C(٣٣)-H(٧٥)	١,٠٨٦	C(٣٣)-C(٣٤)	١,٣٩٦
C(٣٢)-H(٧٤)	١,٠٨٥	C(٣٢)-C(٣٣)	١,٣٩٣	C(٣١)-C(٣٢)	١,٤٠٣
C(٣١)-C(٣٦)	١,٤٠٣	C(٣٠)-N(٣٧)	١,٣٢٥	C(٣٠)-C(٣١)	١,٤٦٧
C(٢٩)-H(٧٣)	١,٠٧٧	C(٢٩)-C(٣٠)	١,٤٢٥	C(٢٨)-C(٢٩)	١,٣٧٥
C(٢٨)-N(٣٨)	١,٣٨٧	N(٢٧)-H(٧٢)	١,٠١٥	N(٢٧)-C(٢٨)	١,٣٨٨
C(٢٦)-H(٧١)	١,٠٨٦	C(٢٥)-H(٧٠)	١,٠٨٢	C(٢٥)-C(٢٦)	١,٣٨٥
C(٢٤)-H(٦٩)	١,٠٩٨	C(٢٤)-H(٦٨)	١,٠٩٩	C(٢٤)-H(٦٧)	١,٠٩١
C(٢٣)-H(٦٦)	١,٠٩٨	C(٢٣)-H(٦٥)	١,١٠١	C(٢٣)-H(٦٤)	١,٠٩١
N(٢٢)-C(٢٤)	١,٤٥٣	N(٢٢)-C(٢٣)	١,٤٤٩	C(٢١)-C(٢٥)	١,٤١٤
C(٢١)-N(٢٢)	١,٣٨١	C(٢٠)-H(٦٣)	١,٠٨٣	C(٢٠)-C(٢١)	١,٤١٤
C(١٩)-H(٦٢)	١,٠٨٦	C(١٩)-C(٢٠)	١,٣٩٢	C(١٨)-C(١٩)	١,٣٩٥
C(١٨)-C(٢٦)	١,٤٠٢	C(١٧)-H(٦١)	١,٠٩٣	C(١٧)-N(٢٧)	١,٤٥٤
C(١٧)-C(١٨)	١,٥١٥	C(١٦)-H(٦٠)	١,٠٨٢	C(١٥)-H(٥٩)	١,٠٨٦
C(١٥)-C(١٦)	١,٣٩٤	C(١٤)-H(٥٨)	١,٠٨٦	C(١٤)-C(١٥)	١,٣٩٦
C(١٣)-H(٥٧)	١,٠٨٦	C(١٣)-C(١٤)	١,٣٩٦	C(١٢)-H(٥٦)	١,٠٨٣
C(١٢)-C(١٣)	١,٣٩٣	C(١١)-C(١٢)	١,٣٩١	C(١١)-C(١٦)	١,٣٩٥
O(١٠)-C(١١)	١,٣٩٦	C(٩)-H(٥٥)	١,٠٨١	C(٨)-H(٥٤)	١,٠٨٥
C(٨)-C(٩)	١,٣٩٢	C(٧)-H(٥٣)	١,٠٨٦	C(٧)-C(٨)	١,٣٩٦
C(٦)-H(٥٢)	١,٠٨٦	C(٦)-C(٧)	١,٣٩٥	C(٥)-H(٥١)	١,٠٨٢
C(٥)-C(٦)	١,٣٩٨	C(٤)-C(٥)	١,٣٩٣	C(٤)-C(٩)	١,٣٩٠
O(٣)-C(٤)	١,٤٠٢	P(٢)-C(١٧)	١,٨٨٢	P(٢)-O(١٠)	١,٦٢٩
P(٢)-O(٣)	١,٦١٥	O(١)-P(٢)	١,٤٨٥		

Table S20: Selected bond angle (°) of **4d**

Angle	(°) Degree	Angle	(°) Degree	Angle	(°) Degree
O(٥٠)-N(٤٨)-O(٤٩)	١٢٥,٧٥٤	O(٥٠)-N(٤٨)-C(٤٧)	١١٦,٨٧٢	O(٤٩)-N(٤٨)-C(٤٧)	١١٧,٢٣١
N(٤٨)-C(٤٧)-C(٤٦)	١١٥,٦٥٠	N(٤٨)-C(٤٧)-C(٣٩)	١٢٢,٥٩٠	C(٤٦)-C(٤٧)-C(٣٩)	١٢١,٦٥٩
H(٨١)-C(٤٦)-C(٤٧)	١٢٠,٥٠٨	H(٨١)-C(٤٦)-C(٤٢)	١٢٠,٨٣٨	C(٤٧)-C(٤٦)-C(٤٢)	١١٨,٦٤٦
O(٤٥)-N(٤٣)-O(٤٤)	١٢٤,٩٥٨	O(٤٥)-N(٤٣)-C(٤٢)	١١٧,٦٣٦	O(٤٤)-N(٤٣)-C(٤٢)	١١٧,٤٠٦
C(٤٦)-C(٤٢)-N(٤٣)	١١٩,١٢٤	C(٤٦)-C(٤٢)-C(٤١)	١٢١,٤٦٧	N(٤٣)-C(٤٢)-C(٤١)	١١٩,٤٠٩
H(٨٠)-C(٤١)-C(٤٢)	١١٩,٤٧٥	H(٨٠)-C(٤١)-C(٤٠)	١٢١,٢٥٢	C(٤٢)-C(٤١)-C(٤٠)	١١٩,٢٣٨
H(٧٩)-C(٤٠)-C(٤١)	١٢٠,٠٧٢	H(٧٩)-C(٤٠)-C(٣٩)	١١٨,٨٣٥	C(٤١)-C(٤٠)-C(٣٩)	١٢١,٠٤٢
C(٤٠)-C(٣٩)-C(٤٧)	١١٧,٩١٢	C(٤٠)-C(٣٩)-N(٣٨)	١٢٠,٢٩٨	C(٤٧)-C(٣٩)-N(٣٨)	١٢١,٦٢٩
C(٣٩)-N(٣٨)-N(٣٧)	١١٨,٨٧٩	C(٣٩)-N(٣٨)-C(٢٨)	١٢٩,٤٤٢	N(٣٧)-N(٣٨)-C(٢٨)	١١١,٦٠٦
N(٣٨)-N(٣٧)-C(٣٠)	١٠٤,٨٤٥	H(٧٨)-C(٣٦)-C(٣٥)	١٢٠,٧٢٤	H(٧٨)-C(٣٦)-C(٣١)	١١٩,٠٢٢
C(٣٥)-C(٣٦)-C(٣١)	١٢٠,٢٥٤	H(٧٧)-C(٣٥)-C(٣٦)	١١٩,٧٢٠	H(٧٧)-C(٣٥)-C(٣٤)	١٢٠,٠٧٧
C(٣٦)-C(٣٥)-C(٣٤)	١٢٠,٢٠٣	H(٧٦)-C(٣٤)-C(٣٥)	١٢٠,١٠١	H(٧٦)-C(٣٤)-C(٣٣)	١٢٠,٠٨٧
C(٣٥)-C(٣٤)-C(٣٣)	١١٩,٨١١	H(٧٥)-C(٣٣)-C(٣٤)	١٢٠,١٥٨	H(٧٥)-C(٣٣)-C(٣٢)	١١٩,٦٤٧
C(٣٤)-C(٣٣)-C(٣٢)	١٢٠,١٩١	H(٧٤)-C(٣٢)-C(٣٣)	١١٩,٦٩٧	H(٧٤)-C(٣٢)-C(٣١)	١٢٠,٠٢٢
C(٣٣)-C(٣٢)-C(٣١)	١٢٠,٢٣٥	C(٣٢)-C(٣١)-C(٣٦)	١١٩,٣٠١	C(٣٢)-C(٣١)-C(٣٠)	١٢٠,٠٥٠
C(٣٦)-C(٣١)-C(٣٠)	١٢٠,٦٣٠	N(٣٧)-C(٣٠)-C(٣١)	١٢١,٣٤٣	N(٣٧)-C(٣٠)-C(٢٩)	١١١,٨٣٩
C(٣١)-C(٣٠)-C(٢٩)	١٢٦,٧٨٦	H(٧٣)-C(٢٩)-C(٣٠)	١٢٧,٠٤٩	H(٧٣)-C(٢٩)-C(٢٨)	١٢٧,٣٦٩
C(٣٠)-C(٢٩)-C(٢٨)	١٠٥,٤٨٨	C(٢٩)-C(٢٨)-N(٣٨)	١٠٦,٢١٣	C(٢٩)-C(٢٨)-N(٢٧)	١٣١,٢٧٠
N(٣٨)-C(٢٨)-N(٢٧)	١٢٢,٥١٣	H(٧٢)-N(٢٧)-C(٢٨)	١١٣,٦٠٤	H(٧٢)-N(٢٧)-C(١٧)	١١٣,٢٤٠
C(٢٨)-N(٢٧)-C(١٧)	١١٧,٠١١	H(٧١)-C(٢٦)-C(٢٥)	١١٨,٨٨٠	H(٧١)-C(٢٦)-C(١٨)	١١٩,٤٦٥
C(٢٥)-C(٢٦)-C(١٨)	١٢١,٥٧٩	H(٧٠)-C(٢٥)-C(٢٦)	١١٨,٣٩٨	H(٧٠)-C(٢٥)-C(٢١)	١٢٠,٦٠٤
C(٢٦)-C(٢٥)-C(٢١)	١٢٠,٩٦٩	H(٦٩)-C(٢٤)-H(٦٨)	١٠٨,٢٣٨	H(٦٩)-C(٢٤)-H(٦٧)	١٠٧,٧١٢
H(٦٩)-C(٢٤)-N(٢٢)	١١١,٩٦٩	H(٦٨)-C(٢٤)-H(٦٧)	١٠٨,٠٢١	H(٦٨)-C(٢٤)-N(٢٢)	١١١,٤٤٣
H(٦٧)-C(٢٤)-N(٢٢)	١٠٩,٣١٠	H(٦٦)-C(٢٣)-H(٦٥)	١٠٧,٨٤٩	H(٦٦)-C(٢٣)-H(٦٤)	١٠٧,٦٢١
H(٦٦)-C(٢٣)-N(٢٢)	١١١,٥٥٢	H(٦٥)-C(٢٣)-H(٦٤)	١٠٧,٩٩٨	H(٦٥)-C(٢٣)-N(٢٢)	١١٢,٢٦٤
H(٦٤)-C(٢٣)-N(٢٢)	١٠٩,٣٨٦	C(٢٤)-N(٢٢)-C(٢٣)	١١٩,٨٧٠	C(٢٤)-N(٢٢)-C(٢١)	١١٩,٥٧١
C(٢٣)-N(٢٢)-C(٢١)	١١٩,٨٨٣	C(٢٥)-C(٢١)-N(٢٢)	١٢١,١٧٧	C(٢٥)-C(٢١)-C(٢٠)	١١٧,٠٩٢
N(٢٢)-C(٢١)-C(٢٠)	١٢١,٧٢٠	H(٦٣)-C(٢٠)-C(٢١)	١٢٠,٤٤٩	H(٦٣)-C(٢٠)-C(١٩)	١١٨,٤٦٥
C(٢١)-C(٢٠)-C(١٩)	١٢١,٠٤٣	H(٦٢)-C(١٩)-C(٢٠)	١١٨,٣٣٠	H(٦٢)-C(١٩)-C(١٨)	١٢٠,٣٠٦
C(٢٠)-C(١٩)-C(١٨)	١٢١,٣٦١	C(١٩)-C(١٨)-C(٢٦)	١١٧,٧١٨	C(١٩)-C(١٨)-C(١٧)	١٢٣,٤١٤
C(٢٦)-C(١٨)-C(١٧)	١١٨,٥٢١	H(٦١)-C(١٧)-N(٢٧)	١٠٧,٧٨٥	H(٦١)-C(١٧)-C(١٨)	١٠٨,٧٥٥
H(٦١)-C(١٧)-P(٢)	١٠٣,٧٣٦	N(٢٧)-C(١٧)-C(١٨)	١١٤,١٠٨	N(٢٧)-C(١٧)-P(٢)	١١٤,١٤٧
C(١٨)-C(١٧)-P(٢)	١٠٧,٧٠٥	H(٦٠)-C(١٦)-C(١٥)	١٢١,٤٩١	H(٦٠)-C(١٦)-C(١١)	١١٩,٨٩٤
C(١٥)-C(١٦)-C(١١)	١١٨,٦٠٤	H(٥٩)-C(١٥)-C(١٦)	١١٩,١١٢	H(٥٩)-C(١٥)-C(١٤)	١٢٠,١١٠
C(١٦)-C(١٥)-C(١٤)	١٢٠,٧٧٧	H(٥٨)-C(١٤)-C(١٥)	١٢٠,١٧٠	H(٥٨)-C(١٤)-C(١٣)	١٢٠,٢١٠
C(١٥)-C(١٤)-C(١٣)	١١٩,٦١٨	H(٥٧)-C(١٣)-C(١٤)	١٢٠,٢٥٧	H(٥٧)-C(١٣)-C(١٢)	١١٩,٤٢٢
C(١٤)-C(١٣)-C(١٢)	١٢٠,٣١٩	H(٥٦)-C(١٢)-C(١٣)	١٢١,٦٨٦	H(٥٦)-C(١٢)-C(١١)	١١٩,١٠٠
C(١٣)-C(١٢)-C(١١)	١١٩,٢٠١	C(١٢)-C(١١)-C(١٦)	١٢١,٤٧٢	C(١٢)-C(١١)-O(١٠)	١١٦,٥٤٢
C(١٦)-C(١١)-O(١٠)	١٢١,٩١٨	C(١١)-O(١٠)-P(٢)	١٢٥,٥٧٩	H(٥٥)-C(٩)-C(٨)	١٢١,٣٧٠
H(٥٥)-C(٩)-C(٤)	١١٩,٤١٣	C(٨)-C(٩)-C(٤)	١١٩,٢٠٤	H(٥٤)-C(٨)-C(٩)	١١٨,٩٨٢
H(٥٤)-C(٨)-C(٧)	١٢٠,٨٦٥	C(٩)-C(٨)-C(٧)	١٢٠,١٤١	H(٥٣)-C(٧)-C(٨)	١٢٠,١٤٤
H(٥٣)-C(٧)-C(٦)	١٢٠,١٥٠	C(٨)-C(٧)-C(٦)	١١٩,٧٠٥	H(٥٢)-C(٦)-C(٧)	١٢٠,٠٦٥
H(٥٢)-C(٦)-C(٥)	١١٨,٩٨٧	C(٧)-C(٦)-C(٥)	١٢٠,٩٤٨	H(٥١)-C(٥)-C(٦)	١٢١,٤٦٨
H(٥١)-C(٥)-C(٤)	١٢٠,٤٤٣	C(٦)-C(٥)-C(٤)	١١٨,٠٨١	C(٥)-C(٤)-C(٩)	١٢١,٩١٤
C(٥)-C(٤)-O(٣)	١٢٢,٩٩٣	C(٩)-C(٤)-O(٣)	١١٥,٠٦٨	C(٤)-O(٣)-P(٢)	١٢٨,٢٠٠
C(١٧)-P(٢)-O(١٠)	١٠٤,٦٣٢	C(١٧)-P(٢)-O(٣)	١٠٦,٦٥٧	C(١٧)-P(٢)-O(١)	١١٢,١٥١
O(١٠)-P(٢)-O(٣)	٩٥,١٢٩	O(١٠)-P(٢)-O(١)	١١٧,٦٤٨	O(٣)-P(٢)-O(١)	١١٨,٥٥١
